# Supplementary material for: A naturally occurring polyacetylene isolated from carrots promotes health and delays signatures of aging
Source: Nat Commun. 2023 Dec 8;14:8142. doi: 10.1038/s41467-023-43672-7 (PMC10709416; doi:10.1038/s41467-023-43672-7)
Supplement: Supplementary file 1 — Supplementary Information [file 41467_2023_43672_MOESM1_ESM.pdf]

Supplementary Information

A naturally occurring polyacetylene isolated from carrots promotes health and delays signatures of aging

Carolin Thomas [1], Reto Erni [2,3], Jia Yee Wu [1], Fabian Fischer [1,4], Greta Lamers [1], Giovanna Grigolon [1], Sarah J. Mitchell [5], Kim Zarse [1,6], Erick M. Carreira [2 *], Michael Ristow [1,6 *]

[1] Laboratory of Energy Metabolism, Institute of Translational Medicine, Department of Health Sciences and Technology, Swiss Federal Institute (ETH) Zurich, Schorenstrasse 16, 8603 Schwerzenbach, Switzerland

[2] Laboratory of Chemistry and Applied Biosciences, Department of Organic Chemistry, Swiss Federal Institute (ETH) Zurich, Vladimir-Prelog-Weg 1-5/10, 8093 Zurich, Switzerland

[3] Biozentrum, University of Basel, 4056 Basel, Switzerland

[4] CureVac SE, 72076 Tübingen, Germany

[5] Ludwig Princeton Branch, Princeton University, Princeton NJ 08540 USA

[6] Institute of Experimental Endocrinology, Charité Universitätsmedizin Berlin, Berlin, D-10117, Germany

***Email:** carreira@ethz.ch; michael.ristow@charite.de

These authors contributed equally: Carolin Thomas, Reto Erni
These authors jointly supervised this work: Erick M. Carreira, Michael Ristow

Contents:

Supplementary Results	Pages 2 - 7
Supplementary Figures 1 - 16	Pages 8 - 23
Supplementary Tables 1 - 10	Pages 24 - 33
Chemical Synthesis Procedures	Pages 31 - 192
Supplementary References	Pages 193 - 194

Supplementary Results

Asymmetric synthesis of isofalcarintriol and configurational assignment

Retrosynthetically we envisioned a modular approach for **1** (refers to continuous numbering of chemical structures introduced in **Fig. 1c**; also applies to all subsequent labels) wherein alkyne **4a** or **4b** were joined with bromoalkyne **5** at late-stage by Cadiot–Chodkiewicz cross-coupling reaction. A similar disconnection has been successfully applied in the synthesis of various other natural polyacetylenes[1-4]. The plan included introduction of the alkyl side-chain by a cross-metathesis reaction of the corresponding olefins, enabling the independent conjoining of molecular fragments at a late stage. *Anti*-alkyne **4a** and *syn*-alkyne **4b** can be traced back to readily available chiral pool starting materials. Dimethyl tartrate (**6**) would provide access to *anti*-alkyne **4a** via previously reported alkene **7**[5]. *Syn*-alkyne **4b** would be formed from known alkene **S6** obtained in two steps form ribose (**8**)[6]. Bromoalkyne **5** derived from known protected alkynes (*S*)-**9** and (*R*)-**10** obtained by enzymatic resolution of the corresponding racemic propargylic alcohol **9**[7,8].
The syntheses of all four *syn*-1,2-diol containing isofalcarintriol (**1a**,**b**) stereoisomers are depicted in **Fig. 2c**. Alkane **7** was obtained from (−)-dimethyl D-tartrate (**6**) in five steps. Ruthenium-catalyzed cross-metathesis of alkene **7** with excess 1-octene using Grubbs Catalysts 2nd Generation gave rise to pure E-alkene. Silyl deprotection with *n*-Bu_4NF (TBAF) resulted in alcohol **11** in 71% over two steps. Oxidation of the primary alcohol to the corresponding aldehyde by Dess–Martin periodinane (DMP) and subsequent Seyferth–Gilbert homologation using Ohira–Bestmann reagent yielded (4*R*,5*R*)-*anti*-alkyne **4a** in 76% over two steps. The enantiomeric (4*S*,5*S*)-*anti*-alkyne *ent*-**4a** was produced accordingly from (+)-dimethyl L-tartrate (**6**). Both enantiomers of bromoalkyne **5** were prepared from alcohol (*S*)-**9** and acetate (*R*)-**10** respectively. Treatment of alcohol (*S*)-**9** with potassium carbonate (K_2CO_3) in methanol resulted in the desilylated alkyne. Alcohol protection with *t*-butyldimethylsilyl chloride (TBSCl) and imidazole, and bromination of the terminal acetylene using *N*-bromosuccinimide (NBS) and catalytic silver nitrate gave bromoalkyne (*S*)-**5** in 59% yield over three steps. Analogous acetate (*R*)-**10** was converted to bromoalkyne (*R*)-**10** in 38% yield. All combinations of *anti*-alkynes **4a** and *ent*-**4a** with bromoalkynes (*S*)-**10** and (*R*)-**10** were carried out by Cu-catalyzed Cadiot–Chodkiewicz cross-coupling reactions in 61–91% yield. Global deprotection with aq. F_3CCO_2H or aq. HCl gave rise to isofalcarintriol (**1a**,**b**, *ent*-**1a**,**b**) *syn*-1,2-diol stereoisomers in 87–99% yield. Synthesis of the corresponding *anti*-1,2-diols (**1c**,**d**, *ent*-**1c**,**d**) is shown in **Supplementary Fig. 2a**.
Intriguingly, ^1H and ^{13}C-NMR spectra of the diastereomer pairs (3*S*,8*R*,9*R*)-isofalcarintriol (**1a**) and (3*S*,8*S*,9*S*)-isofalcarintriol (**1b**) as well as (3*R*,8*S*,9*S*)-isofalcarintriol (*ent*-**1a**) and (3*R*,8*R*,9*R*)-isofalcarintriol (*ent*-**1b**) respectively were identical and matched the NMR spectra of isolated **1** (**Supplementary Table 3**). This observation can be rationalized by the spatial separation of the stereogenic centers groups by the two alkynes leading to independent spin systems. The positive optical rotation observed in naturally occurring **1** was also seen in **1a** and **1b**, thereby assigning the configuration of the diol as (8*R*,9*R*). To determine the absolute configuration of the 3-OH and optical purity, an enantioselective supercritical fluid chromatography (eSFC) separation method was developed (see **Supplementary Fig. 3a-e**). Authentic **1** was compared with *syn*-1,2-diol containing isofalcarintriol (**1a**,**b**, *ent*-**1a**,**b**) stereoisomers, the identity of **1** was assigned by co-injection with synthetic standards (eSFC traces **Supplementary Fig. 3a-e**). Retention times of **1a** matched with isolated **1**, and no trace of its enantiomer *ent*-**1a** was observed in the sample.

Natural abundance of isofalcarintriol and its spatial distribution in *D. carota* roots

The natural abundance of other falcarinol type polyacetylenes in *D. carota* such as falcarinol, falcarindiol, and falcarindiol-3-acetate depends greatly on the exact carrot variety used[9]. Moreover, polyacetylenes have been reported to be unequally distributed within the carrot root and with highest concentration of polyenes observed in the carrot peel[10,11]. To quantify the natural abundance of isofalcarintriol (**1a**) and its spatial distribution in *D. carota* roots an extraction procedure and a liquid chromatography–mass spectrometry (LC-MS) separation method were developed. As internal standard for direct evaluation of isofalcarintriol (**1a**) content in complex matrices, such as *D. carota* extracts, a stable isotope labeled analog of isofalcarintriol (**1a**) was envisioned and thus led us to the design of isofalcarintriol-1,1,1,2,2-d_5 (**12**). Our modular synthesis

route towards isofalcarintriol (**1a**) conveniently allows for the introduction of the pentadeuterium label at the 1,2-positions via bromo alkene **13**, which was traced back to commercially available bromoethane-d_5 (**14**) (**Supplementary Fig. 4**). With isofalcarintriol-d_5 (**12**) in hand, we set out to investigate the natural abundance of isofalcarintriol (**1a**) and its spatial distribution in the carrot root. Commercial orange table carrots were purchased at a local grocer (Coop Supermarkt Zürich Eleven, Karotten PG, Lot: 7297251197; according to the supplier the carrots were grown by Fehr Gebrüder A & P, 8478 Thalheim an der Thur, Switzerland). The prewashed carrots were separated in different batches at random. All batches were cut into thin slices (~2 mm) employing a food processor, extracted overnight at room temperature with the respective solvent (2 ml/g carrots), namely ethyl acetate, ethanol or pentane. The solid matter was removed by filtration and the resulting extract was dried over Na_2SO_4, filtered and concentrated in vacuo (**Supplementary Table 4, entries 1-3**). Additionally, some of the carrots were dissected resulting in core (~17% w/w), flesh (~57% w/w) and peel (~26% w/w) fractions and subsequently extracted separately with ethyl acetate only (**Supplementary Table 4, entries 4-6**). As internal standard, isofalcarintriol-d_5 (**12**) at 1.0*10-2 mg/ml was employed and quantification was conducted according to standard addition method. Unsurprisingly, extraction by a non-polar solvent like pentane was unable to extract any detectable amount of isofalcarintriol (**1a**) from whole carrots (**Supplementary Table 4, entry 1**). Conversely, extraction using ethanol gave rise to a high amount of a viscous oil containing ~1.7*10-5 mg/mg of isofalcarintriol (**1a**) (**Supplementary Table 4, entry 2**). Ethyl acetate extracts of whole carrots contained ~4.8*10-4 mg/mg of isofalcarintriol (**1a**) and dissection of carrots showed isofalcarintriol (**1a**) to be most abundant in the peel fraction at ~1.2*10-3 mg/mg accounting for approximately 85% of all isofalcarintriol (**1a**) extracted (**Supplementary Table 4, entries 3-6).** The natural abundance of isofalcarintriol (**1a**) was estimated at 3.8–8.9 µg/g of dry weight (assuming 90% water content).

Generally, the total polyacetylenes content in commercial carrots is estimated at 400–3000 µg/g of dry weight depending on isolation protocol and carrot variety used[9,10]. Due to the low abundance of isofalcarintriol (**1a**) in nature, the absence of reported isolations is hardly surprising. Nonetheless, using stable isotope labeled isofalcarintriol-d_5 (**12**) as an internal standard for standard addition enables quantification of isofalcarintriol (**1a**). In addition, the accumulation of isofalcarintriol (**1a**) in the carrot peel is well in line with the spatial distribution other polyacetylenes[10,11]. Regardless, of its abundance isofalcarintriol (**1a**) was shown to be occurring in commercial orange table carrots and as such part of the human diet.

<u>Identification of the pharmacophore and development of pulldown label biotin-isofalcarintriol</u>

After structural confirmation and configurational assignment our attention shifted towards exploring the mode of action isofalcarintriol (**1a**). We set out to identify the molecular target of **1a** by chemical proteomics as such requiring a molecular probe derived from **1a.** To examine the pharmacophore and identify an appropriate site for label attachment a set of truncations and structural analogues were synthesized and tested for NRF2 activation (**Supplementary Fig. 5a-b**). Slight alterations to our modular synthesis route enable quick access to these modified compounds (syntheses and NRF2 activations are outlined **Supplementary Fig. 5a-f**). Firstly, we set out to identify a minimal structure capable of NRF2 activation. Propargylic alcohol fragments (*S*)-**16**, (*R*)-**16**, and terminal alkene (3*S*,8*R*,9*R*)-**17** did not activate NRF2, showcasing the importance of the vicinal diol, and an alkyl side-chain. Although previous experiments (**Fig. 2d** and **Supplementary Fig. 2b**) have shown the importance of the 3*S*-hydroxy group for NRF2 activation, the 3-keto derivate (8*R*,9*R*)-**18** was still able to induce a mild response. Conversely, the configuration of the vicinal diol was largely inconsequential (**Fig. 2d** and **Supplementary Fig. 2b**) and the acetonide protected derivate (3*S*,8*R*,9*R*)-**19** was also able to moderately activate NRF2. Whereas, the 10,11-dihydro analogue (3*S*,8*R*,9*R*)-**20** showed significant NRF2 activation, showcasing the high tolerance for structural diversity around the vicinal diol. The structure activity relationship (SAR) study allowed us to identify the pharmacophore, thereby enabling rational probe design.

Taking this SAR-data into account the design of an activity-based probe for chemical proteomics has resulted in a conjugate, namely biotin-isofalcarintriol (3*S*,8*R*,9*R*)-**15**. The biotin label was attached furthest away from the crucial 3*S*-hydroxy group whilst keeping the rest of the molecule

unchanged. The flexibility of our modular synthesis enabled the convenient incorporation of a biotinylated side-chain. The syntheses biotin-isofalcarintriol (3*S*,8*R*,9*R*)-**15** is depicted in **Supplementary Fig. 6c**. Alkene **22** was prepared from D-biotin ethyl ester (**21**) in a three-step sequence. Selective reduction of ester **21** with diisobutylaluminum hydride (DIBAL) afforded the corresponding aldehyde, which was subjected to Wittig olefination. Subsequent tert-butyloxycarbonyl (Boc) protection of the biotin amines resulted in alkene **22** in 59% from **21**. Employing thioether containing alkenes in cross-metathesis poses challenges as the Lewis basic sulfur interferes with the ruthenium catalyst[12]. Cross-metathesis of alkene (4*R*,5*R*)-**7** with excess **22** using Grubbs Catalyst 2nd Generation did not result in product formation. Whilst reactivity could be achieved by the addition of titanium(IV) iso-propoxide and the use of Hoveyda-Grubbs Catalyst 2nd Generation, concomitant partial displacement of the Boc tert-butyl groups by iso-propyl was observed. Employing titanium(IV) tert-butoxide thwarted this exchange resulting in pure E-alkene **23** in 66% yield. Subsequent Boc deprotection with K_2CO_3 in methanol and silyl deprotection employing TBAF gave alcohol **24** in 78% over two steps. Whilst oxidation of the primary alcohol to the corresponding aldehyde by DMP resulted in the desired aldehyde, concurrent partial sulfur oxidation was observed. Under Pfitzner-Moffat conditions clean conversion to the aldehyde was achieved and subsequent Seyferth–Gilbert homologation using Ohira–Bestmann reagent yielded alkyne **25** in 59% from **24**. Alkyne **25** and bromoalkyne (*S*)-**5** were coupled by selective Cadiot–Chodkiewicz cross-coupling and resulted in acetal **26** in 82% yield. Global deprotection with aq. HCl gave rise to biotin-isofalcarintriol (3*S*,8*R*,9*R*)-**15** in 92% yield. To confirm the bioactivity of biotin-isofalcarintriol (3*S*,8*R*,9*R*)-**15**, we performed a NRF2 activation assay with a HepG2 luciferase reporter cell line (**Supplementary Fig. 6d-e**). While HepG2 cells contain a biotin transporter in their plasma membrane, enabling them to actively take up biotin-labelled compounds, HEK293 lack this ability. Consequently, treatment with biotin-isofalcarintriol (**15**) resulted in NRF2 activation in HepG2 but not HEK293 cells most likely due to impaired cellular uptake. Thereby the alkyl side-chain was identified as a potent site for the incorporation of chemical probes and biotin-isofalcarintriol (3*S*,8*R*,9*R*)-**15** was confirmed as active probe for proteomics.

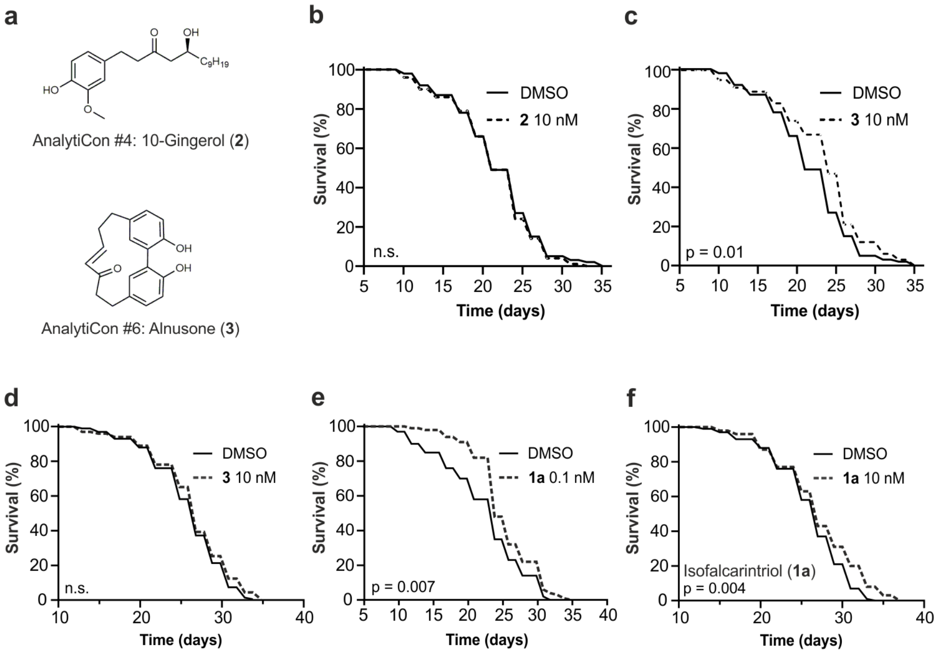

Supplementary Fig. 1. Lifespan assays of *C. elegans* treated with 10-gingerol, alnusone, and isofalcarintriol in different concentrations. a) Structures of 10-gingerol (**2**) and alnusone (**3**). **b)** *C. elegans* lifespan applying 10 nM 10-gingerol (**2**), **c)** 10 nM alnusone (**3**) (first independent experiment) **d)** again 10 nM alnusone (**3**) (second independent experiment), **e)** 0.1 nM isofalcarintriol (**1a**) and **f)** 10 nM isofalcarintriol (**1a**). Data include three biologically independent samples and are represented as average. Statistics: log-rank test; p-values as indicated.

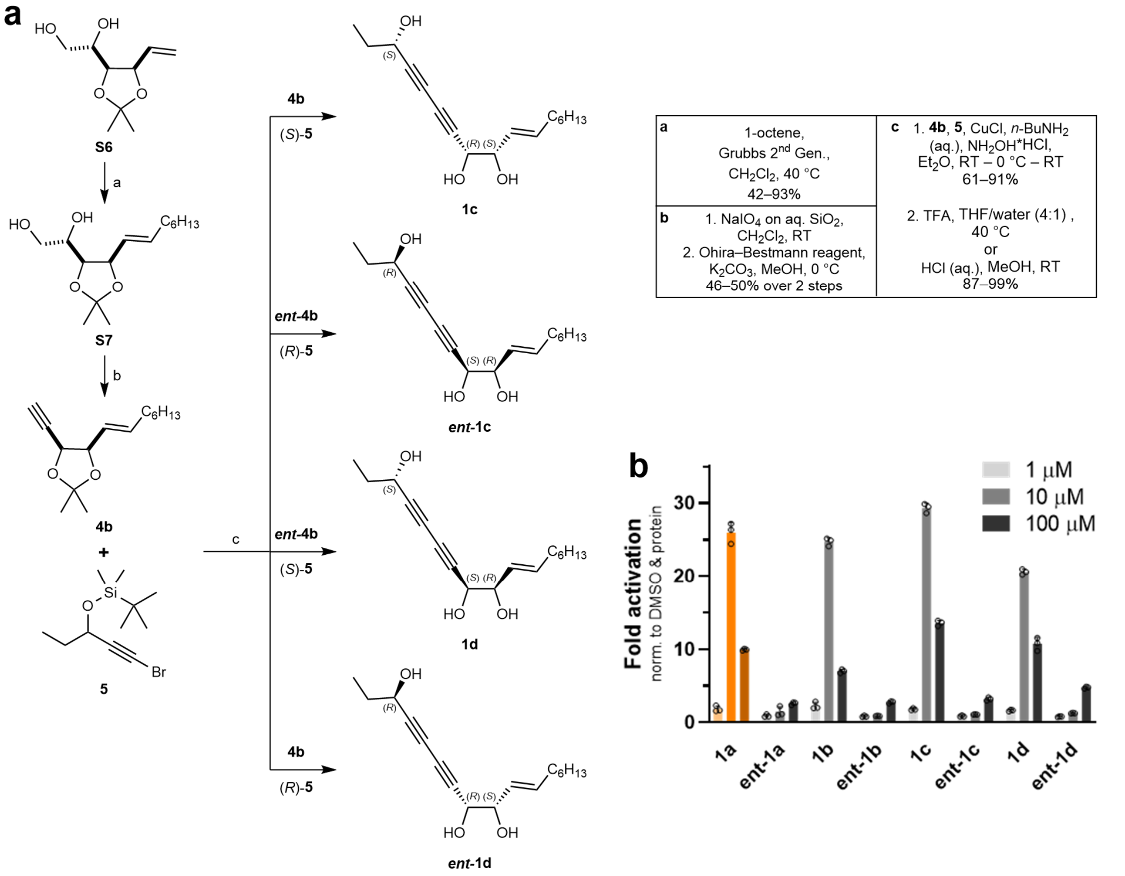

Supplementary Fig. 2. Synthesis of isofalcarintriol stereoisomers and NRF2 activations of all eight stereoisomers. a) Synthesis scheme of all four *anti*-1,2-diol containing isofalcarintriol (**1c,d**) stereoisomers. **b)** NRF2 luciferase reporter assay after overnight treatment in transgenic HEK293 cells where predominantly 3*S* configurated isofalcarintriols (**1a-d**) activated NRF2. Data include three technical replicates and are represented as average + SD.

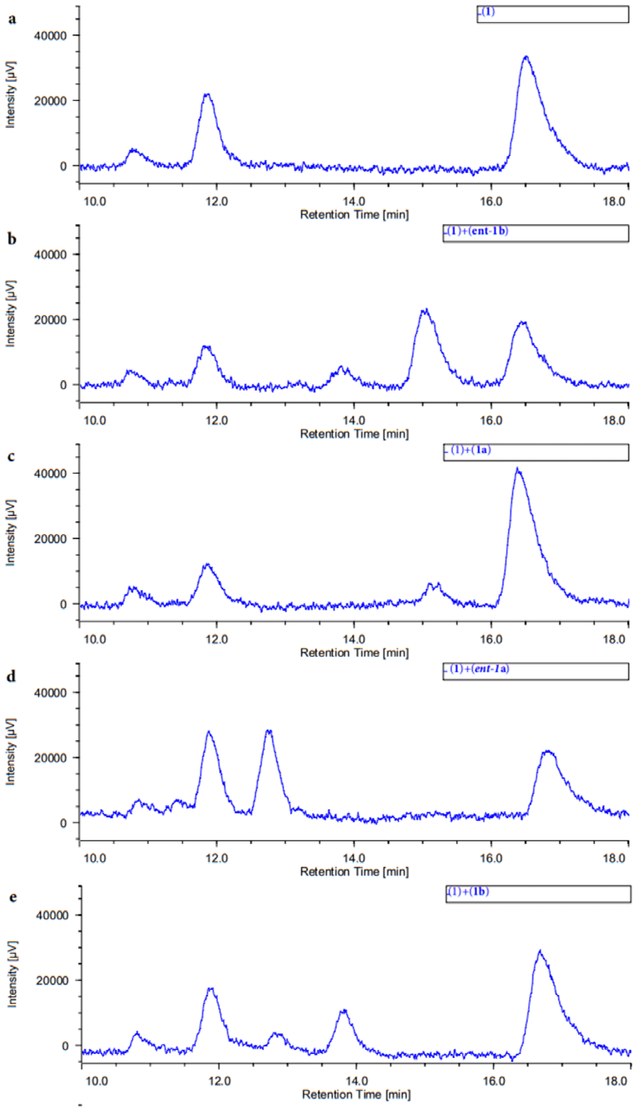

Supplementary Fig. 3. Enantioselective supercritical fluid chromatography (eSFC) traces of isofalcarintriol and its co-injection. a) eSFC trace of **1** showing RT 16.5 min, impurity peak in sample at RT 11.9 min. **b-e)** eSFC trace of 1 co-injected with (3*S*,8*R*,9*R*)-isofalcarintriol (**1a**) (16.5 min), (3*S*,8*S*,9*S*)-isofalcarintriol (**1b**) (13.9 min), (3*R*,8*S*,9*S*)-isofalcarintriol (*ent*-**1a**) (12.8 min), and (3*R*,8*R*,9*R*)-isofalcarintriol (*ent*-**1b**) (15.1 min). Retention times of **1a** matched with authentic **1** and no trace of its enantiomer *ent*-**1a** was observed.

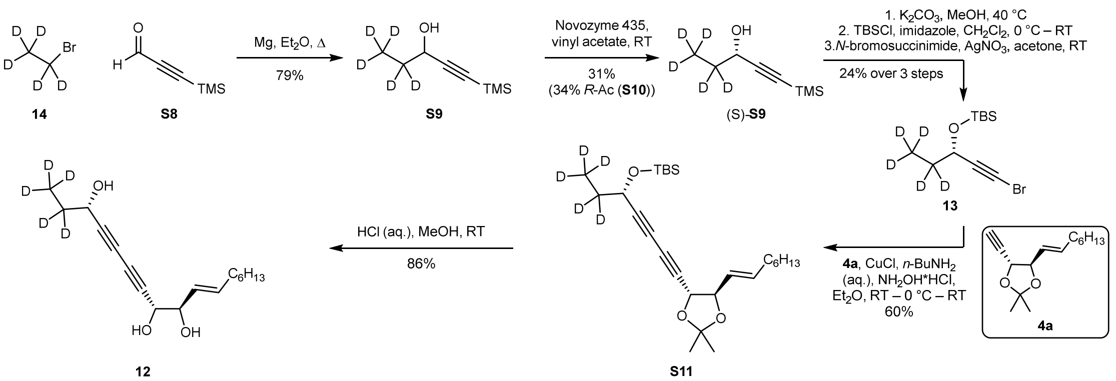

Supplementary Fig. 4. Synthesis of isofalcarintriol-1,1,1,2,2-d$_5$ (12). Synthesis scheme of isofalcarintriol-1,1,1,2,2-d$_5$ (**12**) conveniently enables the introduction of the pentadeuterium label at the 1,2-positions *via* bromo alkene **13**.

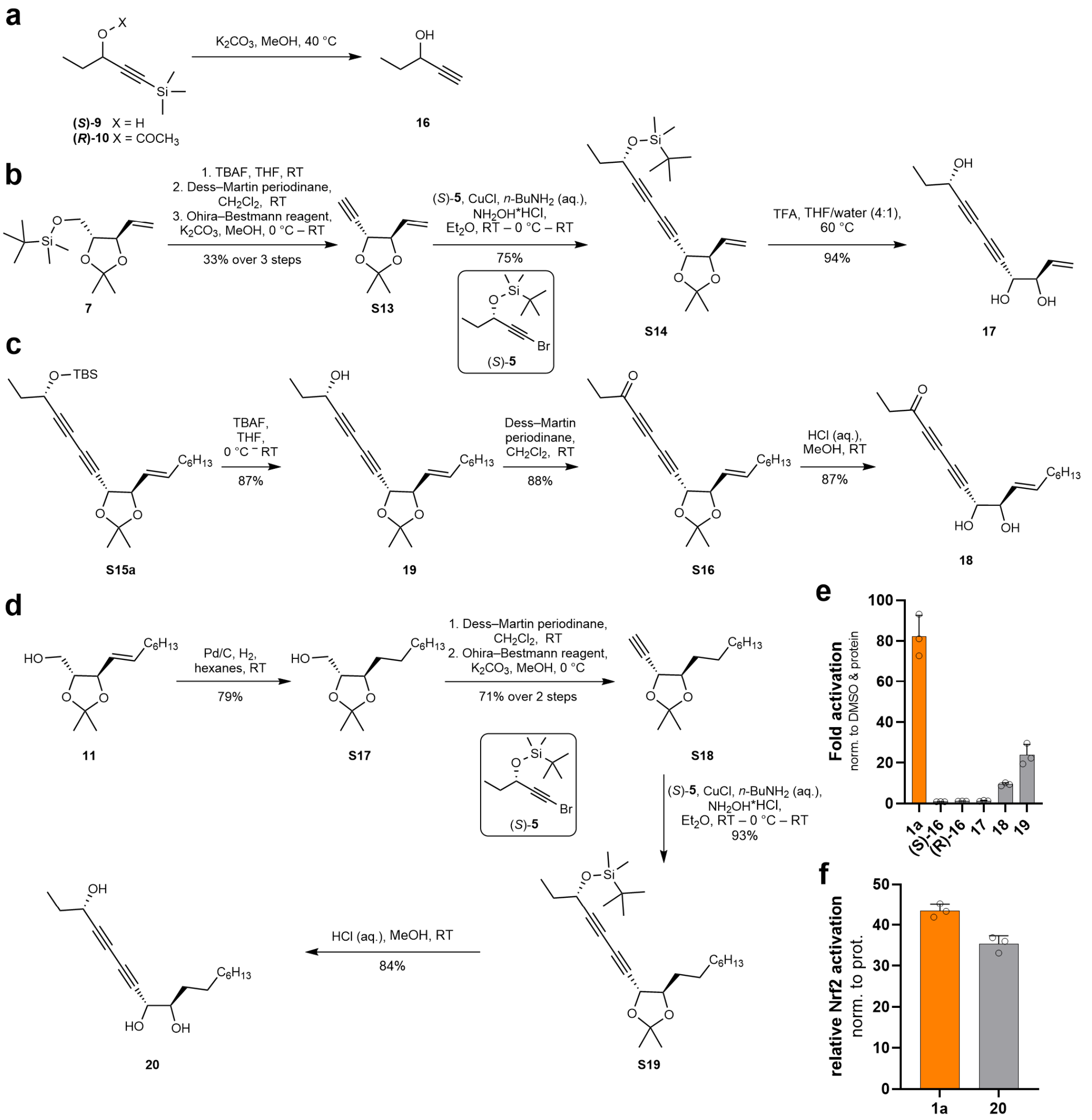

Supplementary Fig. 5. Synthesis of isofalcarintriol derivates and respective NRF2 activations. a-d) Synthesis schemes propargylic alcohol fragments (*S*)-**16**, (*R*)-**16**, terminal alkene (3*S*,8*R*,9*R*)-**17**, 3-keto derivate (8*R*,9*R*)-**18**, acetonide protected derivate (3*S*,8*R*,9*R*)-**19**, and 10,11-dihydro analogue (3*S*,8*R*,9*R*)-**20**, respectively. **e-f)** NRF2 luciferase reporter assay after overnight treatment in transgenic HEK293 cells with isofalcarintriol (**1a**) derivates. Data include three technical replicates and are represented as average + SD.

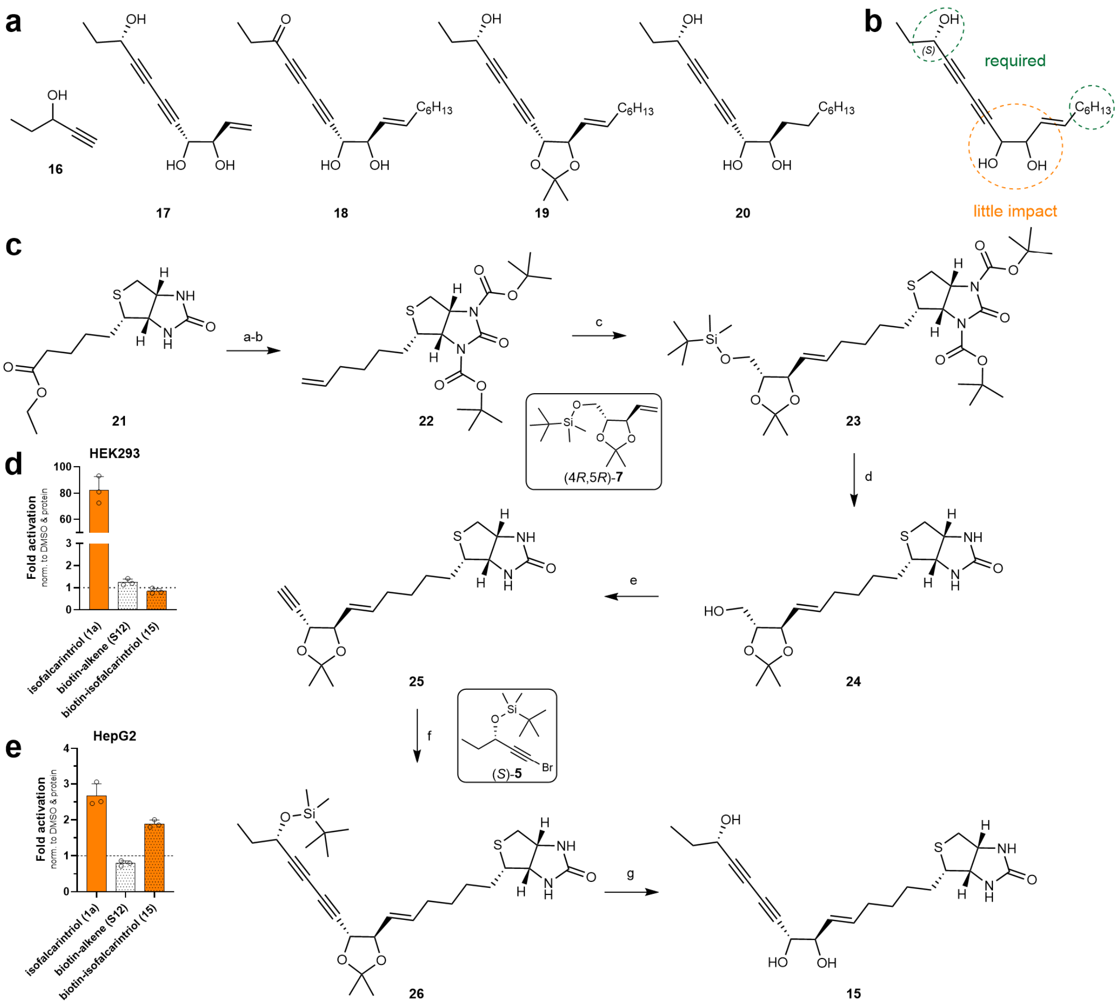

Supplementary Fig. 6. Design and Synthesis of pull-down probe biotin-isofalcarintriol and NRF2 activations in HEK293 and HepG2. a) Structure of SAR compounds (**16-20**). **b)** Depiction of the isofalcarintriol (**1a**) pharmacophore **c)** Synthesis of (3*S*,8*R*,9*R*)-biotin-isofalcarintriol (**15**). Reagents and conditions: a, 1: DIBAL-H, CH_2Cl_2, –78 °C; 2: PPh_3MeBr, KO*t*-Bu, THF, RT, 60% over two steps. b, Boc_2O, DMAP cat., TEA, CH_2Cl_2, RT, 99%. c, (4*R*,5*R*)-**7**, Hoveyda-Grubbs Catalyst 2nd Generation cat., Ti(O*t*-Bu)$_4$, CH_2Cl_2, 40 °C, 66% (30% recovered 10). d, 1: K_2CO_3, MeOH, 60 °C; 2: TBAF, THF, 0 °C to RT, 78% over two steps. e, 1: EDCI, TFA, pyridine, DMSO, RT, 2: Ohira–Bestmann reagent, K_2CO_3, MeOH, 0 °C to RT, 59% over 2 steps. f, CuCl cat., n-BuNH2 (aq.), Et2O, RT, then (S)-**5**, 0 °C to RT, 82%. g, HCl (aq.), MeOH, RT, 92%. **d-e)** NRF2 luciferase reporter assay with biotin-isofalcarintriol (**15**) after overnight treatment in transgenic HEK293 cells and HepG2 cells, respectively. isofalcarintriol (**1a**) used as positive control and biotin-alkene (**S12**) used as negative control. Data include three technical replicates and are represented as average + SD.

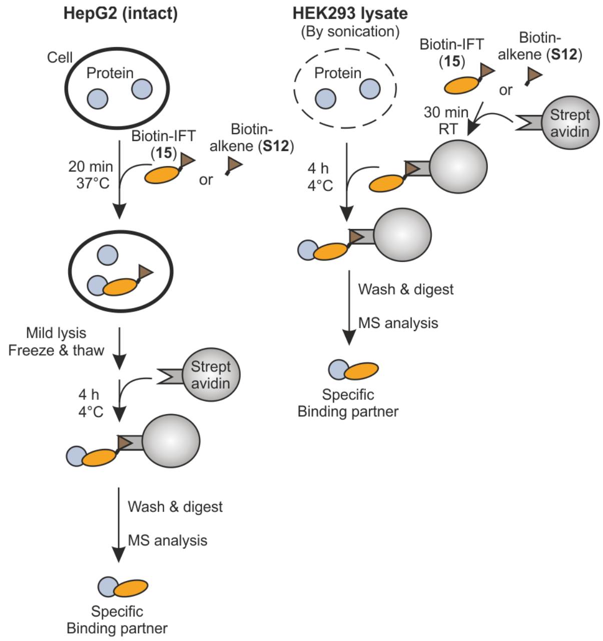

Supplementary Fig. 7. Experimental steps applied in HepG2 and HEK293 pulldown experiments

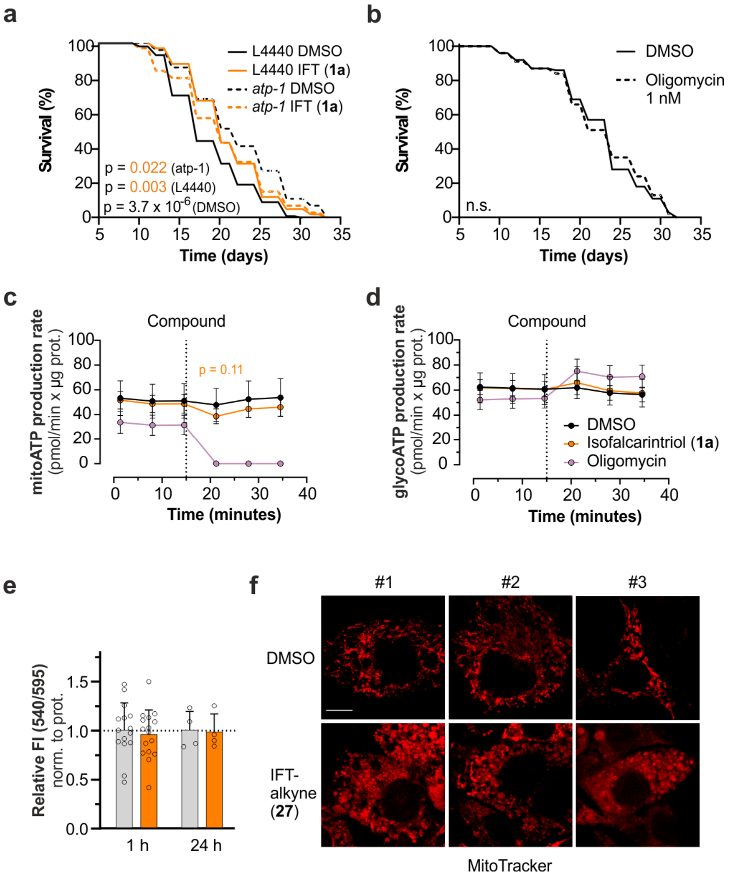

Supplementary Fig. 8. Impact of impaired ATP synthase function by RNAi and pharmacological inhibition by oligomycin and isofalcarintriol (IFT) on mitochondrial shape and function. a) Epistasis lifespan by *atp-1* RNAi on solvent-control treated compared to L4440 control nematodes with isofalcarintriol (**1a**). **b)** Lifespan of 1nM oligomycin. **c-d)** Seahorse Real-Time ATP Rate Assay (n = 8 independent cell samples per condition) indicating **c)** mitoATP production rate and **d)** glycolytic ATP production rate after injection of isofalcarintriol (**1a**). **e)** Membrane potential after isofalcarintriol (**1a**) treatment after 1 h (DMSO: n = 15; IFT: n = 16 independent cell samples) and 24 h (n = 4 independent cell samples per condition). **f)** Confocal microscopy of isofalcarintriol **(1a)**-treated mitochondria in three independent experiments. Scale bar: 10 µM. Data are represented as average or average + SD. *C. elegans* data include three biologically independent samples. Statistics: log-rank test, two-sided unpaired student's t-test, two-way ANOVA; p-values as indicated.

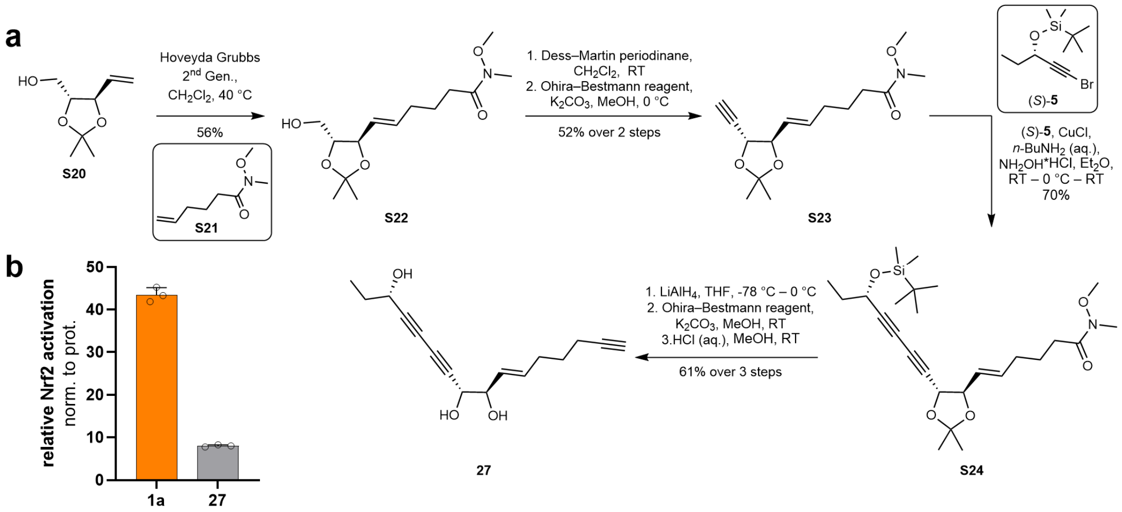

Supplementary Fig. 9. Synthesis of isofalcarintriol-derivate for click chemistry and its NRF2 activation. a) Synthesis scheme for isofalcarintriol-alkyne (3*S*,8*R*,9*R*)-**27**. **b)** NRF2 luciferase reporter assay after overnight treatment in transgenic HEK293 cells with **27**. Data include three technical replicates and are represented as average + SD.

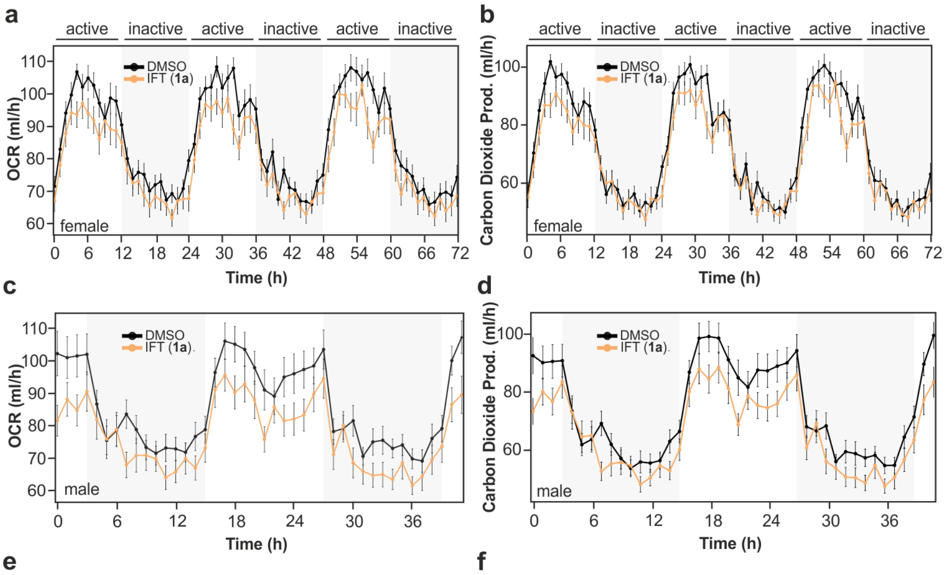

e

Males	Time period	Group effect (p value)
Oxygen Consumption	active phase	0.089
	full day	0.205
Carbon Dioxide Production	active phase	0.061
	full day	0.124

f

Females	Time period	Group effect (p value)
Oxygen Consumption	active phase	**0.031 ***
	full day	**0.046 ***
Carbon Dioxide Production	active phase	**0.042 ***
	full day	0.090

Supplementary Fig. 10. Indirect calorimetry revealing decreased oxygen consumption and carbon dioxide production upon isofalcarintriol (IFT) (1a)-treatment in female and male mice on chow diet. a) Oxygen consumption rate of female mice. **b)** Carbon dioxide production of female mice. **c)** Oxygen consumption rate of male mice. **d)** Carbon Dioxide production of ~~fe~~male mice. **e-f)** One-way ANOVA statistics for **e)** male (DMSO: n = 9; IFT: n =11) and **f)** female (DMSO: n = 10; IFT: n =11) mice. Data are represented as average ± SEM; p-values as indicated.

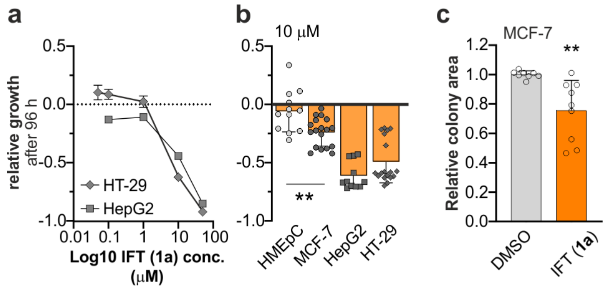

Supplementary Fig. 11. Inhibition of tumor cell growth and colony formation by isofalcarintriol (IFT). a) Inhibition of HT-29 and HepG2 cell growth with increasing concentration of isofalcarintriol (**1a**) (HT-29: n = 6; HepG2: n = 3 independent cell samples per condition). **b)** Inhibition of cell growth by 10 μM isofalcarintriol (**1a**) in several cell lines (HMEpC: n = 12; MCF-7: n = 18; HepG2: n = 11; HT-29: n = 18) including in 3 independent experiments (p = 0.0027). **c)** Quantification of the relative size of MCF-7 colonies formed during the soft agar assay (DMSO: n = 8; IFT: n = 9) in 2 independent experiments (p = 0.0073). Data are represented as average + SD or ± SD. Statistics: two-sided unpaired student's t-test.

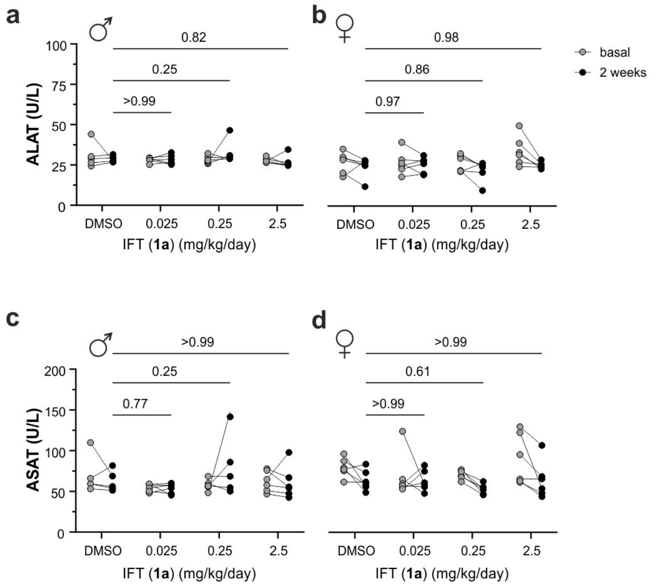

Supplementary Fig. 12. Measures of liver toxicity upon application of different doses of isofalcarintriol (IFT) (1a) to wild-type mice on chow diet. a-b) ALAT and **c-d)** ASAT blood levels before and after 2 weeks of IFT treatment in **a), c)** male (DMSO: n = 5; IFT-treated groups: n = 6) and **b), d)** female (n = 6 per group) mice. Data are represented as single values. Statistics: Two-way ANOVA; p-values as indicated.

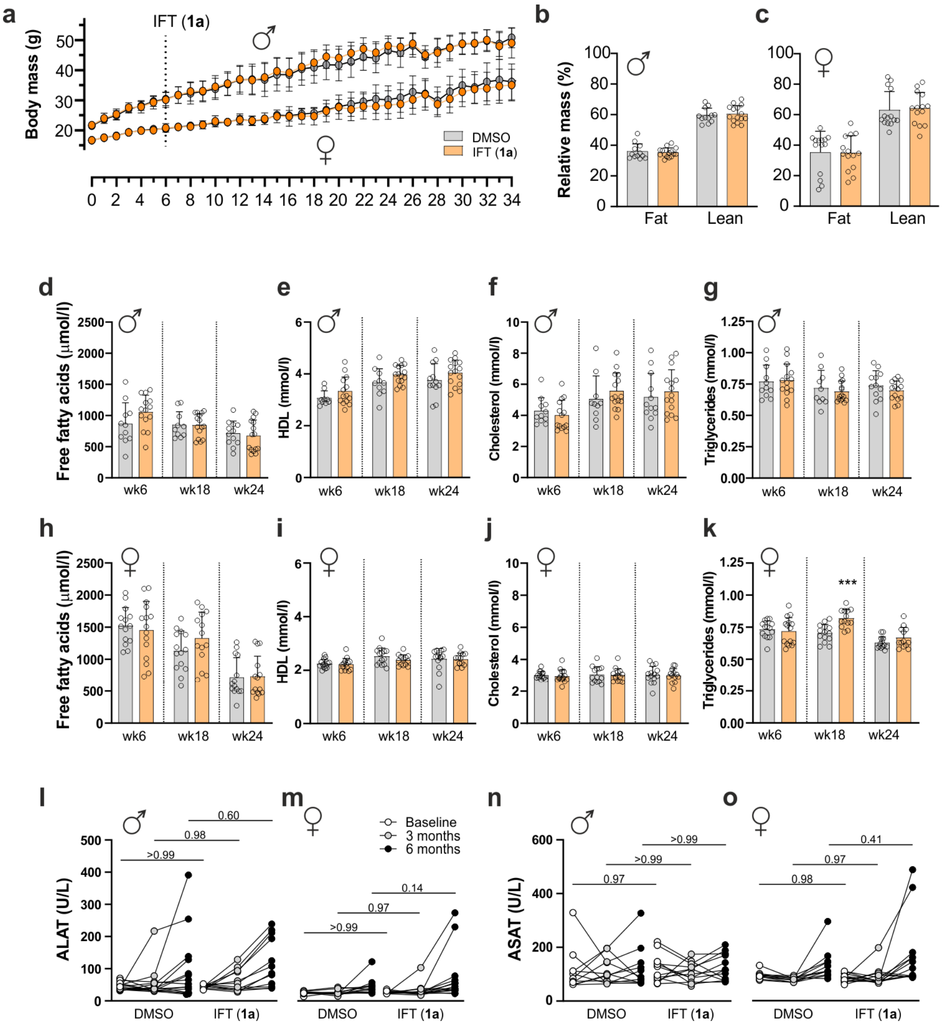

Supplementary Fig. 13. Body mass and composition as well as blood lipid levels and toxicity parameters of high-fat diet wild-type mice on isofalcarintriol (IFT) (1a)-treatment. a) Total body mass over the course of the study (males DMSO: n = 12; males IFT: n = 15; females: n = 14 mice per condition). **b-c)** Relative fat and lean mass of **b)** male and **c)** female mice in study week 24 (males DMSO: n = 12; males IFT: n = 15; females: n = 14 mice per condition). **d-g)** Blood lipid levels of e) male (DMSO: n = 11; IFT: n = 14 mice) including d) free fatty acids, e) HDL, f) cholesterol, and g) triglycerides. **h-k)** Blood lipid levels of female mice (n = 14 mice per condition) (p = 0.0002) including h) free fatty acids, i) HDL, j) cholesterol, and k) triglycerides. **l-m)** ALAT blood levels of l) male and m) female mice. **n-o)** ASAT blood levels of n) male and o) female mice across the study period (males DMSO: n = 12; males IFT: n = 15; females DMSO: n = 12; females IFT: n = 14 mice per condition). Data are represented as single values or average + SD. Statistics: Two-way repeated measures ANOVA; two-sided unpaired student's t-test; p-values as indicated.

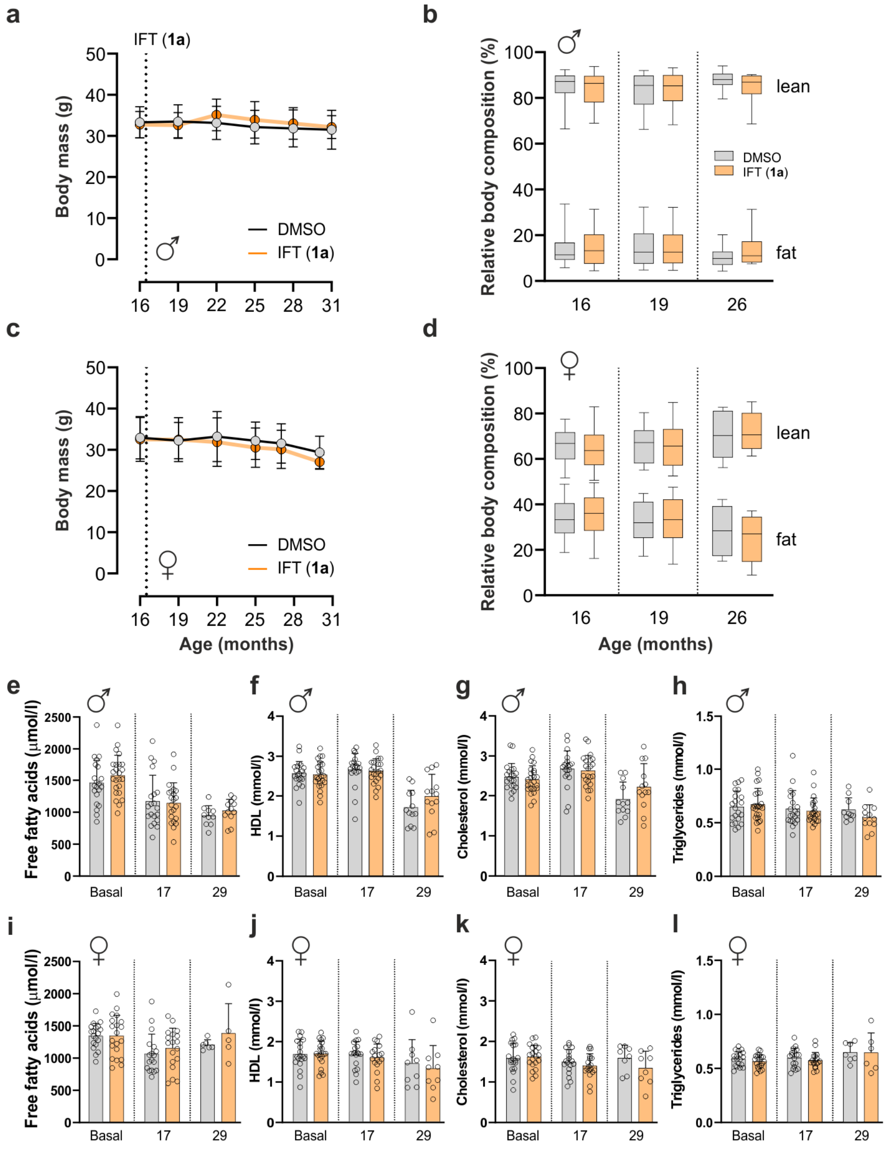

Supplementary Fig. 14. Body mass and composition as well as blood lipid levels of aged wild-type mice on isofalcarintriol (IFT) (1a)-treatment. a, c) Total body mass of a) male (DMSO: n = 44; IFT: n = 43 mice initially) and c) female mice (DMSO: n = 43; IFT: n = 40 mice initially) over the course of the study. **b, d)** Relative fat and lean mass of b) male (n = 23 mice per condition initially) and d) female mice (n = 20 mice per condition initially). **e-h)** Blood lipid levels of male (DMSO: n = 21; IFT: n = 24 mice initially) mice including e) free fatty acids, f) HDL, g) cholesterol, and h) triglycerides. **i-l**) Blood lipid levels of female mice (DMSO: n = 19; IFT: n = 20 mice initially) including i) free fatty acids, j) HDL, k) cholesterol, and l) triglycerides at baseline (15 months of age) and at 17 and 29 months of age. Data are represented as average + SD. Box plots indicate median (middle line), 25th, 75th percentile (box) and min, max values (whiskers). Statistics: Two-way repeated measures ANOVA; two-way ANOVA; two-sided unpaired Student's t-test.

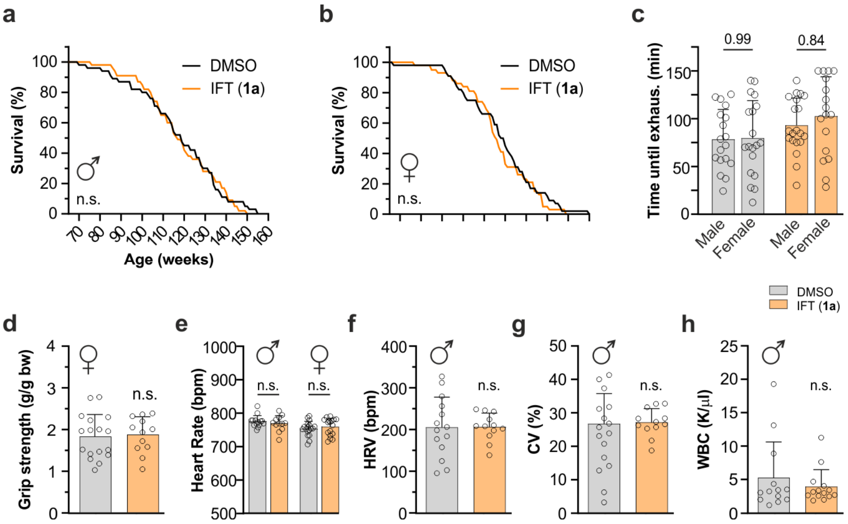

Supplementary Fig. 15. Murine health parameters that were not affected by isofalcarintriol (1a). a-b) Lifespan of a) male (DMSO: n = 47; IFT: n = 48) and b) female mice (DMSO: n = 47; IFT: n = 46). **c)** Comparison of exercise capacity between male and female mice showing no difference (males DMSO: n = 18; males IFT: n = 19; females DMSO: n = 20; females IFT: n = 17). **d)** Grip strength of female mice (DMSO: n = 17; IFT: n = 12). **e)** Heart rate of male and female mice (males DMSO: n = 14; males IFT: n = 12; females DMSO: n = 18; females IFT: n = 17). **f)** The heart rate variability (HRV) and **g)** the coefficient of variation (CV) (DMSO: n = 14; IFT: n = 12) as well as **h)** the white blood cell count (WBC) (DMSO: n = 13; IFT: n = 14) was unchanged in male mice. Data are represented as average + SD. Statistics: Log-rang test; two-way ANOVA; two-sided unpaired Student's t-test; p-values as indicated.

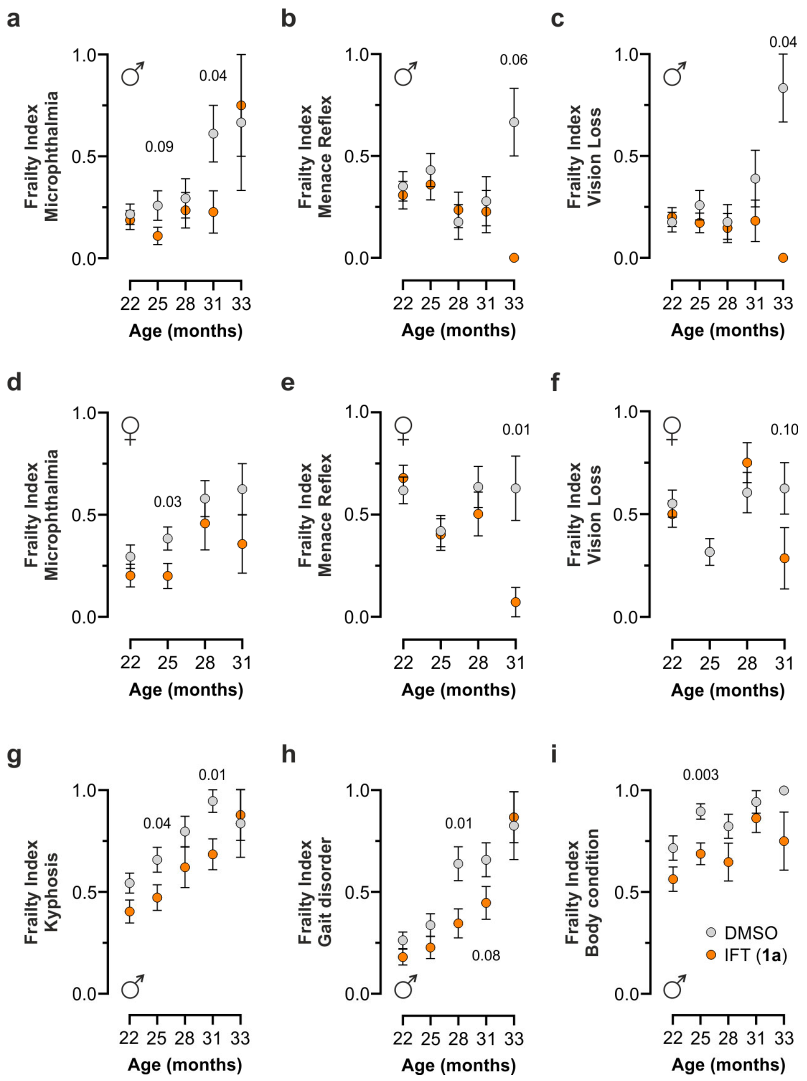

Supplementary Fig. 16. Single frailty index parameters that were improved by isofalcarintriol (1a). a-f) Ocular parameters in a-c) male and d-f) female mice. **g-i)** Physical/musuloskelatal parameters in male mice only (males DMSO: n = 27; males IFT: n = 40; females DMSO: n = 39; females IFT: n = 37). Data are represented as average ± SEM. Statistics: mixed effects analysis; p-values as indicated.

Supplementary Table 1. Overview of top ATP inhibitors and respective potential to active NRF2. Both ATP inhibition and NRF2 activation is shown as percent compared to DMSO control and normalized to protein. NA (not available) = compounds are novel and do not have an assigned name yet.

rank	name	relative ATP + std (norm. to DMSO & protein)	relative Nrf2 activation (norm. to DMSO & protein)
1	NA	0.91	0.79
2	NA	0.91	1.02
3	NA	0.92	1.61
4	10-Gingerol	0.92	6.00
5	NA	0.93	1.06
6	Alnusone	0.93	12.35
7	NA	0.93	0.93
8	NA	0.93	0.96
9	4-(3,4-Dihydroxyphenyl)-6,7-dihydroxy-2-naphthalenecarboxylic acid	0.93	0.91
10	Andrographiside	0.93	1.08
11	NA	0.94	0.90
12	NA	0.94	0.87
13	Asperulosidic acid	0.94	0.65
14	8-Hydroxy-7(11)-eremophilen-12,8-olide	0.94	0.92
15	NA	0.94	0.81
16	3-Hexen-1-ol O-b-D-glucopyranoside	0.95	0.83
17	NA	0.95	0.85
18	NA	0.95	0.77
19	NA/Isofalcarintriol	0.95	30.85
20	Acoric acid	0.95	0.67
21	NA	0.95	1.11
22	[6]-Gingerol	0.95	1.21
23	NA	0.95	0.80
24	Syringaresinol	0.95	1.20
25	NA	0.95	1.31
26	Vulgarin	0.95	1.04
27	3,8''-Biapigenin	0.95	0.80
28	Embelin	0.95	0.68
29	NA	0.95	0.92

Supplementary Table 2. Overview and two-sided log-rank statistics to performed isofalcarintriol (IFT) (**1a**) lifespan assays; green = lifespan extension; red = lifespan shortening

Strain	Name	Treatment/ RNAi	N (censored)	Mean lifespan	± SEM	Median lifespan (days)	Max lifespan (days)	p-value	vs	Mean compared to control (%)	Max compared to control (%)
N2	wild-type	DMSO	193(18)	23.29	0.33	24	26	1.6E-05	DMSO vs IFT 1 nM	107.6	107.7
N2	wild-type	IFT 1 nM	170(17)	25.06	0.39	26	28	4.0E-03	DMSO vs IFT 10 nM	104.6	107.7
N2	wild-type	IFT 10 nM	171(21)	24.36	0.40	24	28				
N2	wild-type	DMSO	91(27)	22.78	0.67	24	26	7.0E-03	DMSO vs IFT 0.1 nM	112.2	107.7
N2	wild-type	IFT 0.1 nM	96(22)	25.54	0.50	24	28	1.8E-05	DMSO vs IFT 1 nM	117.4	119.2
N2	wild-type	IFT 1 nM	90(12)	26.74	0.61	28	31				
N2	wild-type	DMSO	131(17)	20.97	0.41	21	24	1.1E-06	DMSO vs IFT 1 nM	114.2	116.7
N2	wild-type	IFT 1 nM	139(22)	23.94	0.46	24	28				
N2	wild-type	DMSO	158(12)	22.22	0.37	21	26	3.4E-06	DMSO vs IFT 1 nM (old)	109.5	107.7
N2	wild-type	IFT 1 nM (new)	146(7)	24.32	0.45	24	28	4.5E-05	DMSO vs IFT 1 nM (new)	111.7	107.7
N2	wild-type	IFT 1 nM (old)	152(15)	24.83	0.41	24	28				
N2	wild-type	DMSO	150(54)	22.29	0.57	22	27	5.5E-04	N2 DMSO vs N2 IFT	110.8	107.4
N2	wild-type	IFT 1nM	150(58)	24.70	0.60	25	29	1.7E-01	*aak-2* DMSO vs aak2 IFT	105.0	100.0
aak-2	RB754	DMSO	150(90)	20.84	0.64	21	25				
aak-2	RB754	IFT 1nM	150(100)	21.88	0.74	22	25				
N2	wild-type	DMSO	120(30)	20.22	0.51	20	24	5.0E-03	N2 DMSO vs N2 IFT	110.2	104.2
N2	wild-type	IFT 1nM	120(38)	22.29	0.56	21	25	5.3E-01	*skn-1* DMSO vs *skn-1* IFT	98.5	100.0
skn-1	EU31	DMSO	120(54)	17.43	0.61	17	20				
skn-1	EU31	IFT 1nM	120(50)	17.17	0.48	17	20				
aak-2	RB754	DMSO	90(16)	19.88	0.44	20	24	2.0E-01	*aak-2* DMSO vs *aak-2* IFT 1 nM	96.2	87.5
aak-2	RB754	IFT 1nM	66(13)	19.13	0.50	19	21				
skn-1	EU31	DMSO	102(4)	16.91	0.51	17	19	1.4E-04	*skn-1* DMSO vs *skn-1* IFT 1 nM	83.8	89.5
skn-1	EU31	IFT 1nM	115(2)	14.17	0.43	12	17				
Ctl-1 Oex	MIR257	DMSO	143(13)	23.16	0.39	24	26	4.8E-01	*Ctl-1* DMSO vs *Ctl-1* IFT 1 nM	98.2	107.7
Ctl-1 Oex	MIR257	IFT 1nM	140(13)	22.75	0.50	24	28				
Ctl-1 Oex	MIR257	DMSO	126(15)	25.78	0.46	26	31	9.0E-03	*Ctl-1* DMSO vs *Ctl-1* IFT 1 nM	94.4	90.3
Ctl-1 Oex	MIR257	IFT 1nM	127(5)	24.33	0.45	26	28				
N2	wild-type	L4440	131(12)	20.02	0.35	19	24	1.9E-13	L4440 vs *atp-1*	119.8	112.5
N2	wild-type	*atp-1*	160(18)	23.98	0.40	24	27				
N2	wild-type	L4440	131(12)	20.02	0.35	19	24	1.2E-02	L4440 vs *atp-3*	106.3	100.0
N2	wild-type	*atp-3*	159(9)	21.28	0.31	21	24				
N2	wild-type	*atp-1* ; DMSO	128(10)	22.20	0.53	22	28	3.0E-03	L4440 DMSO vs L4440 IFT	111.7	113.6
N2	wild-type	*atp-1* ; IFT 1nM	133(11)	20.39	0.52	20	25	3.7E-06	L4440 DMSO vs atp-1 DMSO	117.9	127.3
N2	wild-type	L4440; DMSO	103(8)	18.83	0.49	17	22	8.0E-01	L4440 IFT vs *atp-1* IFT	96.9	100.0
N2	wild-type	L4440; IFT 1nM	116(14)	21.03	0.46	20	25	2.2E-02	*atp-1* DMSO vs *atp-1* IFT	91.8	89.3

Supplementary Table 3. Comparison of ^{1}H and ^{13}C-NMR spectroscopic data of isofalcarintriol (**1**), (3*S*,8*R*,9*R*)-isofalcarintriol (**1a**) and (3*S*,8*S*,9*S*)-isofalcarintriol (**1b**).

**Atom**	isolated isofalcarintriol **(1)**[a]		(3*S*,8*R*,9*R*)-isofalcarintriol (**1a**)		(3*S*,8*S*,9*S*)-isofalcarintriol (**1b**)	
	^{1}H NMR 500 MHz, $CDCl_3$	^{13}C NMR 126 MHz, $CDCl_3$	^{1}H NMR 500 MHz, $CDCl_3$	^{13}C NMR 126 MHz, $CDCl_3$	^{1}H NMR 500 MHz, $CDCl_3$	^{13}C NMR 126 MHz, $CDCl_3$
1	1.01 (t, *J* = 7.4 Hz, 3H)	9.5 CH_3	1.01 (t, *J* = 7.4 Hz, 3H)	9.5 CH_3	1.01 (t, *J* = 7.5 Hz, 3H)	9.5 CH_3
2	1.75 (qdd, *J* = 7.3, 6.4, 2.7 Hz, 2H)	30.8 CH_2	1.79 – 1.71 (m, 2H)	30.7 CH_2	1.74 (qdd, *J* = 7.3, 6.4, 3.0 Hz, 2H)	30.7 CH_2
3	4.38 (td, J = 6.4, 0.7 Hz, 1H)	64.2 CH	4.37 (t, *J* = 6.5 Hz, 1H)	64.1 CH	4.37 (t, *J* = 6.5 Hz, 1H)	64.1 CH
4				80.7 C		80.7 C
5		80.6 C[b], 77.4 C[b],		69.0 C		69.0 C
6		70.6 C[b], 69.0 C[b]		70.6 C		70.6 C
7				77.5 C		77.5 C
8	4.27 (dd, *J* = 6.5, 0.8 Hz, 1H)	66.8 CH	4.26 (d, *J* = 6.5 Hz, 1H)	66.7 CH	4.26 (dd, *J* = 6.5, 0.8 Hz, 1H)	66.7 CH
9	4.13 (t, J = 7.1 Hz, 2H)*	75.7 CH	4.12 (t, *J* = 6.7 Hz, 1H)	75.6 CH	4.12 (t, *J* = 6.7 Hz, 1H)	75.6 CH
10	5.51 (ddt, *J* = 15.4, 6.9, 1.4 Hz, 1H)	126.7 CH	5.50 (ddt, *J* = 15.4, 6.9, 1.5 Hz, 1H),	126.6 CH	5.50 (ddt, *J* = 15.4, 6.9, 1.5 Hz, 1H),	126.6 CH
11	5.86 (dtd, *J* = 15.0, 6.9, 1.1 Hz, 1H)	136.7 CH	5.86 (dtd, *J* = 15.4, 6.9, 1.1 Hz, 1H)	136.7 CH	5.86 (dtd, *J* = 15.5, 6.8, 1.1 Hz, 1H)	136.6 CH
12	2.08 (q, *J* = 7.2 Hz, 2H)	32.5 CH_2	2.12 – 2.03 (m, 2H)	32.5 CH_2	2.12 – 2.03 (m, 2H)	32.5 CH_2
13	1.41 – 1.34 (m, 2H)	29.0 CH_2	1.46 – 1.35 (m, 2H)	29.0 CH_2	1.46 – 1.35 (m, 2H)	29.0 CH_2
14	1.34 – 1.21 (m, 12H)*	31.8 CH_2[b],	1.34 – 1.21 (m, 6H)	29.1 CH_2	1.34 – 1.21 (m, 6H)	29.1 CH_2
15	1.34 – 1.21 (m, 12H)*	29.1 CH_2[b],	1.34 – 1.21 (m, 6H)	31.9 CH_2	1.34 – 1.21 (m, 6H)	31.8 CH_2
16	1.34 – 1.21 (m, 12H)*	22.7 CH_2[b]	1.34 – 1.21 (m, 6H)	22.8 CH_2	1.34 – 1.21 (m, 6H)	22.8 CH_2
17	0.91 – 0.86 (m, 4H)*	14.3 CH_3	0.91 – 0.86 (m, 2H)	14.3 CH_3	0.91 – 0.86 (m, 2H)	14.3 CH_3

OH resonances were not observed by ^{1}H NMR spectroscopy due to prior H–D exchanged with CD_3OD. [a] Contains significant impurities, [b] not assigned, * Overlapping impurities in the spectra.

Supplementary Table 4. Quantification of isofalcarintriol in extracts from commercial *D. carota*.

Entry	Solvent, parts of carrots used	Mass [g]		Extract	Concentration of 1a	
		Total	Extracted residue	[%]	in extracts [mg/mg][a]	in carrots [µg/g][b]
1	pentane, whole	348	0.0401	0.0127	-	-
2	ethanol, whole	364	19.1	5.23	~$1.7*10^{-5}$	~8.9
3	ethyl acetate, whole	319	0.253	0.0792	~$4.8*10^{-4}$	~3.8
4	ethyl acetate, core	168	0.0508	0.0508	~$9.5*10^{-5}$	~3.5 [c]
5	ethyl acetate, flesh	560	0.377	0.0673	~$9.2*10^{-5}$	
6	ethyl acetate, peel	251	0.213	0.0792	~$1.2*10^{-3}$	

[a] determined by LC-MS using standard addition method, [b] dry weight basis (assuming 90% water content), [c] reconstituted from entries 4-6.
- = not detected

Supplementary Table 5. Overview of common protein hits based on biotin-isofalcarintriol pulldown in HepG2 and HEK23. The cut off was set to ≥ 1.5-fold intensity over negative control (Biotin-alkene) with at least 2 peptides found per identified protein.

Protein name	Prot ID	Intensity over neg ctrl. HEK293	Intensity over neg ctrl. HepG2	Average value
60S ribosomal protein L13a	Q8J015	not detected in neg. ctrl.	1.57	1.57
AP-3 complex subunit delta-1	A0A2R8Y611	2.46	1.86	2.16
ATP synthase subunit alpha, mitochondrial	P25705	2.10	1.87	1.99
ATP synthase subunit O, mitochondrial	P48047	not detected in neg. ctrl.	2.13	2.13
BAG family molecular chaperone regulator 2	O95816	3.31	2.63	2.97
Dedicator of cytokinesis protein 7	Q96N67	4.07	3.81	3.94
Desmoplakin	P15924	2.14	3.31	2.73
Gem-associated protein 5	Q8TEQ6	1.87	5.46	3.67
Heterogeneous nuclear ribonucleoprotein H3	P31942	2.44	2.19	2.31
Histone H2A.V	Q71UI9	5.57	4.16	4.87
Keratin, type I cytoskeletal 18	P05783	1.52	1.53	1.53
Large proline-rich protein BAG6	A0A024RCR6	not detected in neg. ctrl.	2.56	2.56
Pericentriolar material 1 protein	Q15154	9.53	7.49	8.51
Plectin	Q15149	1.91	2.19	2.05
Protein dpy-30 homolog	Q9C005	not detected in neg. ctrl.	1.72	1.72
Protein LSM14 homolog A	Q8ND56	2.07	2.90	2.48
Protein phosphatase 1 regulatory subunit 12A	O14974	2.28	5.79	4.03
Ras-related protein Rab-1A	P62820	1.59	1.58	1.58
Ribosomal RNA processing protein 1 homolog B	Q14684	3.26	8.09	5.68
SAFB-like transcription modulator	Q9NWH9	1.53	18.25	9.89
Septin-9	Q9UHD8	4.20	1.84	3.02
Serine hydroxymethyltransferase, mitochondrial	P34897	not detected in neg. ctrl.	2.18	2.18
Serine/arginine-rich splicing factor 3	P84103	1.61	4.99	3.30
Sorting nexin-2	O60749	7.46	2.20	4.83
Spectrin beta chain, non-erythrocytic 1	Q01082	1.59	1.64	1.61
Syntaxin-5	Q13190	not detected in neg. ctrl.	1.87	1.87
Transcriptional repressor p66-beta	A0A0U1RRM1	1.81	1.84	1.83
U1 small nuclear ribonucleoprotein 70 kDa	P08621	1.68	9.19	5.43
X-ray repair cross-complementing protein 5	P13010	3.93	1.67	2.80
Zinc finger CCCH domain-containing protein 15	Q8WU90	2.05	2.37	2.21

Supplementary Table 6. Gene annotation enrichment analysis showing overrepresented functional pathways of biotin-pulldown protein hits. Analysis was done via DAVID Bioinformatics Resources applying a one-sided EASE Score (Modified Fisher Exact P-value) [22,23]

REACTOME PATHWAY - Functional Annotation			Selection List		Reference List			
**Term**	**Category**	**Genes**	**Count**	**Total**	**Count**	**Total**	**Fold change**	**P value**
R-HSA-163210	Formation of ATP by chemiosmotic coupling	P25705, P48047	2	23	18	10957	53	0.036
R-HSA-9609523	Insertion of tail-anchored proteins into the endoplasmatic reticulum membrane	A0A024RCR6, Q13190	2	23	22	10957	43	0.043
R-HSA-6807878	COPI-mediated anterograde transport	Q13190, Q01082, P62820	3	23	101	10957	14	0.017
R-HSA-199977	ER to Golgi anterograde transport	Q13190, Q01082, P62820	3	23	154	10957	9	0.038
R-HSA-9609507	Protein localization	A0A024RCR6, **P25705**, Q13190	3	23	164	10957	9	0.042
R-HSA-9012999	RHO GTPase cycle	Q96N67, Q13190, P15924, Q01082, P34897	5	23	449	10957	5	0.011
R-HSA-194315	Signaling by Rho GTPases	Q96N67, Q13190, P15924, Q01082, O14974, Q71UI9, P34897	7	23	706	10957	5	0.002
R-HSA-9716542	Signaling by Rho GTPases, Miro GTPases and RHOBTB3	Q96N67, Q13190, P15924, Q01082, O14974, Q71UI9, P34897	7	23	722	10957	5	0.002

Supplementary Table 7. Overview and two-sided log-rank statistics to performed compound lifespans other than isofalcarintriol (IFT) (**1a**); green = lifespan extension; red = lifespan shortening

Strain	Treatment	N (censored)	Mean lifespan	± SEM	Median lifespan (days)	Max lifespan (days)	p-value	vs	Mean compared to control (%)	Max compared to control (%)
N2	DMSO	131 (18)	21.96	0.48	24	26	0.050	DMSO vs Alnusone 1 nM	105.8	100.0
N2	Alnusone 1 nM	118(10)	23.23	0.51	24	26	0.010	DMSO vs Alnusone 10 nM	107.1	100.0
N2	Alnusone 10 nM	132(17)	23.52	0.52	24	26	0.818	DMSO vs Alnusone 100 nM	100.9	100.0
N2	Alnusone 100 nM	107(12)	22.15	0.52	24	26				
N2	DMSO	131 (18)	21.96	0.48	24	26	0.784	DMSO vs 10-Gingerol 1 nM	99.4	100.0
N2	10-Gingerol 1 nM	125(15)	21.82	0.54	24	26	0.548	DMSO vs 10-Gingerol 10 nM	98.3	92.3
N2	10-Gingerol 10 nM	115(11)	21.58	0.50	21	24	0.003	DMSO vs 10-Gingerol 100 nM	92.7	92.3
N2	10-Gingerol 100 nM	126(9)	20.36	0.41	21	24				
N2	DMSO	193(18)	23.29	0.33	24	26	0.524	DMSO vs Alnusone 1 nM	100.5	107.7
N2	Alnusone 1 nM	173(16)	23.39	0.37	24	28	0.123	DMSO vs Alnusone 10 nM	102.0	107.7
N2	Alnusone 10 nM	190(17)	23.75	0.36	24	28				
N2	DMSO	107(7)	21.96	0.46	21	26	0.17	DMSO vs Bz-423 1 nM	103.3	100.0
N2	Bz-423 1 nM	112(27)	22.68	0.50	24	26	0.002	DMSO vs Bz-423 10 nM	107.6	107.7
N2	Bz-423 10 nM	114(24)	23.63	0.48	26	28	0.737	DMSO vs Bz-423 100 nM	100.1	100.0
N2	Bz-423 100 nM	122(20)	21.98	0.45	21	26				
N2	DMSO	101(10)	22.71	0.57	24	27	0.887	DMSO vs Oligomycin 1 nM	100.4	100.0
N2	Oligomycin 1 nM	124(13)	22.79	0.55	24	27	0.982	DMSO vs Oligomycin 10 nM	99.1	100.0
N2	Oligomycin 10 nM	134(15)	22.50	0.55	24	27	0.006	DMSO vs Oligomycin 100 nM	93.9	88.9
N2	Oligomycin 100 nM	135(12)	21.33	0.42	21	24				
N2	DMSO	158(21)	22.15	0.37	21	26	0.578	DMSO vs Piceatannol 1 nM	100.7	100.0
N2	Piceatannol 1 nM	155(7)	22.31	0.39	24	26	6E-05	DMSO vs Piceatannol 10 nM	109.5	107.7
N2	Piceatannol 10 nM	143(18)	24.26	0.46	24	28	0.004	DMSO vs Piceatannol 100 nM	106.1	100.0
N2	Piceatannol 100 nM	158(9)	23.51	0.41	24	26				

Supplementary Table 8. Overview and two-sided log-rank statistics to performed paraquat stress assay and Alzheimer's disease paralysis assay. Green = prolonged survival/time until paralysis

Strain	Treatment	N (censored)	Mean (h)	± SEM	Median (h)	Max (h)	p-value	Mean compared to control (%)	Max compared to control (%)
N2	Paraquat; DMSO	200(58)	68.68	2.08	62	86	7.26E-06	122.9	127.9
N2	Paraquat; IFT	197(49)	84.37	2.78	72	110			
N2	Paraquat; DMSO	137(39)	76.17	2.46	74	90	0.047	109.4	126.7
N2	Paraquat; IFT	144(53)	83.30	2.67	74	114			
GMC101	DMSO	194(0)	92.16	3.75	82	120	1.74E-09	144.6	180.0
GMC101	IFT 1 nM	172(0)	133.26	5.87	106	216			

Supplementary Table 9. Overview and statistics (two-tailed Mann-Whitney test) of pro-inflammatory cytokine plasma levels of DMSO and IFT-treated female mice (month 29).

	Average (pg/ml)		Standard Deviation		Mann-Whitney test
	**DMSO**	**IFT**	**DMSO**	**IFT**	**p-value**
**IFN-γ**	1.25	1.93	1.83	2.47	0.42
**IL-1β**	0.20	0.17	0.17	0.15	0.74
**IL-2**	1.79	2.73	0.84	1.60	0.17
**IL-5**	2.14	5.78	2.49	5.86	0.22
**IL-6**	39.24	70.95	29.99	57.85	0.28
**IL-12p7**	3.67	4.71	8.08	6.75	0.88
**KC**	83.31	92.60	33.86	45.87	0.96
**TNF-α**	20.83	52.51	11.49	34.60	0.07

Supplementary Table 10. List of used sgRNAs and PCR primers; * obtained from Refs. [24,25]

Type	Name	Sequence (5’-3’)
sgRNA	Nrf2 position 1725	ACT AAA CAC AAG TCC CAG TG
sgRNA	Nrf2 position 2031	TTG TGA GAT GAG CCT CCA AG
PCR primer	Nrf2 1205-1725 for	CCA AAA CCA CCC TGA AAG CA
PCR primer	Nrf2 1205-1725 del rev	TGA AGT CAA CAA CAG GGA GGT
PCR primer	Nrf2 1205-1725 WT rev	CTG CCC CTG AGA TGG TGA C
qPCR primer	Human nDNA (b2M) for*	TGC TGT CTC CAT GTT TGA TGT ATC T
qPCR primer	Human nDNA (b2M) rev*	TCT CTG CTC CCC ACC TCT AAG T
qPCR primer	Human mtDNA (16S rRNA) for*	GCC TTC CCC CGT AAA TGA TA
qPCR primer	Human mtDNA (16S rRNA) rev*	TTA TGC GAT TAC CGG GCT CT
qPCR primer	*C. elegans* nDNA (act-3) for*	TGC GAC ATT GAT ATC CGT AAG G
qPCR primer	*C. elegans* nDNA (act-3) rev*	GGT GGT TCT CCG GAA AGA A
qPCR primer	*C. elegans* mtDNA (nd-1) for*	AGC GTC ATT TAT TGG GAA GAA GAC
qPCR primer	*C. elegans* mtDNA (nd-1) rev*	AAG CTT GTG CTA ATC CCA TAA ATG T
qPCR primer	Mouse mtDNA for	AAG ACA CCT TGC CTA GCC ACA C
qPCR primer	Mouse mtDNA rev	TGG CTG GCA CGA AAT TTA CC
qPCR primer	Mouse nDNA (18rRNA) for	AAC TTT CGA TGG TAG TCG CCG
qPCR primer	Mouse nDNA (18rRNA) rev	CCT TGG ATG TGG TAG CCG TTT

Chemical Synthesis Procedure

1 Chemicals and Equipment

Chemicals were purchased from ABCR, Alfa Aesar, ACROS, Sigma Aldrich, TCI, Strem, Combi-Blocks, or Fluorochem, and used without further purification unless otherwise stated. Anhydrous solvents were purchased over molecular sieves or obtained using an LC Technology Solutions SP-1 solvent purification system. Deuterated solvents were purchased from Armar Chemicals or Cambridge Isotope Laboratories. Triethylamine was distilled from CaH_2 under an atmosphere of dry nitrogen and pyridine from KOH, respectively. All non-aqueous reactions were performed in vacuum dried glassware under a positive pressure of dry nitrogen. Thin layer chromatography was performed on MERCK silica gel F254 TLC glass plates and visualized with UV fluorescence quenching, $KMnO_4$ stain, Vanillin stain, or CAM stain. Chromatographic purification was performed as flash column chromatography with 0.3–0.5 bar pressure using Sigma-Aldrich or SILICYCLE SiliaFlash® Silica Gel P60. Nuclear Magnetic Resonance spectra were recorded on BRUKER ASCEND, BRUKER AVIII, BRUKER DRX or BRUKER NEO (400 MHz / 500 MHz /600 MHz for ^{1}H NMR, 101 MHz / 126 MHz / 151 MHz for ^{13}C NMR and 92 MHz for ^{2}H NMR) spectrometers. Chemical shifts (δ) are reported in ppm using the residual solvent signal as internal standard (chloroform at 7.26 and 77.16 ppm, methanol at 3.31 and 49.00 ppm). The spectroscopic data is reported as (s = singlet, d = doublet, t = triplet, m = multiplet or unresolved, coupling constant(s), integration). ^{13}C NMR spectra were recorded with complete ^{1}H decoupling. Infrared spectra were recorded on a PERKIN ELMER TWO-FT-IR spectrometer as thin films. Absorptions are given in wavenumbers (cm–1). Mass spectrometric analyses were performed as high resolution ESI and EI measurements by the mass spectrometry service of the Laboratorium für Organische Chemie at ETH Zürich. Optical rotations were measured on a Jasco P-2000 Polarimeter, 10 cm, 1.5 mL cell. Supercritical fluid chromatography (SFC) was performed on a Jasco 2080 Plus system with a diode array detector. Quantitative LS-MS samples were separated on an Agilent LC system (1290 series, Bruker Compass Hystar 5.0 service pack 1) using an Agilent Eclipse Plus C18, 3.0x1500, 3.5μm column at room temperature connected to a Bruker maXisII - ESI-Qq-TOF-MS. Chromatograms were processed and analyzed using Bruker Compass DataAnalysis 5.3. The measured area counts were corelated by linear regression using GraphPad Prism 9.3.0.

2 *General Procedures*

General Procedure A:

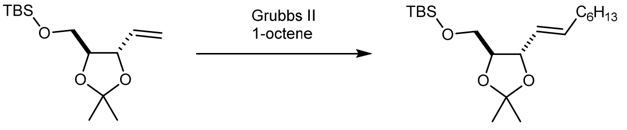

7 **S25**

To a degassed solution of **7** (1.0 equiv) in CH_2CH_2 (0.15 M) were added freshly distilled octene (3.0–15 equiv) and GRUBBS 2nd generation catalyst (4.0–7.0 mol%). The reaction mixture was sparged with argon and the reaction mixture was stirred at 40 °C. After 40–140 h the reaction mixture was directly loaded on a column and purified by flash column chromatography using silica impregnated with silver nitrate (pentane–diethyl ether, 20:1).[26]

General Procedure B:

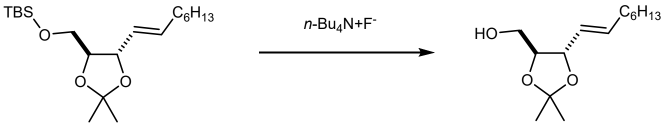

S25 **11**

To a solution of **S25** (1.0 equiv) in THF (0.15 M) at 0 °C *n*-$Bu_4N^+F^-$ (2.0 equiv) was slowly added. The reaction was allowed to reach room temperature and after complete consumption of the starting material the reaction was stopped by addition of sat. aq. NH_4Cl solution. The crude reaction mixture was extracted with ethyl acetate. The combined organic layers were dried over Na_2SO_4, filtered and concentrated under reduced pressure. The crude mixture was purified by flash column chromatography (hexanes–ethyl acetate, 4:1).

General Procedure C:

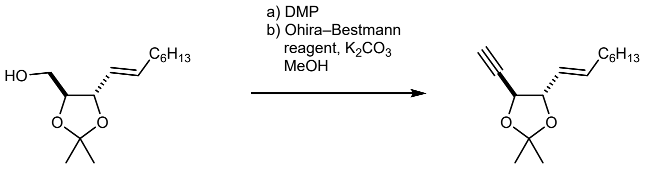

11 **4a**

To a solution of **11** (1.0 equiv) in CH_2Cl_2 (0.10 M) was added DESS–MARTIN periodinane (1.2 equiv) at room temperature. After complete consumption of the starting material, the reaction was stopped by addition of sat. aq. $Na_2S_2O_3$ solution and sat. aq. $NaHCO_3$ solution. The suspension was stirred vigorously for 10 min and extracted with diethyl ether. The combined organic layers were washed with water, dried over $MgSO_4$, filtered and concentrated under reduced pressure.

OHIRA–BESTMANN reagent (3.0 equiv) and K_2CO_3 (4.1 equiv) were suspended in MeOH at 0 °C. After 60 min the crude product dissolved in MeOH was slowly added resulting in a 50 mM solution. After complete consumption of the starting material the reaction was stopped by addition of brine and the crude reaction mixture was extracted with pentane. The combined organic layers were dried over $MgSO_4$, filtered and concentrated under reduced pressure. The crude mixture was purified by flash column chromatography (pentane–diethyl ether, 25:1).

General Procedure D:

Procedure adapted from: [27]

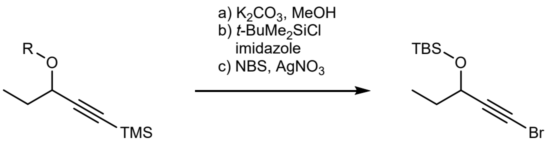

(*S*)-**9** (R = H)
(*R*)-**10** (R = Ac)

5

To a solution of (*S*)-**9** and (*R*)-**10** (1.0 equiv) in MeOH (0.10–0.60 M) K_2CO_3 (1.5–2.0 equiv) was added and the reaction was stirred at 40 °C. After complete consumption of the starting material the reaction was stopped by addition of sat. aq. NH_4Cl solution. The crude reaction mixture was extracted with diethyl ether. The combined organic layers were washed with water, dried over $MgSO_4$, filtered and concentrated under reduced pressure.
A small sample of propargylic alcohol was esterified using 3,5-dinitrobenzoyl chloride and Et_3N and the resulting esters **S26** was then used to determine the enantiomeric excess by SFC. Procedure adapted from:[28]
The crude product was dissolved in CH_2Cl_2 (0.10–0.40 M), imidazole (2.2 equiv) and *t*-$BuMe_3SiCl$ (1.1 equiv) were added at 0 °C. The reaction was allowed to reach room temperature and after complete consumption of the starting material the reaction was stopped by addition of water. The crude reaction mixture was extracted with CH_2Cl_2. The combined organic layers were washed with water, dried over $MgSO_4$, filtered and concentrated under reduced pressure. The crude product was dissolved in hexanes, filtered through a short silica plug and concentrated under reduced pressure. (*R*)-((1-bromopent-1-yn-3-yl)oxy)(tert-butyl)dimethylsilane has previously been synthesized, see: [29]
The crude product was dissolved in acetone (0.44 M) under light exclusion. *N*-bromosuccinimide (1.5 equiv) and $AgNO_3$ (0.20 equiv) were added. After complete consumption of the starting material the reaction mixture was filtered through a short silica plug and concentrated under reduced pressure. The crude mixture was purified by flash column chromatography (pentane–diethylether, 150:1).

General Procedure E:

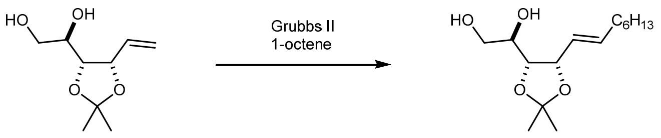

S6

S7

To a degassed solution of **S6** (1.0 equiv) in CH_2CH_2 (0.10 M) were added freshly distilled octene (5.0–7.0 equiv) and GRUBBS 2nd generation catalyst (7–10 mol%). The reaction mixture was sparged with argon and the reaction mixture was stirred at 40 °C. After 22–160 h the reaction mixture was directly loaded on a column and purified by flash column chromatography (hexanes–ethyl acetate, 1:1).

General Procedure F:

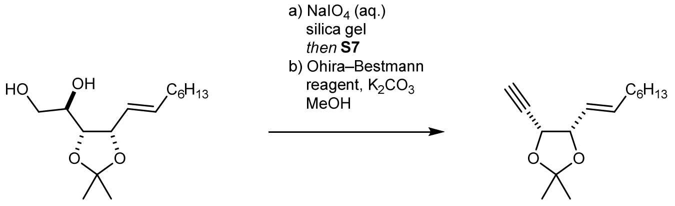

S7 **4b**

To a vigorously stirred suspension of silica gel (3.0 g/mmol) in CH_2Cl_2 (50 mM) was slowly added $NaIO_4$ (0.65 M in water, 2.0 equiv). After 5 min a solution of **S7** (1.0 equiv) in MeOH was slowly added. After complete consumption of the starting material, the reaction was filtered through a $MgSO_4$ plug and concentrated under reduced pressure.

OHIRA–BESTMANN reagent (3.0 equiv.) and K_2CO_3 (4.1 equiv.) were suspended in MeOH at 0 °C. After 60 min the crude product dissolved in MeOH was slowly added resulting in a 50 mM solution. After complete consumption of the starting material, the reaction was stopped by addition of brine and the crude reaction mixture was extracted with pentane. The combined organic layers were dried over $MgSO_4$, filtered, and concentrated under reduced pressure. The crude mixture was purified by flash column chromatography (hexanes–ethyl acetate 20:1).

General Procedure G:

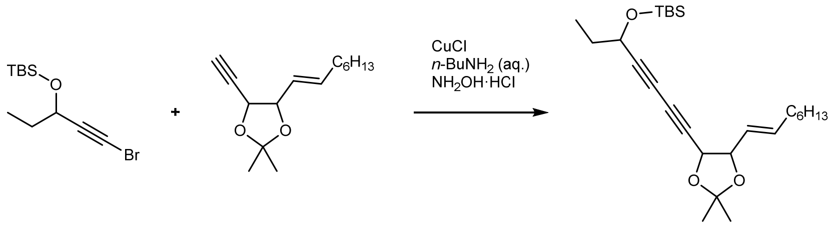

5 **4a-b** **S15a-d**

To a solution of **4** (1.0 equiv) in diethyl ether (70 mM) at room temperature was added a solution of copper(I) chloride (6.0–7.0 mol%) in *n*-$BuNH_2$ (30% in water, 25–35 equiv) resulting in a faint blue solution. After addition the reaction was cooled to 0 °C. A few crystals of hydroxylamine hydrochloride were added to discharge the blue color (indication of other than copper(I) species). A solution of **5** (1.2–1.7 equiv) in diethyl ether (70 mM) was added and the reaction was allowed to reach room temperature. After 1–7 h the reaction was stopped, diluted with water, and extracted with diethyl ether. The combined organic layers were dried over $MgSO_4$, filtered, and concentrated under reduced pressure. The crude mixture was purified by flash column chromatography (pentane–diethyl ether, 40:1).

General Procedure H:

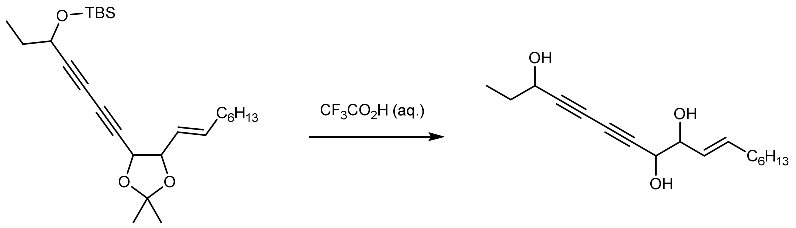

S15a-d **1a-d**

To a solution of **S15** (1.0 equiv) in THF–water (4:1, 20 mM) at room temperature was added CF_3CO_2H (30 equiv). The reaction was caped and stirred at 60 °C. After complete consumption of the starting material, the reaction was stopped, diluted with sat. aq. NH4Cl solution. The crude reaction was extracted with ethyl acetate, the combined organic layers were dried over Na_2SO_4, filtered and concentrated under reduced pressure. The crude mixture was purified by flash column chromatography (hexanes–ethyl acetate 2:1).

General Procedure I:

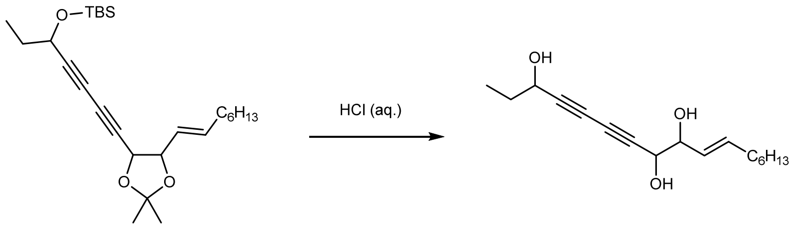

S15a-d **1a-d**

To a solution of **S15** (1.0 equiv) in MeOH (10 mM) at room temperature was added HCl (2.0 M in water, 50 equiv). After complete consumption of the starting material, the reaction was stopped, diluted with brine and water. The crude reaction was extracted with ethyl acetate, the combined organic layers were dried over Na_2SO_4, filtered, and concentrated under reduced pressure. The crude mixture was purified by flash column chromatography (hexanes–ethyl acetate 1:1).

3 *Asymmetric Synthesis of Isofalcarintriol*

3.1 Synthesis of anti-Fragment

(4*R*,5*R*)-**7** and (4*S*,5*S*)-*ent*-**7** were prepared in 5 steps from dimethyl-tartrate similarly to BROOK *et al.* [5]

Synthesis of S25

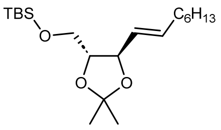

S25

The corresponding compound was prepared from **7** (8.2 g, 30 mmol, 1.0 equiv) following **general procedure A** using octene (3.0 equiv) and GRUBBS 2nd generation catalyst (4.0 mol%). The reaction was stirred for 40 h resulting in title compound as a light-brown oil (7.9 g, 73% yield).

^{1}**H NMR** (400 MHz, $CDCl_3$) δ = 5.85 – 5.72 (m, 1H), 5.45 (ddt, J = 15.3, 7.8, 1.5 Hz, 1H), 4.28 (td, J = 8.0, 0.8 Hz, 1H), 3.80 – 3.64 (m, 3H), 2.12 – 1.97 (m, 2H), 1.43 – 1.39 (m, 6H), 1.39 – 1.33 (m, 2H), 1.31 – 1.20 (m, 6H), 0.90 (s, 12H), 0.10 – 0.02 (m, 6H)
^{13}**C NMR** (101 MHz, $CDCl_3$) δ = 136.6, 127.1, 108.8, 81.6, 79.2, 62.5, 32.5, 31.8, 29.1, 29.0, 27.3, 27.0, 26.1, 22.7, 18.5, 14.3, -5.2, -5.3
IR (neat, v_{max}/cm^{-1}) 2986, 2956, 2928, 1770, 1463, 1378, 1369, 1251, 1143, 1093, 837, 777
ESI-MS calcd for $C_{20}H_{40}NaO_3Si$ [M+Na]$^+$ 379.2639; found 379.2630
[α]$^{26}_D$ = 1.6 (c = 1.0, $CHCl_3$)

Synthesis of 11

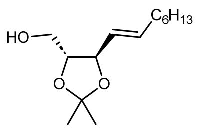

11

The corresponding compound was prepared from **S25** (7.9 g, 22 mmol, 1.0 equiv) following **general procedure B**. The reaction was stirred for 90 min resulting in title compound as colorless oil (5.2 g, 96% yield).

^{1}**H NMR** (500 MHz, $CDCl_3$) δ = 5.83 (dtd, J = 15.3, 6.8, 0.8 Hz, 1H), 5.43 (ddt, J = 15.3, 8.1, 1.5 Hz, 1H), 4.31 – 4.24 (m, 1H), 3.82 (dd, J = 12.0, 2.9 Hz, 1H), 3.76 (ddd, J = 8.6, 3.9, 2.9 Hz, 1H), 3.58 (dd, J = 12.0, 3.9 Hz, 1H), 2.11 – 2.00 (m, 2H), 1.46 – 1.41 (m, 6H), 1.37 (dddd, J = 13.1, 7.7, 4.0, 2.1 Hz, 2H), 1.33 – 1.22 (m, 6H), 0.91 – 0.86 (m, 3H)
^{13}**C NMR** (126 MHz, $CDCl_3$) δ = 137.6, 126.5, 109.0, 81.2, 78.4, 60.9, 32.5, 31.8, 29.0, 29.0, 27.3, 27.1, 22.7, 14.2
IR (neat, v_{max}/cm^{-1}) 3467, 2986, 2957, 2927, 2858, 1457, 1379, 1371, 1240, 1219, 1051, 968
ESI-MS calcd for $C_{14}H_{26}NaO_3$ [M+Na]$^+$ 265.1774; found 265.1769
[α]$^{25}_D$ = 4.5(c = 1.0, $CHCl_3$)

Synthesis of 4a

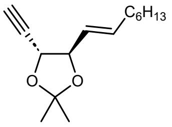

4a

The corresponding compound was prepared from **11** (0.43 g, 1.8 mmol, 1.0 equiv) following **general procedure C**. The reactions were stirred for 30 min and 90 min respectively resulting in title compound as pale-yellow oil (0.32 g, 76% yield) and recovered **11** (50 mg, 11% yield).

^{1}H NMR (400 MHz, $CDCl_3$) δ = 5.92 (dtd, J = 15.3, 6.8, 0.8 Hz, 1H), 5.42 (ddt, J = 15.3, 7.7, 1.5 Hz, 1H), 4.39 (td, J = 7.9, 0.8 Hz, 1H), 4.25 (dd, J = 8.0, 2.1 Hz, 1H), 2.51 (d, J = 2.0 Hz, 1H), 2.08 (dtd, J = 8.4, 6.9, 1.4 Hz, 2H), 1.54 – 1.35 (m, 8H), 1.34 – 1.19 (m, &H), 0.95 – 0.82 (m, 3H)
^{13}C NMR (101 MHz, $CDCl_3$) δ = 138.0, 124.8, 110.3, 83.0, 80.0, 74.9, 70.6, 32.5, 31.8, 29.0, 28.9, 27.1, 26.5, 22.7, 14.2
IR (neat, v_{max}/cm^{-1}) 3312, 2989, 2958, 2927, 2857, 1457, 1381, 1372, 1237, 1165, 1055, 967, 877
ESI-MS calcd for $C_{15}H_{25}O_2$ $[M+H]^+$ 237.1849; found 237.1851
[α]$^{25}_D$ = 6.3 (c = 1.0, $CHCl_3$)

Synthesis of *ent*-S25

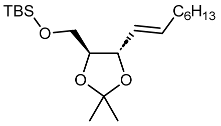

ent-**S25**

The corresponding compound was prepared from *ent*-**7** (0.93 g, 3.4 mmol, 1.0 equiv) following **general procedure A** using octene (5.0 equiv) and Grubbs 2nd Gen. catalyst (3.0 mol%). After 22 h octene (10 equiv) and GRUBBS 2nd generation catalyst (2.0 mol%) and after 46 h GRUBBS 2nd generation catalyst (2.0 mol%) were added. The reaction was stirred for 110 h resulting in title compound as a light-brown oil (1.0 g, 84% yield) and recovered *ent*-**7** (94 mg, 10% yield).

^{1}H NMR, ^{13}C NMR, IR, spectra were identical with its enantiomer *vide supra*.
ESI-MS calcd for $C_{20}H_{40}NaO_3Si$ $[M+Na]^+$ 379.2639; found 379.2633
[α]$^{25}_D$ = –3.17 (c = 0.5, $CHCl_3$)

Synthesis of *ent*-11

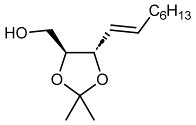

ent-**11**

The corresponding compound was prepared from *ent*-**S25** (1.0 g, 2.9 mmol, 1.0 equiv) following **general procedure B**. The reaction was stirred for 60 min resulting in title compound as colorless oil (0.52 g, 76% yield).

^{1}H NMR, ^{13}C NMR, IR spectra were identical with its enantiomer *vide supra*.
ESI-MS calcd for $C_{14}H_{26}NaO_3$ $[M+Na]^+$ 265.1774; found 265.1777
[α]$^{25}_D$ = –7.8 (c = 1.0, $CHCl_3$)

Synthesis of *ent*-4a

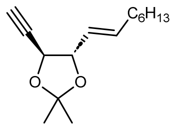

ent-**4a**

The corresponding compound was prepared from *ent*-**11** (0.36 g, 1.5 mmol, 1.0 equiv) following **general procedure C**. The reactions were stirred for 40 min and 2.5 h respectively resulting in title compound as pale-yellow oil (0.34 g, 72% yield) and recovered *ent*-**11** (51 mg, 14% yield).

^{1}H NMR, ^{13}C NMR, IR spectra were identical with its enantiomer *vide supra*.
ESI-MS calcd for $C_{15}H_{25}O_2$ $[M+H]^+$ 237.1849; found 237.1847
$[\alpha]^{25}_D$ = –20.2 (c = 1.0, $CHCl_3$)

3.2 Synthesis of Bromo Alkyne Fragment

(*S*)-**9** and (*R*)-**10** were prepared by enzymatic resolution (according to LIAN *et al*)[7] of 1-(trimethylsilyl)pent-1-yn-3-ol (**9**) prepared according to DENMARK and co-workers.[28]

Synthesis of (*S*)-5

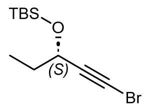

(*S*)-**5**

The corresponding compound was prepared from (*S*)-**9** (4.6 g, 30 mmol, 1.0 equiv) following **general procedure D**. Reactions were run in MeOH (0.20 M) with K_2CO_3 (2.0 equiv) and in CH_2Cl_2 (0.40 M). The reactions were stirred for 20 min, 80 min and 90 min respectively resulting in title compound as pale-yellow oil (4.8 g, 59% yield).

^{1}H NMR (400 MHz, $CDCl_3$) δ = 4.30 (t, J = 6.3 Hz, 1H), 1.68 (qd, J = 7.4, 6.3 Hz, 3H), 0.96 (t, J = 7.4 Hz, 3H), 0.90 (s, 9H), 0.12 (s, 3H), 0.10 (s, 3H)
^{13}C NMR (101 MHz, $CDCl_3$) δ = 81.7, 65.1, 43.6, 31.7, 25.8, 18.3, 9.6, -4.6, -5.1
IR (neat, v_{max}/cm^{-1}) 2958, 2930, 2858, 1728, 1463, 1253, 1112, 1069, 836, 777
$[\alpha]^{25}_D$ = –39.2 (c = 1.0, $CHCl_3$)
SFC ≥96% (Chiralpak IB; flow: 2.00 ml/min; 6.9 min (minor), 7.9 min (major); 90% CO_2, 10% MeOH at 100 bar, 25 °C) Enantiomeric excess was determined by SFC analysis of the corresponding 3,5-dinitrobenzoic esters **S26**.

Synthesis of (*R*)-5

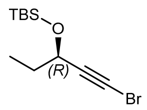

(*R*)-**5**

The corresponding compound was prepared from (*R*)-**10** (1.1 g, 5.3 mmol, 1.0 equiv) following **general procedure D**. Reactions were run in MeOH (0.10 M) with K_2CO_3 (1.5 equiv) and in CH_2Cl_2 (0.10 M) The reactions were stirred for 90 min, 2 h and 2 h respectively resulting in title compound as pale-yellow oil (0.56 mg, 38% yield).

^{1}H NMR, ^{13}C NMR, IR spectra were identical with its enantiomer *vide supra*.
$[\alpha]^{25}_D$ = 39.4 (c = 1.0, $CHCl_3$)
SFC ≥95% (Chiralpak IB; flow: 2.00 ml/min; 6.8 min (major), 7.9 min (minor); 90% CO_2, 10% MeOH at 100 bar, 25 °C). Enantiomeric excess was determined by SFC analysis of the corresponding 3,5-dinitrobenzoic esters **S26**.

3.3 Synthesis of syn-Fragment

(4*R*,5*S*)-**S6** and (4*S*,5*R*)-*ent*-**S6** were prepared from ribose in 2 steps according to MOON *et al.*[6]

Synthesis of S7

Although diol S7 had been previously synthesized by DAHLHOFF and later by YADAV by WITTIG olefination, the corresponding (Z)-olefin was obtained as the major product in both instances.[30,31]

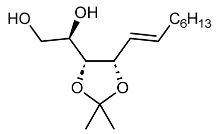

S7

The corresponding compound was prepared from **S6** (56 mg, 0.30 mmol, 1.0 equiv) following **general procedure E** using octene (5.0 equiv) and GRUBBS 2nd generation catalyst (10 mol%). The reaction was stirred for 22 h resulting in title compound as a light-brown oil (76 mg, 93% yield).

^{1}H NMR (400 MHz, $CDCl_3$) δ = 5.91 (dtd, J = 15.4, 6.7, 0.9 Hz, 1H), 5.66 – 5.55 (m, 1H), 4.71 – 4.63 (m, 1H), 4.13 – 3.92 (m, 1H), 3.87 – 3.78 (m, 1H), 3.81 – 3.67 (m, 2H), 2.17 – 1.95 (m, 2h), 1.46 (s, 3H), 1.42 – 1.37 (m, 2H), 1.35 (s, 3H), 1.32 – 1.24 (m, 6H), 0.90 – 0.85 (m, 3H)
^{13}C NMR (101 MHz, $CDCl_3$) δ = 137.2, 125.1, 109.0, 78.6, 78.5, 70.1, 64.5, 32.5, 31.8, 29.1, 29.1, 27.9, 25.3, 22.7, 14.2
IR (neat, v_{max}/cm^{-1}) 3416, 2927, 2856, 1475, 1379, 1217, 1167, 1058, 970, 874
ESI-MS calcd for $C_{15}H_{28}NaO_4$ $[M+Na]^+$ 295.1880; found 295.1879
$[\alpha]^{27}_D$ = 4.8 (c = 0.1, $CHCl_3$)

Synthesis of 4b

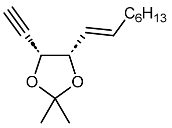

4b

The corresponding compound was prepared from **S7** (0.23 g, 0.84 mmol, 1.0 equiv) following **general procedure F**. The reactions were stirred for 2.5 h and 60 min respectively resulting in title compound as pale-yellow oil (90 mg, 46% yield).

^{1}H NMR (400 MHz, $CDCl_3$) δ = 5.87 (dtd, J = 15.4, 6.7, 0.7 Hz, 1H), 5.67 (ddt, J = 15.3, 8.3, 1.4 Hz, 1H), 4.73 (dd, J = 5.7, 2.2 Hz, 1H), 4.50 (dd, J = 8.3, 5.7, Hz, 1H), 2.53 (d, J = 2.2 Hz, 1H), 2.15 – 2.05 (m, 2H), 1.57 (s, 3H), 1.45 – 1.38 (m, 2H), 1.37 (s , 3H), 1.33 – 1.23 (m, 6H), 0.91 – 0.85 (m, 3H)
^{13}C NMR (101 MHz, $CDCl_3$) δ = 138.3, 124.7, 110.3, 80.4, 79.5, 76.0, 69.4, 32.5, 31.8, 29.0, 28.9, 27.9, 26.3, 22.8, 14.2
IR (neat, v_{max}/cm^{-1}) 3312, 2987, 2928, 2857, 1457, 1380, 1370, 1227, 1054, 968, 867
EI-MS calcd for $C_{14}H_{21}O_2$ $[M–CH_3]$ 221.536; found 221.536
$[\alpha]^{26}_D$ = 83.4 (c = 0.5, $CHCl_3$)

Synthesis of *ent*-S7

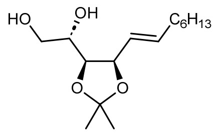

ent-**S7**

The corresponding compound was prepared from *ent*-**S6** (0.46 g, 2.4 mmol, 1.0 equiv) following **general procedure E** using octene (5.0 equiv) and GRUBBS 2nd generation catalyst (5.0 mol%). After 62 h octene (2.0 equiv) and GRUBBS 2nd generation catalyst (2.0 mol%) were added. The reaction was stirred for 160 h resulting in title compound as a light-brown oil (0.28 g, 42% yield).

^{1}H NMR, ^{13}C NMR, IR spectra were identical with its enantiomer *vide supra*.
ESI-MS calcd for $C_{15}H_{28}NaO_4$ $[M+Na]^+$ 295.1880; found 295.1878
$[\alpha]^{26}_{D}$ = –1.6 (c = 0.1, $CHCl_3$)

Synthesis of *ent*-4b

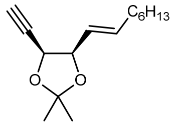

ent-**4b**

The corresponding compound was prepared from *ent*-**S7** (0.23 g, 0.85 mmol, 1.0 equiv) following **general procedure F**. The reactions were stirred for 2.5 h and 60 min respectively resulting in title compound as pale-yellow oil (0.10 g, 50% yield).

^{1}H NMR, ^{13}C NMR, IR spectra were identical with its enantiomer *vide supra*.
EI-MS calcd for $C_{12}H_{19}O$ [M–acetone] 179.1430; found 179.1429
$[\alpha]^{27}_{D}$ = –78.5 (c = 0.5, $CHCl_3$)

3.4 Synthesis of Isofalcarintriols

Synthesis of S15a

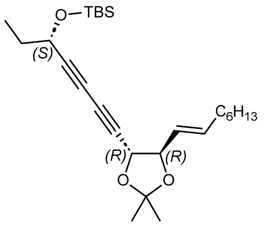

15Sa

The corresponding compound was prepared from **4a** (1.2 g, 5.2 mmol, 1.0 equiv) and (*S*)-**5** (1.6 g, 5.7 mmol, 1.1 equiv) following **general procedure G**. After 75 min additional copper(I) chloride (1.0 mol%) in *n*-BuNH$_2$ (30% in water, 5.0 equiv) and (*S*)-**5** (0.10 equiv) were added. The reaction was stirred 4.5 h resulting in title compound as pale-yellow oil (2.1 g, 91% yield) and recovered **4a** (72 mg, 6% yield).

^{1}H NMR (500 MHz, CDCl$_3$) δ = 5.92 (dtd, J = 15.4, 6.8, 0.9 Hz, 1H), 5.41 (ddt, J = 15.3, 7.7, 1.5 Hz, 1H), 4.41 (td, J = 7.7, 0.8 Hz, 1H), 4.37 – 4.29 (m, 2H), 2.12 – 2.03 (m, 2H), 1.69 (qd, J = 7.4, 6.3 Hz, 2H), 1.47 (d, J = 0.7 Hz, 3H), 1.42 (d, J = 0.7 Hz, 3H), 1.41 – 1.35 (m, 2H), 1.33 – 1.23 (m, 6H), 0.97 (t, J = 7.4 Hz, 3H), 0.90 (s, 12H), 0.12 (s, 3H), 0.10 (s, 3H)
^{13}C NMR (126 MHz, CDCl$_3$) δ = 138.1, 124.8, 110.5, 82.7, 81.8, 74.7, 71.2, 71.0, 68.2, 64.6, 32.5, 31.8, 31.7, 29.0, 28.9, 27.1, 26.4, 25.9, 22.7, 18.4, 14.2, 9.6, -4.5, -5.0
IR (neat, ν_{max}/cm^{-1}) 2957, 2929, 2857, 1464, 1380, 1340, 1252, 1109, 1052, 838, 778
ESI-MS calcd for $C_{26}H_{44}NaO_3Si$ [M+Na]$^+$ 455.2952; found 455.2944
$[\alpha]^{25}_D$ = 18.6 (c = 1.0, CHCl$_3$)

Synthesis of 1a

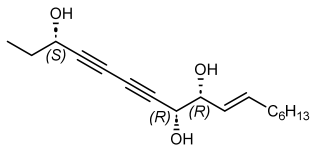

1a

The corresponding compound was prepared from **S15a** (1.1 g, 2.6 mmol, 1.0 equiv) following **general procedure I**. The reaction was stirred 23 h resulting in title compound as pale-yellow oil (0.67 g, 94% yield).

^{1}H NMR (500 MHz, CDCl$_3$) δ = 5.86 (dtd, J = 15.4, 6.9, 1.1 Hz, 1H), 5.50 (ddt, J = 15.4, 6.9, 1.5 Hz, 1H), 4.37 (t, J = 6.5 Hz, 1H), 4.26 (d, J = 6.7, 1H), 4.12 (t, J = 6.7 Hz, 1H), 2.12 – 2.03 (m, 2H), 1.79 – 1.71 (m, 2H), 1.46 – 1.36 (m, 2H), 1.34 – 1.21 (m, 6H), 1.01 (t, J = 7.4 Hz, 3H), 0.91 – 0.86 (m, 3H)
^{13}C NMR (126 MHz, CDCl$_3$) δ = 136.7, 126.6, 80.7, 77.5, 75.6, 70.6, 69.0, 66.7, 64.1, 32.5, 31.9, 30.7, 29.1, 29.0, 22.8, 14.3, 9.5
IR (neat, ν_{max}/cm^{-1}) 3351, 2959, 2927, 2856, 1671, 1459, 1337, 1095, 1052, 1016, 968
ESI-MS calcd for $C_{17}H_{26}NaO_3$ [M+Na]$^+$ 301.1774; found 301.1772
$[\alpha]^{25}_D$ = 33.7 (c = 1.0, CHCl$_3$)

Synthesis of *ent*-S15a

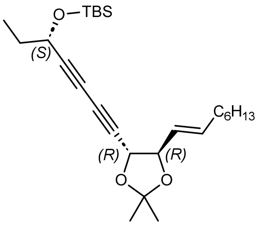

S15a

The corresponding compound was prepared from **4a** (87 mg, 0.37 mmol, 1.0 equiv) and (*R*)-**5** (0.11 g, 0.41 mmol, 1.1 equiv) following **general procedure G**. After 2 h additional copper(I) chloride (2.0 mol%) in *n*-BuNH$_2$ (30% in water, 10 equiv) and (*R*)-**5** (0.20 equiv) were added. The reaction was stirred 3 h resulting in title compound as pale-yellow oil (0.14 g, 87% yield).

^{1}H NMR, ^{13}C NMR, IR spectra were identical with its enantiomer *vide supra*.
ESI-MS calcd for $C_{26}H_{44}NaO_3Si$ $[M+Na]^+$ 455.2952; found 455.2950
$[\alpha]^{25}_D$ = –25.5 (c = 0.5, $CHCl_3$)

Synthesis of *ent*-1a

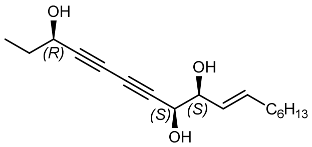

ent-**1a**

The corresponding compound was prepared from *ent*-***S*15Sa** (5.0 mg, 10 mmol, 1.0 equiv) following **general procedure H**. The reaction was stirred 42 h resulting in title compound as pale-yellow oil (2.8 mg, 99% yield).

^{1}H NMR, ^{13}C NMR, IR spectra were identical with its enantiomer *vide supra*.
ESI-MS calcd for $C_{17}H_{26}NaO_3$ $[M+Na]^+$ 301.1774; found 301.1774
$[\alpha]^{25}_D$ = –25.7 (c = 0.25, $CHCl_3$)

Synthesis of S15b

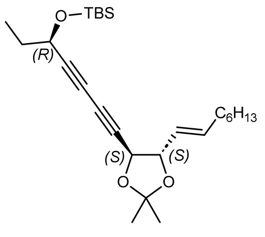

ent-**S15a**

The corresponding compound was prepared from *ent*-**4a** (87 mg, 0.37 mmol, 1.0 equiv) and (*S*)-**5** (0.11 g, 0.41 mmol, 1.1 equiv) following **general procedure G**. After 2 h additional copper(I) chloride (2.0 mol%) in *n*-BuNH2 (30% in water, 10 equiv) and **52a** (0.20 equiv) were added. The reaction was stirred 3 h resulting in title compound as pale-yellow oil (0.10 g, 61% yield) and recovered *ent*-**4a** (34 mg, 35% yield).

^{1}H NMR (500 MHz, $CDCl_3$) δ = 5.92 (dtd, J = 15.4, 6.7, 0.9 Hz, 1H), 5.41 (ddt, J = 15.3, 7.7, 1.5 Hz, 1H), 4.41 (td, J = 7.7, 0.8 Hz, 1H), 4.37 – 4.29 (m, 2H), 2.12 – 2.03 (m, 2H), 1.69 (qd, J = 7.4, 6.3 Hz, 2H), 1.47 (s, 3H), 1.42 (s, 3H), 1.41 – 1.36 (m, 2H), 1.34 – 1.22 (m, 6H), 0.97 (t, J = 7.4 Hz, 3H), 0.92 – 0.85 (m, 12H), 0.13 (s, 3H), 0.10 (s, 3H)
^{13}C NMR (126 MHz, $CDCl_3$) δ = 138.1, 124.8, 110.5, 82.7, 81.8, 74.7, 71.2, 71.0, 68.2, 64.6, 32.5, 31.8, 31.7, 29.0, 28.9, 27.1, 26.4, 25.9, 22.7, 18.4, 14.2, 9.6, -4.4, -5.05
IR (neat, ν_{max}/cm^{-1}) 2957, 2929, 2857, 1463, 1380, 1340, 1252, 1226, 1109, 1052, 838, 778
ESI-MS calcd for $C_{26}H_{44}NaO_3Si$ $[M+Na]^+$ 455.2952; found 455.2944
$[\alpha]^{25}_D$ = –88.2 (c = 0.25, $CHCl_3$)

Synthesis of 1b

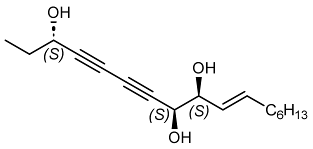

1b

The corresponding compound was prepared from **S15b** (37 mg, 90 mmol, 1.0 equiv) following **general procedure H**. The reaction was stirred 26 h resulting in title compound as pale-yellow oil (24 mg, 99% yield).

^{1}H NMR (500 MHz, $CDCl_3$) δ = 5.86 (dtd, J = 15.4, 6.8, 1.1 Hz, 1H), 5.50 (ddt, J = 15.4, 6.9, 1.5 Hz, 1H), 4.37 (t, J = 6.5 Hz, 1H), 4.26 (dd, J = 6.5, 0.8 Hz, 1H), 4.12 (t, J = 6.7 Hz, 1H), 2.12 – 2.03 (m, 2H), 1.74 (qdd, J = 7.3, 6.4, 3.0 Hz, 2H), 1.46 – 1.36 (m, 2H), 1.34 – 1.21 (m, 6H), 1.01 (t, J = 7.5 Hz, 3H), 0.91 – 0.86 (m, 3H)
^{13}C NMR (126 MHz, $CDCl_3$) δ = 136.7, 126.6, 80.7, 77.5, 75.6, 70.6, 69.0, 66.7, 64.1, 32.5, 31.9, 30.7, 29.1, 29.0, 22.8, 14.3, 9.5
IR (neat, ν_{max}/cm^{-1}) 3341, 2958, 2925, 2855, 1671, 1458, 1335, 1095, 1050, 1015, 967
ESI-MS calcd for $C_{17}H_{26}NaO_3$ $[M+Na]^+$ 301.1774; found 301.1775
$[\alpha]^{25}_D$ = –26.4 (c = 0.25, $CHCl_3$)

Synthesis of *ent*-15Sb

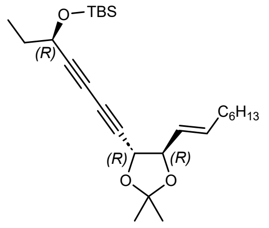

ent-**15Sb**

The corresponding compound was prepared from **4a** (75 mg, 0.32 mmol, 1.0 equiv) and (*R*)-**5** (97 mg, 0.35 mmol, 1.1 equiv) following **general procedure G**. After 75 min additional copper(I) chloride (2.0 mol%) in *n*-$BuNH_2$ (30% in water, 10 equiv) and (*R*)-**5** (0.20 equiv) were added. The reaction was stirred 2 h resulting in title compound as pale-yellow oil (93 mg, 67% yield).

Synthesis of *ent*-1b

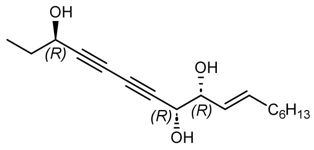

ent-**1b**

The corresponding compound was prepared from *ent*-**15Sb** (30 mg, 90 mmol, 1.0 equiv) following **general procedure H**. The reaction was stirred 40 h resulting in title compound as pale-yellow oil (18 mg, 87% yield).

^{1}H NMR, ^{13}C NMR, IR spectra were identical with its enantiomer *vide supra*.
ESI-MS calcd for $C_{17}H_{26}NaO_3$ $[M+Na]^+$ 301.1774; found 301.1772
$[\alpha]^{25}_{D}$ = 43.5 (c = 1.0, $CHCl_3$)

Synthesis of S15c

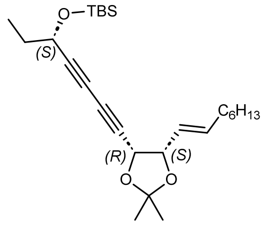

S15c

The corresponding compound was prepared from **4b** (30 mg, 0.18 mmol, 1.0 equiv) and (*S*)-**5** (59 mg, 0.21 mmol, 1.2 equiv) following **general procedure G**. After 2 h additional copper(I) chloride (2.0 mol%) in *n*-$BuNH_2$ (30% in water, 10 equiv) and after 3 h (*S*)-**5** (0.20 equiv) were added. The reaction was stirred 6 h resulting in title compound as pale-yellow oil (37 mg, 49% yield).

^{1}H NMR (500 MHz, $CDCl_3$) δ = 5.87 (dtd, J = 15.4, 6.7, 0.7 Hz, 1H), 5.63 (ddt, J = 15.3, 8.3, 1.5 Hz, 1H), 4.79 (dt, J = 5.7, 0.9 Hz, 1H), 4.50 (ddd, J = 8.3, 5.7, 0.8 Hz, 1H), 4.34 (td, J = 6.4, 0.8 Hz, 1H), 2.14 – 2.06 (m, 2H), 1.69 (qd, J = 7.4, 6.5 Hz, 2H), 1.56 (d, J = 0.8 Hz, 3H), 1.45 – 1.38 (m, 2H), 1.38 – 1.35 (m, 3H), 1.33 – 1.21 (m, 6H), 0.96 (t, J = 7.4 Hz, 3H), 0.92 – 0.87 (m, 12H), 0.13 (s, 3H), 0.10 (s, 3H)
^{13}C NMR (126 MHz, $CDCl_3$) 138.4, 124.5, 110.5, 81.6, 79.9, 75.31, 72.0, 70.1, 68.4, 64.7, 32.5, 31.8, 31.7, 29.0, 28.9, 27.9, 26.3, 25.9, 22.8, 18.4, 14.3, 9.7, -4.4, -4.9
IR (neat, v_{max}/cm^{-1}) 2957, 2929, 2857, 1463, 1380, 1370, 1251, 1226, 1109, 1052, 837, 778
ESI-MS calcd for $C_{26}H_{44}NaO_3Si$ $[M+Na]^+$ 455.2952; found 455.2950
$[\alpha]^{26}_{D}$ = 78.6 (c = 1.0, $CHCl_3$)

Synthesis of 1c

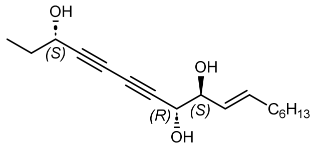

1c

The corresponding compound was prepared from **S15c** (30 mg, 70 mmol, 1.0 equiv) following **general procedure H**. The reaction was stirred 17 h resulting in title compound as pale-yellow wax (12 mg, 60% yield).

^{1}H NMR (400 MHz, $CDCl_3$) δ = 5.84 (dtd, J = 15.1, 6.8, 1.2 Hz, 1H), 5.54 (ddt, J = 15.4, 6.7, 1.5 Hz, 1H), 4.42 – 4.31 (m, 2H), 4.24 – 4.17 (m, 1H), 2.13 – 2.02 (m, 2H), 1.82 – 1.67 (m, 2H), 1.40 (dd, J = 14.8, 7.4 Hz, 2H), 1.36 – 1.22 (m, 6H), 1.02 (t, J = 7.4 Hz, 3H), 0.95 – 0.84 (m, 3H)
^{13}C NMR (101 MHz, $CDCl_3$) δ = 136.4, 126.7, 80.5, 77.1, 75.1, 70.8, 69.0, 66.8, 64.1, 32.5, 31.8, 30.7, 29.1, 29.0, 22.8, 14.2, 9.5
IR (neat, v_{max}/cm^{-1}) 3352, 2958, 2926, 2856, 1460, 1379, 1096, 1052, 1019, 967
ES-MS calcd for $C_{17}H_{24}O_2$ [M–H_2O] 260.17708; found 260.17691
[α]$^{24}_{D}$ = 65.2 (c = 0.25, $CHCl_3$)

Synthesis of *ent*-S15c

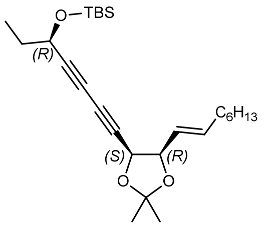

ent-**S15c**

The corresponding compound was prepared from *ent*-**4b** (40 mg, 0.17 mmol, 1.0 equiv) and (*R*)-**5** (57 mg, 0.21 mmol, 1.2 equiv) following **general procedure G**. After 3 h additional copper(I) chloride (2.0 mol%) in *n*-$BuNH_2$ (30% in water, 10 equiv) and after 4 h (*R*)-**5** (0.30 equiv) were added. The reaction was stirred 7 h resulting in title compound as pale-yellow oil (34 mg, 47% yield) and recovered *ent*-**4b** (13 mg, 33% yield).

^{1}H NMR, ^{13}C NMR, IR spectra were identical with its enantiomer *vide supra*.
ESI-MS calcd for $C_{26}H_{44}NaO_3Si$ $[M+Na]^+$ 455.2952; found 455.2950
[α]$^{26}_{D}$ = –85.6 (c = 0.5, $CHCl_3$)

Synthesis of *ent*-1c

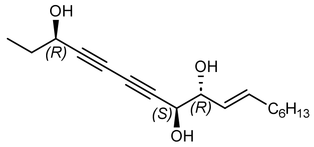

ent-**1c**

The corresponding compound was prepared from *ent*-**S15c** (14 mg, 30 mmol, 1.0 equiv) following **general procedure H**. The reaction was stirred 40 h resulting in title compound as pale-yellow wax (8.0 mg, 90% yield).

^{1}H NMR, ^{13}C NMR, IR spectra were identical with its enantiomer *vide supra*.
ES-MS calcd for $C_{17}H_{24}O_2$ [M–H_2O] 260.17708; found 260.17697
[α]$^{24}_{D}$ = –54.6 (c = 0.25, $CHCl_3$)

Synthesis of S15d

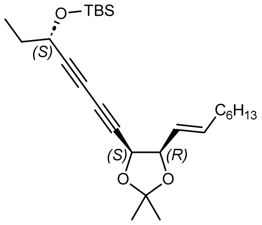

S15d

The corresponding compound was prepared from *ent*-**4b** (40 mg, 0.17 mmol, 1.0 equiv) and (*S*)-**5** (57 mg, 0.21 mmol, 1.2 equiv) following **general procedure G**. After 3 h additional copper(I) chloride (2.0 mol%) in *n*-BuNH$_2$ (30% in water, 10 equiv) and after 4 h (*S*)-**5** (0.30 equiv) were added. The reaction was stirred 7 h resulting in title compound as pale-yellow oil (44 mg, 60% yield) and recovered *ent*-**4b** (15 mg, 36% yield).

^{1}**H NMR** (500 MHz, $CDCl_3$) δ = 5.86 (dtd, J = 15.4, 6.7, 0.7 Hz, 1H), 5.63 (ddt, J = 15.3, 8.3, 1.4 Hz, 1H), 4.78 (dt, J = 5.7, 0.8 Hz, 1H), 4.50 (ddd, J = 8.3, 5.8, 0.8 Hz, 1H), 4.34 (td, J = 6.4, 0.7 Hz, 1H), 2.14 – 2.06 (m, 2H), 1.69 (qd, J = 7.3, 5.7 Hz, 2H), 1.55 (s, 3H), 1.45 – 1.38 (m, 1H), 1.36 (s, 3H), 1.34 – 1.23 (m, 6H), 0.96 (t, J = 7.4 Hz, 3H), 0.91 – 0.86 (m, 12H), 0.13 (s, 3H), 0.10 (s, 3H).
^{13}**C NMR** (126 MHz, $CDCl_3$) δ = 138.4, 124.5, 110.5, 81.6, 79.9, 75.3, 72.0, 70.1, 68.4, 64.7, 32.5, 31.8, 31.7, 29.0, 28.9, 27.9, 26.3, 25.9, 22.8, 18.4, 14.3, 9.7, -4.5, -4.9
IR (neat, v_{max}/cm^{-1}) 2957, 2929, 2857, 1464, 1380, 1370, 1251, 1226, 1109, 1053, 865, 778
ESI-MS calcd for $C_{26}H_{44}NaO_3Si$ [M+Na]$^+$ 455.2952; found 455.2948
[α]^{25}$_D$ = –185.5 (c = 1.0, $CHCl_3$)

Synthesis of 1d

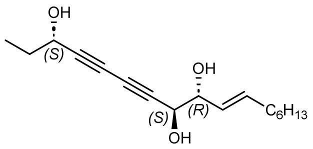

1d

The corresponding compound was prepared from **S15d** (13 mg, 30 mmol, 1.0 equiv) following **general procedure H**. The reaction was stirred 40 h resulting in title compound as pale-yellow wax (5.8 mg, 67% yield).

^{1}**H NMR** (400 MHz, $CDCl_3$) δ = 5.83 (dtd, J = 15.1, 6.8, 1.2 Hz, 1H), 5.54 (ddt, J = 15.4, 6.7, 1.5 Hz, 1H), 4.42 – 4.31 (m, 2H), 4.24 – 4.17 (m, 1H), 2.08 (q, J = 6.7 Hz, 2H), 1.82 – 1.67 (m, 2H), 1.44 – 1.35 (m, 2H), 1.33 – 1.19 (m, 6H), 1.02 (t, J = 7.4 Hz, 3H), 0.95 – 0.83 (m, 3H)
^{13}**C NMR** (101 MHz, $CDCl_3$) δ = 136.4, 126.7, 80.5, 77.1, 75.1, 70.8, 69.0, 66.82, 64.1, 32.5, 31.8, 30.7, 29.1, 29.0, 22.8, 14.3, 9.5
IR (neat, v_{max}/cm^{-1}) 3348, 2962, 2926, 2856, 1463, 1375, 1096, 1068, 1017, 970
ES-MS calcd for $C_{17}H_{24}O_2$ [M–H_2O] 260.17708; found 260.17666
[α]^{24}$_D$ = –52.0 (c = 0.25, $CHCl_3$)

Synthesis of *ent*-S15d

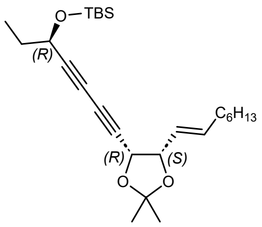

ent-**S15d**

The corresponding compound was prepared from **4d** (42 mg, 0.18 mmol, 1.0 equiv) and (*R*)-**5** (59 mg, 0.21 mmol, 1.2 equiv) following **general procedure G**. After 2 h additional copper(I) chloride (2.0 mol%) in *n*-$BuNH_2$ (30% in water, 10 equiv) and after 3 h (*R*)-**5** (0.20 equiv) were added. The reaction was stirred 6 h resulting in title compound as pale-yellow oil (45 mg, 58% yield).

^{1}H NMR, ^{13}C NMR, IR spectra were identical with its enantiomer *vide supra*.
ESI-MS calcd for $C_{26}H_{44}NaO_3Si$ $[M+Na]^+$ 455.2952; found 455.2952
$[\alpha]^{26}_D$ = 166.9 (c = 1.0, $CHCl_3$)

Synthesis of *ent*-1d

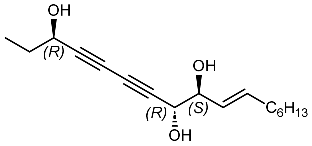

ent-**1d**

The corresponding compound was prepared from *ent*-**S15d** (30 mg, 70 mmol, 1.0 equiv) following **general procedure H**. The reaction was stirred 17 h resulting in title compound as pale-yellow wax (13 mg, 66% yield).

^{1}H NMR, ^{13}C NMR, IR spectra were identical with its enantiomer *vide supra*.
ES-MS calcd for $C_{17}H_{24}O_2$ $[M–H_2O]$ 260.17708; found 260.17691
$[\alpha]^{25}_D$ = 63.6 (c = 0.25, $CHCl_3$)

4 Configurational Assignment of Isofalcarintriol (1a)

#NP017896 (AnalytiCon Discovery GmbH, Potsdam, Germany)

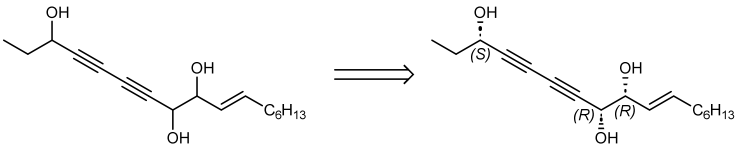

1 proposed — **1a** confirmed and assigned

^{1}H NMR (500 MHz, $CDCl_3$) δ = 5.86 (dtd, J = 15.0, 6.9, 1.1 Hz, 1H), 5.51 (ddt, J = 15.4, 6.9, 1.4 Hz, 1H), 4.38 (td, J = 6.4, 0.7 Hz, 1H), 4.27 (dd, *J* = 6.5, 0.8 Hz, 1H), 4.13 (t, *J* = 7.1 Hz, 2H)*, 2.08 (q, J = 7.2 Hz, 2H), 1.75 (qdd, J = 7.3, 6.4, 2.7 Hz, 2H), 1.41 – 1.34 (m, 2H), 1.34 – 1.21 (m, 12H)*, 1.01 (t, J = 7.4 Hz, 3H), 0.88 (td, J = 7.0, 0.8 Hz, 4H)*

^{13}C NMR (126 MHz, $CDCl_3$) δ = 136.7, 126.7, 80.4, 77.4, 75.7, 70.6, 69.0, 66.8, 64.2, 32.5, 31.8, 30.8, 29.1, 29.0, 22.7, 14.3, 9.5

IR (neat, v_{max}/cm^{-1}) 3383, 2928, 2856, 1710, 1609, 1461, 1379, 1271, 1165, 1050, 968

$[\alpha]^{28}_D$ = 17.0 (c = 0.05, $CHCl_3$)

SFC ≥99% (Chiralpak IA; flow: 2.00 ml/min; 7.9 min (major); 90% CO_2, 10% MeOH at 100 bar, 25 °C)

* = Overlapping impurities in the spectra

5 *Natural abundance of isofalcarintriol*

5.1 Synthesis of Isofalcarintriol-d_5

Synthesis of S9

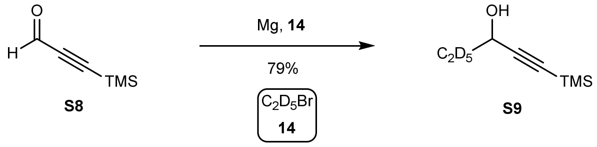

Mg turnings (88 mg, 3.6 mmol, 1.2 equiv) were covered with diethyl ether and a single crystal of iodine was added. Subsequently, a solution of bromoethane-d_5 (**14**) (0.41 g, 3.6 mmol, 1.2 equiv) in diethyl ether (0.40 M) was slowly added under reflux. The reaction was maintained at reflux for 60 min. A solution of aldehyde **S8** (0.45 ml, 3.0 mmol, 1.0 equiv) in diethyl ether (0.40 M) was slowly added under reflux. The reaction was maintained at reflux for another 30 min. After allowing the reaction to reach rt, it was quenched with 1.0 M aq. HCl solution and the crude reaction was extracted with diethyl ether. The combined organic layers were dried over Na_2SO_4, filtered, and concentrated under reduced pressure. The crude mixture was purified by flash column chromatography (pentane–diethyl ether, 6:1) resulting in title compound as pale-yellow oil (0.38 g, 79% yield).

^{1}H NMR (500 MHz, $CDCl_3$) δ = 4.30 (d, J = 4.9 Hz, 1H), 1.76 (d, J = 5.4 Hz, 1H), 0.17 (s, 9H)
^{2}H NMR (92 MHz, $CDCl_3$) δ = 1.66 (d, J = 2.1 Hz, 2D), 0.95 (s, 3D)
^{13}C NMR* (126 MHz, $CDCl_3$) δ = 106.8, 89.6, 64.2, 0.0
* CD_2CD_3 not visible.
IR (neat, v_{max}/cm^{-1}) 3328, 2961, 2900, 2224, 2174, 1280, 1068, 1000, 838, 759
ESI-MS calcd for $C_8H_{11}D_5NaOSi$ [M+Na]$^+$ 184.1176; found 184.117

Synthesis of (*S*)-S9 and (*R*)-S10

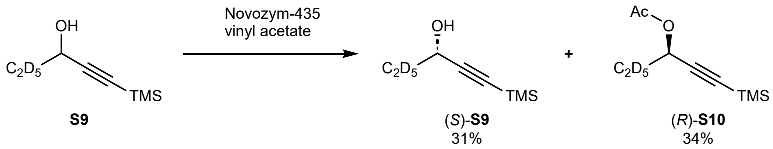

To a solution of **S9** (0.32 g, 2.0 mmol, 1.0 equiv) in vinyl acetate (0.42 M) was added Novozyme-435 (immobilized on acrylic resin) (50 mg, 5000 u/g). The reaction was stirred at rt for 40 h. The reaction was stopped, filtered through a short silica plug and concentrated under reduced pressure. The crude mixture was purified by flash column chromatography (hexane–ethyl acetate, 10:1) resulting in (*S*)-**S9** as a pale-yellow oil (0.10 g, 31% yield) and (*R*)-**S10** (0.14 g, 34% yield).

(*S*)-**S9**
^{1}H NMR (500 MHz, $CDCl_3$) δ = 4.30 (d, J = 4.9 Hz, 1H), 1.76 (d, J = 5.4 Hz, 1H), 0.17 (s, 9H)
^{2}H NMR (92 MHz, $CDCl_3$) δ = 1.66 (d, J = 2.1 Hz, 2D), 0.95 (s, 3D)
^{13}C NMR (126 MHz, $CDCl_3$) δ = 106.8, 89.6, 64.2, 29.8*, 8.2*, 0.0
* CD_2CD_3 assigned by HMBC.
IR (neat, v_{max}/cm^{-1}) 3343, 2959, 2925, 2855, 2223, 2174, 1250, 1069, 1000, 840, 760
ESI-MS calcd for $C_8H_{11}D_5NaOSi$ [M+Na]$^+$ 184.1176; found 184.117
$[\alpha]^{25}_D$ = –2.7 (c = 0.25, $CHCl_3$)

(*R*)-**S10**
^{1}H NMR (400 MHz, $CDCl_3$) δ = 5.33 (s, 1H), 2.08 (s, 3H), 0.17 (s, 9H)
^{2}H NMR (92 MHz, $CDCl_3$) δ = 1.71 (s, 2D), 0.94 (s, 3D).
^{13}C NMR (101 MHz, $CDCl_3$) δ = 170.1, 102.7, 90.5, 65.5, 27.2*, 21.2, 8.2*, 0.0
* CD_2CD_3 assigned by HMBC.
IR (neat, v_{max}/cm^{-1}) 2960, 2927, 2855, 2229, 2181, 1743, 1230, 1063, 1021, 841, 730
ESI-MS calcd for $C_{10}H_{13}D_5NaO_2Si$ [M+Na]$^+$ 226.1282; found 226.1286

[α]^{24}D = 93.2 (c = 0.25, $CHCl_3$)

Synthesis of 13

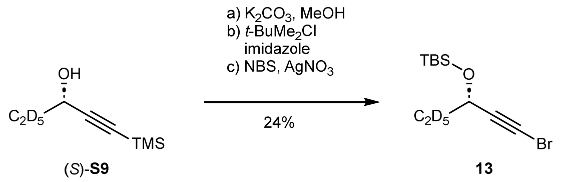

The corresponding compound was prepared from (*S*)-**S9** (81 mg, 0.50 mmol, 1.0 equiv) following **general procedure D**. Reactions were run in MeOH (0.20 M) with K_2CO_3 (1.5 equiv) and in CH_2Cl_2 (0.40 M). The reactions were stirred for 20 min, 80 min and 90 min respectively resulting in title compound as pale-yellow oil (34 mg, 24% yield).

^{1}H NMR (500 MHz, $CDCl_3$) δ = 4.29 (s, 1H), 0.90 (s, 9H), 0.12 (s, 3H), 0.10 (s, 3H)
^{13}C NMR (126 MHz, $CDCl_3$) δ = 81.6, 64.9, 43.5, 30.8*, 25.7, 18.14, 8.1*, -4.7, -5.2
* CD_2CD_3 assigned by HMBC.
IR (neat, v_{max}/cm^{-1}) 2930, 2859, 1770, 1759, 1247, 1057
[α]^{25}D = –36.2 (c = 0.5, $CHCl_3$)
SFC ≥94% (Chiralpak IB; flow: 2.00 ml/min; 6,0 min (minor), 6,9 min (major); 90% CO_2, 10% MeOH at 100 bar, 25 °C) Enantiomeric excess was determined by SFC analysis of the corresponding 3,5-dinitrobenzoic esters **S27**.

Synthesis of S11

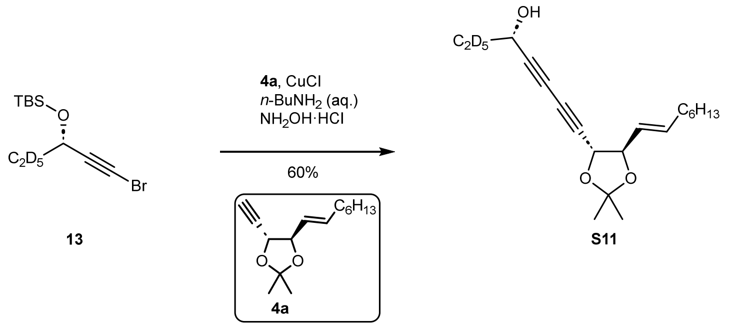

The corresponding compound was prepared from **4a** (19 mg, 78 µmol, 1.1 equiv) and **13** (19 mg, 68 µmol, 1.0 equiv) following **general procedure G**. The reaction was stirred 60 min resulting in title compound as pale-yellow oil (18 mg, 60% yield).

^{1}H NMR (400 MHz, $CDCl_3$) δ = 5.92 (dtd, J = 15.3, 6.8, 0.8 Hz, 1H), 5.41 (ddt, J = 15.3, 7.7, 1.5 Hz, 1H), 4.41 (td, J = 7.8, 0.9 Hz, 1H), 4.31 (d, J = 7.6 Hz, 2H), 2.12 – 2.02 (m, 2H), 1.47 (s, 3H), 1.42 (s, 3H), 1.41 – 1.36 (m, 2H), 1.36 – 1.20 (m, 6H), 0.89 (s, 12H), 0.12 (s, 3H), 0.09 (s, 3H)
^{13}C NMR (101 MHz, $CDCl_3$) δ = 138.2, 124.8, 110.5, 82.7, 81.8, 74.7, 71.2, 71.0, 68.1, 64.5, 32.5, 31.8, 30.5*, 29.0, 28.8, 27.1, 26.3, 25.9, 22.7, 18.4, 14.2, 8.0*, -4.5, -5.0
* CD_2CD_3 assigned by HMBC.
IR (neat, v_{max}/cm^{-1}) 2957, 2929, 2858, 2226, 1770, 1463, 1381, 1250, 1083, 1054, 838, 778
ESI-MS calcd for $C_{26}H_{39}D_5NaO_3Si$ $[M+Na]^+$ 460.3266; found 460.3266
[α]^{25}D = 36.4 (c = 0.5, $CHCl_3$)

Synthesis of 12

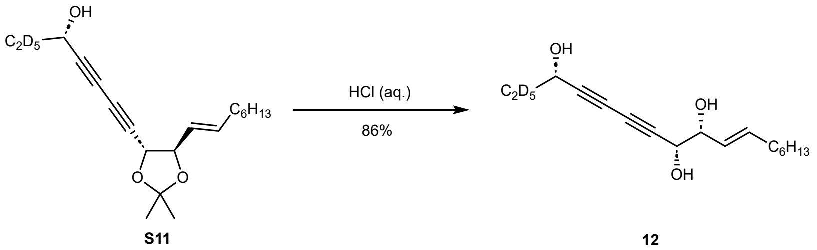

The corresponding compound was prepared from **S11** (13 mg, 29 mmol, 1.0 equiv) following **general procedure I**. The reaction was stirred 16 h resulting in title compound as pale-yellow oil (7.0 mg, 86% yield).

^{1}H NMR (500 MHz, $CDCl_3$) δ = 5.87 (dtd, J = 15.1, 6.9, 1.1 Hz, 1H), 5.51 (ddt, J = 15.4, 6.9, 1.5 Hz, 1H), 4.37 (d, J = 4.2 Hz, 1H), 4.30 – 4.24 (m, 1H), 4.13 (t, J = 6.8 Hz, 1H), 2.46 (s, 1H), 2.32 (s, 1H), 2.14 – 1.99 (m, 2H), 1.89 (s, 1H), 1.48 – 1.36 (m, 2H), 1.36 – 1.26 (m, 6H), 0.89 (td, J = 6.9, 3.6 Hz, 3H)
^{2}H NMR (92 MHz, $CDCl_3$) δ = δ 1.70 (s, 2D), 0.96 (s, 3D)
^{13}C NMR (126 MHz, $CDCl_3$) δ = 136.7, 126.7, 80.7, 77.4, 75.7, 70.6, 69.0, 66.8, 64.1, 32.5, 31.8, 29.8*, 29.0, 28.9, 22.8, 14.3, 8.7*
* CD_2CD_3 assigned by HMBC.
IR (neat, v_{max}/cm^{-1}) 3343, 2956, 2925, 2856, 1770, 1759, 1378, 1247, 1056
ESI-MS calcd for $C_{17}H_{21}D_5NaO_3$ [M+Na]$^+$ 306.2088; found 306.2090
[α]^{24}$_D$ = 37.5 (c = 0.5, $CHCl_3$)

5.2 Quantification of Isofalcarintriol from *D. Carota*

For the quantification of **1a** in the crude extracts a liquid chromatography–mass spectrometry method (LC-MS) was developed. Towards this end, penta-deuterated (3*S*,8*R*,9*R*,*E*)-heptadeca-10-en-4,6-diyne-1,1,1,2,2-*d*$_5$-3,8,9-triol (**12**) was synthesized to be used as internal standard and for peak identification in complex matrices. For each sample a solution (water-acetonitrile 98:2) containing 5.0 mg/ml extract and 1.25*10^{-2} mg/ml **12** as internal standard was prepared. Quantification was conducted via standard addition method. In short, to 200 µl of the aforementioned solution external standard **1a** was added and the samples were filled up to 250 µl solution resulting in 4-6 samples each with final concentrations of 4.0 mg/ml extract and 1.0*10^{-2} mg/ml **12** internal standard and ranging from 1.0*10^{-4} to 5.0*10^{-3} mg/ml of **1a** for standard addition (appropriately chosen to the observed abundance of **1a**). All dilutions were verified gravimetrically and corrected accordingly for data analysis. Samples (4 ml injected) were separated on an Agilent LC system (1290 series, Bruker Compass Hystar 5.0 service pack 1) using an Agilent Eclipse Plus C18, 3.0x1500, 3.5µm column at room temperature connected to a Bruker maXisII - ESI-Qq-TOF-MS. The LC mobile phase (0.6 ml/min flow) consisted of water-acetonitrile (98:2), after two minutes changed with a linear gradient up to (50:50) over 14 minutes and as modifier 0.1% formic acid was employed. Chromatograms were processed and analyzed using Bruker Compass DataAnalysis 5.3. The measured area counts were corelated by linear regression using GraphPad Prism 9.3.0.

Entry 2

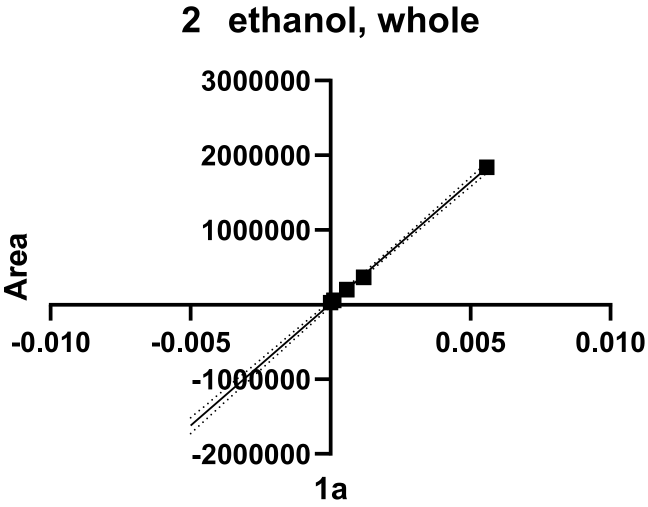

Best-fit values

Slope	326357823
Y-intercept	11047
X-intercept	-3.385e-005
1/slope	3.064e-009

Std. Error

Slope	4839323
Y-intercept	12427

95% Confidence Intervals

Slope	310956936 to 341758709
Y-intercept	-28500 to 50593
X-intercept	-0.0001596 to 8.504e-005

Goodness of Fit

R squared	0.9993
Sy.x	22598

Entry 3

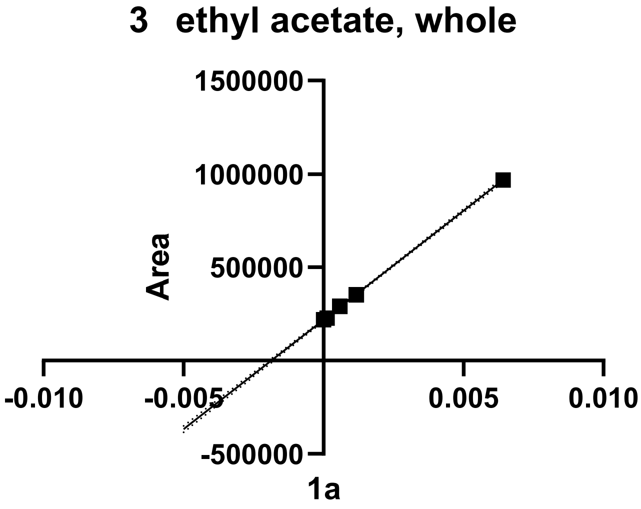

Best-fit values

Slope	116974358
Y-intercept	217827
X-intercept	-0.001862
1/slope	8.549e-009

Std. Error

Slope	756494
Y-intercept	2215

95% Confidence Intervals

Slope	114566858 to 119381858
Y-intercept	210777 to 224877
X-intercept	-0.001952 to -0.001776

Goodness of Fit

R squared	0.9999
Sy.x	4081

Entry 4

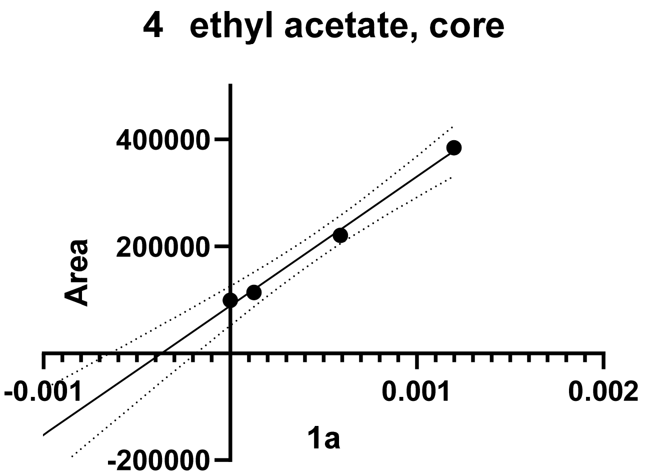

Best-fit values

Slope	241347105
Y-intercept	88502
X-intercept	-0.0003667
1/slope	4.143e-009

Std. Error

Slope	12746879
Y-intercept	8563

95% Confidence Intervals

Slope	186501712 to 296192498
Y-intercept	51657 to 125347
X-intercept	-0.0006434 to -0.0001822

Goodness of Fit

R squared	0.9945
Sy.x	11996

Entry 5

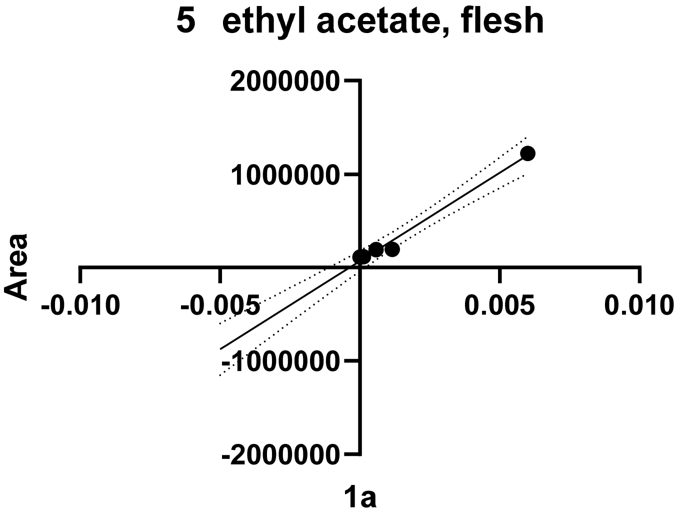

Best-fit values

Slope	189021061
Y-intercept	69192
X-intercept	-0.0003661
1/slope	5.290e-009

Std. Error

Slope	12544116
Y-intercept	34455

95% Confidence Intervals

Slope	149100085 to 228942037
Y-intercept	-40458 to 178842
X-intercept	-0.001105 to 0.0001918

Goodness of Fit

R squared	0.9870
Sy.x	63130

Entry 6

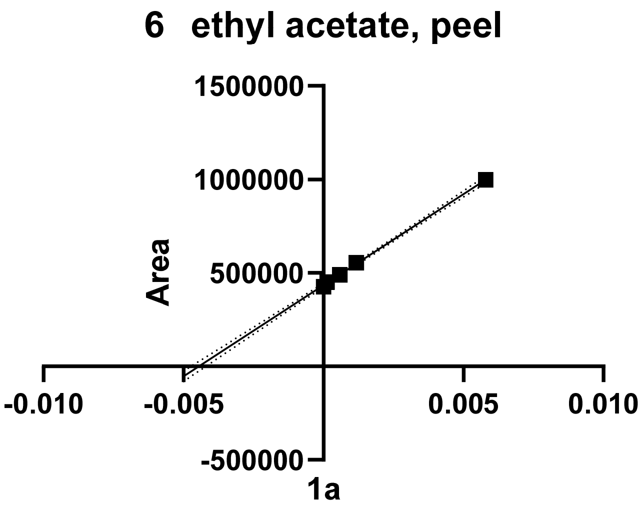

Best-fit values	
Slope	97444804
Y-intercept	434255
X-intercept	-0.004456
1/slope	1.026e-008
Std. Error	
Slope	1270590
Y-intercept	3376
95% Confidence Intervals	
Slope	93401220 to 101488388
Y-intercept	423510 to 445000
X-intercept	-0.004732 to -0.004202
Goodness of Fit	
R squared	0.9995
Sy.x	6161

6 Structure-Activity Relationship of Isofalcarintriol

Synthesis of S20

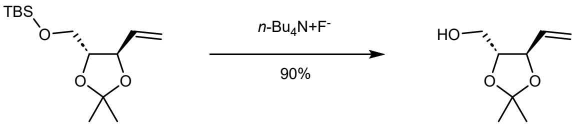

7 **S20**

To a solution of **7** (3.0 g, 9.5 mmol, 1.0 equiv) in THF (0.20 M) at rt and n-$Bu_4N^+F^-$ (22 ml, 1.0 M in THF, 22 mmol, 2.3 equiv) was slowly added. After complete consumption of the starting material, the reaction was stopped by addition of sat. aq. NH_4Cl solution. The crude reaction mixture was extracted with diethyl ether. The combined organic layers were dried over Na_2SO_4, filtered, and concentrated under reduced pressure. The crude mixture was purified by flash column chromatography (pentane–diethyl ether, 2:1) resulting in title compound as pale-yellow oil (1.3 g, 90% yield).

^{1}H NMR (400 MHz, $CDCl_3$) δ = 5.83 (ddd, J = 17.1, 10.3, 7.3 Hz, 1H), 5.38 (ddd, J = 17.1, 1.4, 1.0 Hz, 1H), 5.26 (ddd, J = 10.3, 1.4, 0.8 Hz, 1H), 4.31 (ddt, J = 8.3, 7.3, 0.9 Hz, 1H), 3.87 – 3.75 (m, 2H), 3.65 – 3.55 (m, 1H), 2.08 (s, 1H), 1.43 (s, 6H)
^{13}C NMR (101 MHz, $CDCl_3$) δ = 135.2, 119.4, 109.4, 81.1, 78.5, 60.9, 27.2, 27.1
IR (neat, ν_{max}/cm^{-1}) 2995, 1770, 1759, 1375, 1246, 1057
ESI-MS calcd for $C_8H_{14}NaO_3$ [M+Na]$^+$ 181.0535; found 181.0834
[α]$^{25}_D$ = 3.2 (c = 1.0, $CHCl_3$)

Synthesis of S13

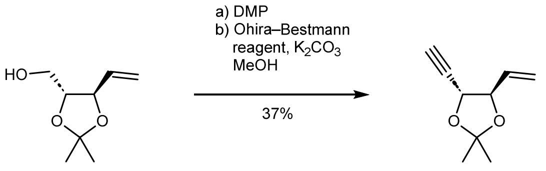

S20 **S13**

To a solution of **S20** (0.50 g, 2.8 mmol, 1.0 equiv) in CH_2Cl_2 (0.10 M) was added DESS–MARTIN periodinane (1.5 g, 3.3 mmol, 1.2 equiv) at room temperature. After 2 h, the reaction was stopped by addition of sat. aq. $Na_2S_2O_3$ solution and sat. aq. $NaHCO_3$ solution. The suspension was stirred vigorously for 10 min and extracted with diethyl ether. The combined organic layers were washed with water, dried over $MgSO_4$, filtered and concentrated under reduced pressure.
OHIRA–BESTMANN reagent (1.0 g, 5.5 mmol, 2.0 equiv) and K_2CO_3 (1.0 g, 7.4 mmol, 2.7 equiv) were suspended in MeOH at 0 °C. After 60 min the crude product dissolved in MeOH was slowly added resulting in a 50 mM solution. The reaction was allowed to reach rt and stirred for 90 min. The reaction was stopped by addition of brine and the crude reaction mixture was extracted with pentane. The combined organic layers were dried over $MgSO_4$, filtered and concentrated under reduced pressure. The crude mixture was purified by flash column chromatography (pentane–diethyl ether, 20:1) resulting in title compound as color-less oil (0.16 g, 37% yield).

^{1}H NMR (500 MHz, $CDCl_3$) δ = 5.84 (ddd, J = 17.2, 10.4, 6.9 Hz, 1H), 5.48 (dt, J = 17.2, 1.2 Hz, 1H), 5.32 (dt, J = 10.4, 1.1 Hz, 1H), 4.43 (ddt, J = 7.9, 6.9, 1.0 Hz, 1H), 4.28 (dd, J = 8.0, 2.1 Hz, 1H), 2.53 (d, J = 2.1 Hz, 1H), 1.49 (s, 3H), 1.44 (s, 3H)
^{13}C NMR (126 MHz, $CDCl_3$) δ = 133.5, 119.8, 110.7, 82.9, 79.8, 75.1, 70.5, 27.0, 26.5
IR (neat, ν_{max}/cm^{-1}) 3297, 2990, 2937, 2885, 1374, 1239, 1171, 1052, 870, 666, 641
EI-MS calcd for C_8H_9O2 [M–CH_3] 137.05971; found 137.05957
[α]$^{26}_D$ = 4.0 (c = 1.0, $CHCl_3$)

Synthesis of S14

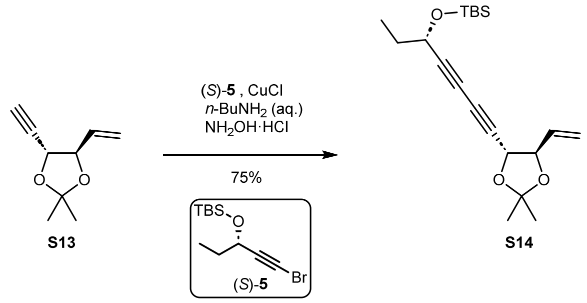

To a solution of **S13** (74 mg, 0.48 mmol, 1.0 equiv) in diethyl ether (70 mM) at room temperature was added a solution of copper(I) chloride (5.0 mol%) in *n*-BuNH$_2$ (4.0 ml, 30% in water, 12 mmol, 25 equiv) resulting in a faint blue solution. After addition the reaction was cooled to 0 °C. A few crystals of hydroxylamine hydrochloride were added to discharge the blue color (indication of other than copper(I) species). A solution of (*S*)-**5** (0.15 g, 0.54 mmol, 1.1 equiv) in diethyl ether (70 mM) was added and the reaction was allowed to reach room temperature. After 60 min the reaction was stopped, diluted with water, and extracted with diethyl ether. The combined organic layers were dried over MgSO$_4$, filtered, and concentrated under reduced pressure. The crude mixture was purified by flash column chromatography (pentane–diethyl ether, 40:1) in title compound as pale-yellow oil (0.13 g, 75% yield).

^{1}H NMR (400 MHz, CDCl$_3$) δ = 5.83 (ddd, J = 17.1, 10.4, 6.7 Hz, 1H), 5.49 (dt, J = 17.1, 1.2 Hz, 1H), 5.32 (dt, J = 10.4, 1.1 Hz, 1H), 4.50 – 4.40 (m, 1H), 4.39 – 4.28 (m, 2H), 1.69 (td, J = 7.4, 6.2 Hz, 2H), 1.50 – 1.48 (s, 3H), 1.44 (s, 3H), 0.97 (t, J = 7.4 Hz, 3H), 0.90 (s, 9H), 0.13 (s, 3H), 0.10 (s, 3H)
^{13}C NMR (101 MHz, CDCl$_3$) δ = 133.5, 119.9, 110.9, 82.6, 82.0, 74.4, 71.2, 71.1, 68.0, 64.6, 31.7, 27.0, 26.4, 25.9, 18.4, 9.6, -4.5, -5.0
IR (neat, v_{max}/cm^{-1}) 2988, 2957, 2931, 2858, 1464, 1381, 1374, 1251, 1064, 1045, 837, 778
ESI-MS calcd for $C_{20}H_{32}NaO_3Si$ [M+Na]$^+$ 371.2013; found 371.2014
[α]$^{26}_D$ = -15.1 (c = 1.0, CHCl$_3$)

Synthesis of 17

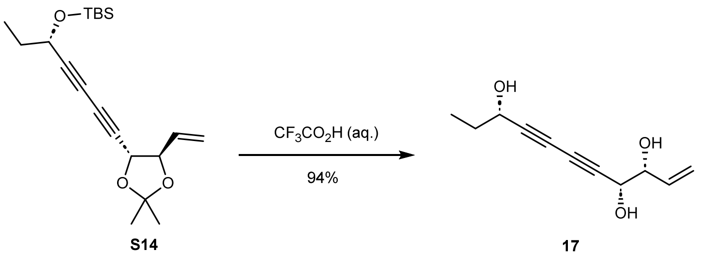

To a solution of **S14** (20 mg, 57 µmol, 1.0 equiv) in THF–water (4:1, 20 mM) at room temperature was added CF_3CO_2H (0.13 ml, 1.7 mmol, 30 equiv). The reaction was caped and stirred at 60 °C. After complete consumption of the starting material, the reaction was stopped, diluted with sat. aq. NH4Cl solution. The crude reaction was extracted with ethyl acetate, the combined organic layers were dried over Na_2SO_4, filtered and concentrated under reduced pressure. The crude mixture was purified by flash column chromatography (hexanes–ethyl acetate 1:1) resulting in title compound as yellow oil (10 mg, 94% yield).

^{1}H NMR (400 MHz, $CDCl_3$) δ = 5.94 (ddd, J = 17.2, 10.6, 5.7 Hz, 1H), 5.47 (dt, J = 17.2, 1.4 Hz, 1H), 5.34 (dt, J = 10.6, 1.4 Hz, 1H), 4.39 (td, J = 6.5, 0.8 Hz, 1H), 4.30 (dd, J = 6.4, 0.8 Hz, 1H), 4.20 (ddt, J = 6.3, 5.7, 1.4 Hz, 1H), 2.44 (s, 2H), 1.83 – 1.68 (m, 2H), 1.02 (t, J = 7.4 Hz, 3H)
^{13}C NMR (101 MHz, $CDCl_3$) δ = 135.1, 118.8, 80.9, 77.1, 75.6, 70.8, 68.9, 66.6, 64.2, 30.7, 9.4
IR (neat, ν_{max}/cm^{-1}) 3347, 2995, 1770, 1759, 1382, 1246, 1056
ESI-MS calcd for $C_{11}H_{14}NaO_3$ $[M+Na]^+$ 217.0837, found 217.0835
$[\alpha]^{25}_D$ = 26.5 (c = 0.5, $CHCl_3$)

Synthesis of 19

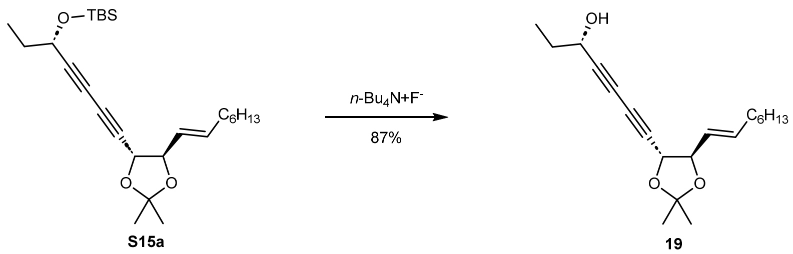

To a solution of **S15a** (96 mg, 0.22 mmol, 1.0 equiv) in THF (0.10 M) at 0 °C n-$Bu_4N^+F^-$ (0.44 ml, 1.0 M in THF, 0.44 mmol, 2.0 equiv) was slowly added. After complete consumption of the starting material, the reaction was stopped by addition of sat. aq. NH_4Cl solution. The crude reaction mixture was extracted with diethyl ether. The combined organic layers were dried over Na_2SO_4, filtered, and concentrated under reduced pressure. The crude mixture was purified by flash column chromatography (hexanes–ethyl acetate, 2:1) resulting in title compound as pale-yellow oil (61 mg, 87% yield).

^{1}H NMR (500 MHz, $CDCl_3$) δ = 5.91 (dtt, J = 15.3, 6.7, 0.9 Hz, 1H), 5.40 (ddt, J = 15.3, 7.7, 1.5 Hz, 1H), 4.44 – 4.34 (m, 2H), 4.31 (dd, *J* = 7.8, 0.7 Hz, 1H), 2.11 – 2.03 (m, 2H), 1.89 (d, J = 4.5 Hz, 1H), 1.75 (qdd, J = 7.3, 6.3, 2.5 Hz, 2H), 1.46 (s, 3H), 1.42 (s, 3H), 1.41 – 1.37 (m, 2H), 1.34 – 1.23 (m, 6H), 1.01 (t, J = 7.4 Hz, 3H), 0.94 – 0.84 (m, 3H)
^{13}C NMR (126 MHz, $CDCl_3$) δ = 138.1, 124.7, 110.5, 82.6, 80.6, 75.5, 71.0, 70.4, 68.9, 64.1, 32.3, 31.7, 30.6, 28.8, 28.7, 27.0, 26.1, 22.6, 14.1, 9.3
IR (neat, ν_{max}/cm^{-1}) 3435, 2960, 2928, 2857, 1457, 1380, 1235, 1049, 967, 877
ESI-MS calcd for $C_{20}H_{30}NaO_3$ $[M+Na]^+$ 341.20872; found 341.20821
$[\alpha]^{24}_D$ = 106.0 (c = 1.0, $CHCl_3$)

Synthesis of S16

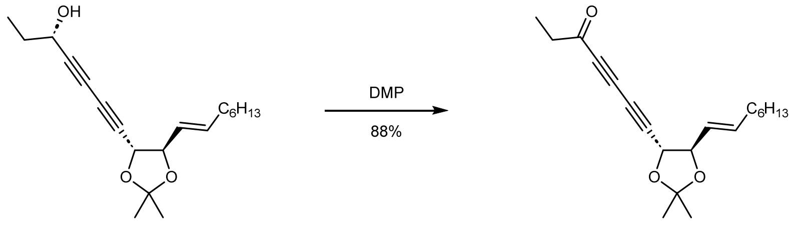

19 S16

To a solution of **19** (34 mg, 0.11 mmol, 1.0 equiv) in CH_2Cl_2 (40 mM) was added DESS–MARTIN periodinane (55 mg, 0.13 mmol, 1.2 equiv) at room temperature. After complete consumption of the starting material, the reaction was stopped by addition of sat. aq. $Na_2S_2O_3$ solution and sat. aq. $NaHCO_3$ solution. The suspension was stirred vigorously for 15 min and extracted with diethyl ether. The combined organic layers were washed with water, dried over $MgSO_4$, filtered, and concentrated under reduced pressure resulting in title compound as pale-yellow oil (30 mg, 88% yield).

^{1}H NMR (400 MHz, $CDCl_3$) δ = 5.41 (ddt, J = 15.3, 7.8, 1.5 Hz, 1H), 4.46 (td, J = 7.8, 0.8 Hz, 1H), 2.61 (q, J = 7.3 Hz, 2H), 2.13 – 2.03 (m, 2H), 1.47 (s, 3H), 1.44 (s, 3H), 1.42 – 1.35 (m, 2H), 1.33 – 1.21 (m, 6H), 1.15 (t, J = 7.4 Hz, 3H), 0.92 – 0.84 (m, 3H)
^{13}C NMR (101 MHz, $CDCl_3$) δ = 187.3, 138.7, 124.5, 124.5, 111.1, 83.7, 82.7, 75.5, 74.1, 70.9, 69.4, 39.0, 32.4, 31.8, 28.9, 28.8, 27.0, 26.1, 22.7, 14.2, 7.9
IR (neat, v_{max}/cm^{-1}) 2987, 2957, 2928, 2857, 2237, 2148, 1678, 1381, 1229, 1106, 1052, 967, 877
ESI-MS calcd for $C_{20}H_{28}NaO_3$ $[M+Na]^+$ 339.19307; found 339.19307
$[\alpha]^{24}_D$ = 94.9 (c = 1.0, $CHCl_3$)

Synthesis of 18

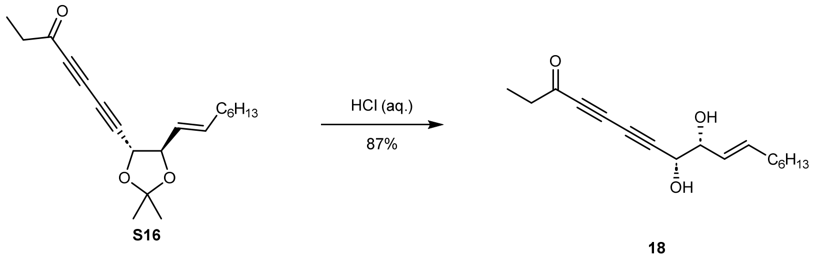

To a solution of **S16** (5.6 mg, 18 mmol, 1.0 equiv) in MeOH (20 mM) at room temperature was added HCl (0.44 ml, 2.0 M in water, 0.89 mmol, 50 equiv). After complete consumption of the starting material, the reaction was stopped and quenched with aq. $NaHCO_3$ solution. The crude reaction was extracted with diethyl ether, the combined organic layers were dried over $MgSO_4$, filtered, and concentrated under reduced pressure. The crude mixture was purified by flash column chromatography (hexanes–ethyl acetate 1:1) resulting in title compound as pale-yellow oil (4.2 mg, 86% yield).

^{1}H NMR (400 MHz, $CDCl_3$) δ = 5.88 (dtd, J = 15.1, 6.8, 1.0 Hz, 1H), 5.50 (ddt, J = 15.4, 7.1, 1.5 Hz, 1H), 4.34 (t, J = 5.8 Hz, 1H), 4.17 (t, J = 6.8 Hz, 1H), 2.60 (q, J = 7.3 Hz, 2H), 2.54 (d, J = 6.1 Hz, 1H), 2.29 – 2.24 (m, 1H), 2.08 (td, J = 7.6, 6.0 Hz, 2H), 1.51 – 1.37 (m, 4H), 1.37 – 1.24 (m, 6H), 1.15 (t, J = 7.3 Hz, 3H), 0.89 (td, J = 6.9, 3.8 Hz, 3H)
^{13}C NMR (101 MHz, $CDCl_3$) δ = 187.4, 137.3, 126.4, 85.4, 75.5, 75.4, 74.2, 69.3, 66.8, 39.0, 32.5, 31.8, 29.0, 29.0, 22.78 14.2, 8.0
IR (neat, v_{max}/cm^{-1}) 3365, 2957, 2926, 2856, 2234, 2143, 1676, 1459, 1100, 1042, 970
ESI-MS calcd for $C_{17}H_{24}NaO_3$ $[M+Na]^+$ 299.1618; found 299.1614
$[\alpha]^{24}_D$ = 39.9 (c = 0.5, $CHCl_3$)

Synthesis of S17
The enantiomer of S17 has previously been synthesized on a different route.[32]

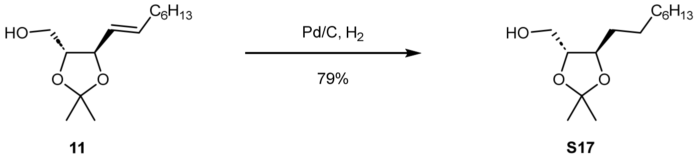

To a solution of **11** (31 mg, 0.13 mmol, 1.0 equiv) in hexane (13 mM) at room temperature was added palladium on carbon (10% wt., 14 mg, 13 mmol, 10 mol%) and the reaction was stirred under an atmosphere of hydrogen. After complete consumption of the starting material, the reaction was stopped. The crude reaction was diluted with CH_2Cl_2, filtered over celite, and concentrated under reduced pressure resulting in title compound as pale-yellow oil (25 mg, 79% yield).

^{1}H NMR (400 MHz, $CDCl_3$) δ = 3.87 (ddd, J = 8.2, 7.4, 4.5 Hz, 1H), 3.79 (dd, J = 11.8, 3.0 Hz, 1H), 3.72 (ddd, J = 8.3, 4.5, 3.0 Hz, 1H), 3.58 (dd, J = 11.9, 4.4 Hz, 1H), 2.10 – 1.96 (m, 1H), 1.68 – 1.17 (m, 20H), 0.96 – 0.81 (m, 3H)
^{13}C NMR (101 MHz, $CDCl_3$) δ = 108.7, 81.7, 77.1, 62.2, 33.3, 32.0, 29.8, 29.6, 29.4, 27.5, 27.2, 26.1, 22.8, 14.2
IR (neat, v_{max}/cm^{-1}) 3457, 2927, 2861, 1456, 1376, 1233, 1055, 857
ESI-MS calcd for $C_{14}H_{28}NaO_3$ $[M+Na]^+$ 267.1931; found 267.1934
$[\alpha]^{25}_D$ = 22.3 (c = 1.0, $CHCl_3$)

Synthesis of S18

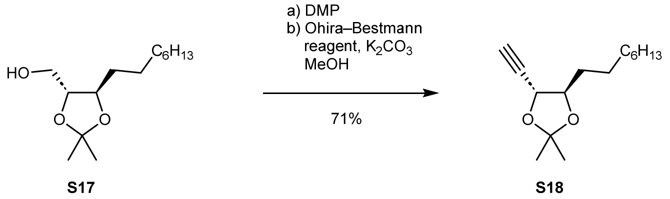

The corresponding compound was prepared from **S17** (25 mg, 0.10 mmol, 1.0 equiv) following **general procedure C** using DESS–MARTIN periodinane (1.5 equiv), OHIRA–BESTMANN reagent (2.0 equiv) and K_2CO_3 (2.7 equiv). The reactions were stirred for 85 min and 60 min respectively resulting in title compound as pale-yellow oil (17 mg, 71% yield).

^{1}H NMR (400 MHz, $CDCl_3$) δ = 4.19 (dd, J = 7.8, 2.1 Hz, 1H), 4.03 (dt, J = 7.8, 6.1 Hz, 1H), 2.51 (d, J = 2.1 Hz, 1H), 1.70 – 1.57 (m, 2H), 1.53 – 1.11 (m, 18H), 0.96 – 0.82 (m, 3H)
^{13}C NMR (101 MHz, $CDCl_3$) δ = 110.0, 81.7, 81.1, 74.6, 70.5, 32.6, 32.0, 29.7, 29.6, 29.4, 27.3, 26.3, 25.8, 22.8, 14.2
IR (neat, v_{max}/cm^{-1}) 3313, 2989, 2926, 2856, 1458, 1381, 1371, 1239, 1057, 876
ESI-MS calcd for $C_{15}H_{26}NaO_2$ $[M+Na]^+$ 261.1825; found 261.1826
$[\alpha]^{23}_D$ = –34.4 (c = 2.0, $CHCl_3$)

Synthesis of S19

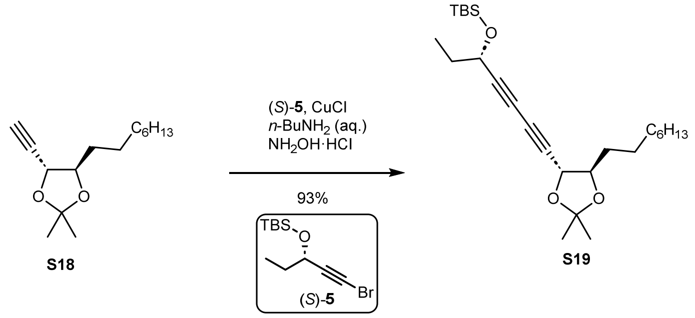

The corresponding compound was prepared from **S18** (13 mg, 53 mmol, 1.0 equiv) and (*S*)-**5** (22 mg, 79 mmol, 1.5 equiv) following **general procedure G**. After 45 min additional (*S*)-**5** (0.30 equiv) was added. The reaction was stirred 60 min resulting in title compound as pale-yellow oil (21.4 mg, 93% yield).

^{1}H NMR (400 MHz, $CDCl_3$) δ = 4.34 (td, J = 6.3, 0.7 Hz, 1H), 4.26 (dd, J = 7.7, 0.8 Hz, 1H), 4.04 (dt, J = 7.6, 6.1 Hz, 1H), 1.70 (td, J = 7.4, 6.3 Hz, 2H), 1.65 – 1.59 (m, 2H), 1.48 – 1.39 (m, 8H), 1.38 – 1.23 (m, 10H), 0.97 (t, J = 7.4 Hz, 3H), 0.90 (s, 12H), 0.13 (s, 3H), 0.10 (s, 3H)
^{13}C NMR (101 MHz, $CDCl_3$) δ = 110.2, 81.9, 81.6, 75.6, 71.0, 70.8, 68.2, 64.7, 32.6, 32.0, 31.7, 29.7, 29.6, 29.4, 27.3, 26.2, 25.9, 25.8, 22.8, 18.4, 14.3, 9.6, -4.4, -5.0
IR (neat, v_{max}/cm^{-1}) 2929, 2857, 1464, 1380, 1371, 1252, 1108, 1064, 1005, 837, 778
ESI-MS calcd for $C_{26}H_{46}NaO_3Si$ $[M+Na]^+$ 457.3105; found 457.3105
$[\alpha]^{25}_D$ = –20.7 (c = 1.0, $CHCl_3$)

Synthesis of 20

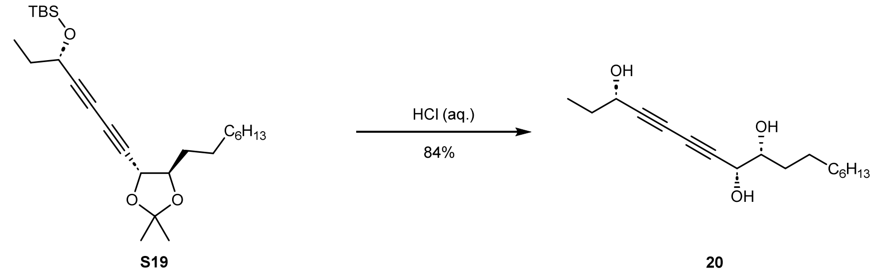

The corresponding compound was prepared from **S19** (21 mg, 49 mmol, 1.0 equiv) following **general procedure I**. The reaction was stirred 22 h resulting in title compound as pale-yellow oil (12 mg, 41 mmol, 84% yield).

^{1}H NMR (500 MHz, $CDCl_3$) δ = 4.42 – 4.35 (m, 1H), 4.23 (m, 1H), 3.66 (m, 1H), 2.56 (s, 1H), 2.39 (s, 1H), 2.01 (s, 1H), 1.76 (tdt, J = 7.5, 6.3, 3.7 Hz, 2H), 1.65 (dddd, J = 13.3, 7.7, 5.9, 2.5 Hz, 2H), 1.58 – 1.45 (m, 2H), 1.45 – 1.14 (m, 10H), 1.02 (t, J = 7.4 Hz, 3H), 0.94 – 0.80 (m, 3H)
^{13}C NMR (126 MHz, $CDCl_3$) δ = 80.6, 77.8, 74.7, 70.2, 68.78 66.6, 64.1, 32.5, 31.9, 30.6, 29.5, 29.5, 29.3, 25.5, 22.7, 14.1, 9.3
IR (neat, v_{max}/cm^{-1}) 3350, 2925, 2856, 1463, 1337, 1091, 1048, 1017, 968
ESI-MS calcd for $C_{17}H_{28}NaO_3$ $[M+Na]^+$ 303.1931; found 303.1934
$[\alpha]^{26}_D$ = 13.2 (c = 0.5, $CHCl_3$)

7 *Isofalcarintriol-Derived Functional Probes*

7.1 Synthesis of Biotin Labeled Isofalcarintriol

Biotin ethyl ester (**21**) was prepared from D-Biotin according to GOSWAMI *et al.*[33]

Synthesis of S12

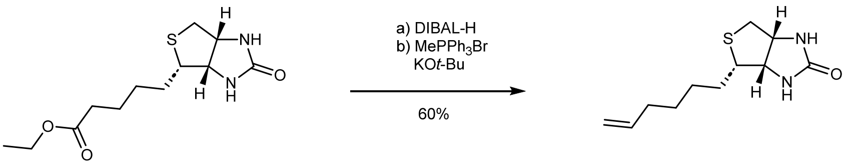

21 **S12**

To a solution of **21** (3.0 g, 11 mmol, 1.0 equiv) in CH_2Cl_2 (0.15 M) was added DIBAL-H (23 ml, 1.0 M in CH_2Cl_2, 23 mmol, 2.1 equiv) at –78 °C and the reaction was stirred for 2 h. The reaction was quenched with ethyl acetate and allowed to reach rt. Sat. aq. potassium sodium tartrate solution was added and the reaction was stirred for 30 min. The crude reaction mixture was extracted with CH_2Cl_2. The combined organic layers were dried over $MgSO_4$, filtered, and concentrated under reduced pressure. The crude aldehyde was used for the next step without further purification.
To a solution of $MePPh_3Br$ (4.7 g, 13 mmol, 1.2 equiv) in THF (0.10 M) was added KO*t*-Bu (10 ml, 1.0 M in THF, 10 mmol, 1.2 equiv) at rt. The reaction was stirred for 2.5 h and crude aldehyde was added over 10 min and stirring was continued for 20 min. The reaction was allowed to reach rt and after 80 min the reaction was quenched with water. The crude reaction mixture was extracted with CH_2Cl_2. The combined organic layers were dried over $MgSO_4$, filtered, and concentrated under reduced pressure. The crude mixture was purified by flash column chromatography (CH_2Cl_2–MeOH, 10:1) resulting in title compound as white crystals (1.5 g, 60% yield).

^{1}H NMR (400 MHz, $CDCl_3$) δ = 5.79 (ddt, J = 16.9, 10.2, 6.7 Hz, 1H), 5.41 (d, J = 13.8 Hz, 2H), 4.50 (ddt, J = 7.6, 5.0, 1.2 Hz, 1H), 4.30 (ddd, J = 7.8, 4.5, 1.6 Hz, 1H), 3.16 (ddd, J = 8.3, 6.6, 4.5 Hz, 1H), 2.92 (dd, J = 12.8, 5.1 Hz, 1H), 2.77 – 2.69 (m, 1H), 2.06 (qd, J = 5.8, 2.4 Hz, 2H), 1.76 – 1.58 (m, 2H), 1.51 – 1.33 (m, 4H)
^{13}C NMR (101 MHz, $CDCl_3$) δ = 163.5, 138.78, 114.8, 62.1, 60.3, 55.7, 40.7, 33.6, 28.8, 28.6
IR (neat, v_{max}/cm^{-1}) 3209, 3119, 3074, 2926, 1699, 1463, 910
ESI-MS calcd for $C_{11}H_{18}N_2NaOS$ $[M+Na]^+$ 249.1032; found 249.1036
$[\alpha]^{26}_D$ = 35.6 (c = 1.0, $CHCl_3$)
MP = 155–156 °C

Synthesis of 22

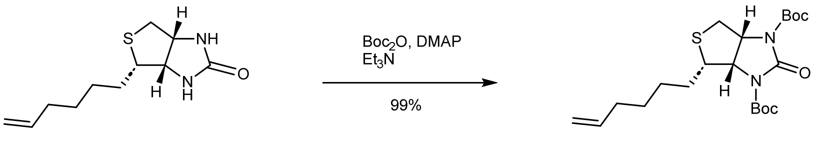

S12 **22**

To a solution of **S12** (0.55 g, 2.4 mmol, 1.0 equiv) in CH_2Cl_2 (0.25 M) was added Boc_2O (2.1 g, 9.8 mmol, 4.0 equiv), Et_3N (0.75 ml, 5.4 mmol, 2.2 equiv) and DMAP (0.66 g, 5.4 mmol, 2.2 equiv). After complete consumption of the starting material, the reaction was stopped by addition of 2% aq. HCl solution. The crude reaction mixture was diluted with ethyl acetate and washed with brine. The organic layer was dried over $MgSO_4$, filtered, and concentrated under reduced pressure. The crude mixture was purified by flash column chromatography (hexane–ethyl acetate, 4:1) resulting in title compound as white crystals (1.0 g, 99% yield).

^{1}H NMR (400 MHz, $CDCl_3$) δ = 5.78 (ddt, J = 16.9, 10.2, 6.7 Hz, 1H), 5.04 – 4.90 (m, 2H), 4.63 – 4.48 (m, 2H), 3.47 (ddd, J = 11.9, 6.0, 3.3 Hz, 1H), 3.34 – 3.24 (m, 1H), 2.91 – 2.81 (m, 1H), 2.04 (m, 2H), 1.73 – 1.13 (m, 24H)
^{13}C NMR (101 MHz, $CDCl_3$) δ = 150.2, 150.1, 148.9, 138.7, 114.7, 84.0, 83.9, 60.4, 58.2, 52.5, 36.6, 33.7, 28.5, 28.2, 28.2, 27.8, 27.7
IR (neat, v_{max}/cm^{-1}) 2978, 2931, 1806, 1714, 1367, 1296, 1273, 1147
ESI-MS calcd for $C_{21}H_{34}N_2NaO_5S$ $[M+Na]^+$ 449.2081; found 449.2084
$[\alpha]^{27}_D$ = –66.1 (c = 1.0, $CHCl_3$)
MP = 141-142 °C

Synthesis of 23

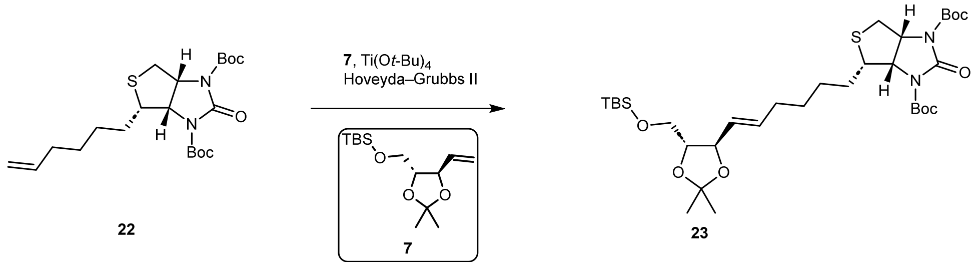

22 **23**

To a solution of **7** (0.10 g, 0.37 mmol, 1.0 equiv) in CH_2CH_2 (0.10 M) were added alkene **22** (0.31 g, 0.73 mmol, 2.0 equiv), Ti(O*t*-Bu)$_4$ (0.84 ml, 2.2 mmol, 6.0 equiv) and HOVEYDA–GRUBBS 2nd generation catalyst (23 mg, 37 μmol, 10 mol%). The reaction mixture was sparged with argon and the reaction mixture was stirred at 40 °C. After 24 h additional HOVEYDA–GRUBBS 2nd generation catalyst (12 mg, 18 μmol, 5.0 mol%) was added. The reaction was stopped after 36 h, filtered through a short silica plug and concentrated under reduced pressure. The crude mixture was purified by flash column chromatography (CH_2Cl_2–MeOH, 10:1) resulting in title compound as a yellow oil (162 mg, 66% yield) and recovered **7** (30 mg, 30% yield).

^{1}H NMR (400 MHz, $CDCl_3$) δ = 5.83 – 5.70 (m, 1H), 5.53 – 5.40 (m, 1H), 4.64 – 4.49 (m, 2H), 4.28 (q, J = 7.6 Hz, 1H), 3.82 – 3.63 (m, 3H), 3.47 (ddd, J = 12.1, 6.0, 3.2 Hz, 1H), 3.29 (dd, J = 12.8, 6.6 Hz, 1H), 2.90 – 2.81 (m, 1H), 2.05 (q, J = 7.1 Hz, 2H), 1.73 – 1.14 (m, 30H), 0.89 (s, 9H), 0.06 (s, 3H), 0.06 (s, 3H)
^{13}C NMR (101 MHz, $CDCl_3$) δ = 150.2, 150.0, 149.0, 135.8, 127.6, 108.9, 84.0, 83.9, 81.6, 79.0, 62.3, 60.4, 58.2, 52.5, 36.6, 32.4, 28.6, 28.2, 28.2, 28.0, 27.7, 27.3, 27.0, 26.1, 18.5, -5.1, -5.3
IR (neat, v_{max}/cm^{-1}) 2983, 2928, 2856, 1810, 1789, 1716, 1298, 1251, 1149, 838, 778
ESI-MS calcd for $C_{33}H_{58}N_2NaO_8SSi$ $[M+Na]^+$ 693.3575; found 693.3572
$[\alpha]^{24}_D$ = –26.1 (c = 0.5, $CHCl_3$)

Synthesis of 24

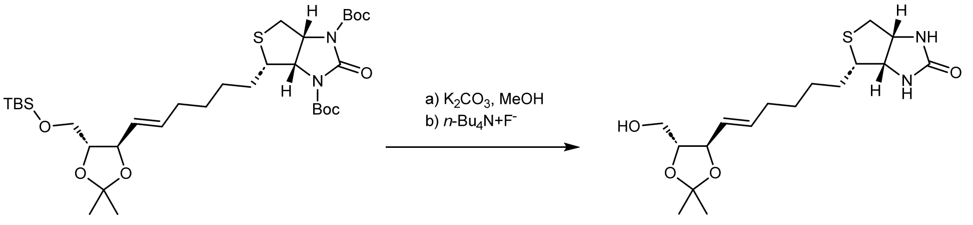

23 **24**

To a solution of **23** (71 mg, 0.11 mmol, 1.0 equiv) in MeOH (0.10 M) was added K_2CO_3 (0.37 g, 2.7 mmol, 25 equiv). The reaction was stirred at 60 °C for 24 h. The crude reaction mixture was quenched with sat. aq. NH_4Cl solution and extracted with CH_2CH_2. The combined organic layers were dried over $MgSO_4$, filtered, and concentrated under reduced pressure. The crude product was used for the next step without further purification.
The crude product was dissolved in THF (50 mM) at 0 °C and n-$Bu_4N^+F^-$ (0.11 ml, 1.0 M in THF, 0.11 mmol, 1.0 equiv) was slowly added. The reaction was allowed to reach rt. After 5 h, the reaction was stopped by addition of sat. aq. NH_4Cl solution. The crude reaction mixture was extracted with ethyl acetate. The combined organic layers were dried over Na_2SO_4, filtered, and concentrated under reduced pressure. The crude mixture was purified by flash column chromatography (CH_2Cl_2–MeOH, 10:1) resulting in title compound as pale-yellow oil (30 mg, 78% yield) over two steps.

^{1}H NMR (400 MHz, $CDCl_3$) δ = 5.91 – 5.73 (m, 1H), 5.47 (dtt, J = 15.3, 8.0, 1.4 Hz, 1H), 4.50 (ddd, J = 7.9, 5.0, 1.1 Hz, 1H), 4.27 (m, 2H), 3.86 – 3.71 (m, 2H), 3.69 – 3.56 (m, 1H), 3.14 (ddt, J = 8.7, 6.1, 4.4 Hz, 1H), 2.91 (ddd, J = 12.9, 5.0, 1.6 Hz, 1H), 2.73 (d, J = 12.8 Hz, 1H), 2.08 (q, J = 6.9 Hz, 2H), 1.77 – 1.53 (m, 2H), 1.51 – 1.39 (m, 10H)
^{13}C NMR (101 MHz, $CDCl_3$) δ = 163.8, 136.7, 127.1, 109.0, 81.3, 78.6, 62.2, 61.1, 60.3, 55.7, 40.7, 32.1, 28.7, 28.6, 28.5, 27.3, 27.1
IR (neat, v_{max}/cm^{-1}) 3259, 2924, 2855, 1696, 1248, 1216, 1050, 970, 858, 759
ESI-MS calcd for $C_{17}H_{28}N_2NaO_4S$ $[M+Na]^+$ 379.1662; found 379.1655
[α]$^{24}_D$ = 42.0 (c = 1.0, $CHCl_3$)

Synthesis of 25

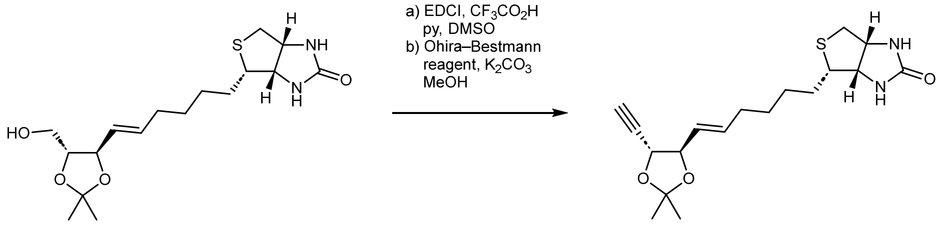

To a solution of **24** (45 mg, 0.12 mmol, 1.0 equiv) in DMSO (0.10 M) was added CF_3CO_2H (7.0 µl, 92 µmol, 0.75 equiv), pyridine (52 µl, 0.64 mmol, 5.3 equiv) and EDCI (0.11 g, 0.55 mmol, 4.5 equiv). After 3 h, the reaction was stopped by addition of sat. aq. NH_4Cl solution and extracted with CH_2Cl_2. The combined organic layers were washed with water, dried over Na_2SO_4, filtered, and concentrated under reduced pressure.

The crude product was dissolved in MeOH (20 mM) at 0 °C, OHIRA–BESTMANN reagent (0.11 g, 0.57 mmol, 4.7 equiv) and K_2CO_3 (0.11 g, 0.77 mmol, 6.3 equiv) were added. After 15 min, the reaction was allowed to reach rt and stirred for 60 min. The reaction was stopped by addition of sat. aq. NH_4Cl solution and the crude reaction mixture was extracted with ethyl acetate. The combined organic layers were dried over $MgSO_4$, filtered, and concentrated under reduced pressure. The crude mixture was purified by flash column chromatography (CH_2Cl_2–MeOH, 10:1) resulting in title compound as color-less oil (25 mg, 59% yield) over two steps.

^{1}H NMR (400 MHz, $CDCl_3$) δ = 5.90 (dtd, J = 15.3, 6.7, 0.8 Hz, 1H), 5.56 (s, 1H), 5.51 – 5.32 (m, 2H), 4.54 – 4.46 (m, 1H), 4.38 (td, J = 7.9, 0.8 Hz, 1H), 4.33 – 4.17 (m, 2H), 3.15 (ddd, J = 8.6, 6.2, 4.5 Hz, 1H), 2.91 (dd, J = 12.9, 5.0 Hz, 1H), 2.73 (d, J = 12.8 Hz, 1H), 2.53 (d, J = 2.1 Hz, 1H), 2.19 – 2.05 (m, 2H), 1.74 – 1.58 (m, 2H), 1.55 – 1.38 (m, 10H)
^{13}C NMR (101 MHz, $CDCl_3$) δ = 163.6, 137.3, 125.4, 110.3, 82.8, 80.0, 75.0, 70.5, 62.2, 60.3, 55.6, 40.7, 32.2, 28.7, 28.7, 28.6, 27.1, 26.4
IR (neat, v_{max}/cm^{-1}) 3215, 2925, 1698, 1380, 1238, 1053, 874, 665
ESI-MS calcd for $C_{18}H_{26}N_2NaO_3S$ $[M+Na]^+$ 373.1556 found 373.1556
$[\alpha]^{24}_D$ = 53.4 (c = 1.0, $CHCl_3$)

Synthesis of 26

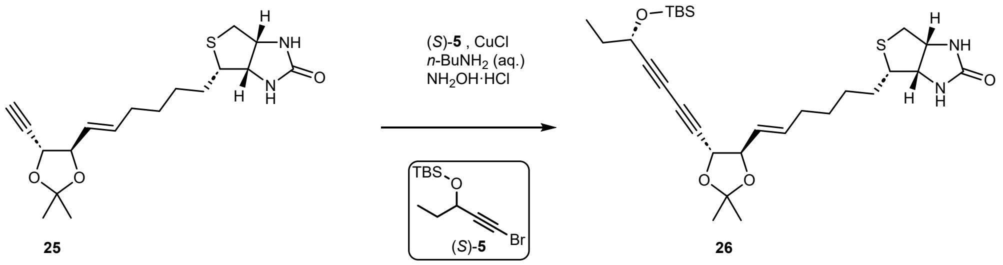

To a solution of **25** (17 mg, 50 µmol, 1.0 equiv) in diethyl ether (20 mM) at room temperature was added a solution of copper(I) chloride (50 mol%) in *n*-$BuNH_2$ (4.1 ml, 30% in water, 12 mmol, 250 equiv) resulting in a faint blue solution. After addition the reaction was cooled to 0 °C. A few crystals of hydroxylamine hydrochloride were added to discharge the blue color (indication of other than copper(I) species). A solution of (*S*)-**5** (22 mg, 74 µmol, 1.5 equiv) in diethyl ether (20 mM) was added and the reaction was allowed to reach room temperature. After 2 h the reaction was stopped, diluted with sat. aq. NH_4Cl solution and extracted with CH_2Cl_2. The combined organic layers were dried over $MgSO_4$, filtered, and concentrated under reduced pressure. The crude mixture was purified by flash column chromatography (CH_2Cl_2–MeOH, 10:1) in title compound as yellow oil 23 mg, 82% yield).

^{1}H NMR (400 MHz, $CDCl_3$) δ = 5.90 (dtd, J = 15.4, 6.7, 0.8 Hz, 1H), 5.48 – 5.37 (m, 2H), 5.33 (s, 1H), 4.50 (ddt, J = 7.7, 5.1, 1.2 Hz, 1H), 4.40 (td, J = 7.6, 0.8 Hz, 1H), 4.35 – 4.26 (m, 3H), 3.15 (ddd, J = 8.5, 6.3, 4.5 Hz, 1H), 2.91 (dd, J = 12.8, 5.0 Hz, 1H), 2.73 (d, J = 12.8 Hz, 1H), 2.17 – 2.05 (m, 2H), 1.74 – 1.59 (m, 4H), 1.54 – 1.37 (m, 10H), 0.96 (td, J = 7.4, 1.3 Hz, 3H), 0.92 – 0.82 (m, 12H), 0.12 (s, 3H), 0.09 (s, 3H)
^{13}C NMR (101 MHz, $CDCl_3$) δ = 163.5, 137.3, 125.3, 110.5, 82.6, 81.9, 74.7, 71.1, 71.0, 68.1, 64.6, 62.2, 60.3, 55.6, 40.7, 32.1, 31.7, 28.7, 28.7, 28.6, 27.1, 26.3, 25.9, 18.3, 9.6, -4.5, -5.0
IR (neat, v_{max}/cm^{-1}) 3214, 2928, 2856, 1697, 1463, 1252, 1050, 873, 778
ESI-MS calcd for $C_{29}H_{46}N_2NaO_4SSi$ $[M+Na]^+$ 569.2840; found 569.2838
$[\alpha]^{25}_D$ = –45.2(c = 1.0, $CHCl_3$)

Synthesis of 15

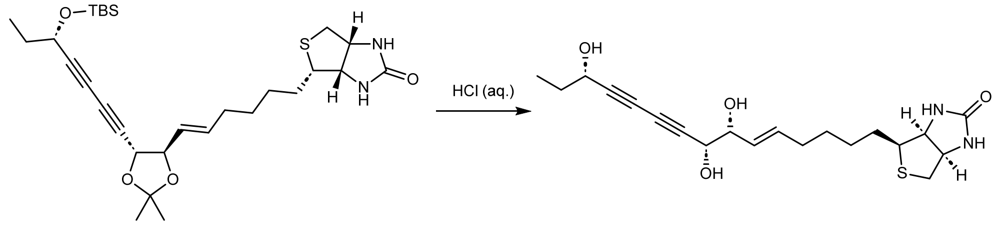

26 15

To a solution of **26** (21 mg, 39 mmol, 1.0 equiv) in MeOH (10 mM) at room temperature was added HCl (0.98 ml, 2.0 M in water, 1.9 mmol, 50 equiv). After complete consumption of the starting material, the reaction was stopped and quenched with aq. $NaHCO_3$ solution. The crude reaction was extracted with CH_2Cl_2, the combined organic layers were dried over $MgSO_4$, filtered, and concentrated under reduced pressure. The crude mixture was purified by flash column chromatography (CH_2Cl_2–MeOH, 10:1) resulting in title compound as pale-yellow wax (14 mg, 92% yield).

^{1}H NMR (500 MHz, CD_3OD) δ = 5.79 (dtd, J = 15.0, 6.8, 1.1 Hz, 1H), 5.60 – 5.46 (m, 1H), 4.49 (ddd, J = 8.0, 5.0, 0.9 Hz, 1H), 4.37 – 4.27 (m, 2H), 4.23 – 4.17 (m, 1H), 3.98 (td, J = 6.8, 1.1 Hz, 1H), 3.29 – 3.17 (m, 1H), 2.93 (dd, J = 12.8, 5.0 Hz, 1H), 2.71 (d, J = 12.7 Hz, 1H), 2.14 – 2.10 (m, 2H), 1.88 – 1.44 (m, 8H), 1.06 – 0.85 (m, 3H)
^{13}C NMR (126 MHz, CD_3OD) δ = 166.2, 135.6, 129.5, 81.6, 79.3, 76.6, 70.6, 69.1, 67.6, 64.3, 63.4, 61.6, 57.1, 41.0, 33.2, 31.8, 30.1, 29.7, 29.7, 9.8
IR (neat, v_{max}/cm^{-1}) 3295, 2927, 2855, 1685, 1432, 1430, 1331, 1268, 1094, 1019, 970, 687
ESI-MS calcd for $C_{20}H_{28}N_2NaO_3S$ $[M+Na]^+$ 415.1662; found 415.1660
$[\alpha]^{25}_D$ = 48.8 (c = 1.0, MeOH)

7.2 Synthesis of Clickable Isofalcarintriol Derivate

N-Methoxy-*N*-methylhex-5-enamide (**S21**) was prepared according to SATCHAROEN *et al.*[34]

Synthesis of S22

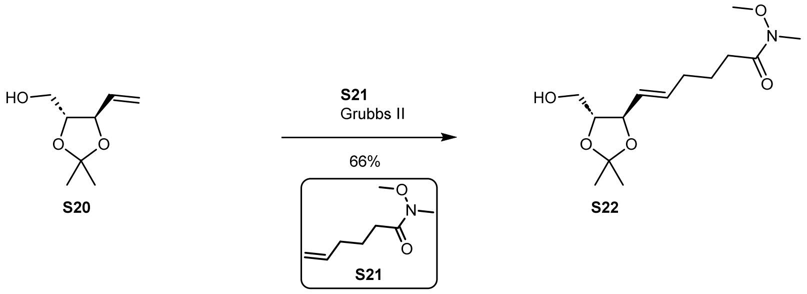

To a solution of **S20** (0.10 g, 0.67 mmol, 1.0 equiv) in CH_2CH_2 (0.20 M) were added alkene **S21** (0.26 g, 1.7 mmol, 2.5 equiv) and GRUBBS 2nd generation catalyst (28 mg, 24 µmol, 5.0 mol%). The reaction mixture was sparged with argon and the reaction mixture was stirred at 40 °C. After 66 h additional GRUBBS 2nd generation catalyst (28 mg, 24 µmol, 5.0 mol%) was added. The reaction was stopped after 88 h and dry loaded on silica. The crude mixture was purified by flash column chromatography (CH_2Cl_2–MeOH, 20:1) resulting in title compound as a pale-yellow oil (0.13 mg, 66% yield)

^{1}H NMR (400 MHz, $CDCl_3$) δ = 5.82 (dtd, J = 15.4, 6.7, 0.8 Hz, 1H), 5.54 – 5.40 (m, 1H), 4.32 – 4.23 (m, 1H), 3.86 – 3.73 (m, 2H), 3.67 (s, 3H), 3.57 (dd, J = 11.9, 3.9 Hz, 1H), 3.17 (s, 3H), 2.41 (t, J = 7.5 Hz, 2H), 2.11 (tt, J = 7.1, 1.5 Hz, 2H), 1.81 – 1.65 (m, 2H), 1.43 (s, 3H), 1.42 (s, 3H)
^{13}C NMR (101 MHz, $CDCl_3$) δ = 174.2, 136.2, 127.5 109.1, 81.2, 78.3, 61.4, 60.9, 32.3, 32.0, 31.3, 27.3, 27.1, 23.8

Synthesis of S23

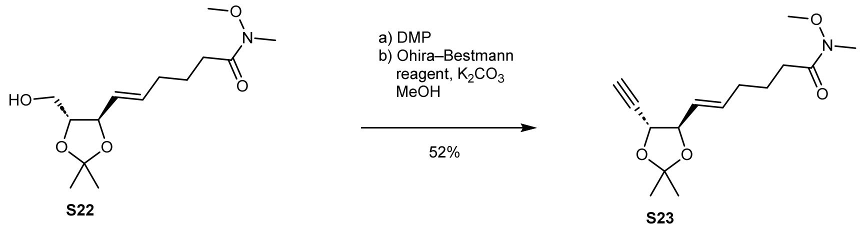

To a solution of **S22** (0.13 g, 0.46 mmol, 1.0 equiv) in CH_2Cl_2 (0.10 M) was added DESS–MARTIN periodinane (0.24 g, 0.56 mmol, 1.2 equiv) at room temperature. After 60 min, the reaction was stopped by addition of sat. aq. $Na_2S_2O_3$ solution and sat. aq. $NaHCO_3$ solution. The suspension was stirred vigorously for 10 min and extracted with diethyl ether. The combined organic layers were washed with water, dried over $MgSO_4$, filtered, and concentrated under reduced pressure.
The crude product was dissolved in MeOH (50 mM) at rt, OHIRA–BESTMANN reagent (0.18 g, 0.93 mmol, 2.0 equiv) and K_2CO_3 (0.18 g, 1.3 mmol, 2.7 equiv) were added. The reaction was stirred for 2.5 h. The reaction was stopped by addition of sat. aq. NH_4Cl solution and the crude reaction mixture was extracted with diethyl ether. The combined organic layers were dried over $MgSO_4$, filtered, and concentrated under reduced pressure. The crude mixture was purified by flash column chromatography (hexane–ethyl acetate, 2:1) resulting in title compound as color-less oil (68 mg, 52% yield) over two steps.

^{1}H NMR (400 MHz, $CDCl_3$) δ = 5.90 (dtd, J = 15.4, 6.7, 0.9 Hz, 1H), 5.46 (ddt, J = 15.4, 7.6, 1.5 Hz, 1H), 4.43 – 4.34 (m, 1H), 4.28 – 4.18 (m, 1H), 3.66 (s, 4H), 3.16 (s, 3H), 2.50 (d, J = 2.1 Hz, 1H), 2.42 (t, J = 7.5 Hz, 2H), 2.24 – 2.05 (m, 2H), 1.86 – 1.67 (m, 2H), 1.46 (s, 3H), 1.41 (s, 3H)
^{13}C NMR (101 MHz, $CDCl_3$) δ = 174.4, 136.6, 125.9, 110.3, 82.8, 80.0, 74.9, 70.6, 61.4, 32.3, 31.9, 31.2, 27.1, 26.4, 23.7
IR (neat, v_{max}/cm^{-1}) 3285, 2988, 2937, 1662, 1382, 1237, 1175, 1054, 995, 970, 878
ESI-MS calcd for $C_{15}H_{23}NNaO_4$ $[M+Na]^+$ 304.159; found 304.1519
$[\alpha]^{25}_D$ = –27.8 (c = 1.0, $CHCl_3$)

Synthesis of S24

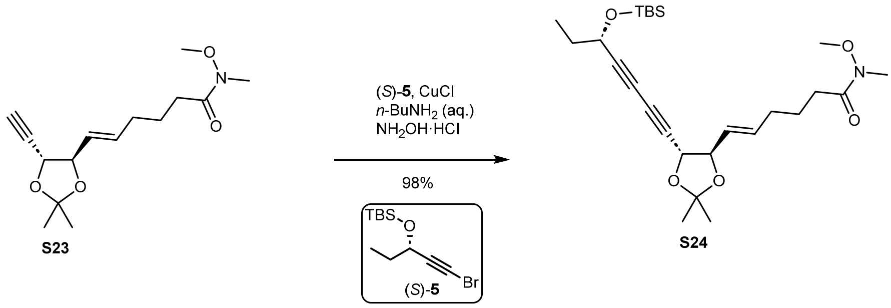

To a solution of **S23** (86 mg, 0.30 mmol, 1.0 equiv) in diethyl ether (70 mM) at room temperature was added a solution of copper(I) chloride (10 mol%) in *n*-$BuNH_2$ (2.5 ml, 30% in water, 7.7 mmol, 25 equiv) resulting in a faint blue solution. After addition the reaction was cooled to 0 °C. A few crystals of hydroxylamine hydrochloride were added to discharge the blue color (indication of other than copper(I) species). A solution of (*S*)-**5** (0.13 g, 0.46 mmol, 1.5 equiv) in diethyl ether (70 mM) was added and the reaction was allowed to reach room temperature. After 20 min, the reaction was stopped, diluted with sat. aq. NH_4Cl solution and extracted with diethyl ether. The combined organic layers were dried over $MgSO_4$, filtered, and concentrated under reduced pressure. The crude mixture was purified by flash column chromatography (hexane–diethyl ether, 1:2) in title compound as pale-yellow oil (0.14 g, 98% yield).

^{1}H NMR (500 MHz, $CDCl_3$) δ = 5.91 (dtd, J = 15.4, 6.7, 0.8 Hz, 1H), 5.46 (ddt, J = 15.4, 7.6, 1.5 Hz, 1H), 4.41 (td, J = 7.7, 0.9 Hz, 1H), 4.36 – 4.28 (m, 2H), 3.67 (s, 3H), 3.17 (s, 3H), 2.46 – 2.38 (m, 2H), 2.19 – 2.11 (m, 2H), 1.82 – 1.73 (m, 2H), 1.69 (qd, J = 7.4, 6.2 Hz, 2H), 1.46 (s, 3H), 1.41 (s, 3H), 0.96 (t, J = 7.4 Hz, 3H), 0.89 (s, 9H), 0.12 (s, 3H), 0.09 (s, 3H)
^{13}C NMR (126 MHz, $CDCl_3$) δ = 174.4, 136.8, 125.9, 110.5, 82.5, 81.9, 74.6, 71.2, 71.0, 68.1, 64.6, 61.4, 32.3, 31.9, 31.7, 31.2, 27.1, 26.3, 25.9, 23.7, 18.3, 9.6, -4.5, -5.0
IR (neat, v_{max}/cm^{-1}) 2931, 2858, 1670, 1463, 1381, 1252, 1109, 1050, 1005, 838, 779
ESI-MS calcd for $C_{26}H_{43}NNaO_5Si$ $[M+Na]^+$ 500.2803; found 500.2798
$[\alpha]^{25}_D$ = 20.1 (c = 1.0, $CHCl_3$)

Synthesis of S28

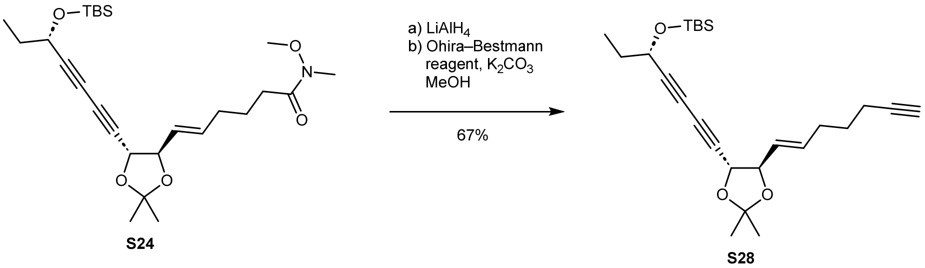

To a solution of **S24** (11 mg, 22 µmol, 1.0 equiv) in THF (50 mM) was added $LiAlH_4$ (18 µl, 2.4 M in THF, 44 µmol. 2.0 equiv) at –78°C. After complete addition and stirring for 60 min, the reaction was allowed to reach 0 °C. After 90 min, the reaction was stopped, allowed to reach rt, and quenched with 3.0 M aq. NaOH solution. Water and ethyl acetate were added, and the mixture was stirred for 1h. The mixture was filtered and concentrated under reduced pressure.
The crude aldehyde was dissolved in MeOH (50 mM) at rt, OHIRA–BESTMANN reagent (8.5 mg, 44 µmol, 2.0 equiv) and K_2CO_3 (8.2 mg, 59 µmol, 2.7 equiv) were added. The reaction was stirred for 18 h. The reaction was stopped by addition of sat. aq. NH_4Cl solution and the crude reaction mixture was extracted with ethyl acetate. The combined organic layers were dried over $MgSO_4$, filtered, and concentrated under reduced pressure. The crude mixture was purified by flash column chromatography (hexane–ethyl acetate, 15:1) resulting in title compound as pale-yellow oil (6.1 mg, 67% yield) over two steps.

^{1}H NMR (400 MHz, $CDCl_3$) δ = 5.98 – 5.80 (m, 1H), 5.47 (ddt, J = 15.3, 7.5, 1.5 Hz, 1H), 4.41 (td, J = 7.6, 0.8 Hz, 1H), 4.37 – 4.29 (m, 2H), 2.25 – 2.16 (m, 4H), 1.95 (t, J = 2.7 Hz, 1H), 1.76 – 1.60 (m, 4H), 1.47 (m, 3H), 1.42 (m, 3H), 0.97 (t, J = 7.4 Hz, 3H), 0.89 (s, 9H), 0.12 (s, 3H), 0.10 (s, 3H)
^{13}C NMR (101 MHz, $CDCl_3$) δ = 136.2, 125.9, 110.4, 84.0, 82.4, 81.8, 74.5, 71.0, 70.9, 68.7, 68.0, 64.5, 31.5, 31.1, 27.5, 26.9, 26.2, 25.7, 18.2, 17.8, 9.5, -4.6, -5.1
IR (neat, v_{max}/cm^{-1}) 2932, 2858, 1463, 1381, 1252, 1109, 1051, 838, 779
ESI-MS calcd for $C_{25}H_{38}NaO_3Si$ $[M+Na]^+$ 437.2482; found 437.2481
$[\alpha]^{26}_D$ = 38.2 (c = 1.0, $CHCl_3$)

Synthesis of 29

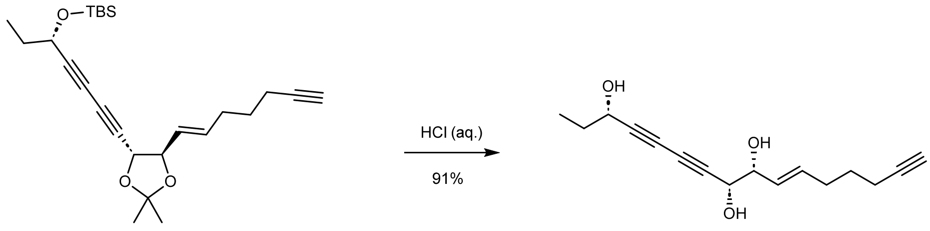

S28 **27**

To a solution of **S28** (6.1 mg, 15 mmol, 1.0 equiv) in MeOH (10 mM) at room temperature was added HCl (0.37 ml, 2.0 M in water, 0.37 mmol, 50 equiv). After complete consumption of the starting material, the reaction was stopped and quenched with aq. $NaHCO_3$ solution. The crude reaction was extracted with ethyl acetate. The combined organic layers were dried over $MgSO_4$, filtered, and concentrated under reduced pressure. The crude mixture was purified by flash column chromatography (hexane–ethyl acetate, 1:1) resulting in title compound as pale-yellow wax (3.5 mg, 91% yield).

^{1}H NMR (400 MHz, $CDCl_3$) δ = 5.85 (dtd, J = 15.1, 7.0, 1.1 Hz, 1H), 5.56 (ddt, J = 15.4, 6.8, 1.4 Hz, 1H), 4.38 (t, J = 6.6 Hz, 1H), 4.27 (d, J = 6.7 Hz, 1H), 4.14 (q, J = 6.2 Hz, 1H), 2.54 (s, 1H), 2.43 (s, 1H), 2.25 – 2.16 (m, 4H), 2.05 – 1.99 (s, 1H), 1.96 (t, J = 2.7 Hz, 1H), 1.82 – 1.70 (m, 2H), 1.70 – 1.58 (m, 2H), 1.02 (t, J = 7.4 Hz, 3H)
^{13}C NMR (101 MHz, $CDCl_3$) δ = 135.1, 127.8, 84.5, 80.9, 77.3, 75.7, 70.8, 68.9, 68.8, 66.8, 64.2, 31.4, 30.7, 27.8, 17.9, 9.5
IR (neat, v_{max}/cm^{-1}) 3351, 3299, 2969, 2935, 2878, 1432, 1336, 1273, 1094, 1051, 1015, 968, 638
ESI-MS calcd for $C_{16}H_{20}NaO_3$ $[M+Na]^+$ 283.1305; found 283.1310
$[\alpha]^{26}_D$ = 41.9 (c = 0.5, $CHCl_3$)

8 Supercritical Fluid Chromatography (SFC) Data

8.1 Synthesis of Bromo Alkyne Fragment

Enantiomeric excess was determined by SFC analysis of the corresponding 3,5-dinitrobenzoic esters **S26.**

For (*R*)-5 and (*S*)-5 (measured in 2018)

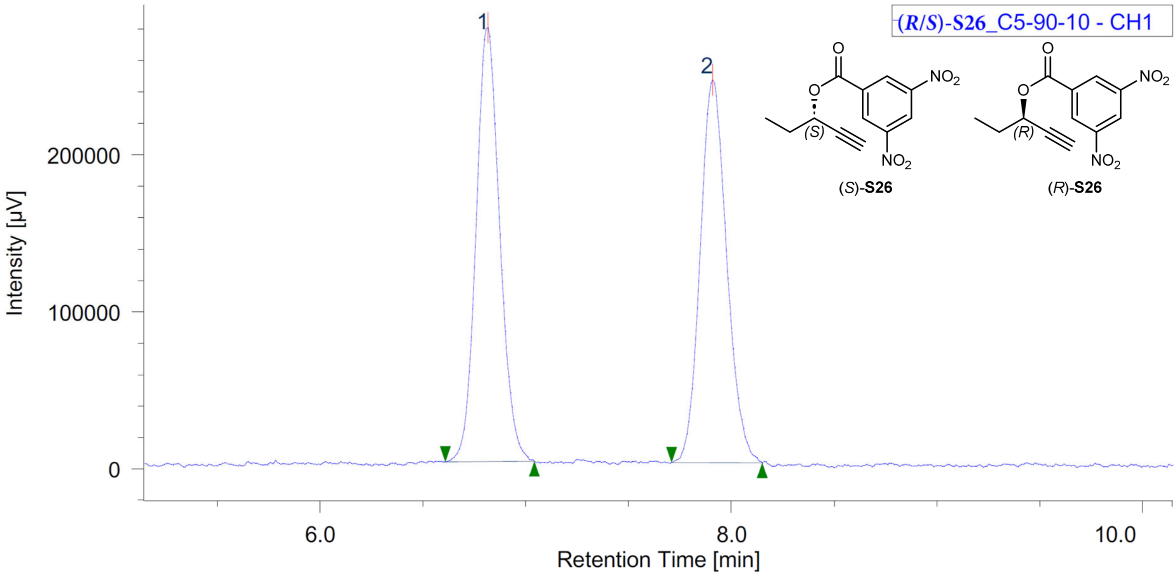

#	Peak Name	CH	tR [min]	Area [µV·sec]	Height [µV]	Area%	Height%
1	(*R*)-S26	1	6.817	2231769	276024	50.399	53.128
2	(*S*)-S26	1	7.908	2196404	243526	49.601	46.872

For (*S*)-**5**

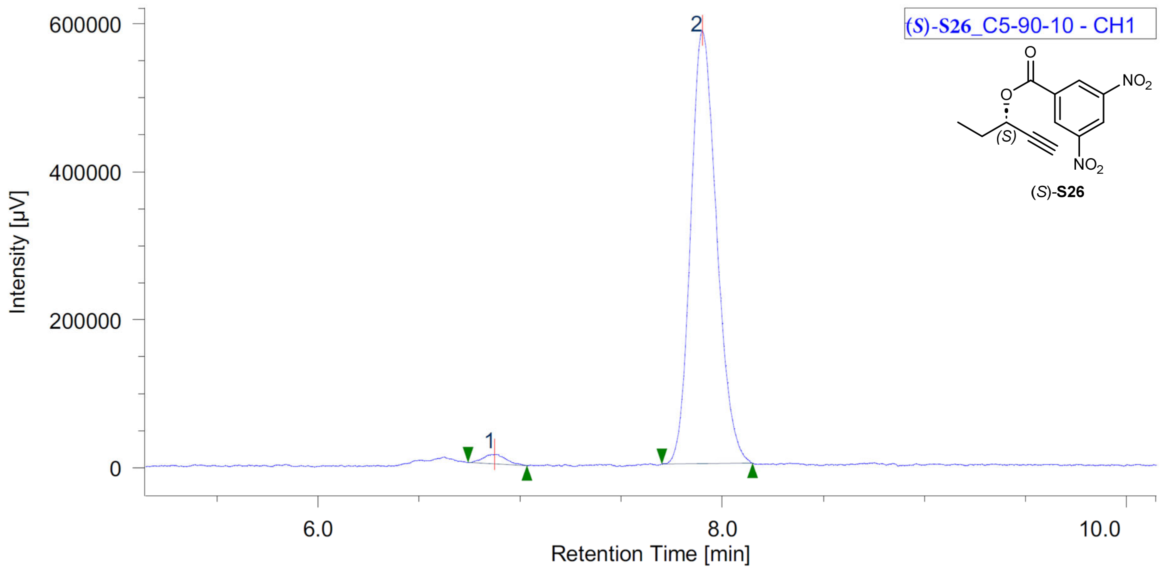

#	Peak Name	CH	tR [min]	Area [µV·sec]	Height [µV]	Area%	Height%
1	(*R*)-S26	1	6.875	100175	12795	1.881	2.140
2	(*S*)-S26	1	7.900	5224167	585174	98.119	97.860

For (*R*)-**5**

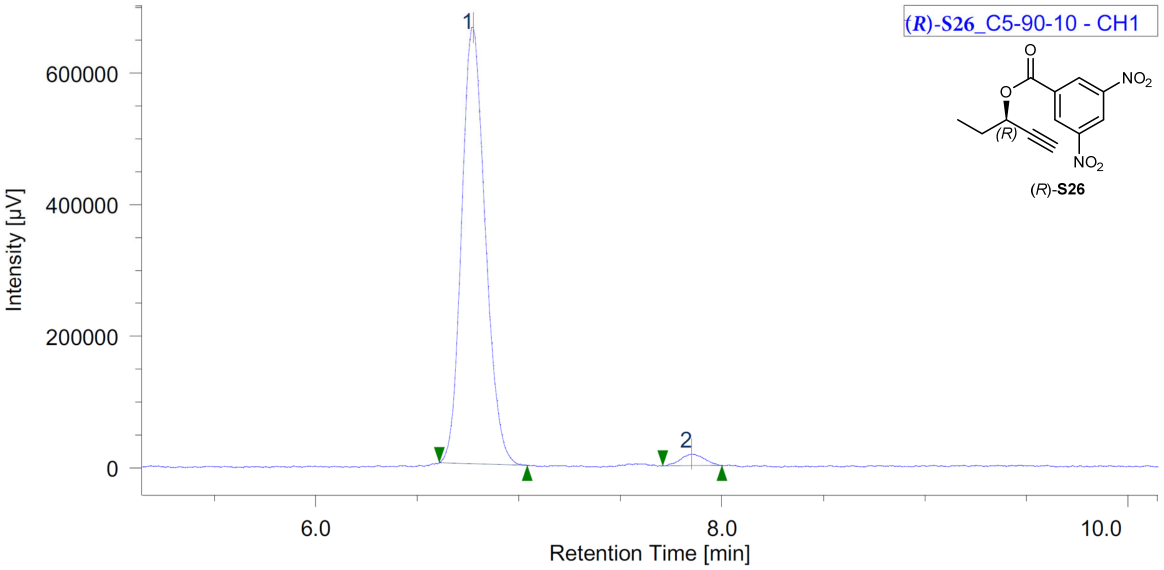

#	Peak Name	CH	tR [min]	Area [µV·sec]	Height [µV]	Area%	Height%
1	(*R*)-S26	1	6.775	5401434	662506	97.419	97.412
2	(*S*)-S26	1	7.850	143096	17598	2.581	2.588

For (*R*)-5 and (*S*)-5 (measured in 2021)

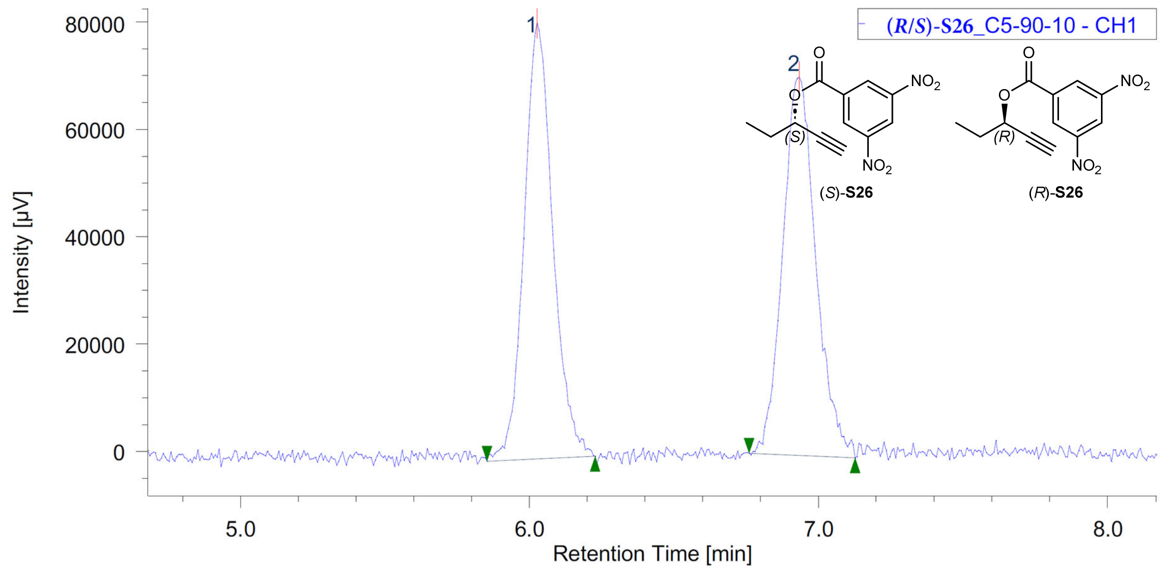

#	Peak Name	CH	tR [min]	Area [µV·sec]	Height [µV]	Area%	Height%
1	(*R*)-S26	9	6.027	560036	81114	49.927	53.512
2	(*S*)-S26	9	6.933	561670	70467	50.073	46.488

For (*S*)-13 (measured in 2021)
Enantiomeric excess was determined by SFC analysis of the corresponding 3,5-dinitrobenzoic esters **S27.**

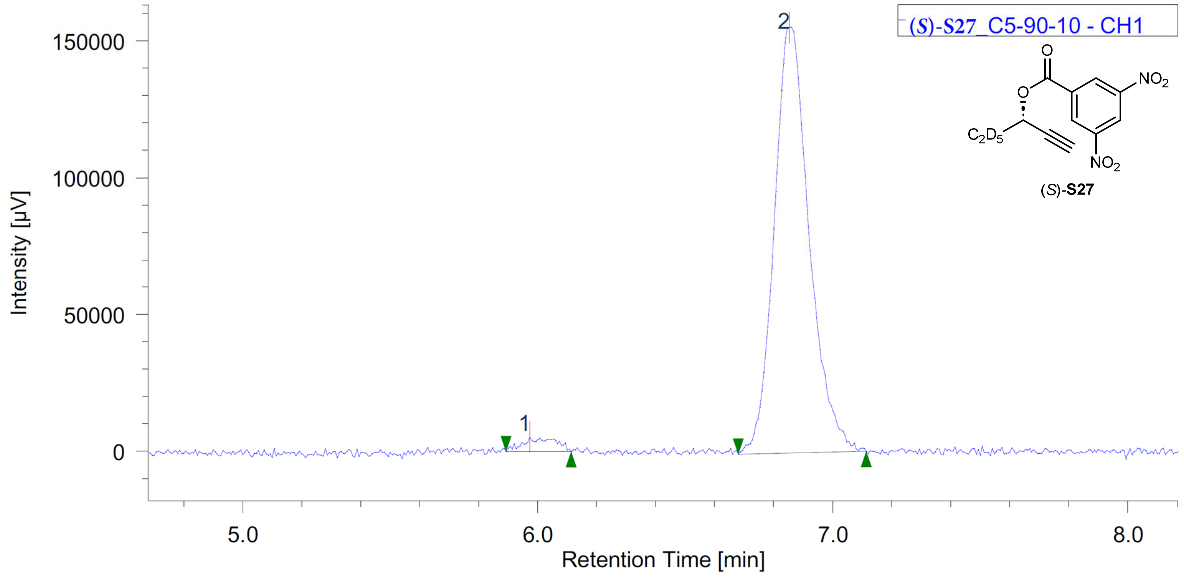

#	Peak Name	CH	tR [min]	Area [µV·sec]	Height [µV]	Area%	Height%
1	(*R*)-S27	9	5.973	39544	5217	2.986	3.250
2	(*S*)-S27	9	6.853	1284859	155321	97.014	96.750

8.2 SFC Data to Configurational Assignment of Isofalcarintriol
#NP017896 (AnalytiCon Discovery GmbH, Potsdam, Germany)

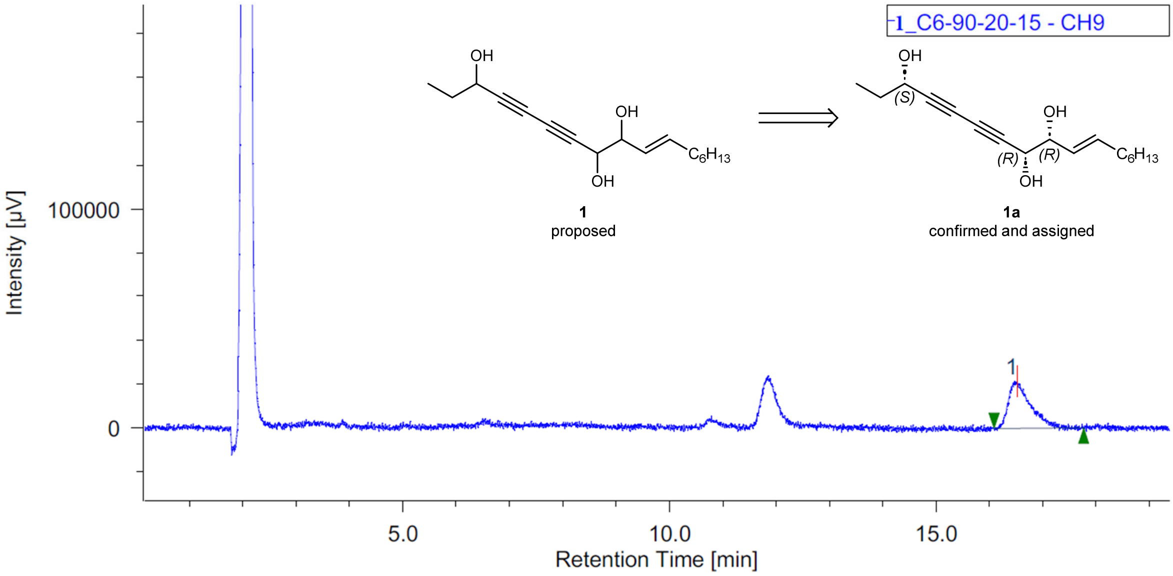

#	Peak Name	CH	tR [min]	Area [µV·sec]	Height [µV]	Area%	Height%
1	**1a**	9	16.520	623811	21537	100.000	100.000

Peaks assigned by retention time (3*R*,8*S*,9*S*)-isofalcarintriol ***ent*-1a** (12.8 min), (3*S*,8*S*,9*S*)-isofalcarintriol **1b** (13.9 min), (3*R*,8*R*,9*R*)-isofalcarintriol ***ent*-1b** (15.2 min) and (3*S*,8*R*,9*R*)-isofalcarintriol **1a** (16.5 min). Peak at 12.0 min is an unknown impurity, isofalcarintriols **1c,d** and their enantiomers can be ruled out by NMR.

1+*ent*-1a

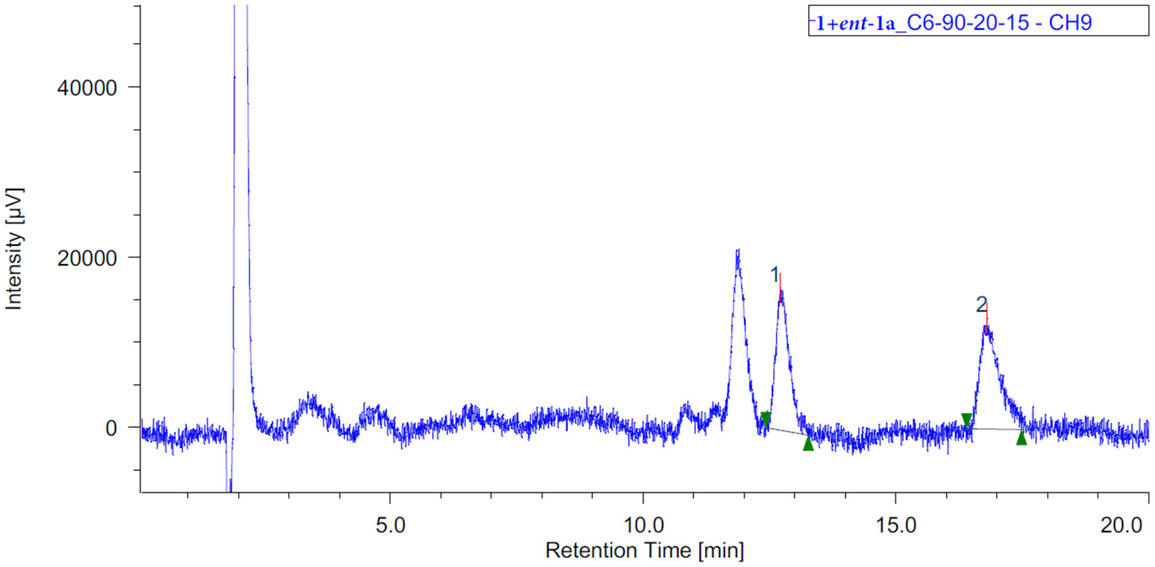

#	Peak Name	CH	tR [min]	Area [µV·sec]	Height [µV]	Area%	Height%
1	***ent*-1a**	9	12.713	313304	16714	47.711	56.264
2	**1a**	9	16.800	343367	12992	52.289	43.736

1+1b

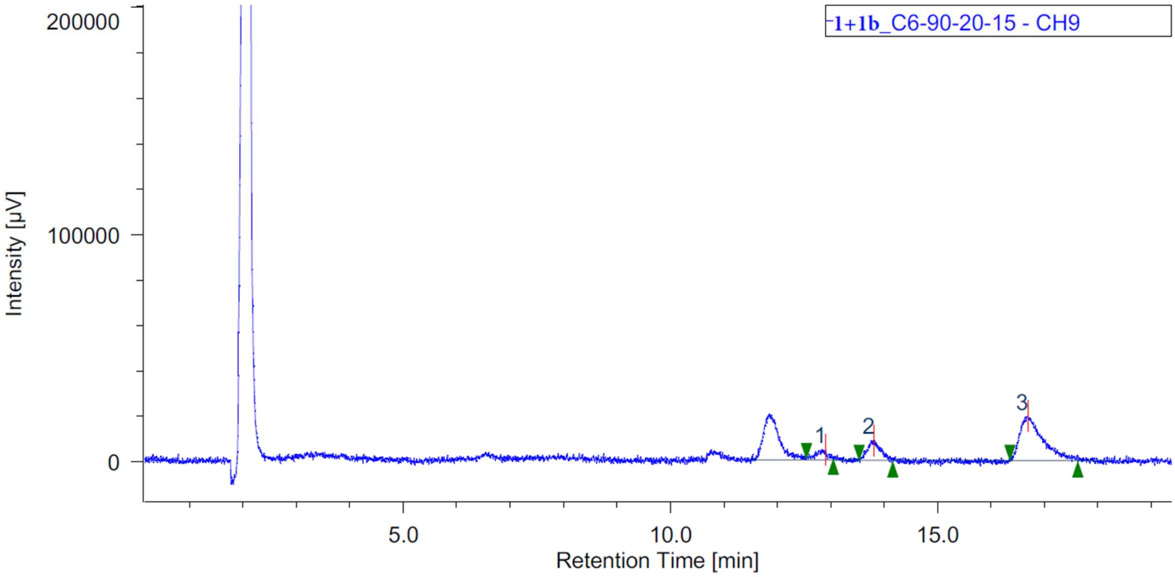

#	Peak Name	CH	tR [min]	Area [µV·sec]	Height [µV]	Area%	Height%
1	***ent*-1a**	9	12.907	71640	4709	9.011	14.288
2	**1b**	9	13.807	144276	8837	18.148	26.814
3	**1a**	9	16.680	579066	19411	72.840	58.898

1+*ent*-1b

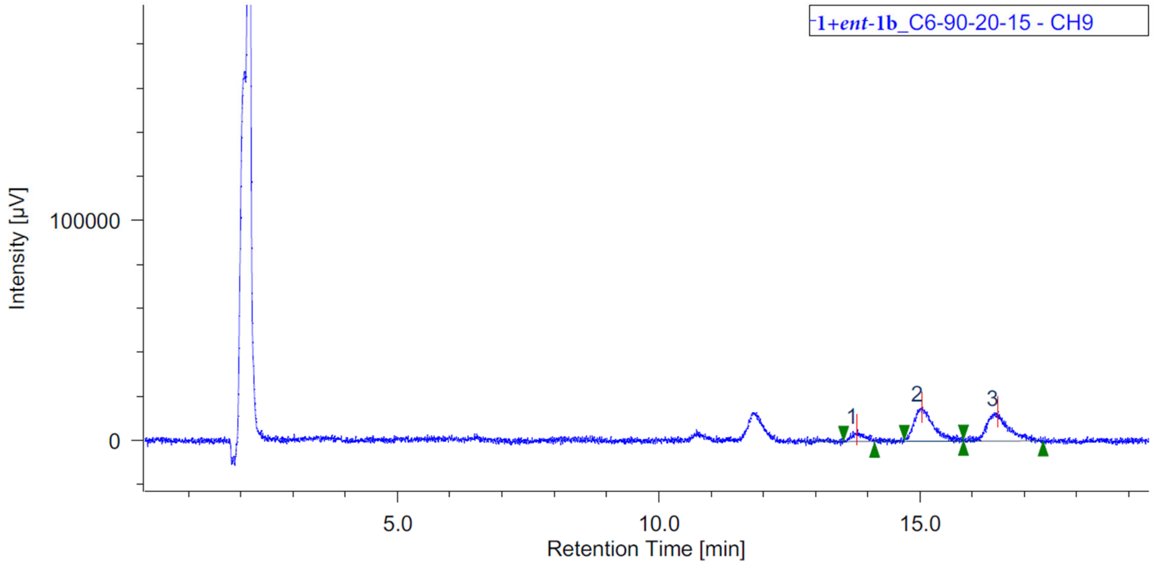

#	Peak Name	CH	tR [min]	Area [µV·sec]	Height [µV]	Area%	Height%
1	**1b**	9	13.800	73460	5371	8.807	15.699
2	***ent*-1b**	9	15.040	388089	15447	46.525	45.154
3	**1a**	9	16.487	372606	13392	44.669	39.147

***ent*-1b**

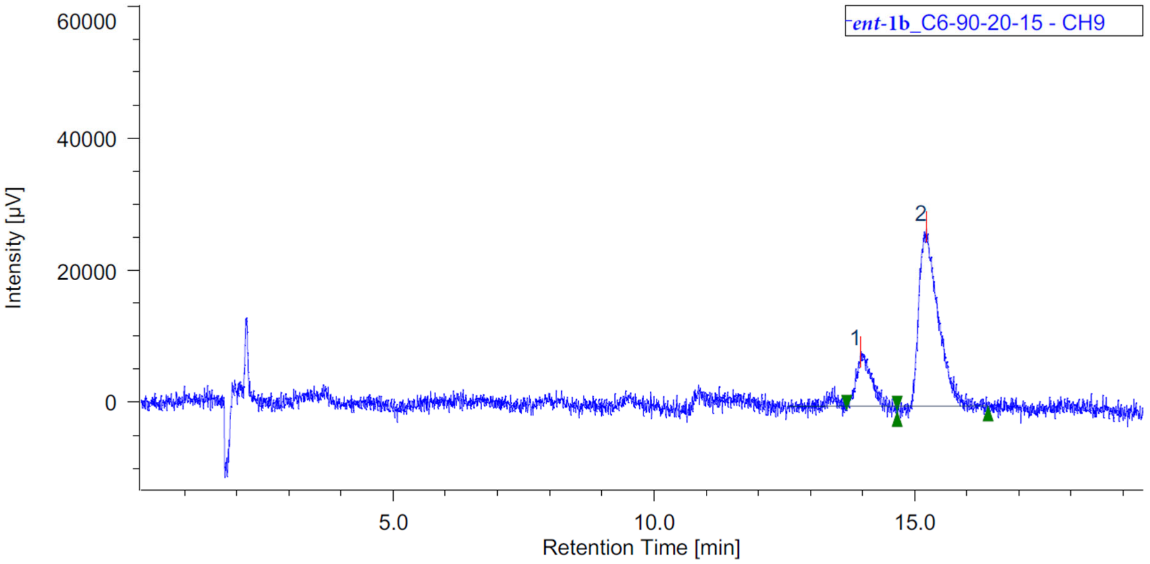

#	Peak Name	CH	tR [min]	Area [µV·sec]	Height [µV]	Area%
1	**1b**	9	13.960	159425	8260	18.892
2	***ent*-1b**	9	15.220	684451	27198	81.108

1+1a

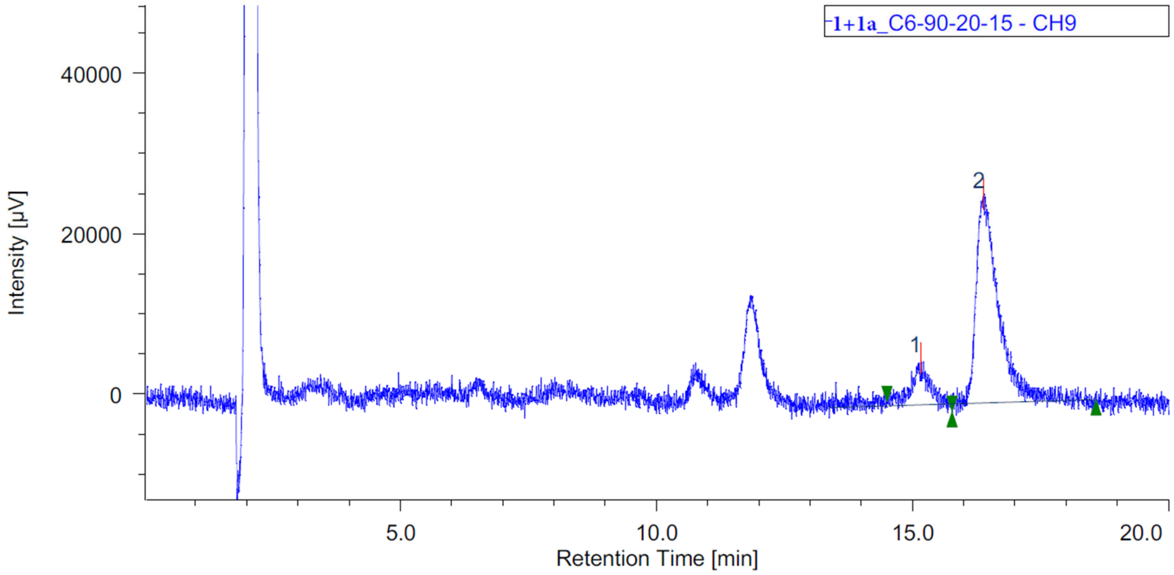

#	Peak Name	CH	tR [min]	Area [µV·sec]	Height [µV]	Area%	Height%
1	***ent*-1b**	9	15.160	133223	5807	14.328	18.264
2	**1a**	9	16.393	796612	25987	85.672	81.736

1a

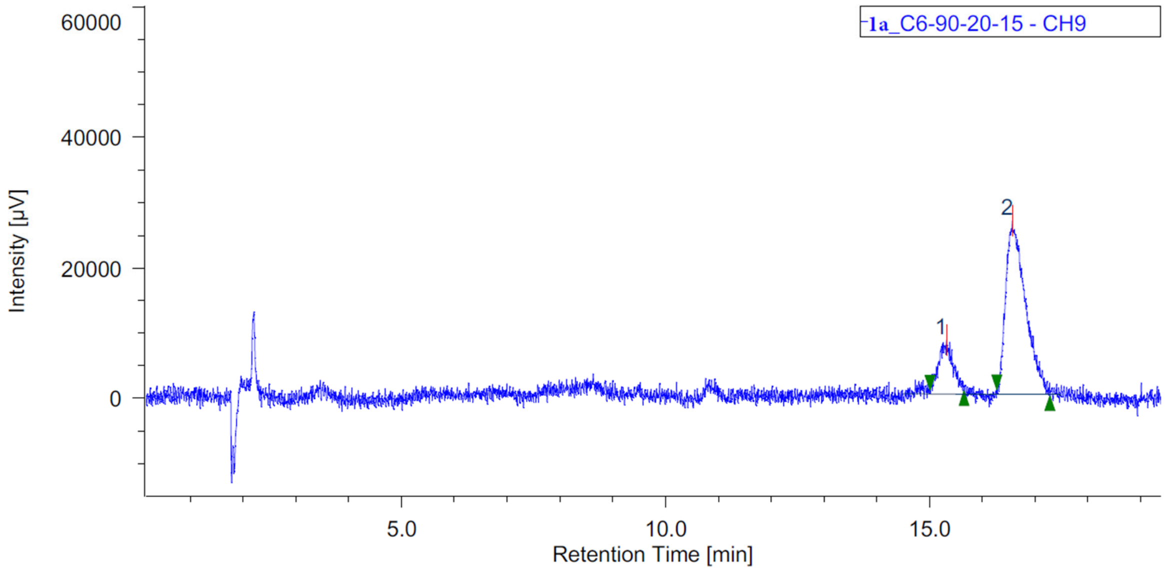

#	Peak Name	CH	tR [min]	Area [µV·sec]	Height [µV]	Area%	Height%
1	***ent*-1b**	9	15.320	156654	8212	18.611	23.601
2	**1a**	9	16.567	685084	26584	81.389	76.399

9 NMR Data

9.1 Spectra to Asymmetric Synthesis of Isofalcarintriol

9.1.1 Synthesis of anti-Fragment

Synthesis of S25 and *ent*-S25 (^{1}H, ^{13}C spectra)

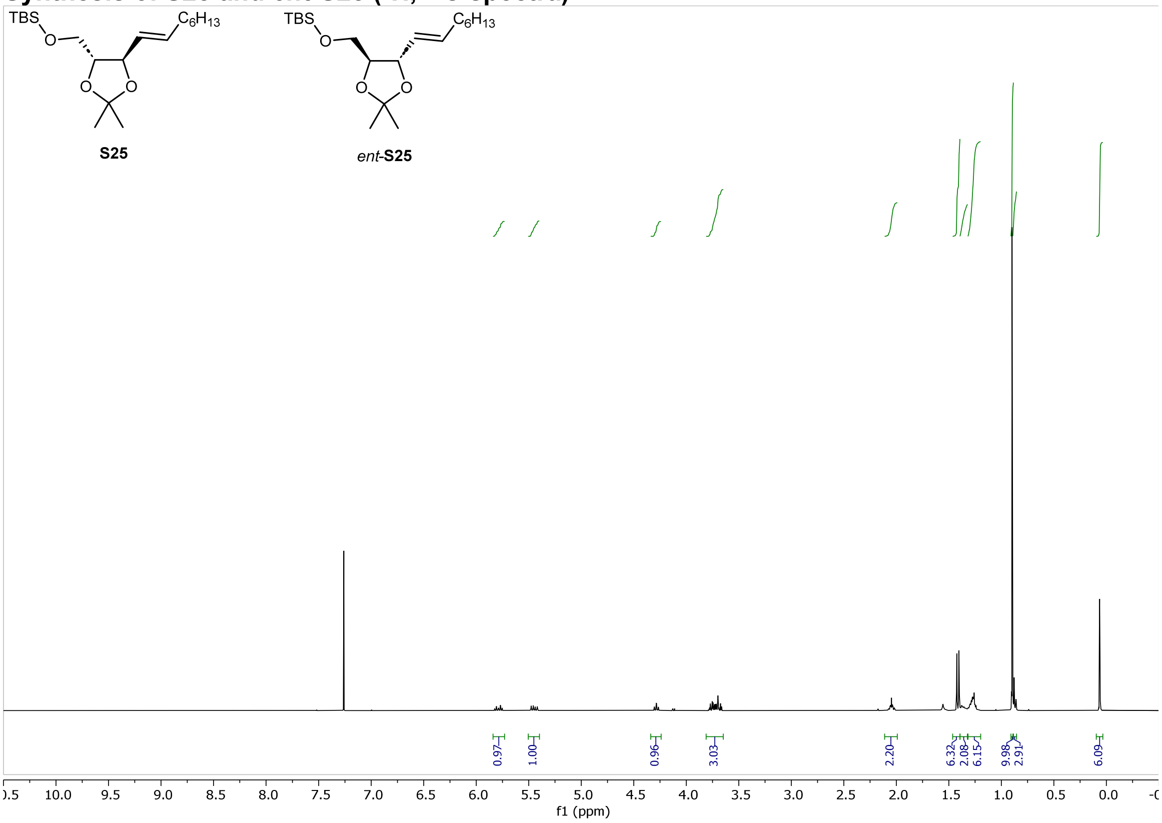

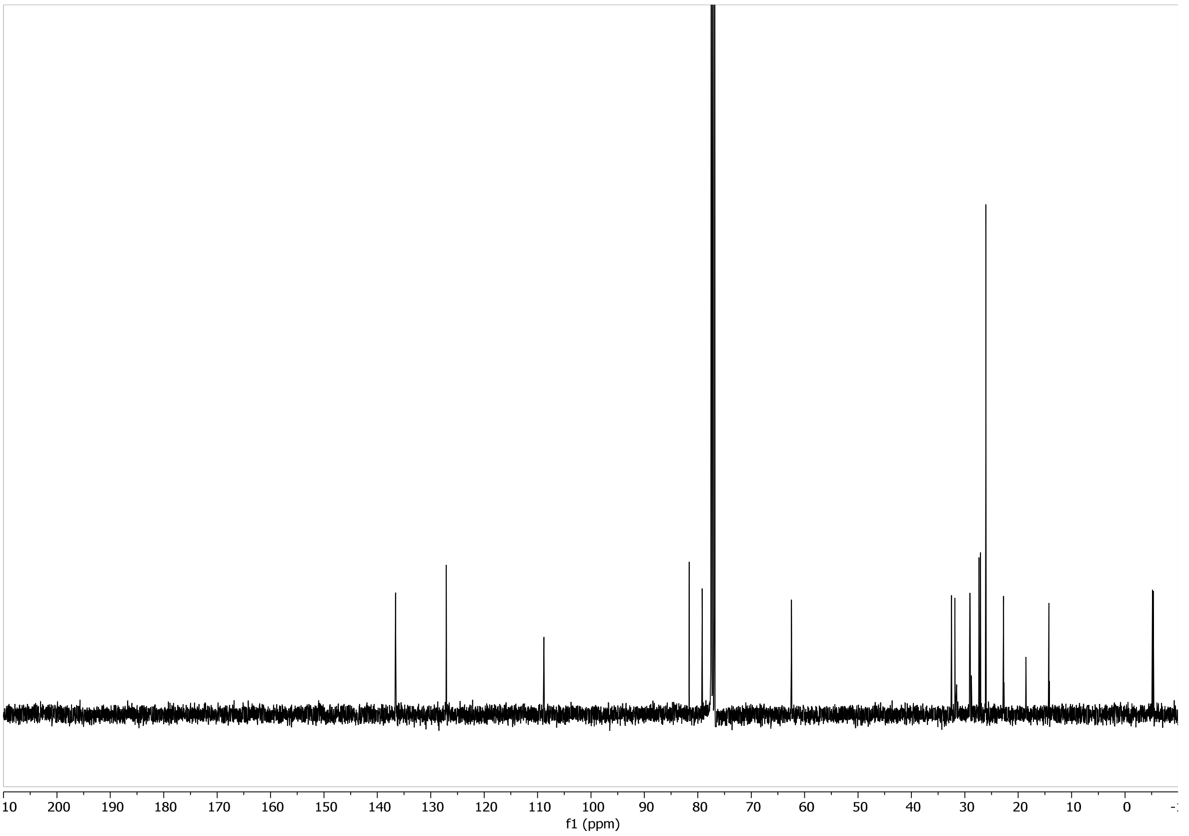

f1 (ppm)

Synthesis of 11 and *ent*-11 (^{1}H, ^{13}C spectra)

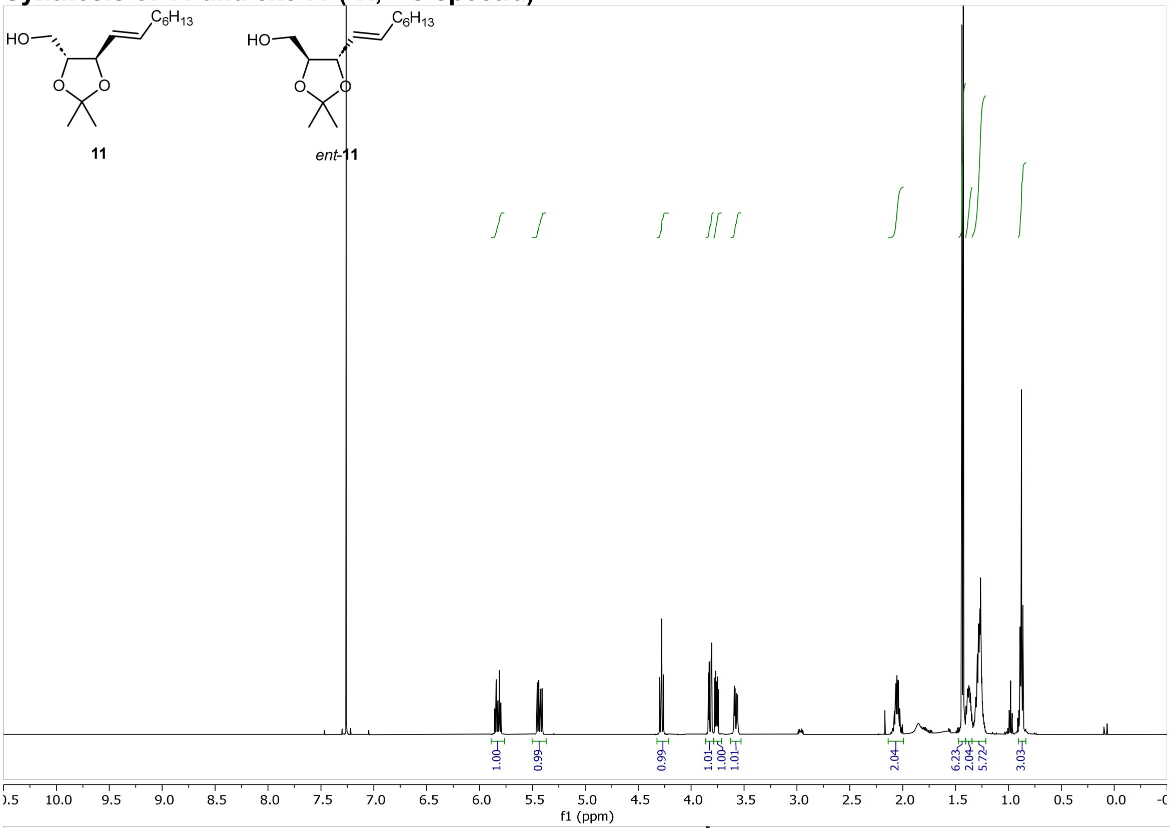

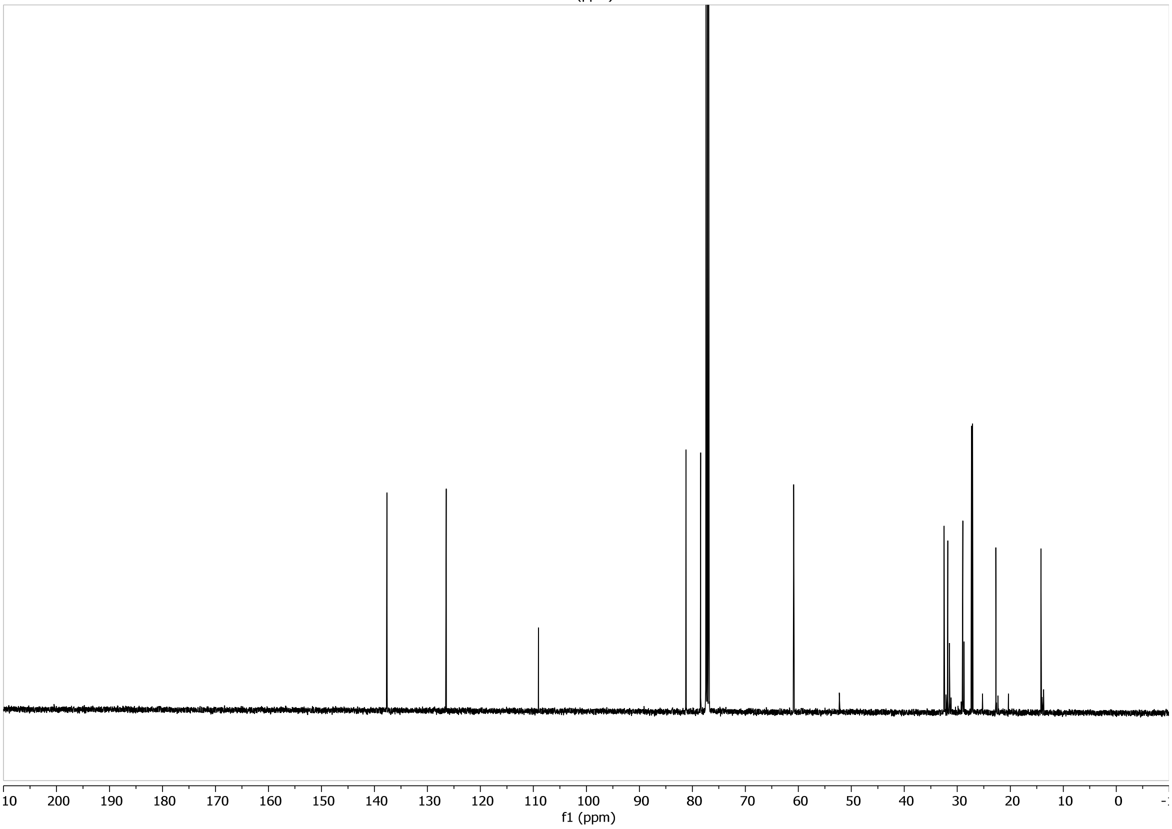

Synthesis of 4a and *ent*-4a (^{1}H, ^{13}C spectra)

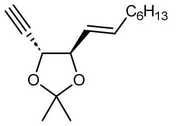

4a

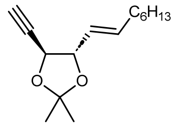

ent-**4a**

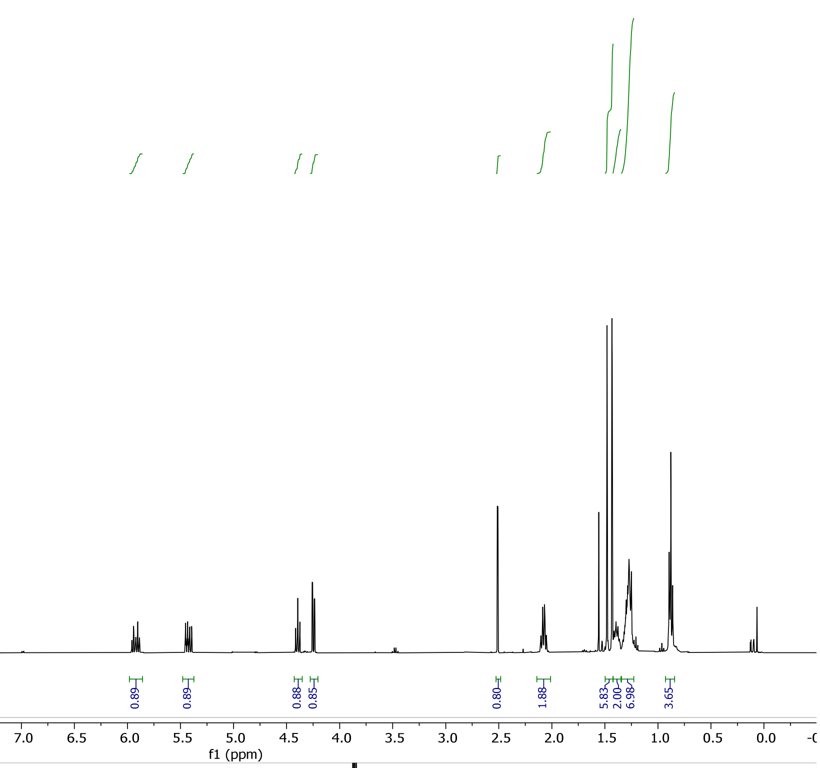

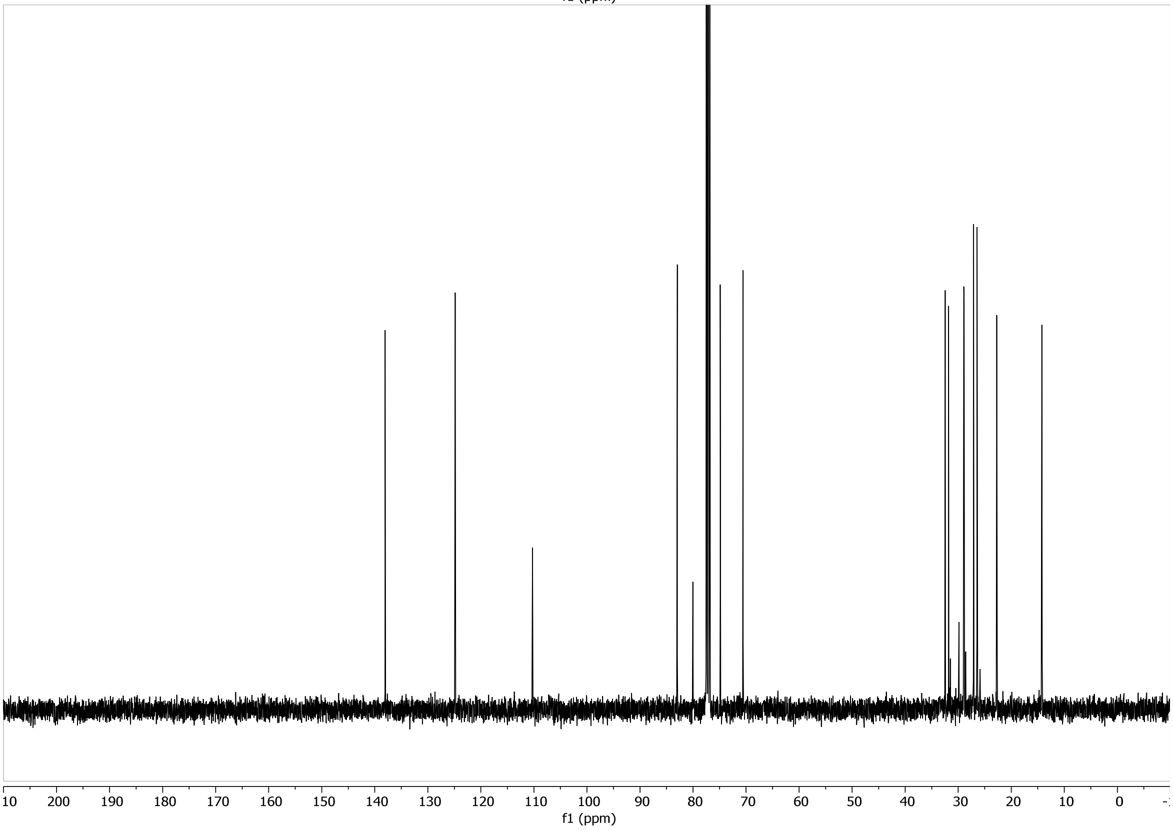

9.1.2 Synthesis of Bromo Alkyne Fragment

Synthesis of (*S*)-5 and (*R*)-5 (^{1}H, ^{13}C spectra)

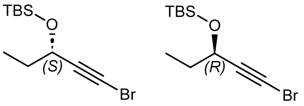

(*S*)-**5** (*R*)-**5**

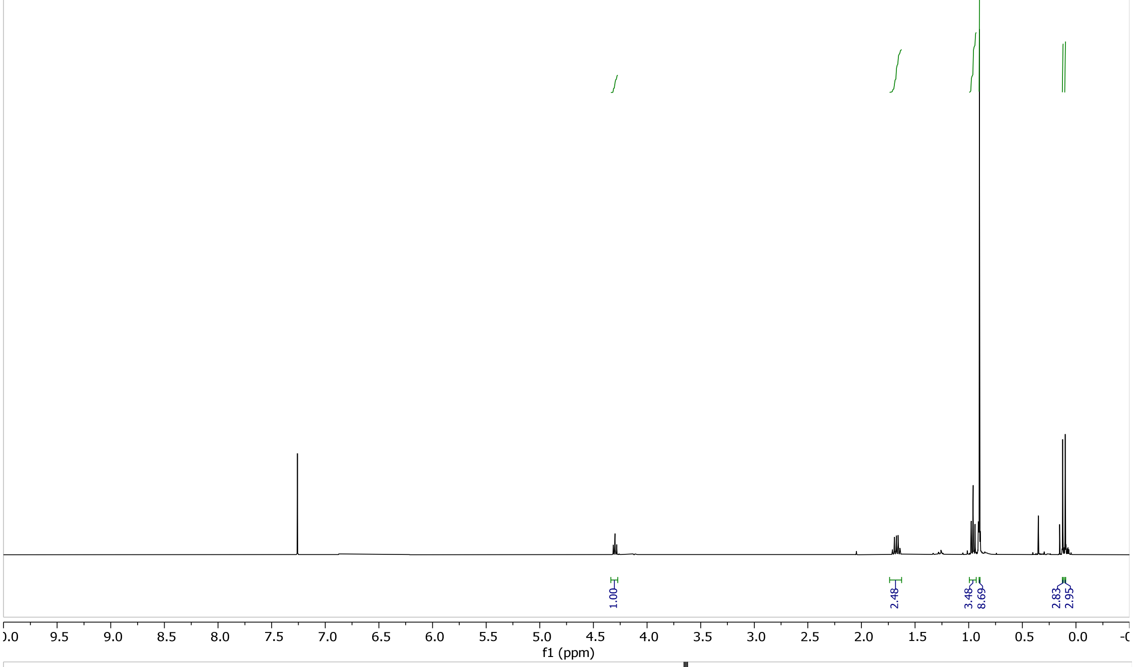

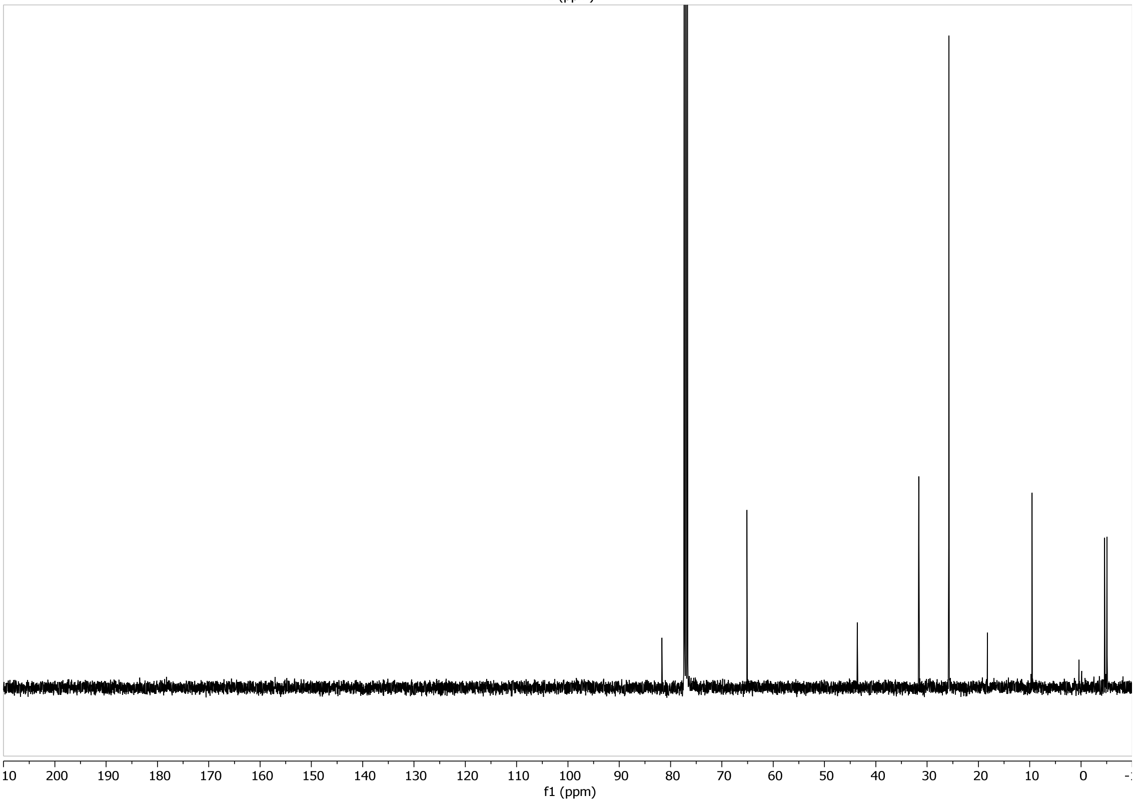

9.1.3 Synthesis of syn-Fragment

Synthesis of S7 and *ent*-S7 (^{1}H, ^{13}C spectra)

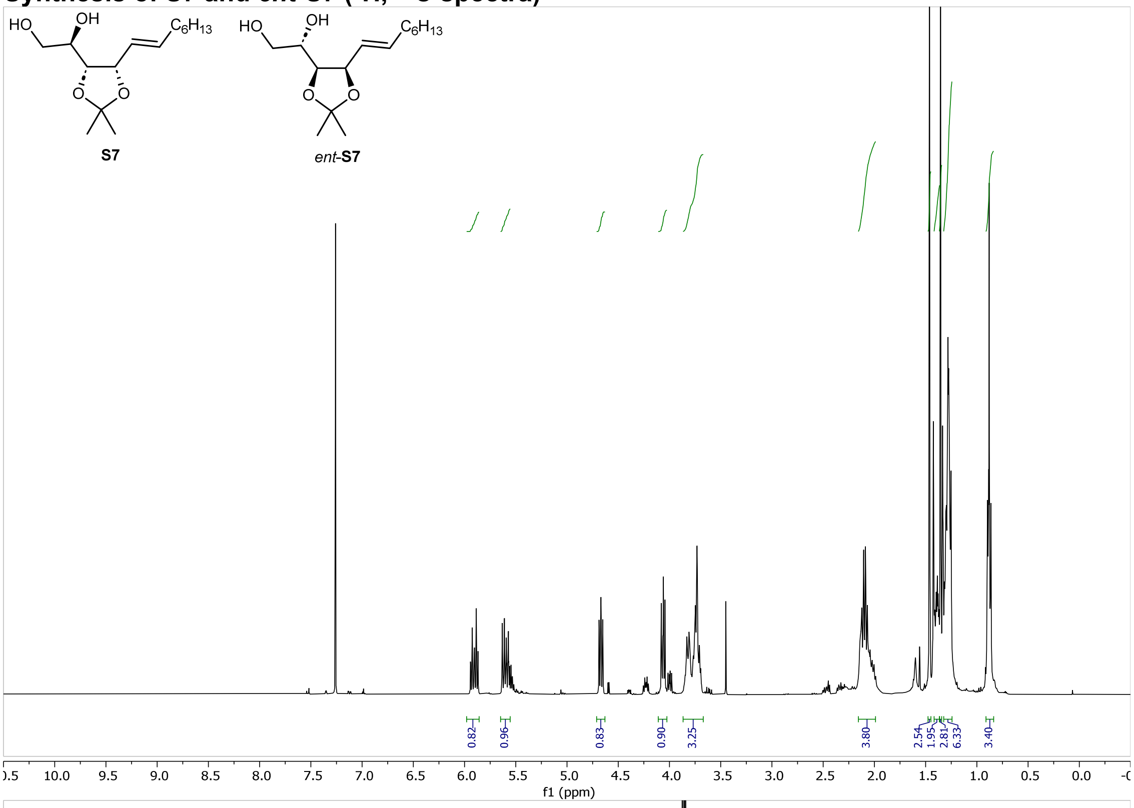

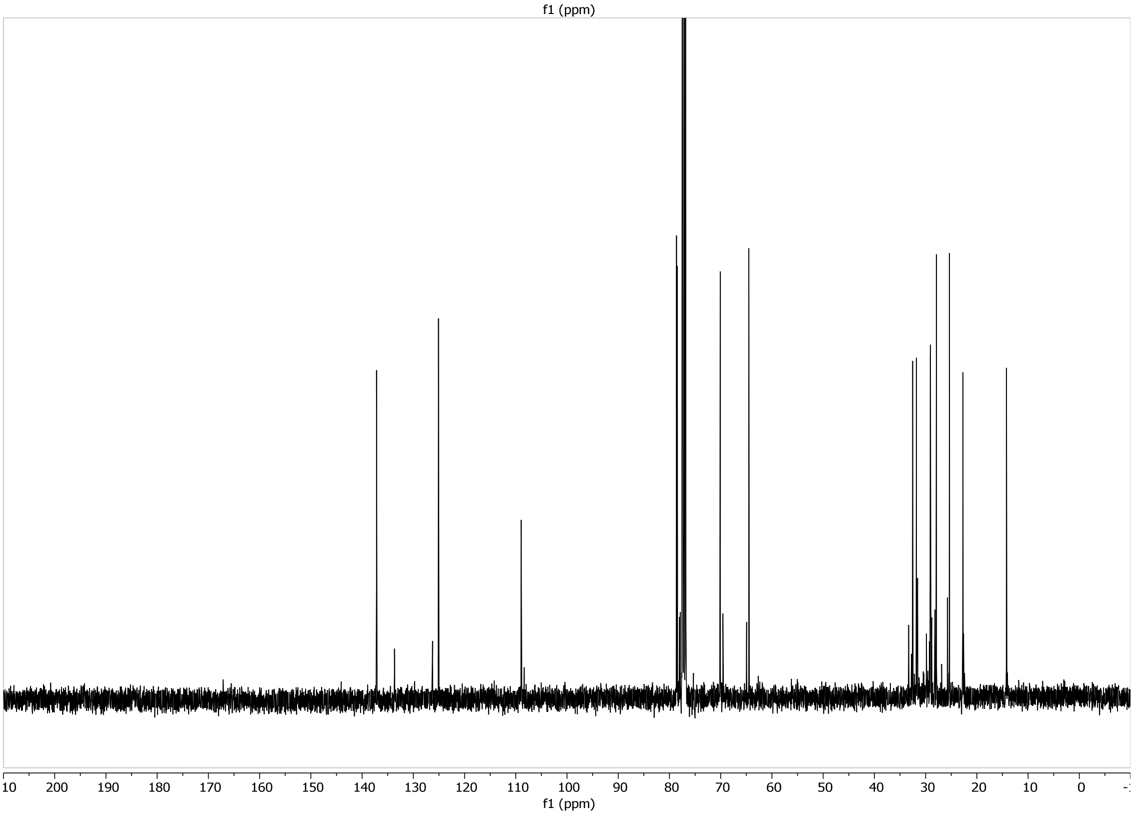

Synthesis of 4b and *ent*-4b (^{1}H, ^{13}C spectra)

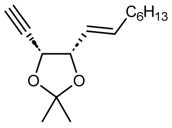

4b

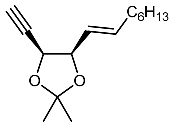

ent-**4b**

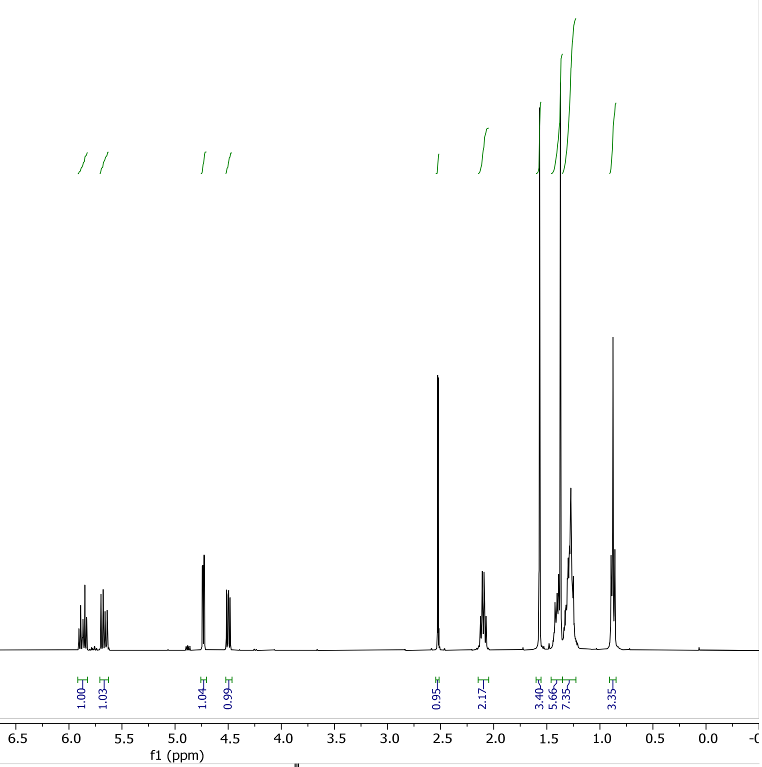

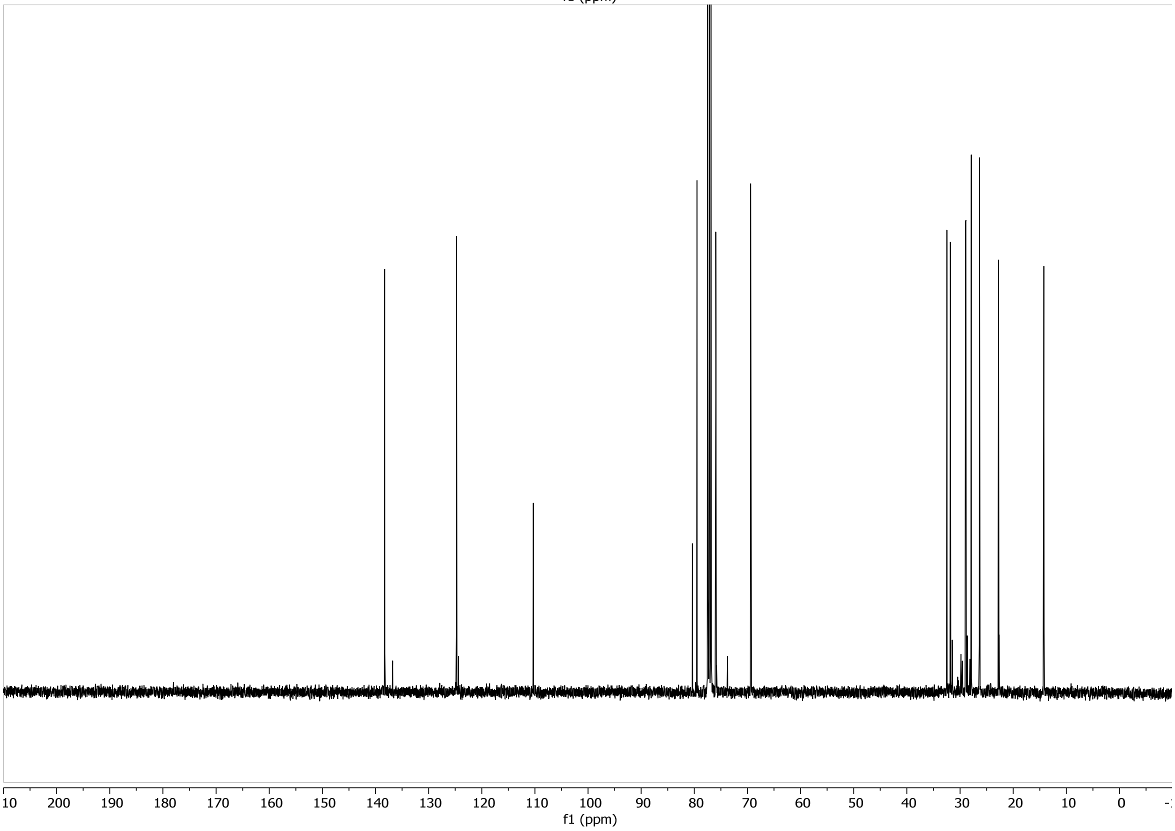

9.1.4 Synthesis of Isofalcarintriols

Synthesis of S15a and *ent*-S15a (^{1}H, ^{13}C, HSQC, HMBC, COSY spectra)

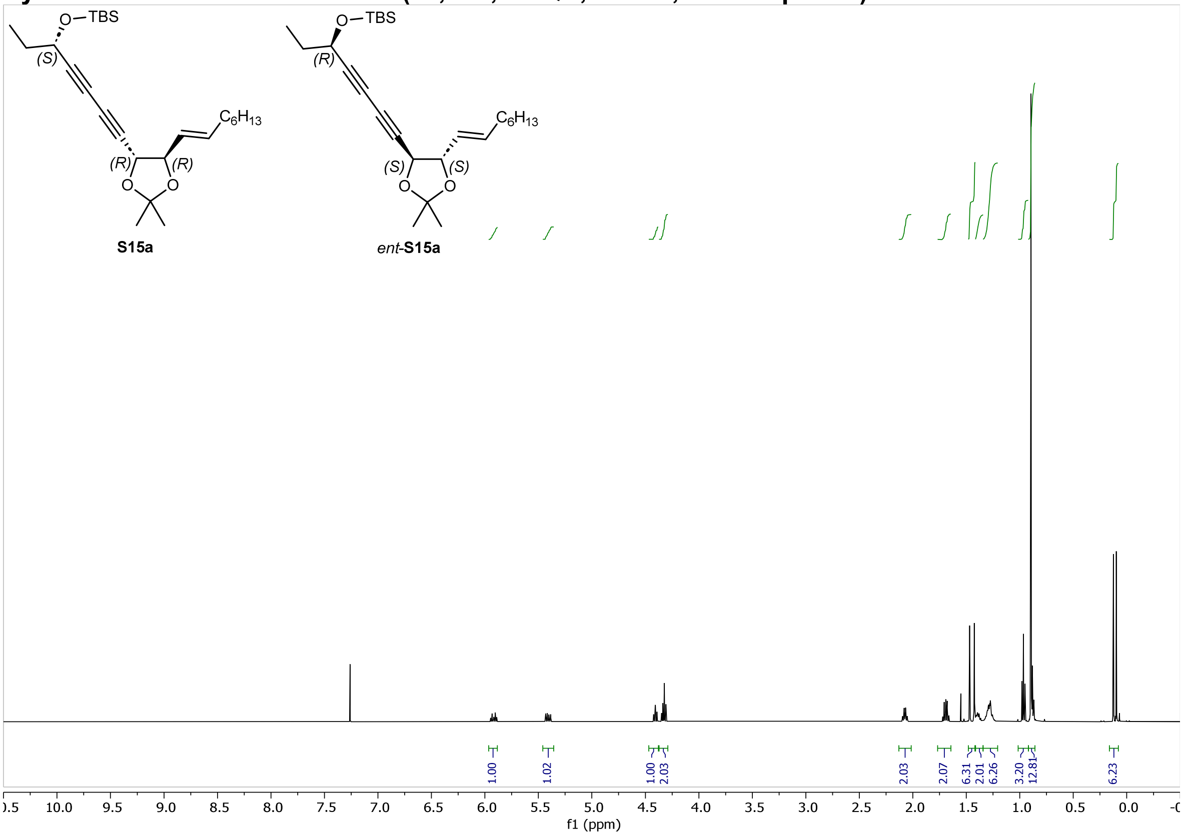

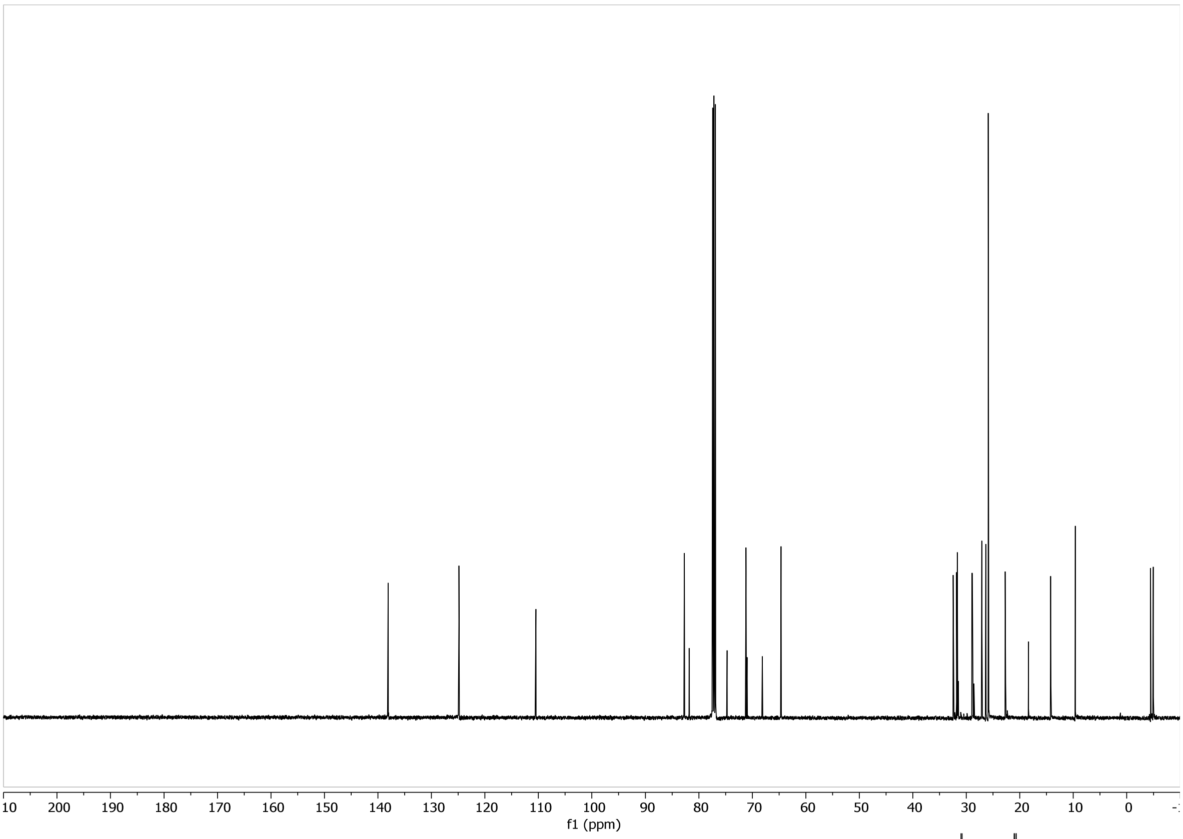
10 200 190 180 170 160 150 140 130 120 110 100 90 80 70 60 50 40 30 20 10 0
f1 (ppm)

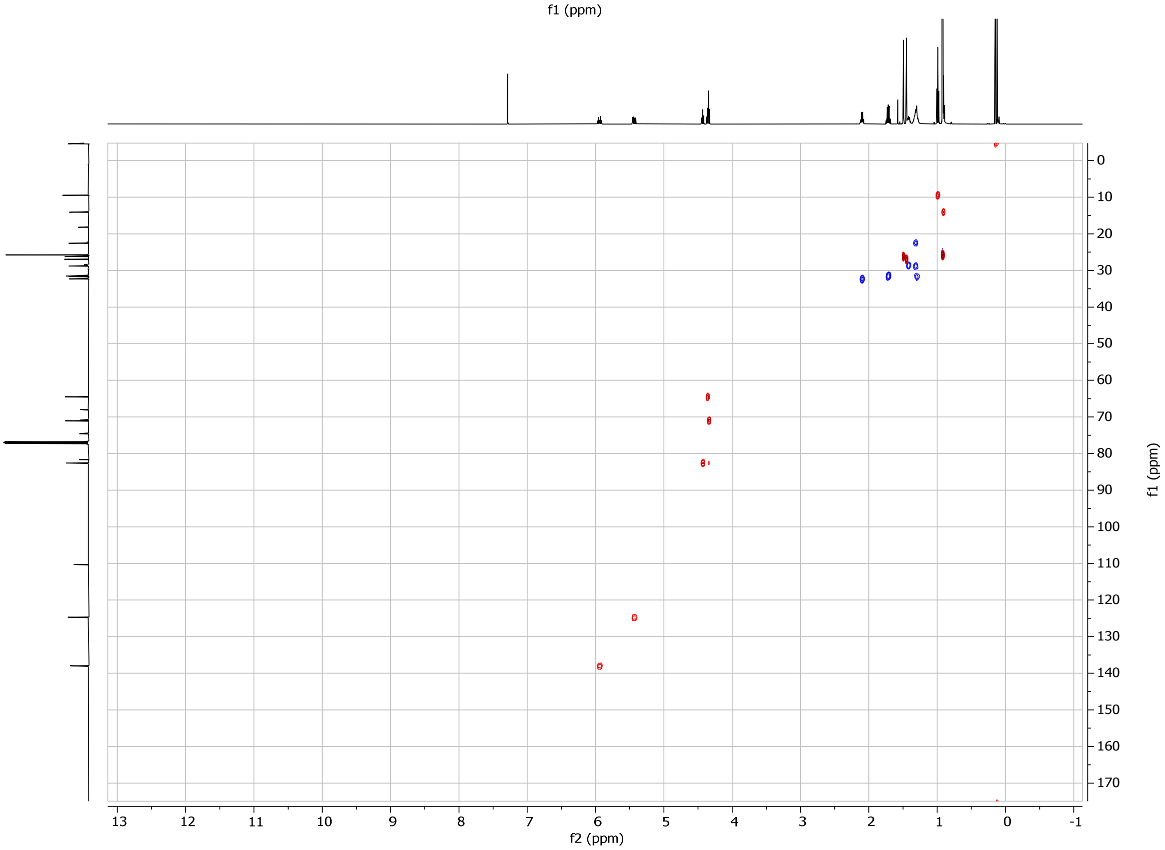
0 10 20 30 40 50 60 70 80 90 100 110 120 130 140 150 160 170
f1 (ppm)
13 12 11 10 9 8 7 6 5 4 3 2 1 0 -1
f2 (ppm)

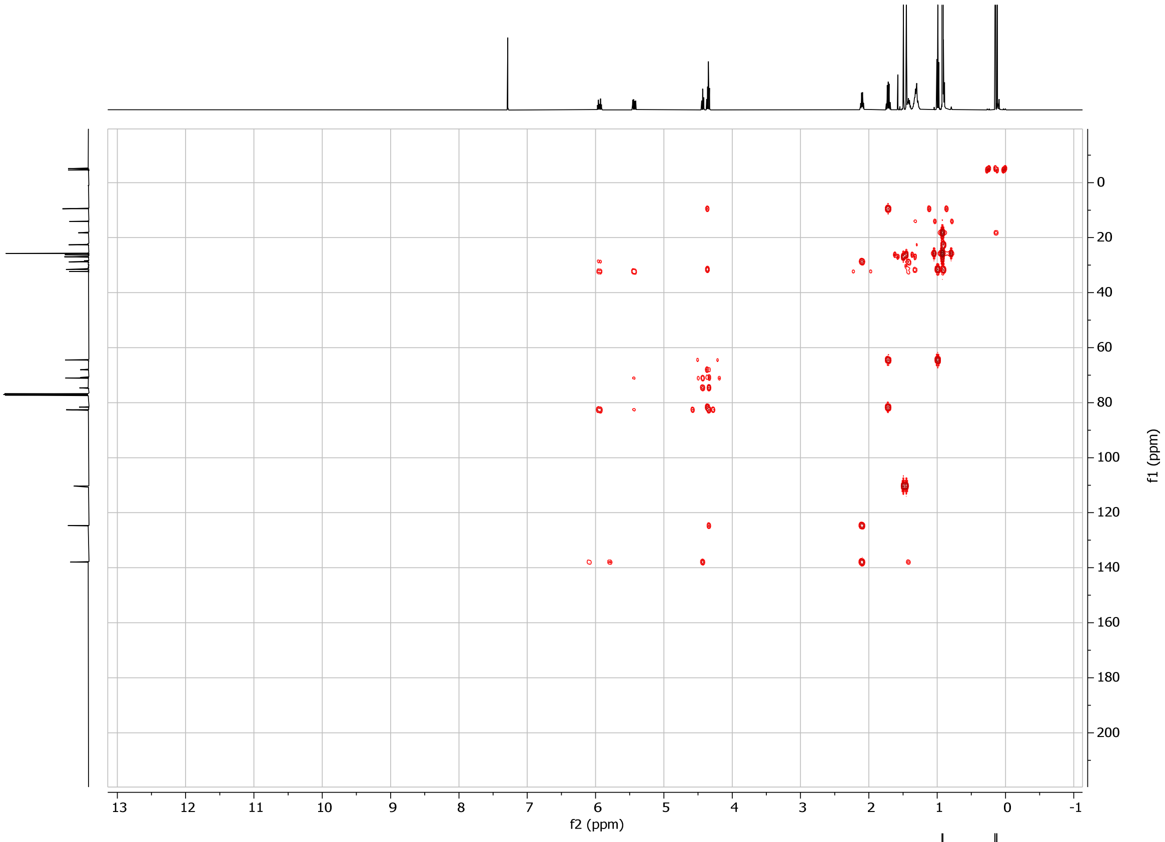
f2 (ppm)
f1 (ppm)

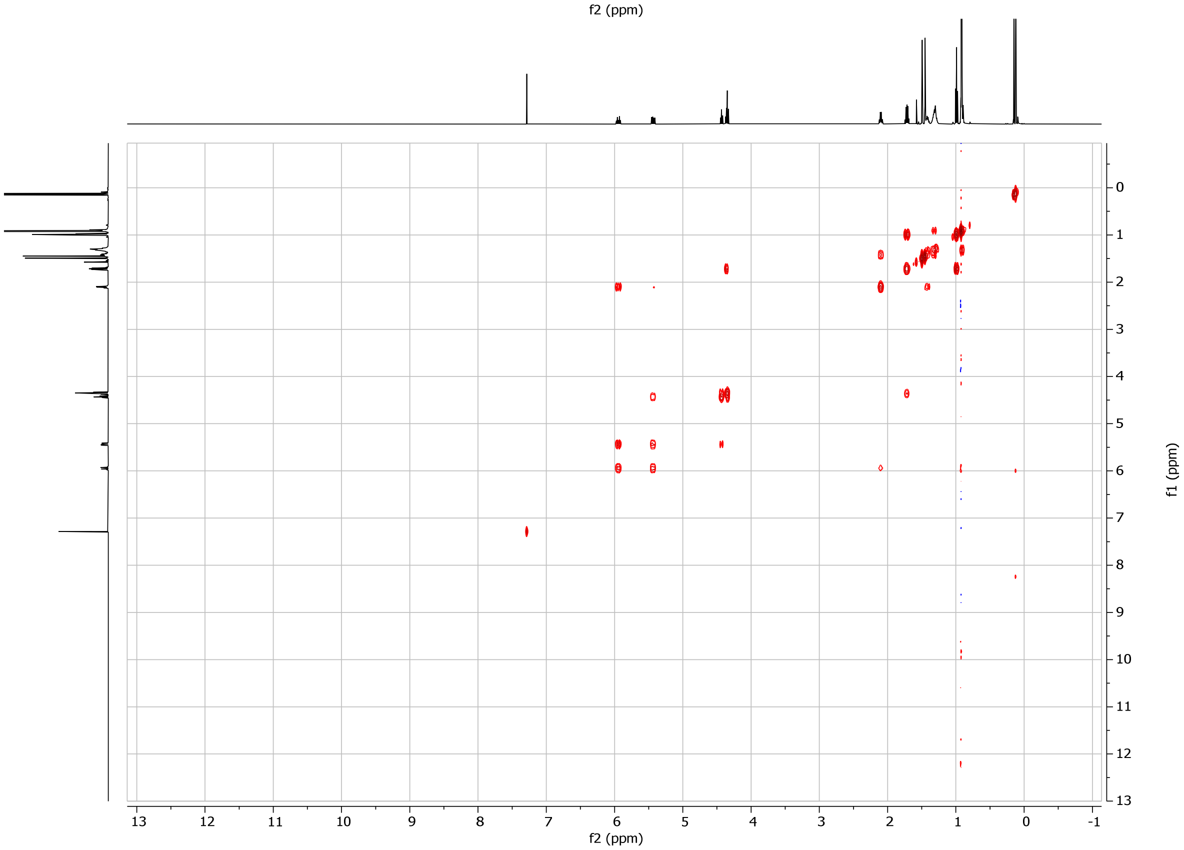
f2 (ppm)
f1 (ppm)

Synthesis of 1a and *ent*-1a (^{1}H, ^{13}C, HSQC, HMBC, COSY spectra)

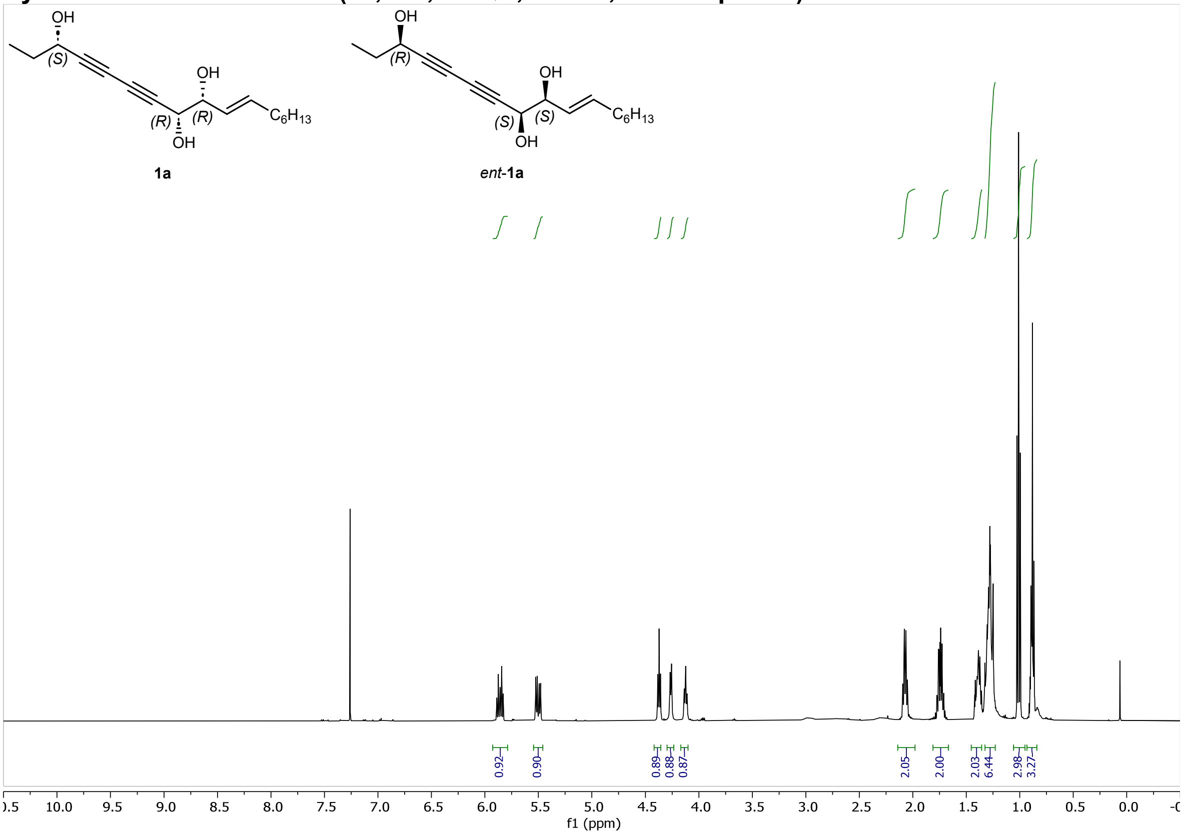

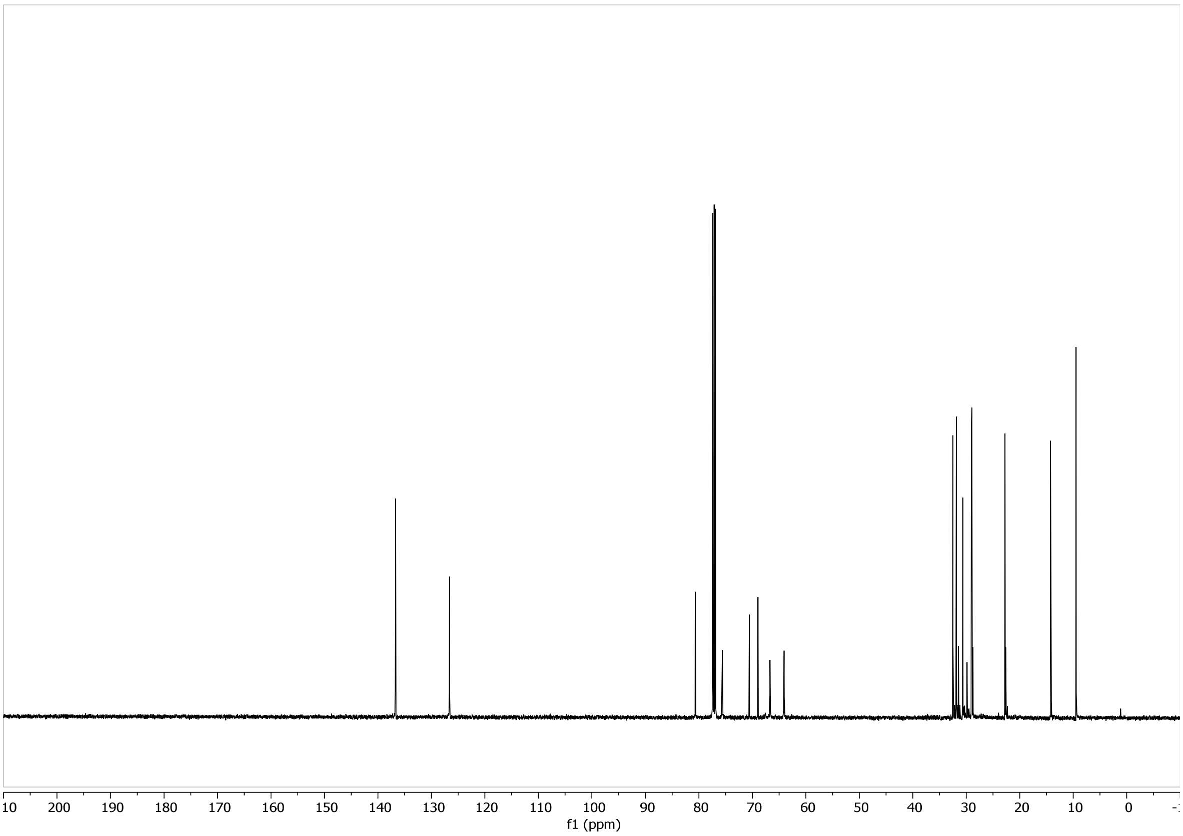

10
200
190
180
170
160
150
140
130
120
110
100
90
80
70
60
50
40
30
20
10
0
f1 (ppm)

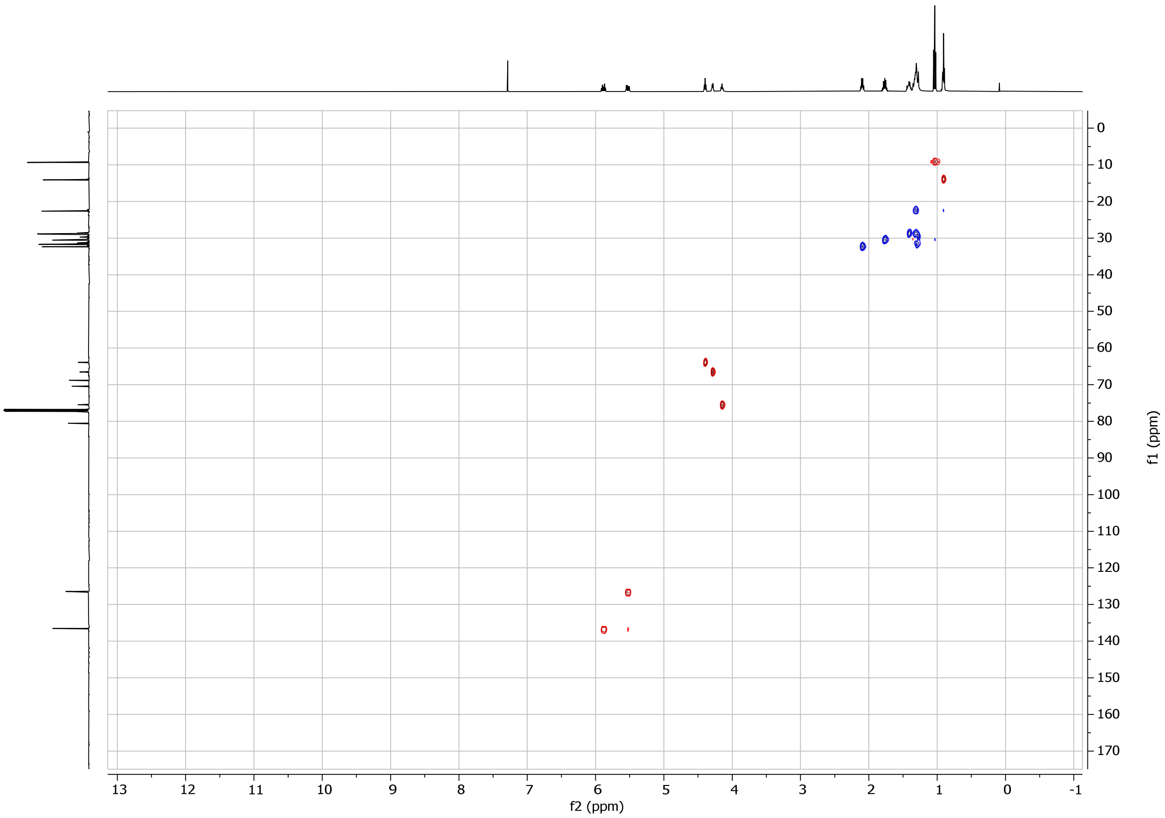

13
12
11
10
9
8
7
6
5
4
3
2
1
0
-1
f2 (ppm)
0
10
20
30
40
50
60
70
80
90
100
110
120
130
140
150
160
170
f1 (ppm)

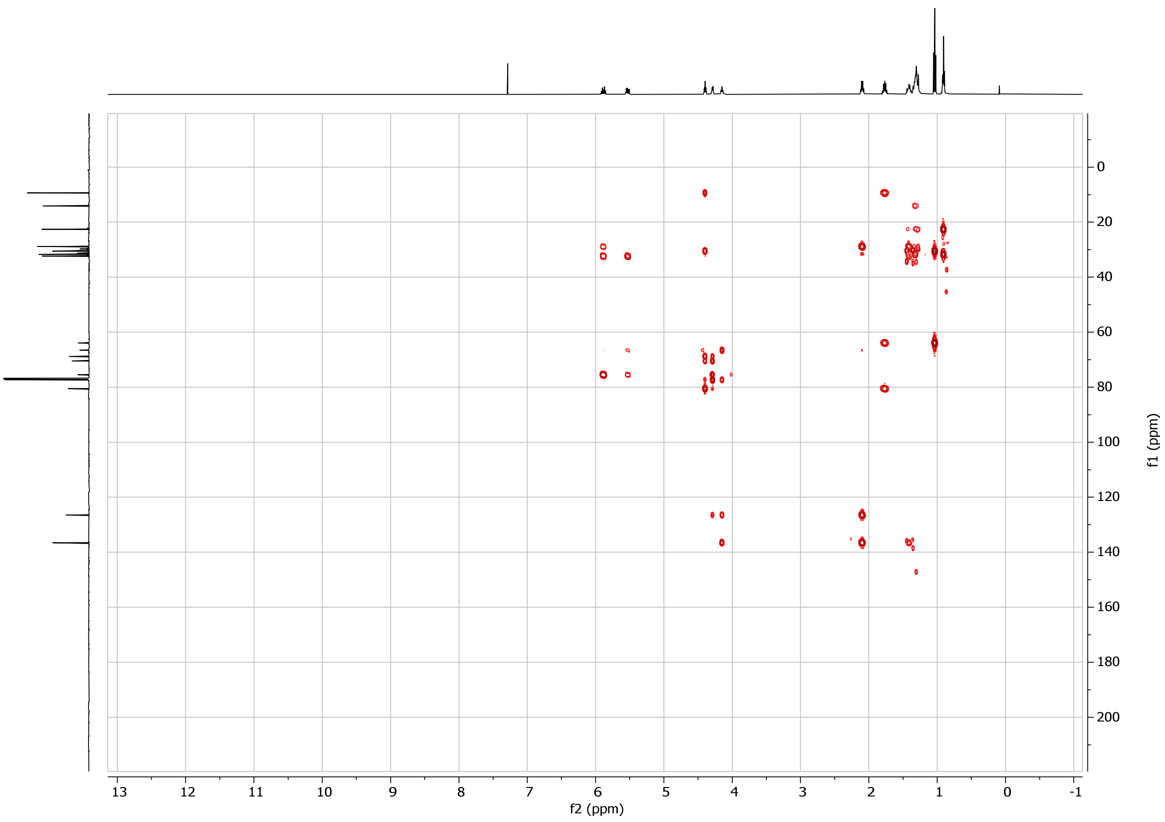
f2 (ppm)
f1 (ppm)

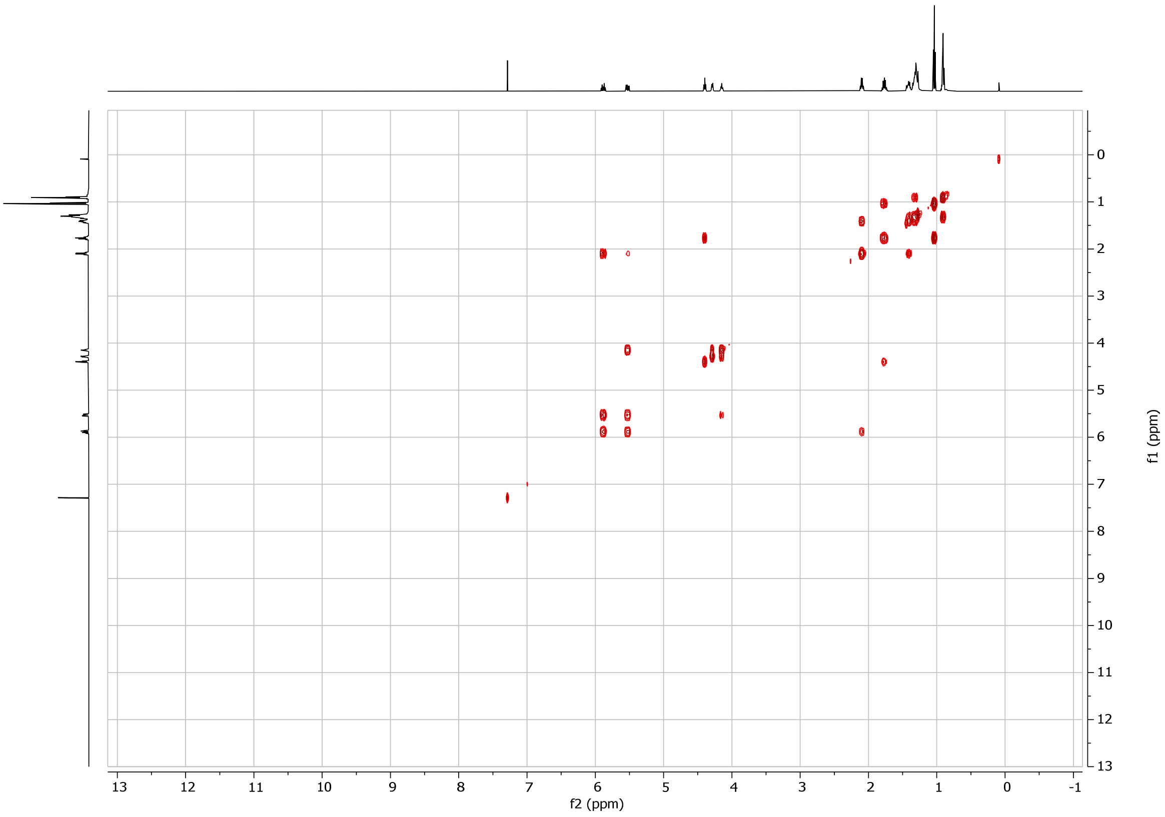
f2 (ppm)
f1 (ppm)

Synthesis of S15b and *ent*-S15b (^{1}H, ^{13}C, HSQC, HMBC, COSY spectra)

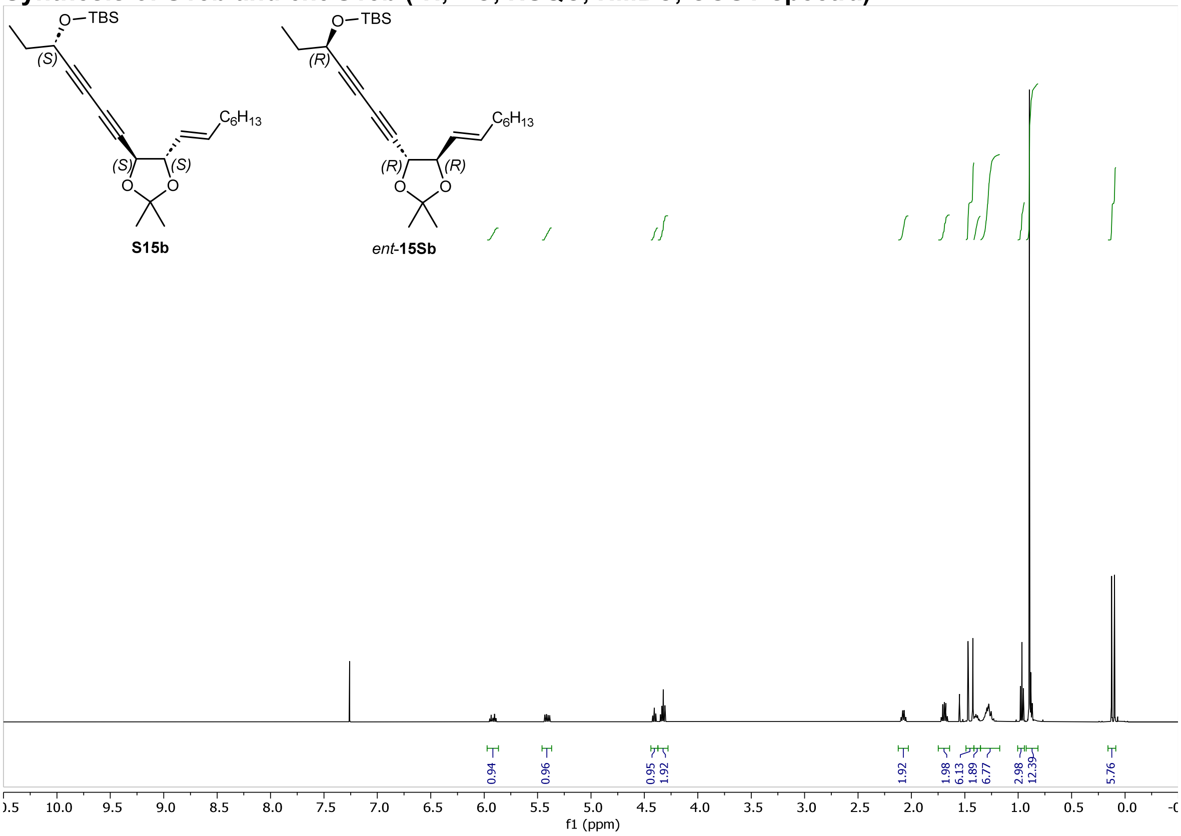

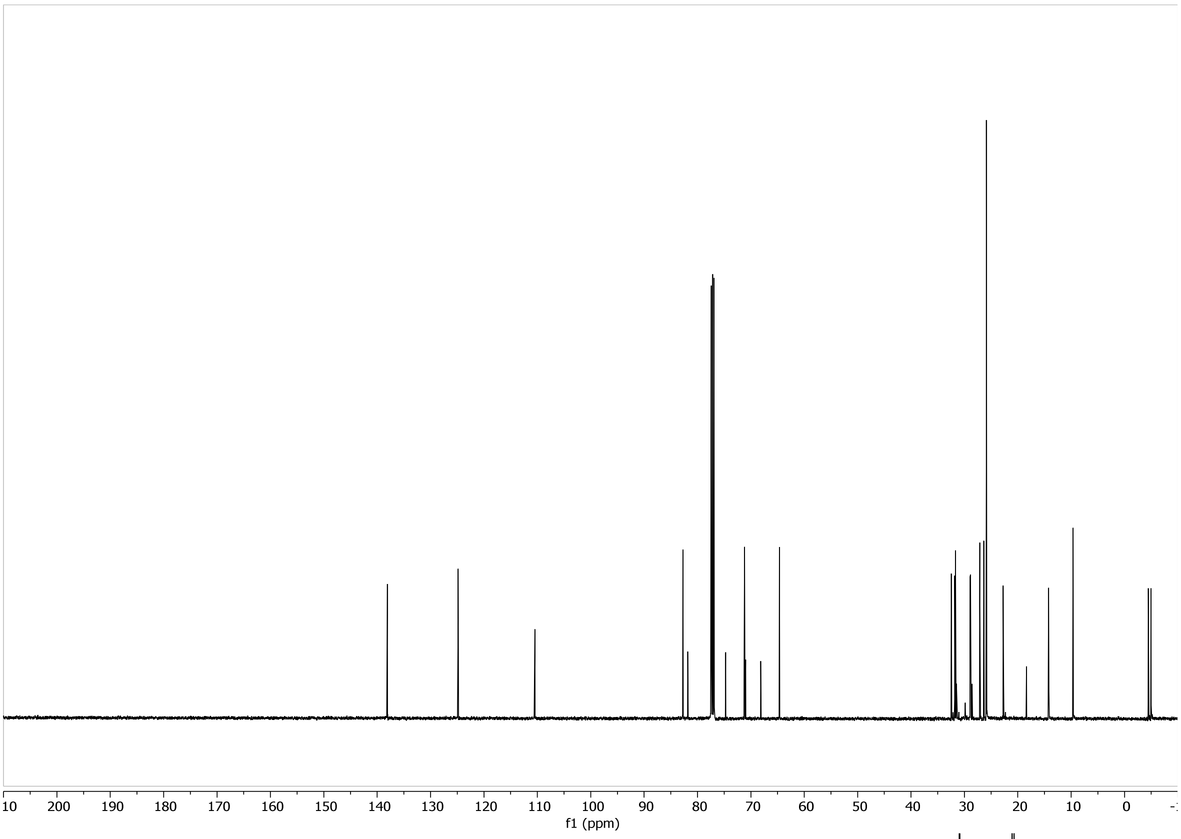

10
200
190
180
170
160
150
140
130
120
110
100
90
80
70
60
50
40
30
20
10
0
f1 (ppm)

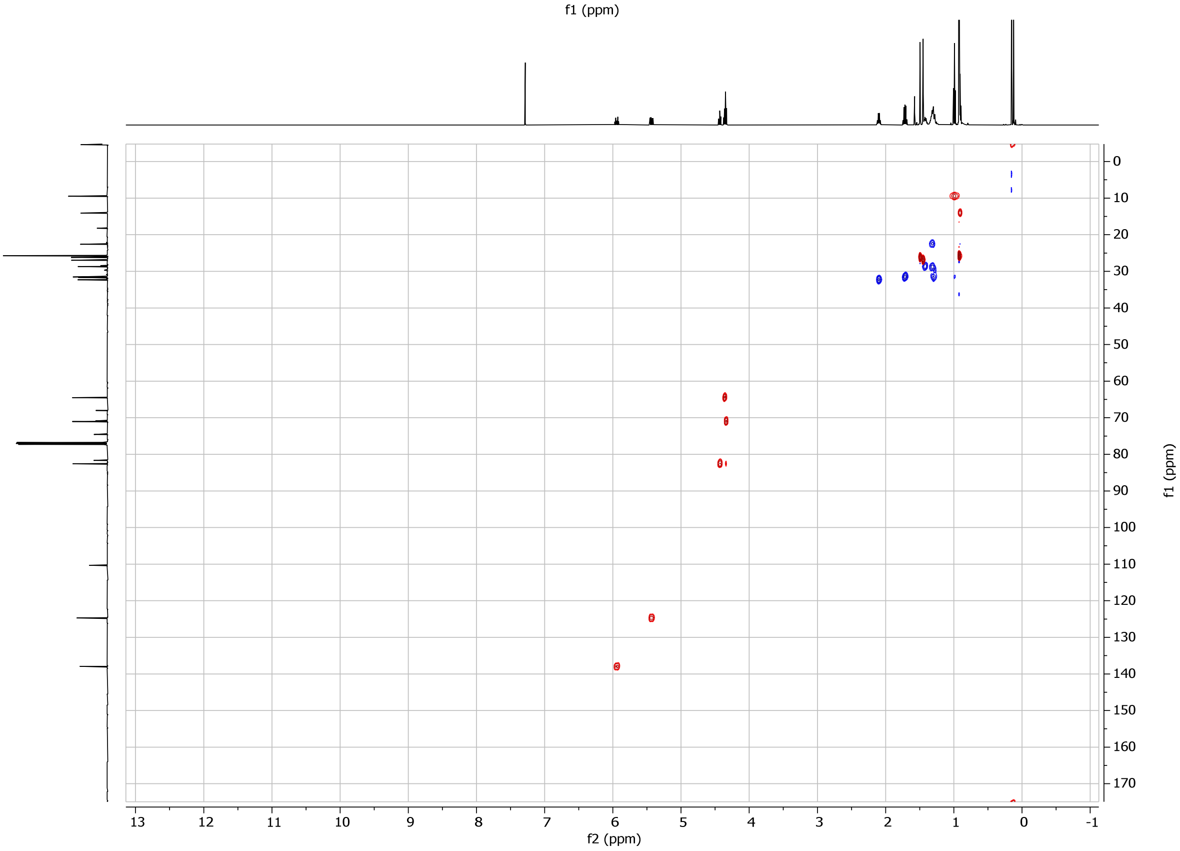

13
12
11
10
9
8
7
6
5
4
3
2
1
0
-1
f2 (ppm)
f1 (ppm)

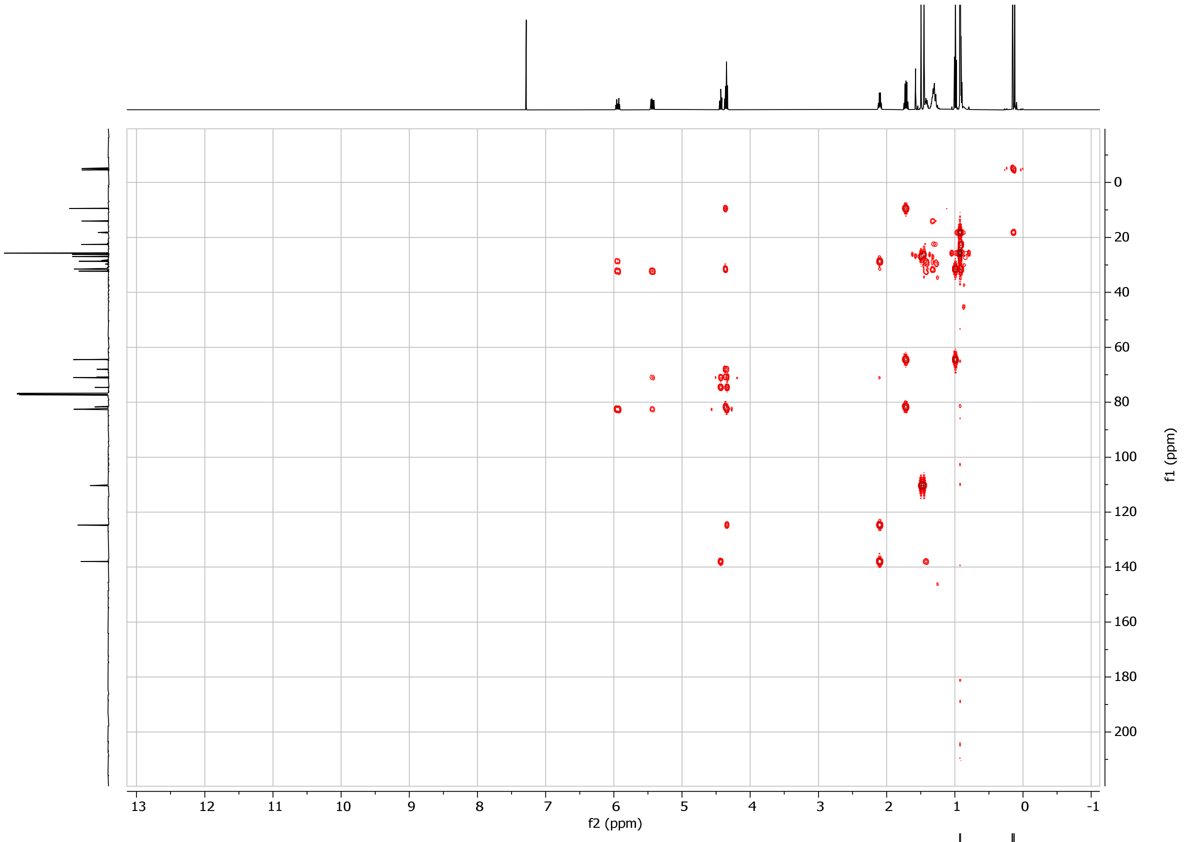
f2 (ppm)
f1 (ppm)

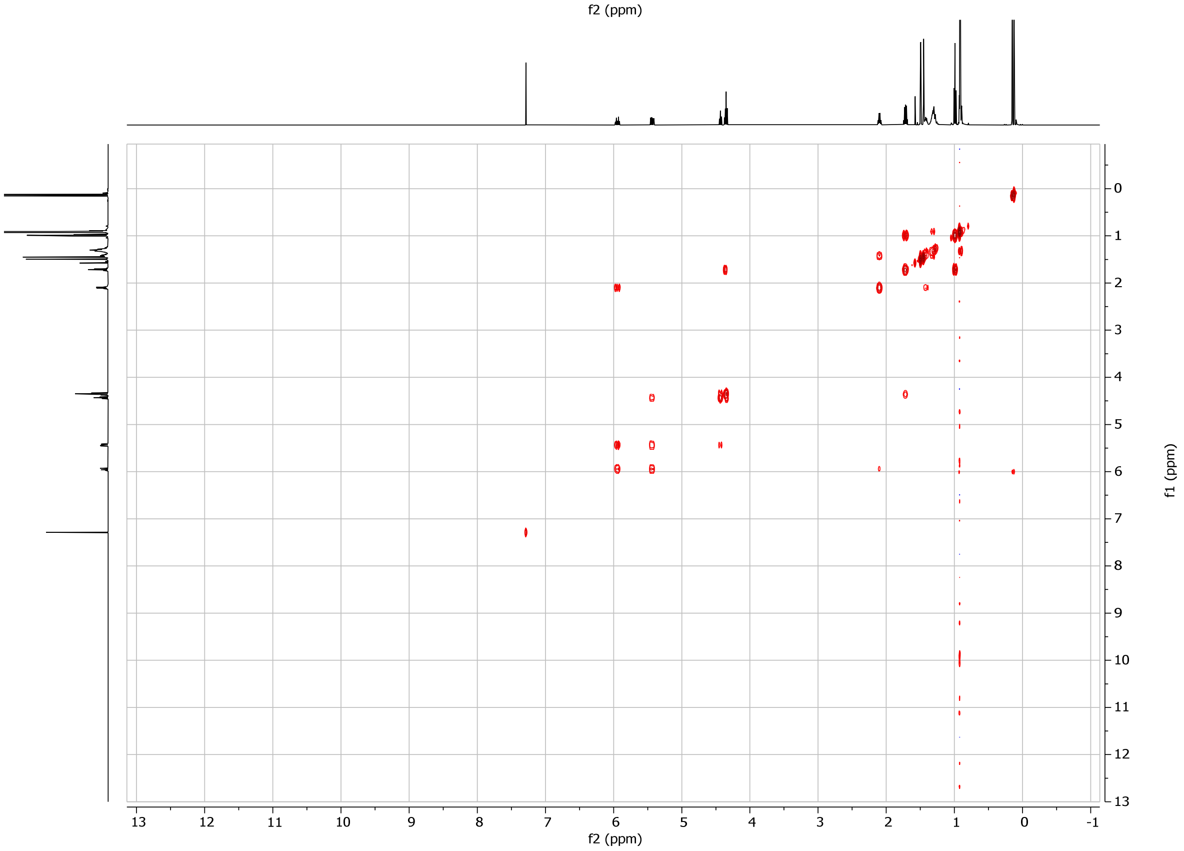
f2 (ppm)
f1 (ppm)

Synthesis of 1b and *ent*-1b (^{1}H, ^{13}C, HSQC, HMBC, COSY spectra)

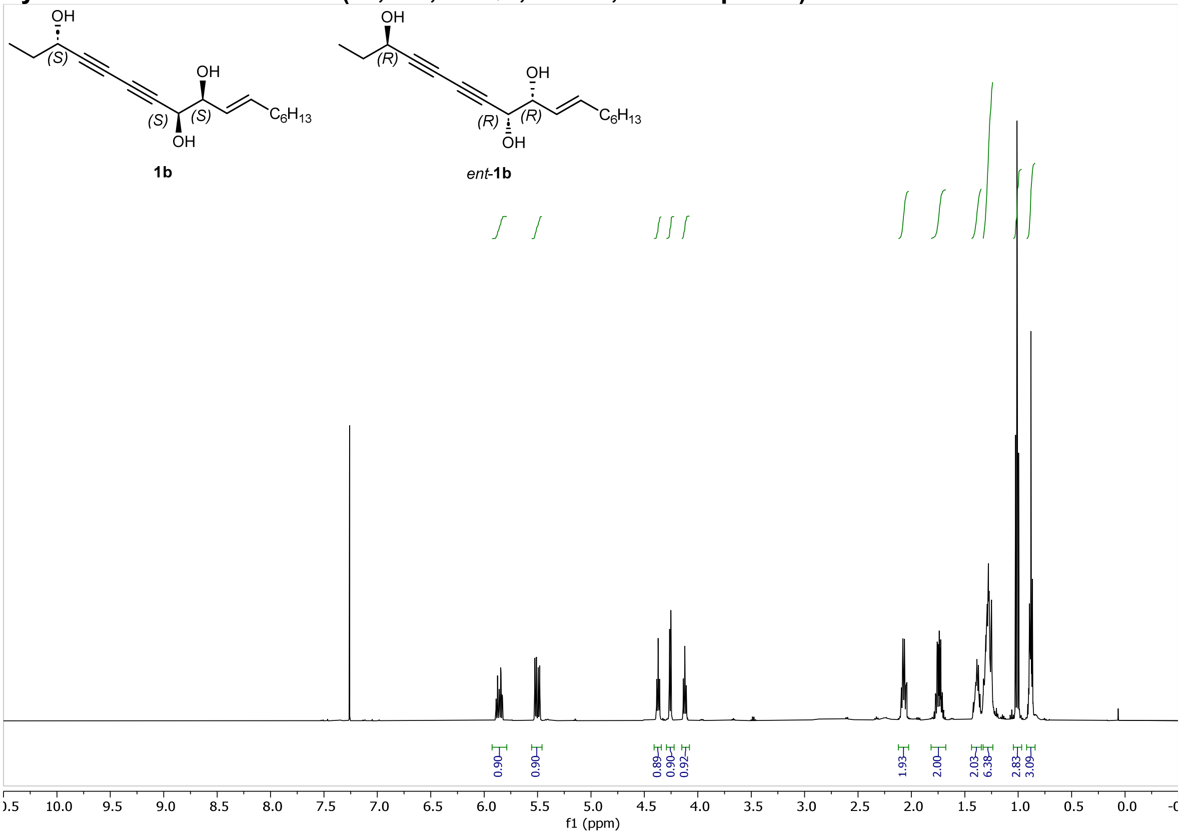

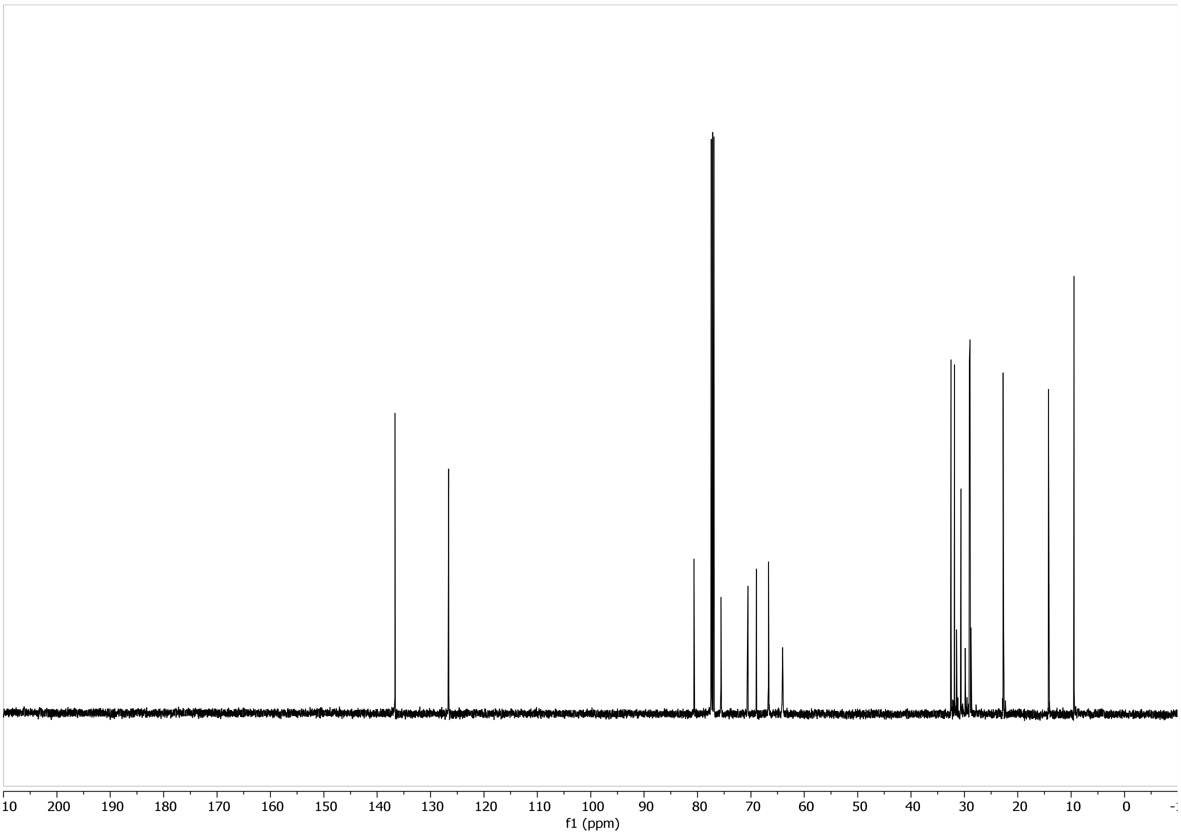

10
200
190
180
170
160
150
140
130
120
110
100
90
80
70
60
50
40
30
20
10
0
f1 (ppm)

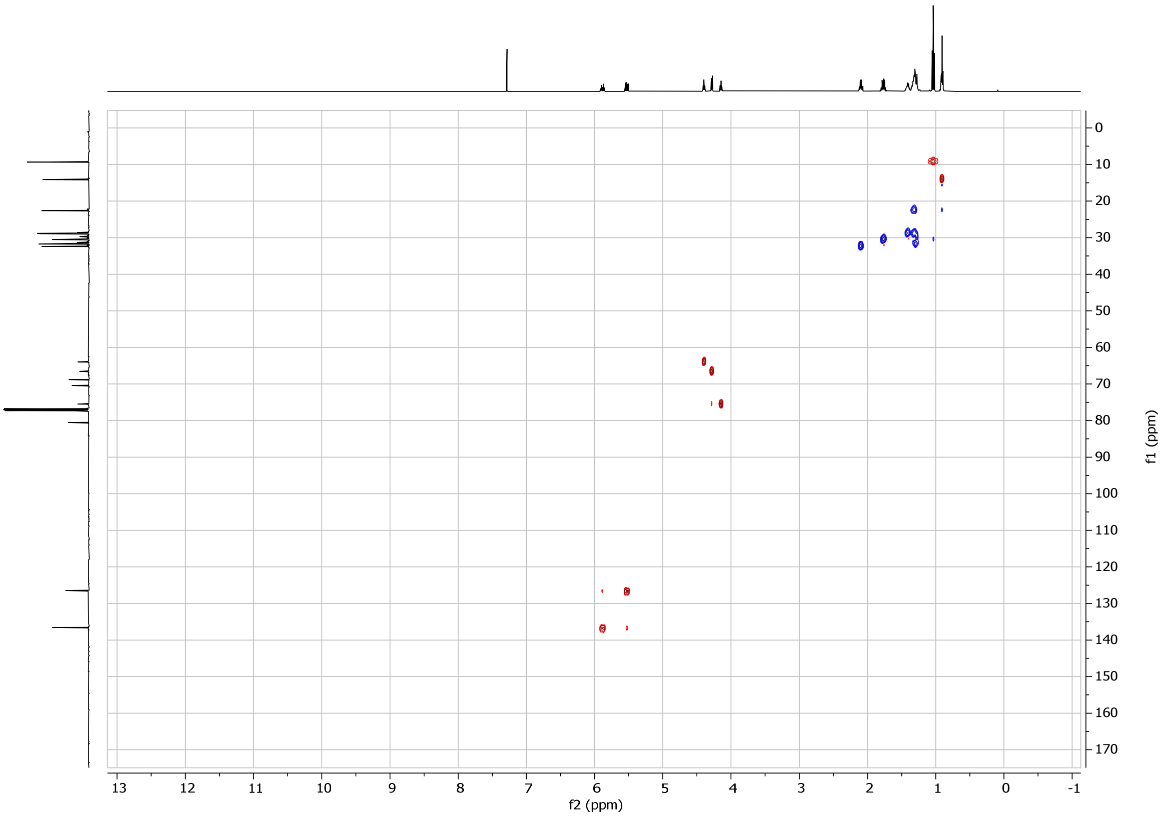

13
12
11
10
9
8
7
6
5
4
3
2
1
0
-1
f2 (ppm)
0
10
20
30
40
50
60
70
80
90
100
110
120
130
140
150
160
170
f1 (ppm)

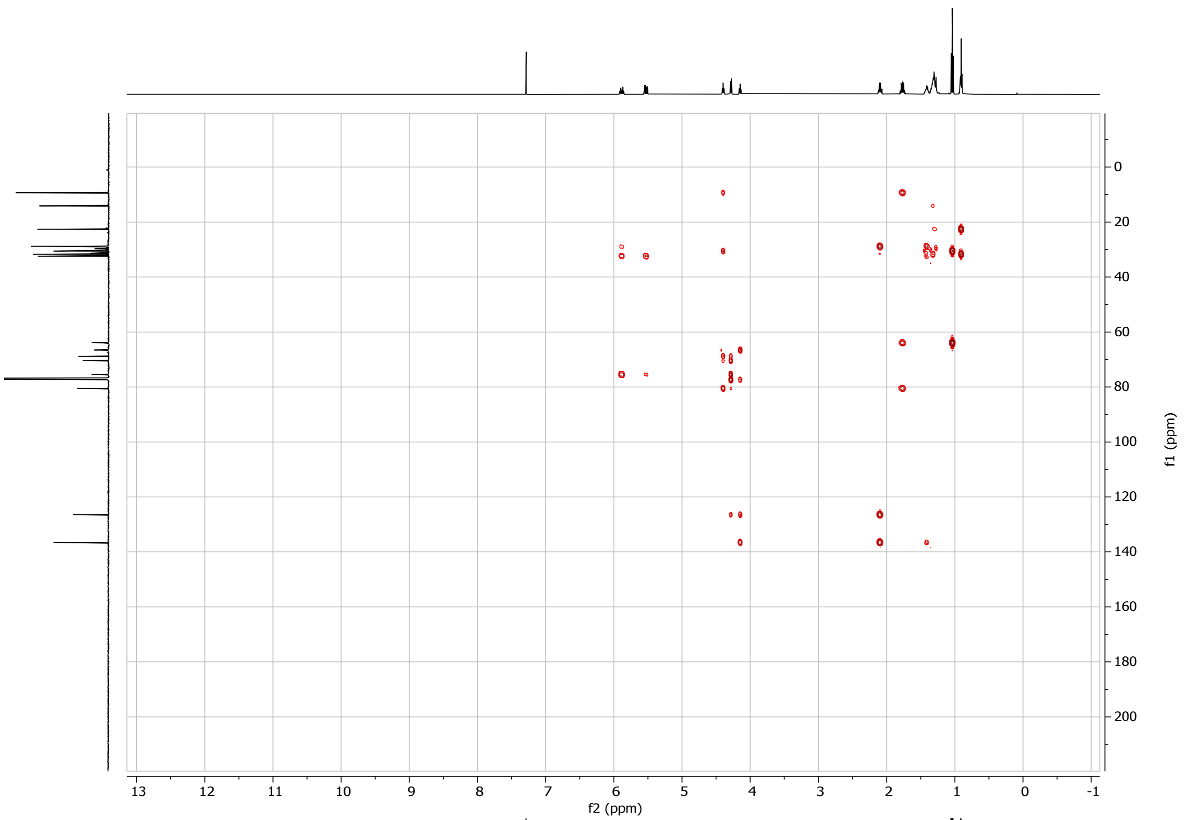
f2 (ppm)
f1 (ppm)

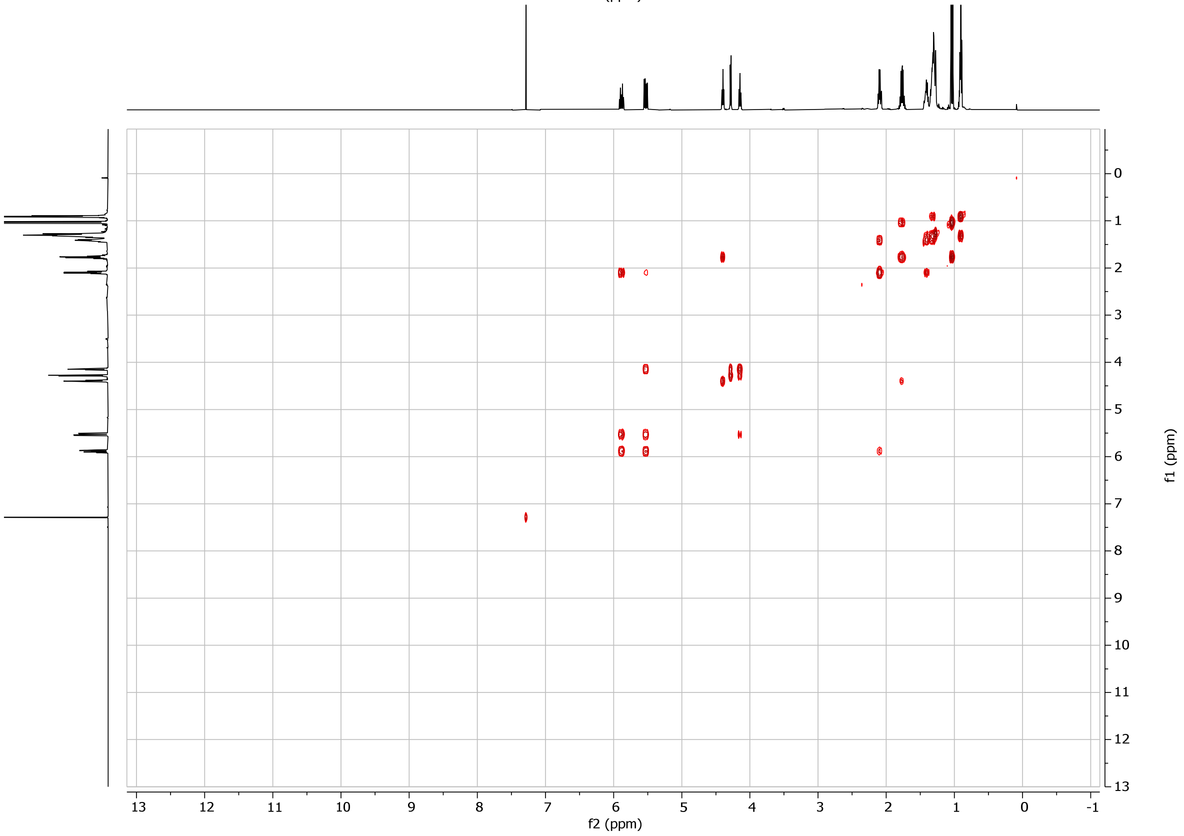
f2 (ppm)
f1 (ppm)

Synthesis of S15c and *ent*-S15c (^{1}H, ^{13}C, HSQC, HMBC, COSY spectra)

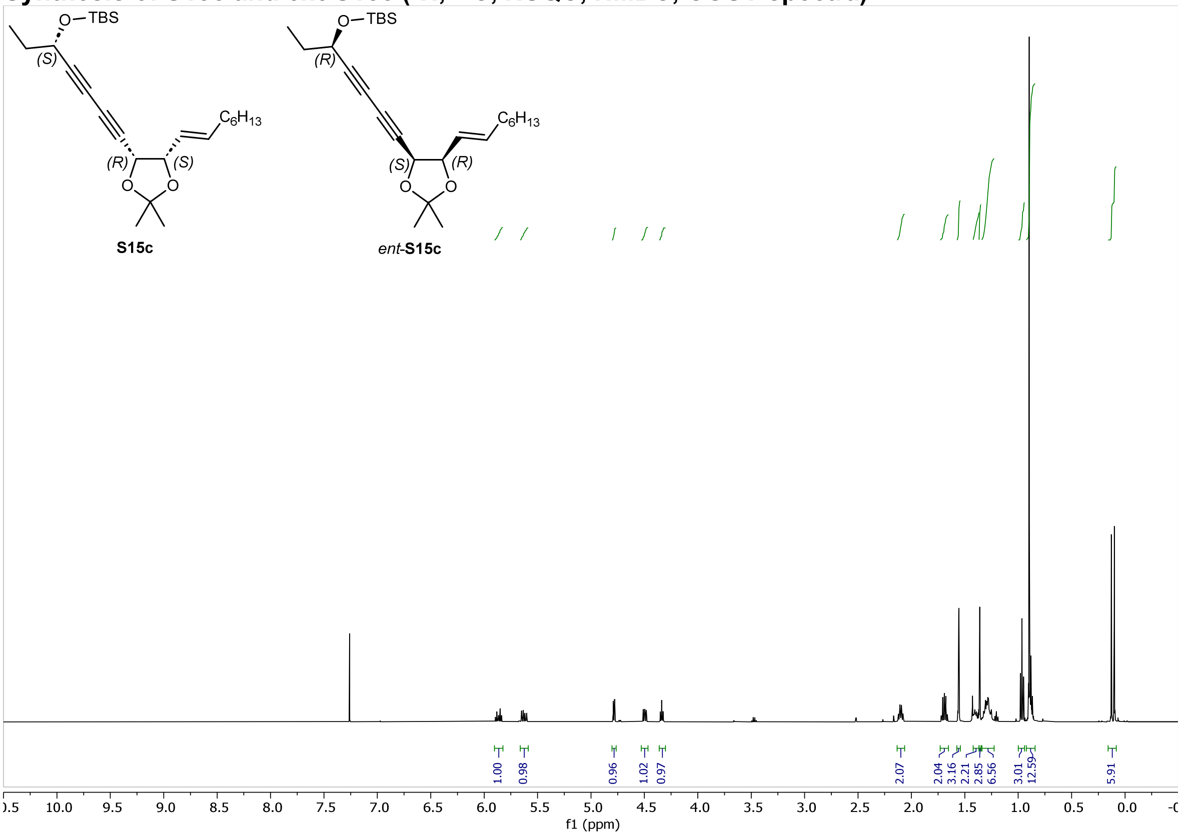

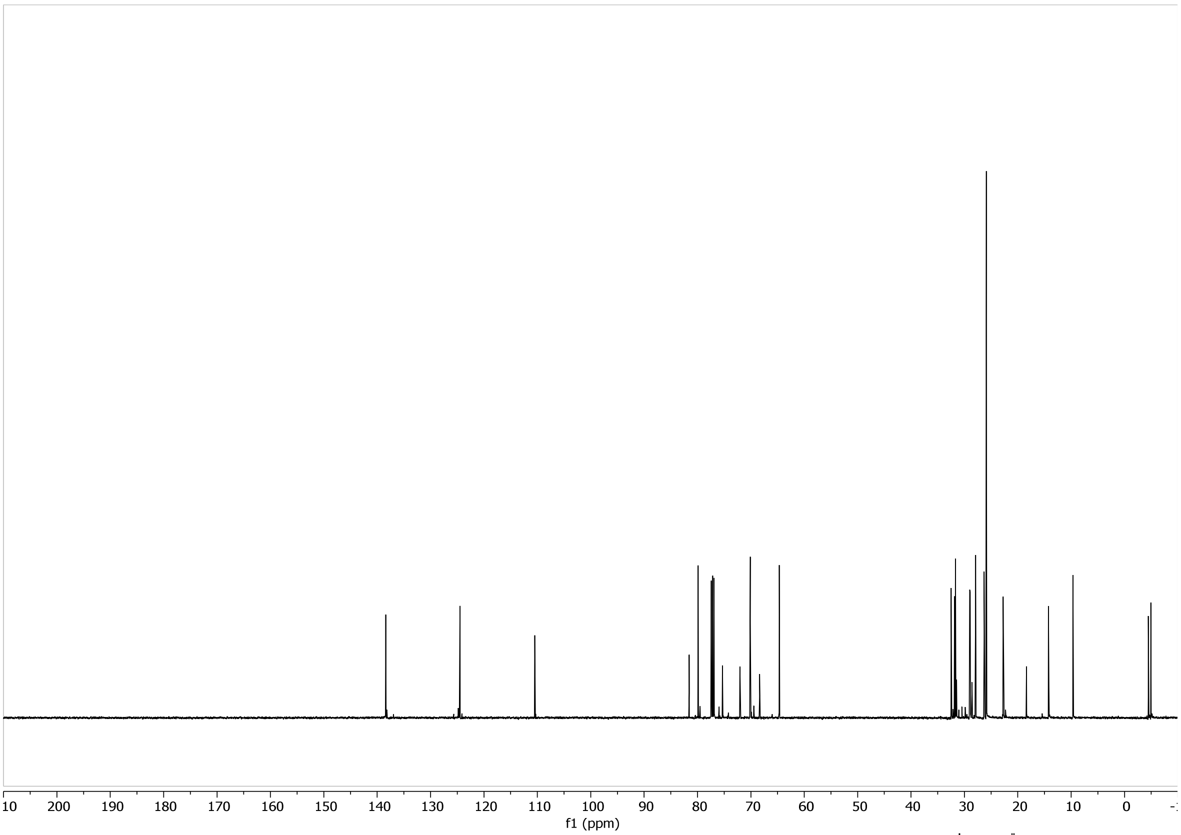
f1 (ppm)

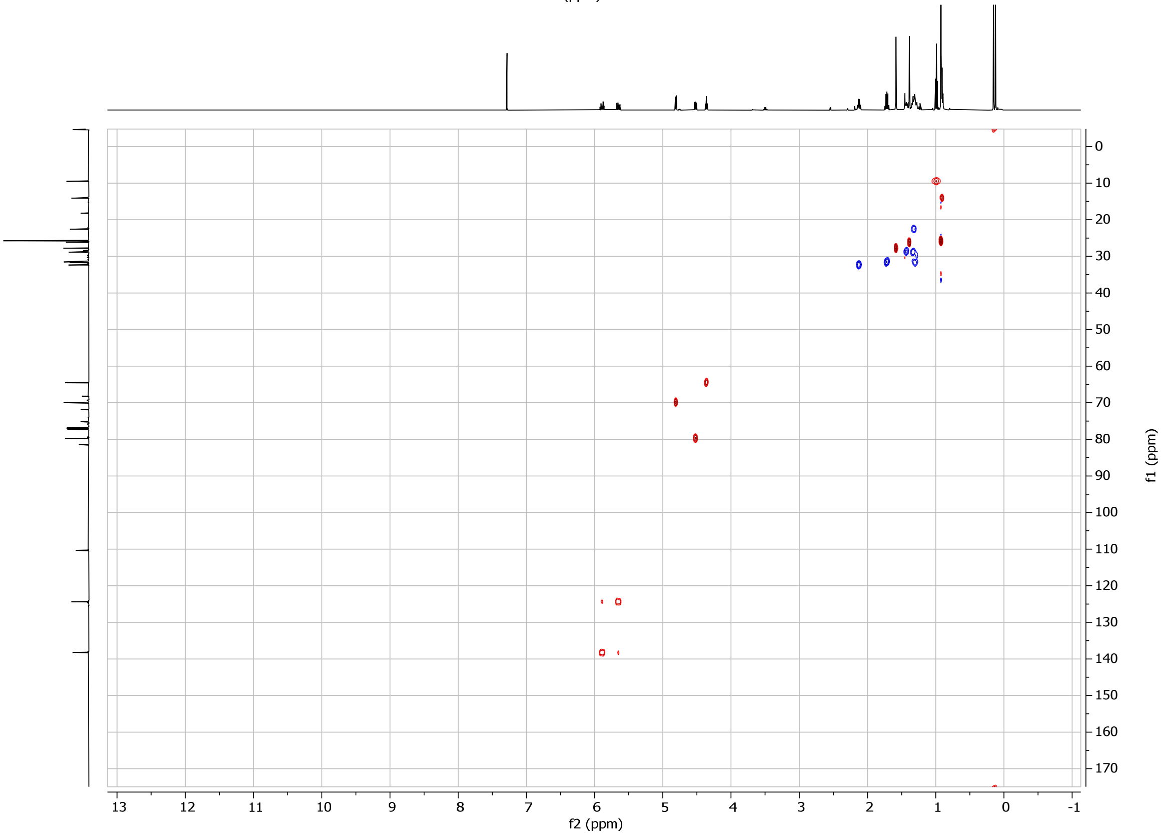
f2 (ppm)
f1 (ppm)

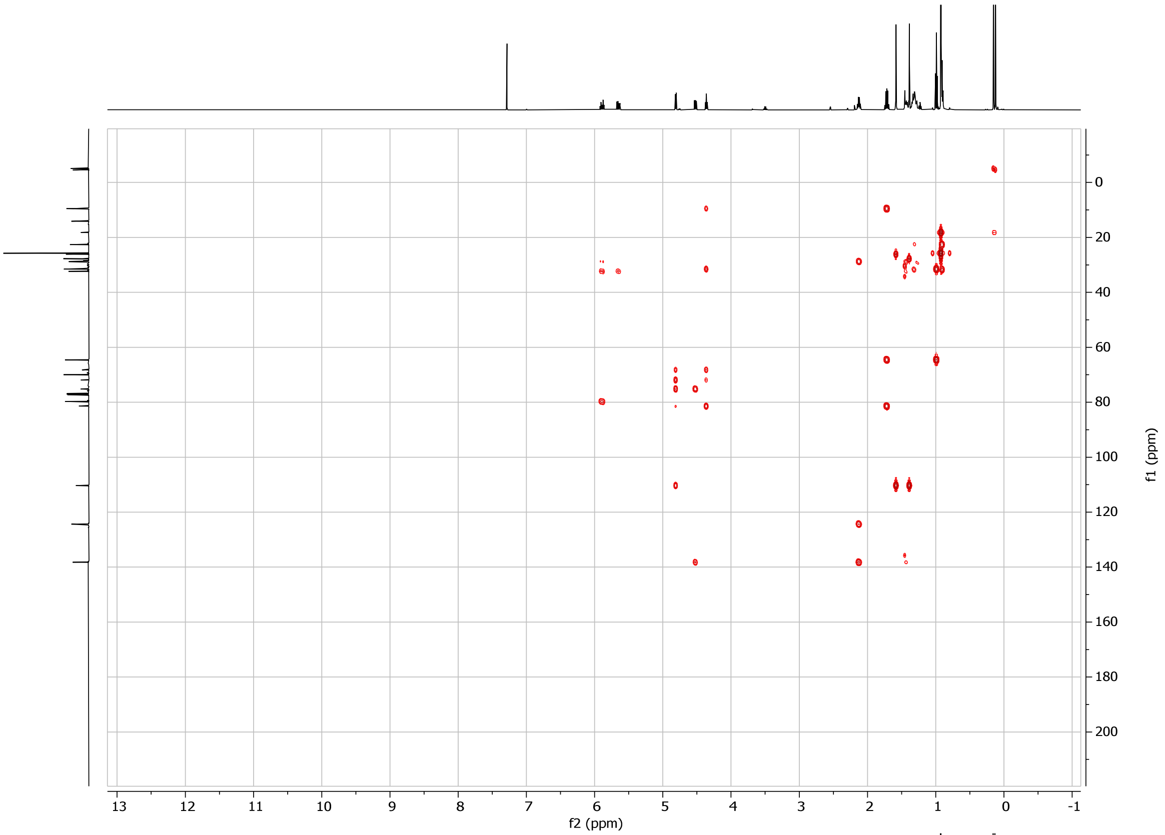

f2 (ppm)
f1 (ppm)

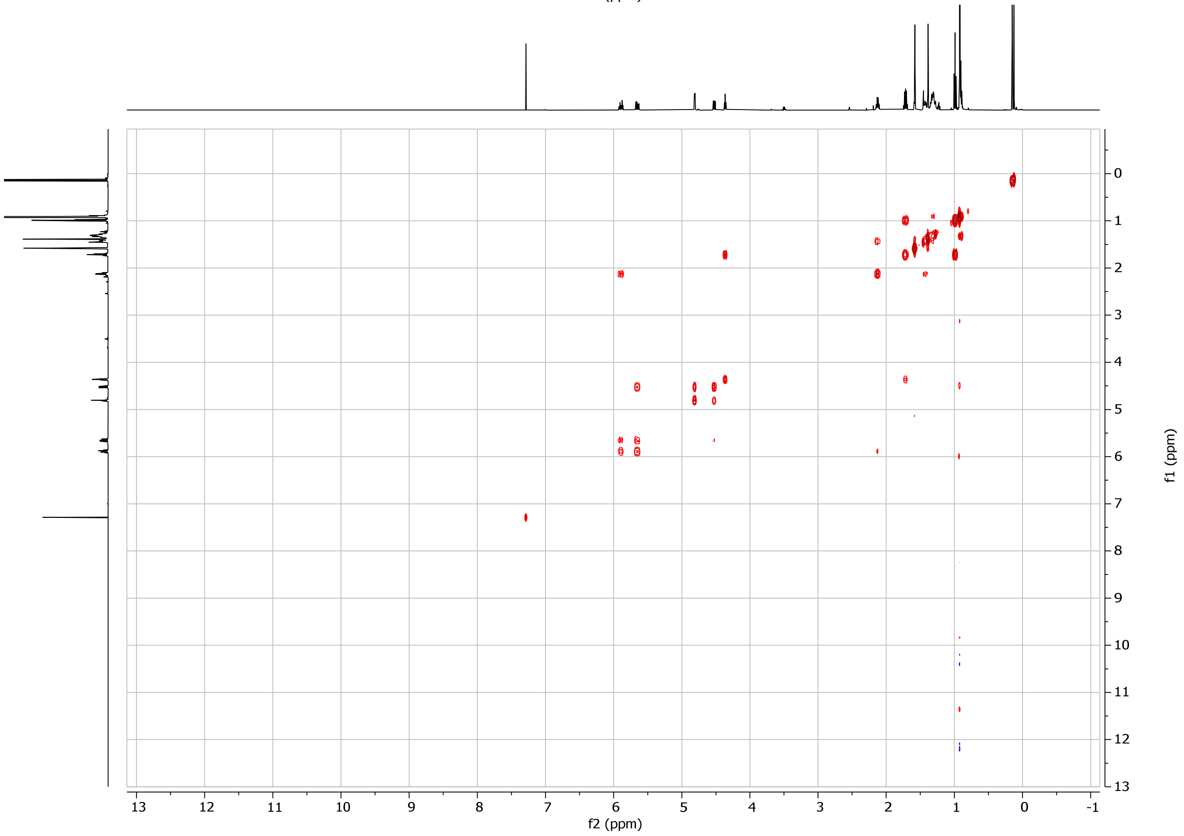

f2 (ppm)
f1 (ppm)

Synthesis of 1c and *ent*-1c (^{1}H, ^{13}C, HSQC, HMBC, COSY spectra)

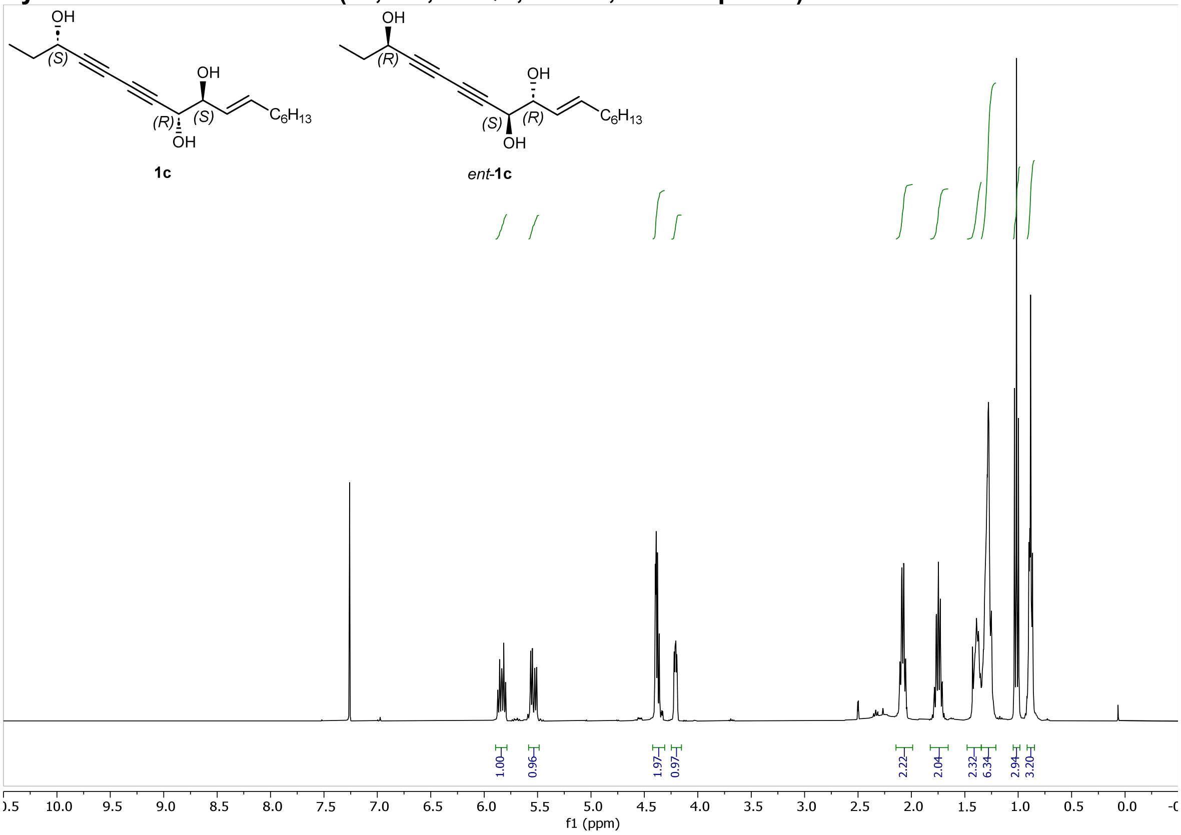

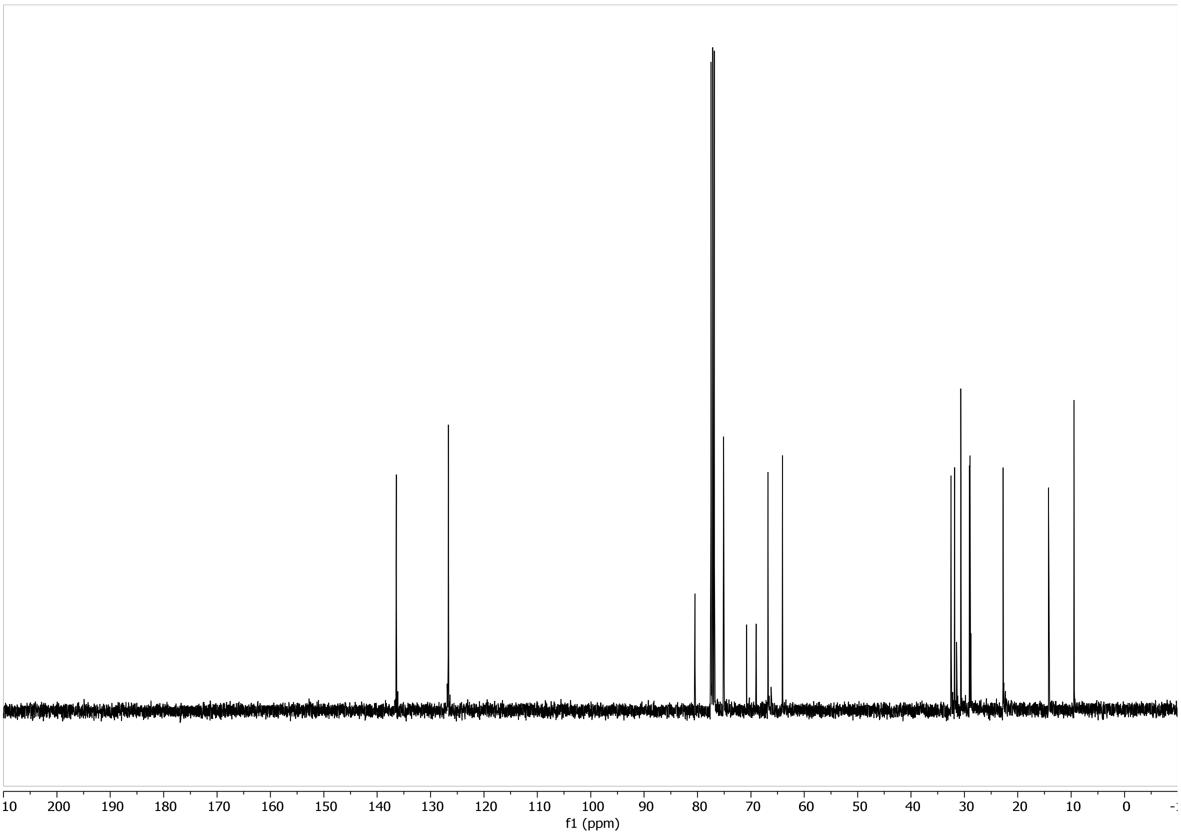

10
200
190
180
170
160
150
140
130
120
110
100
90
80
70
60
50
40
30
20
10
0
f1 (ppm)

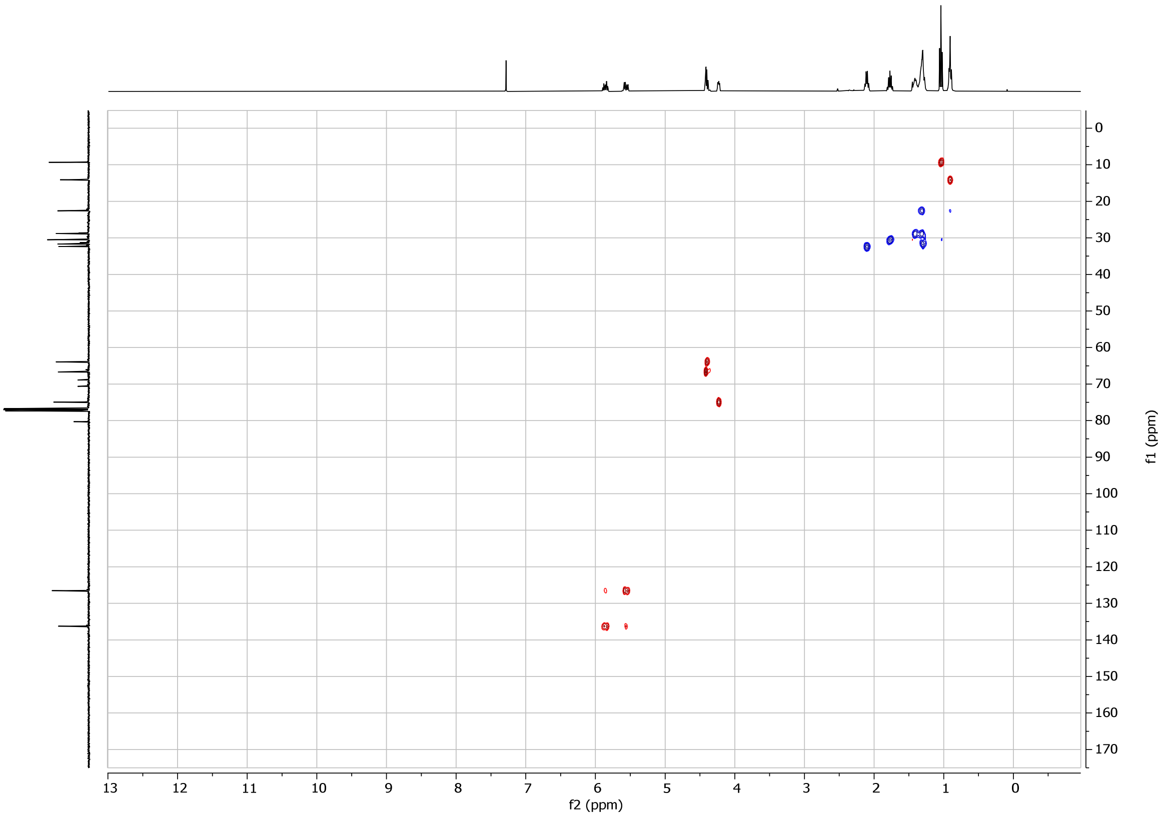

f2 (ppm)
f1 (ppm)

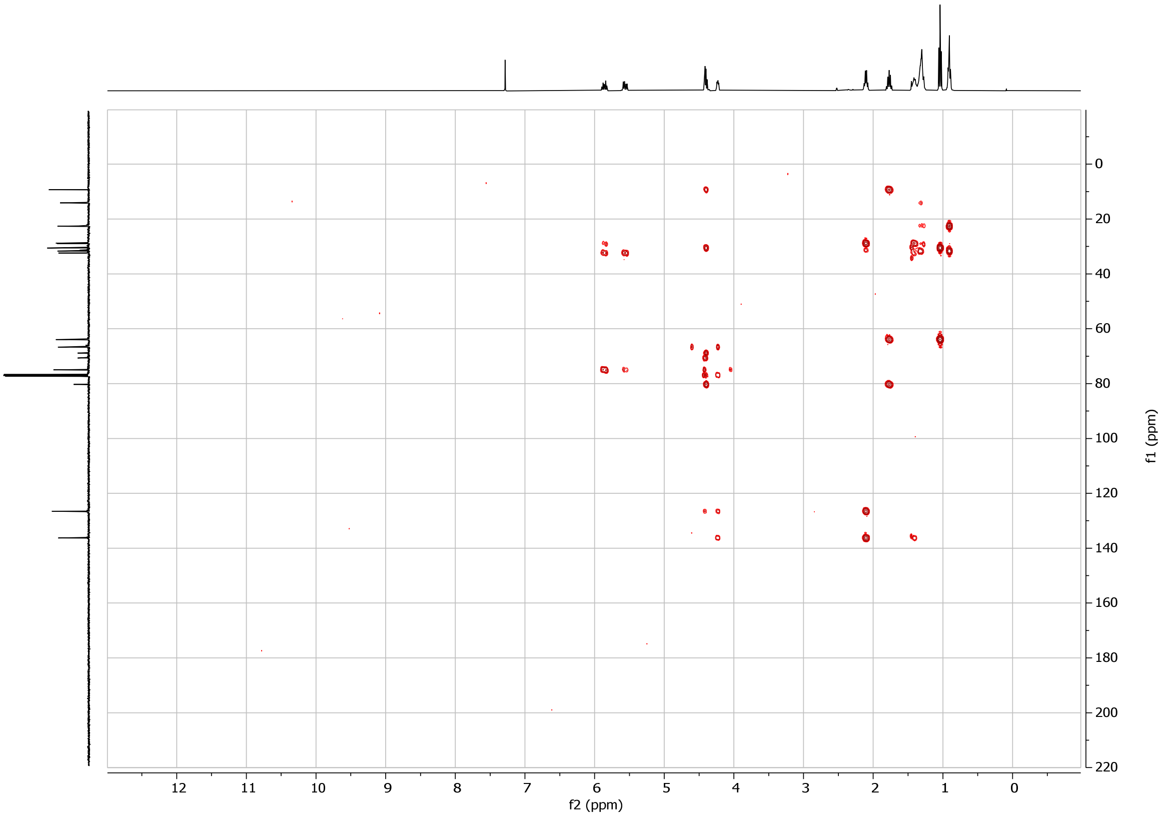

f2 (ppm)
f1 (ppm)

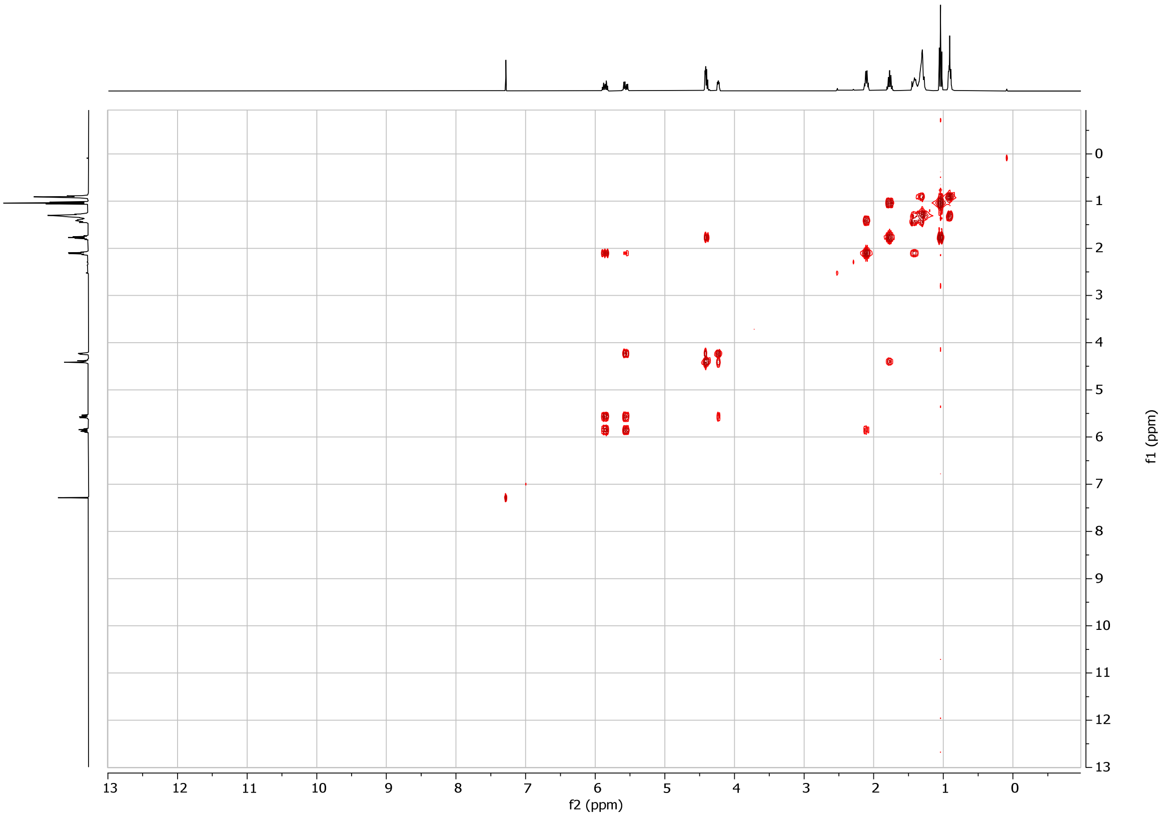

f2 (ppm)
f1 (ppm)

Synthesis of S15d and *ent*-S15d (¹H, ¹³C, HSQC, HMBC, COSY spectra)

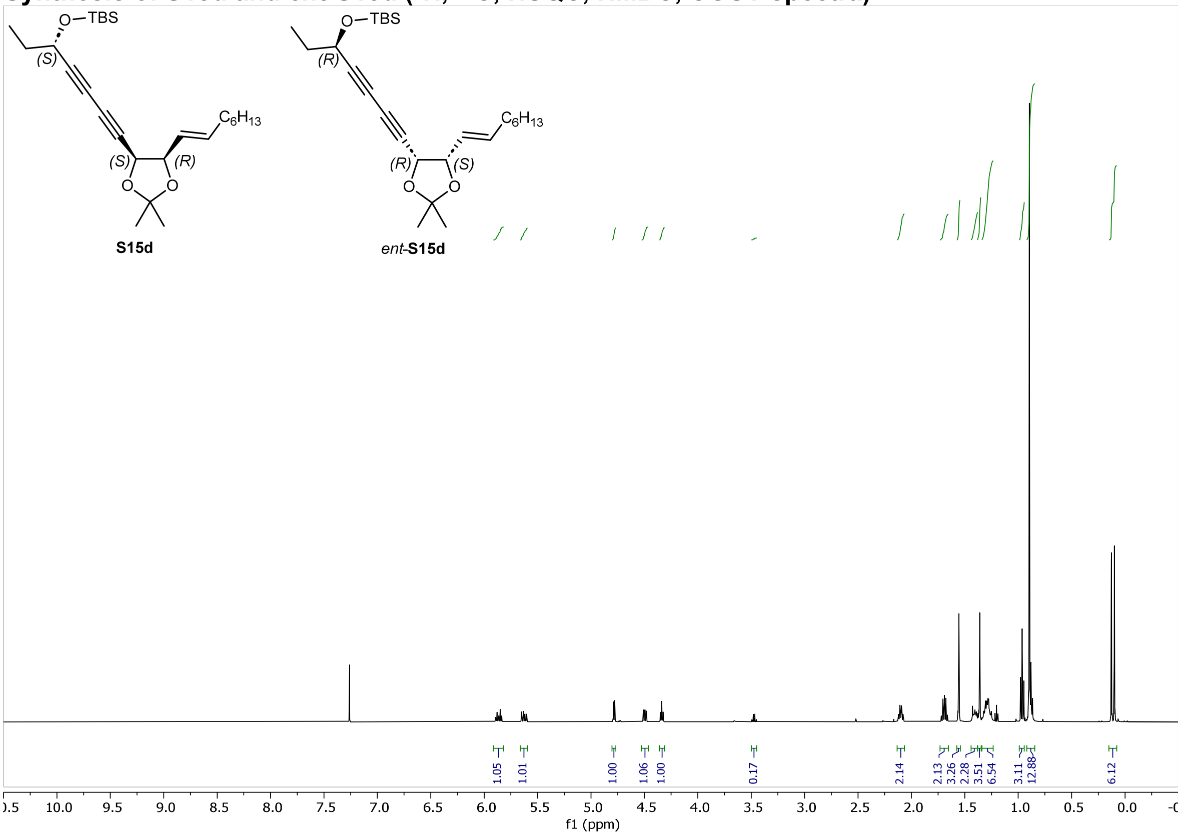

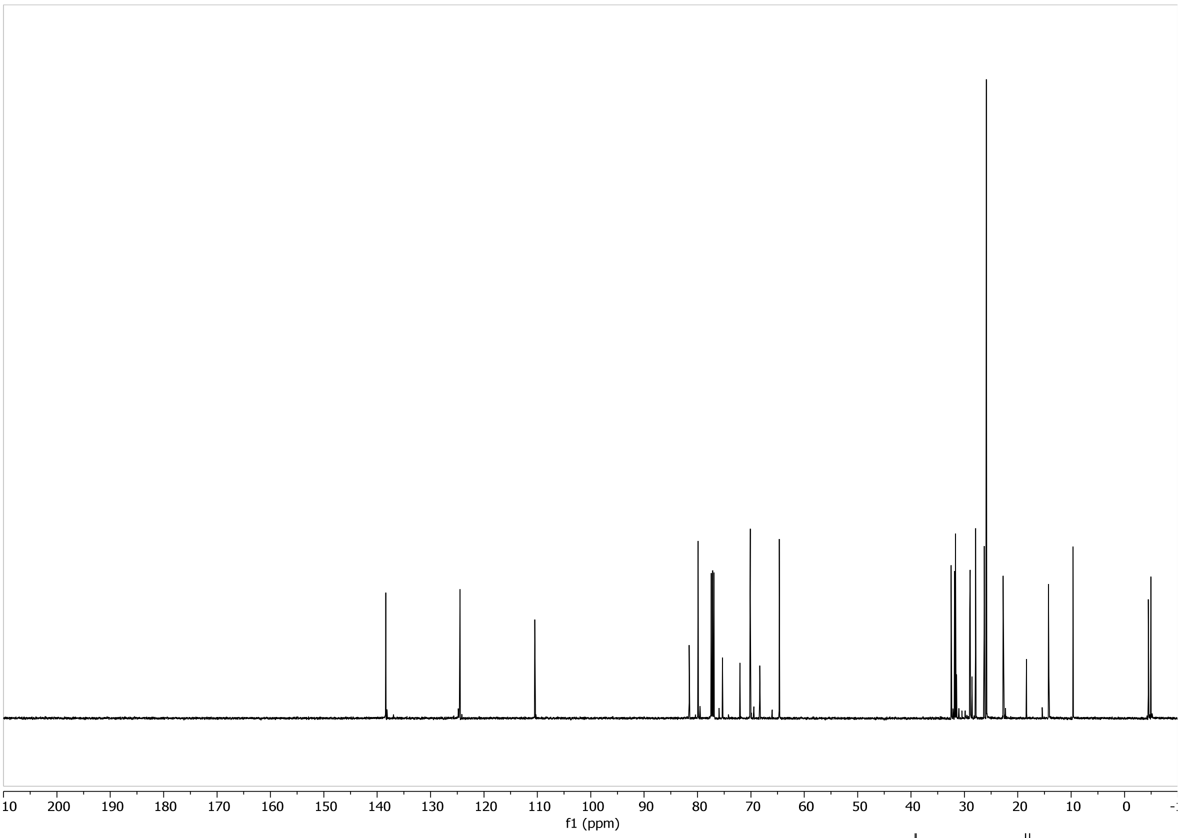
f1 (ppm)

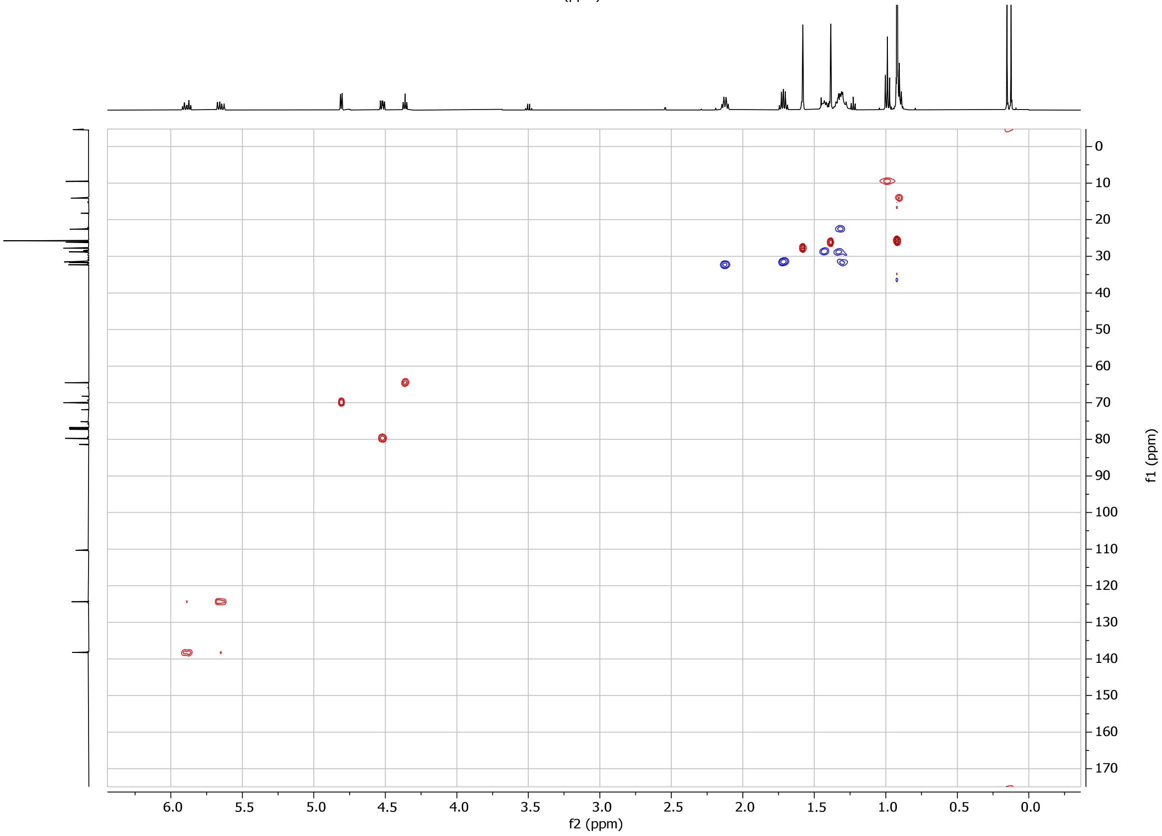
f2 (ppm)
f1 (ppm)

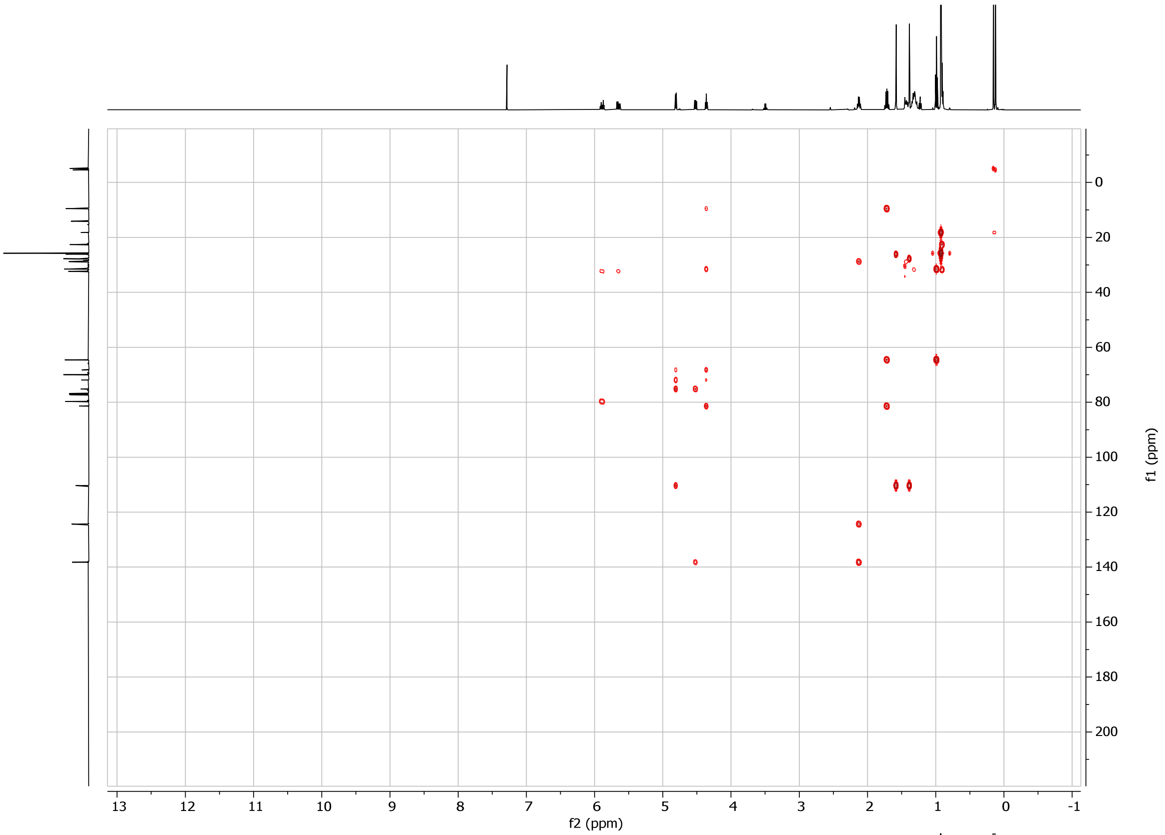
f2 (ppm)
f1 (ppm)

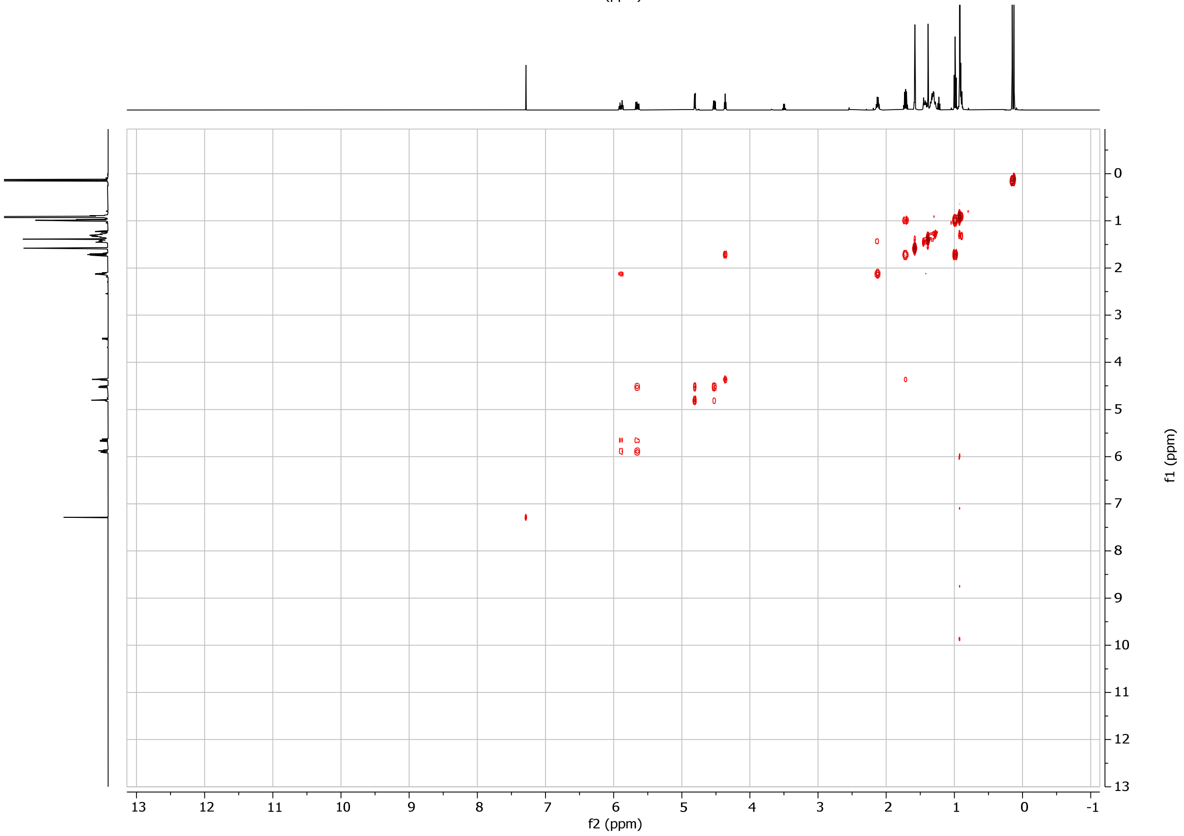
f2 (ppm)
f1 (ppm)

Synthesis of 1d and *ent*-1d (^{1}H, ^{13}C, HSQC, HMBC, COSY spectra)

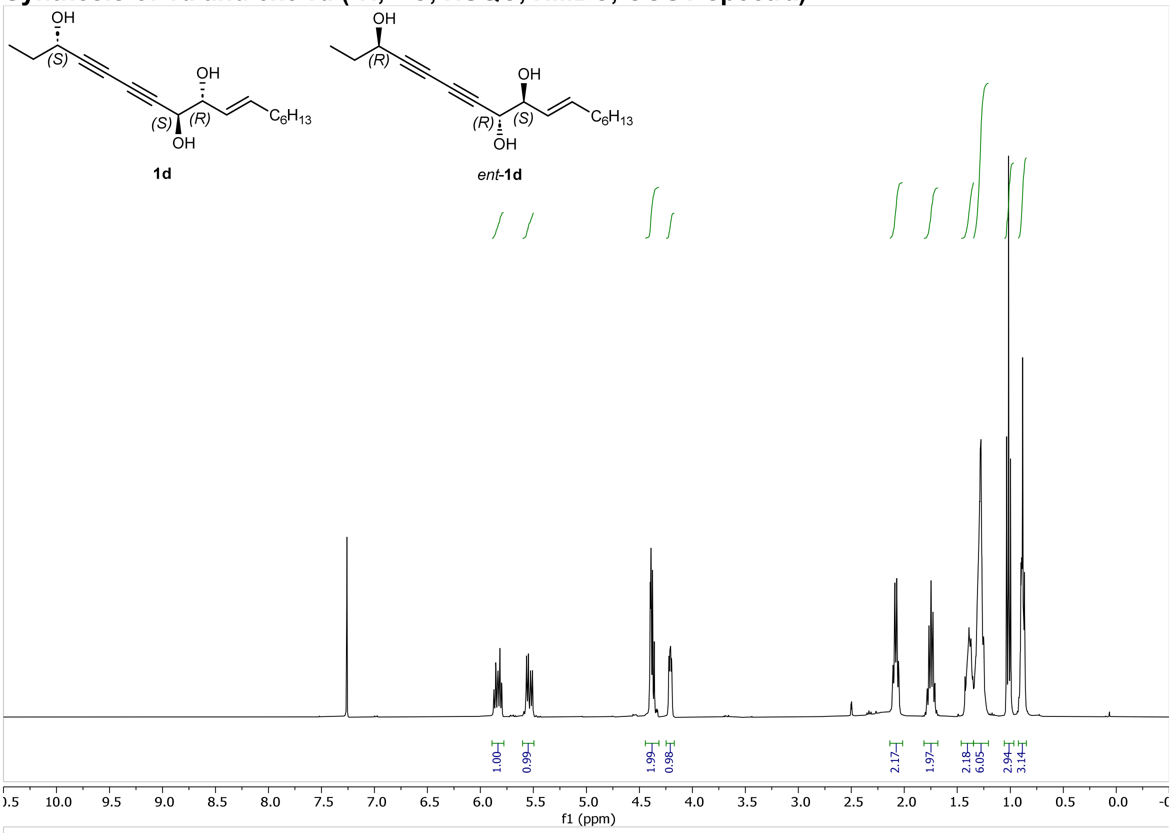

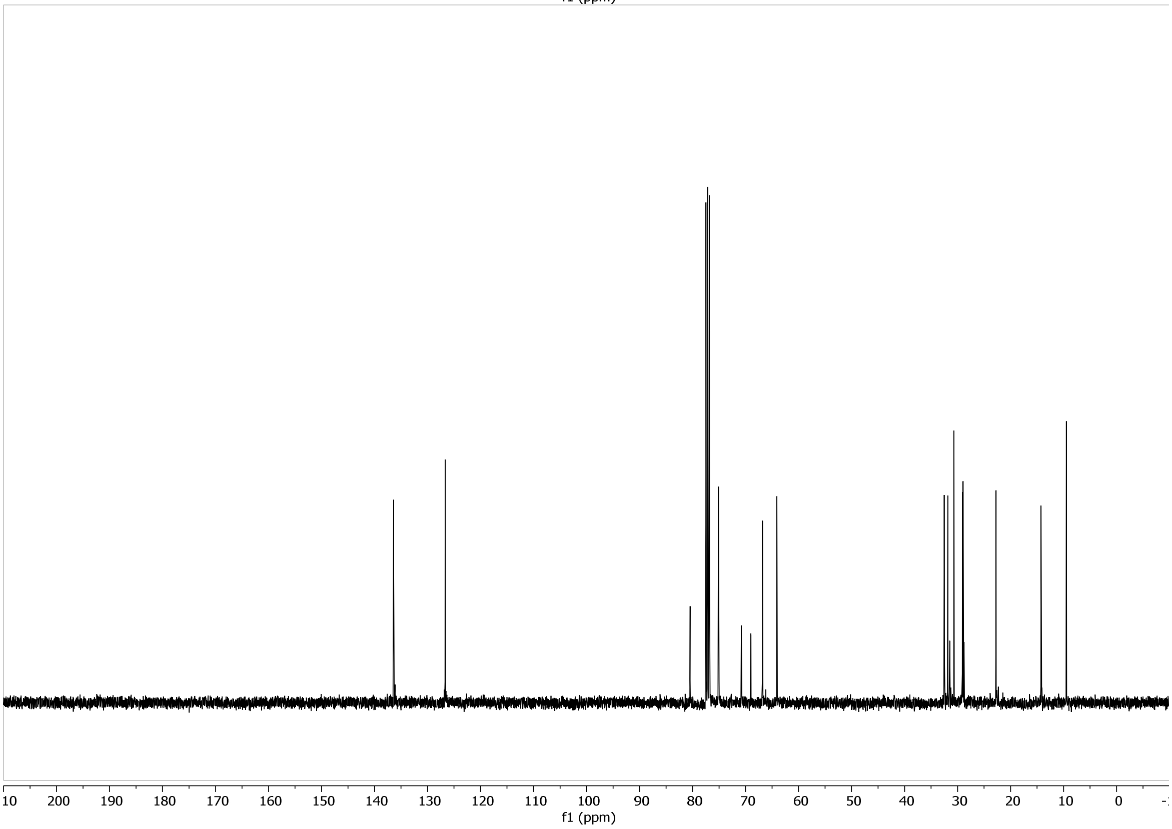

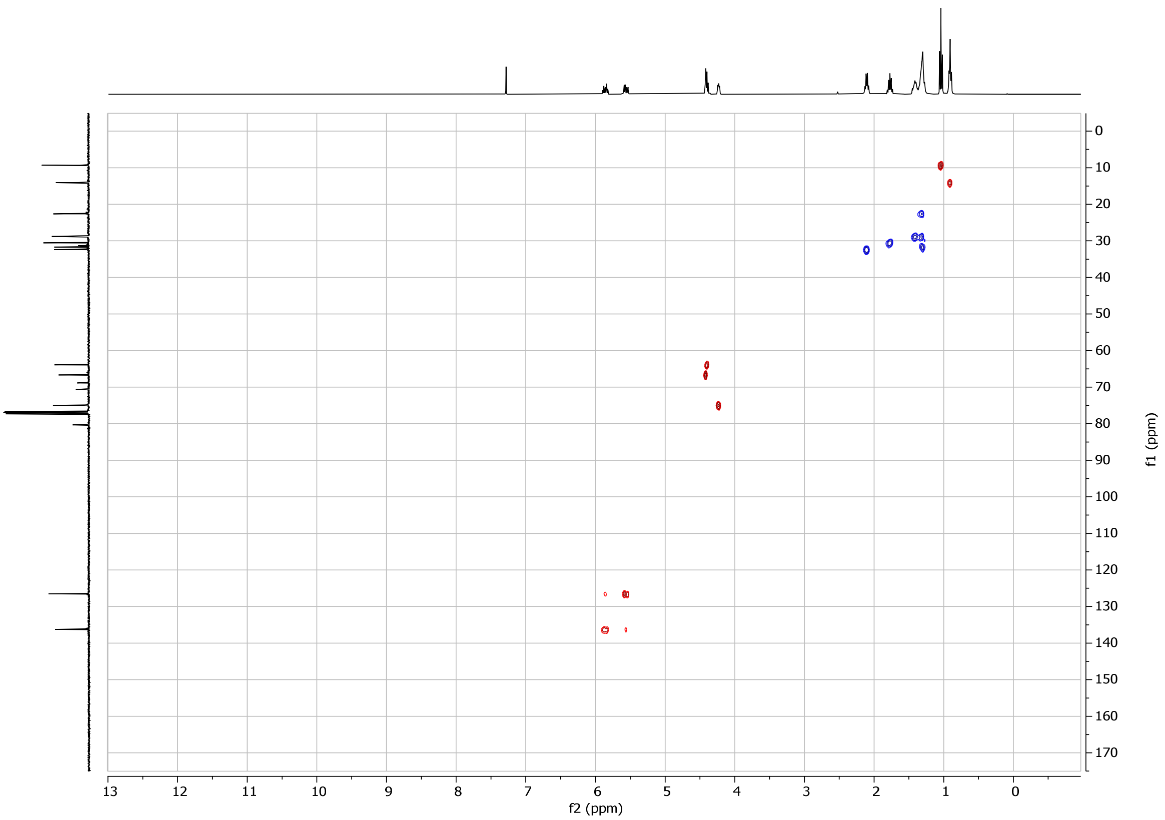
f1 (ppm)
f2 (ppm)

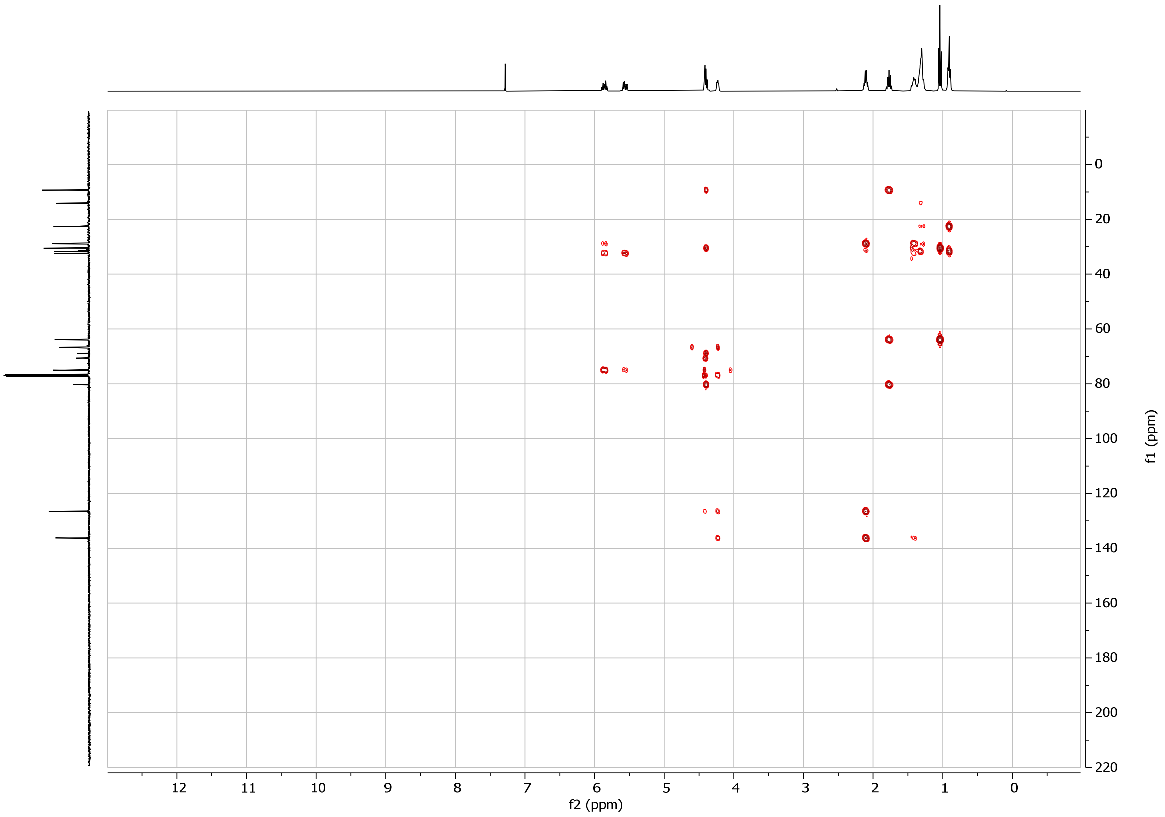
f1 (ppm)
f2 (ppm)

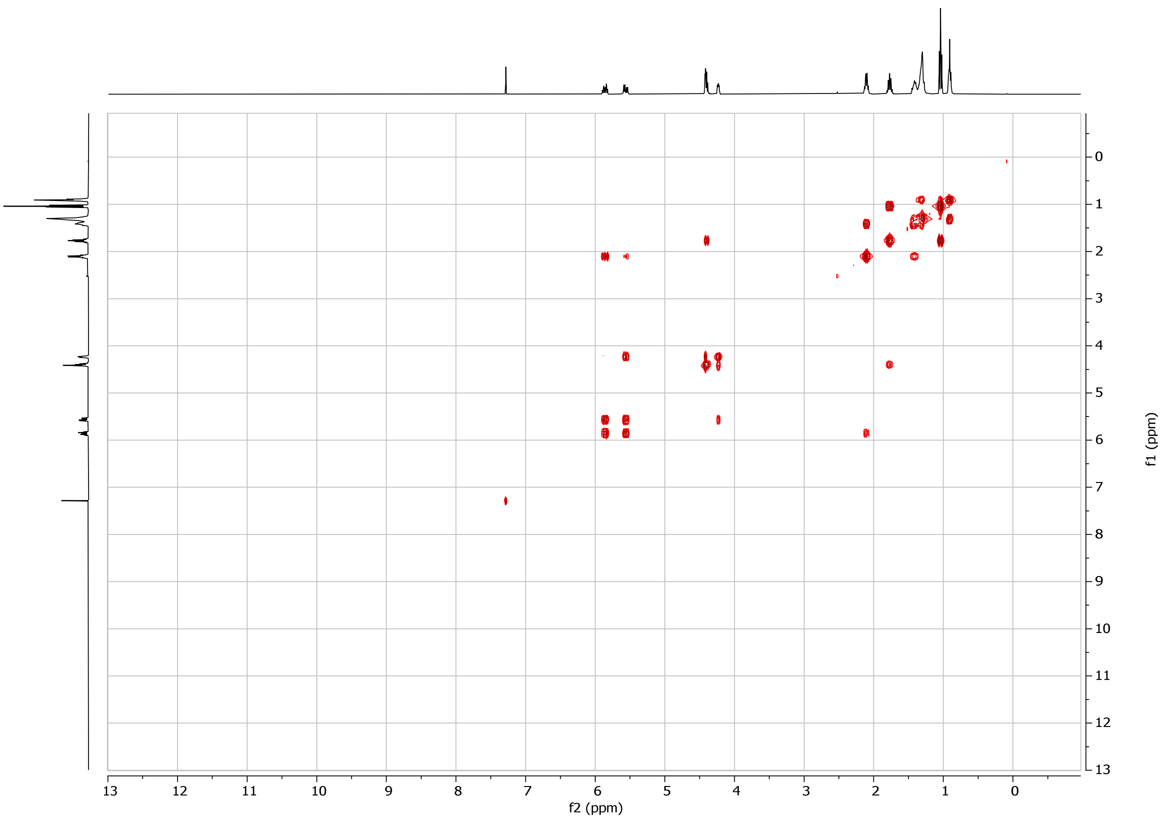

f2 (ppm)
f1 (ppm)

9.2 Spectra to Configurational Assignment of Isofalcarintriol

(^{1}H, ^{13}C, HSQC, HMBC, COSY spectra)

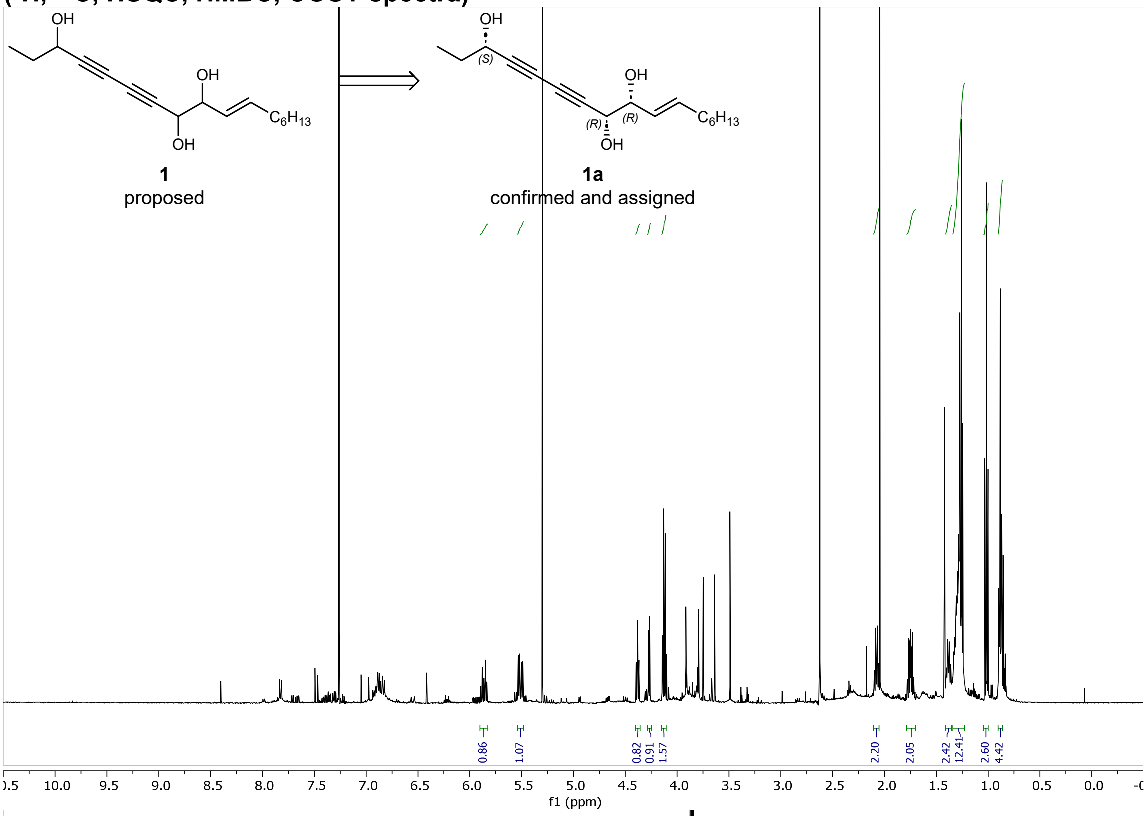

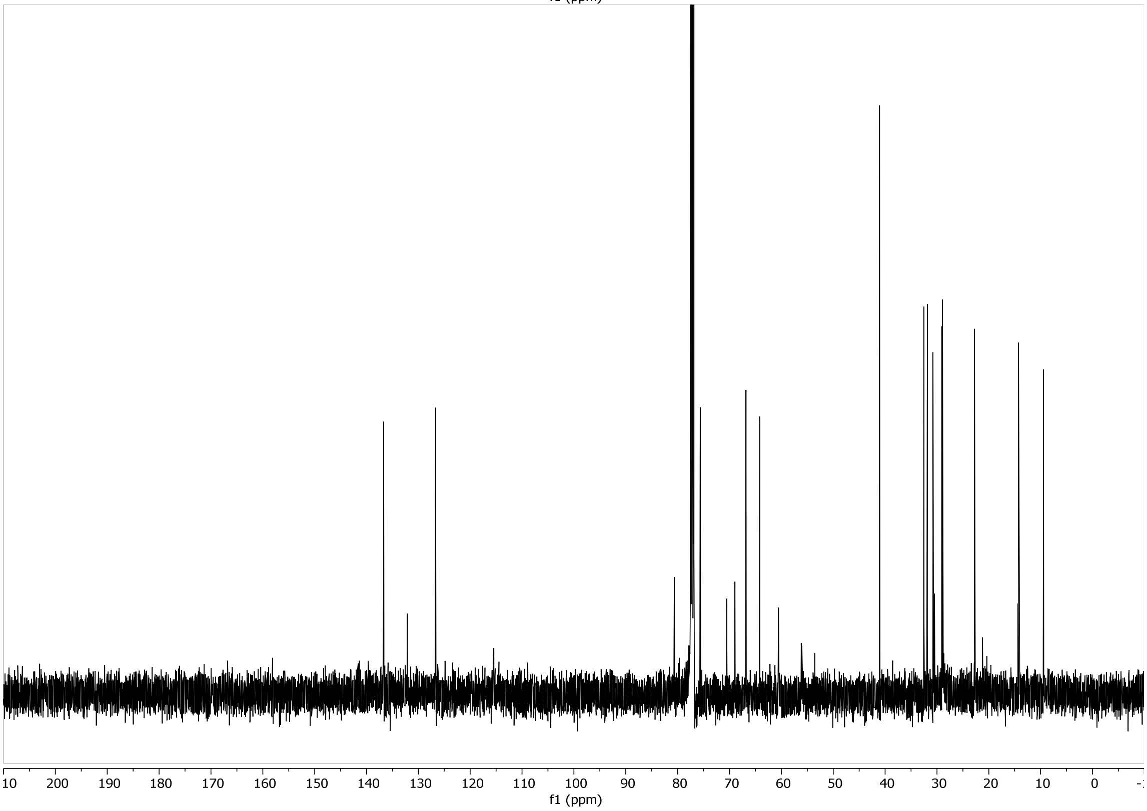

600 MHz

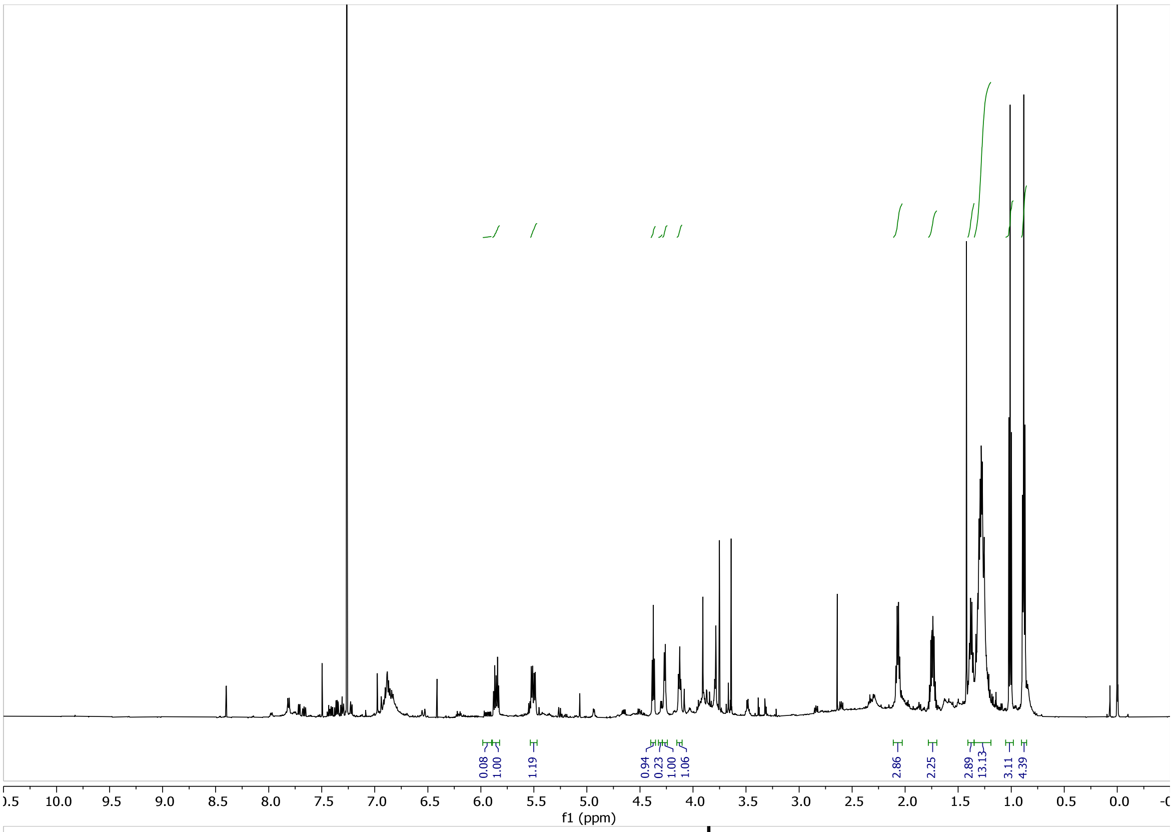

0.08
1.00
1.19
0.94
0.23
1.00
1.06
2.86
2.25
2.89
13.13
3.11
4.39
f1 (ppm)

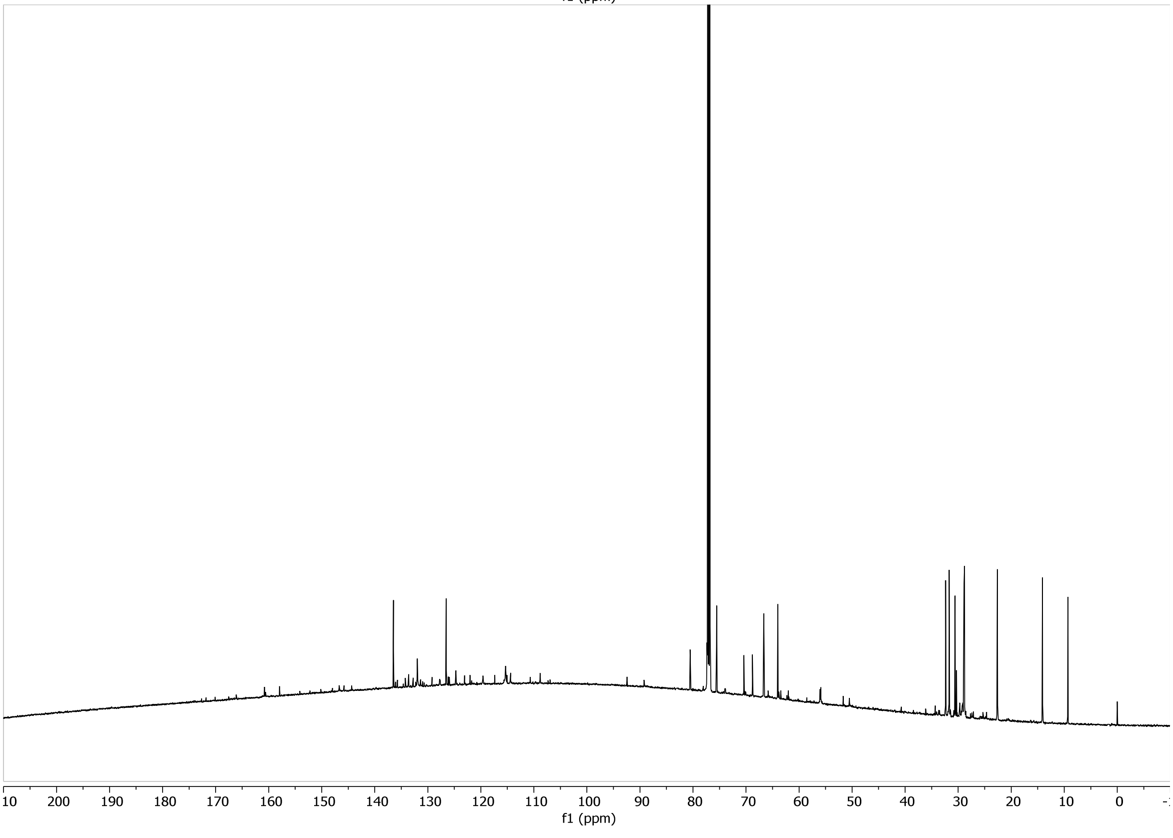

f1 (ppm)

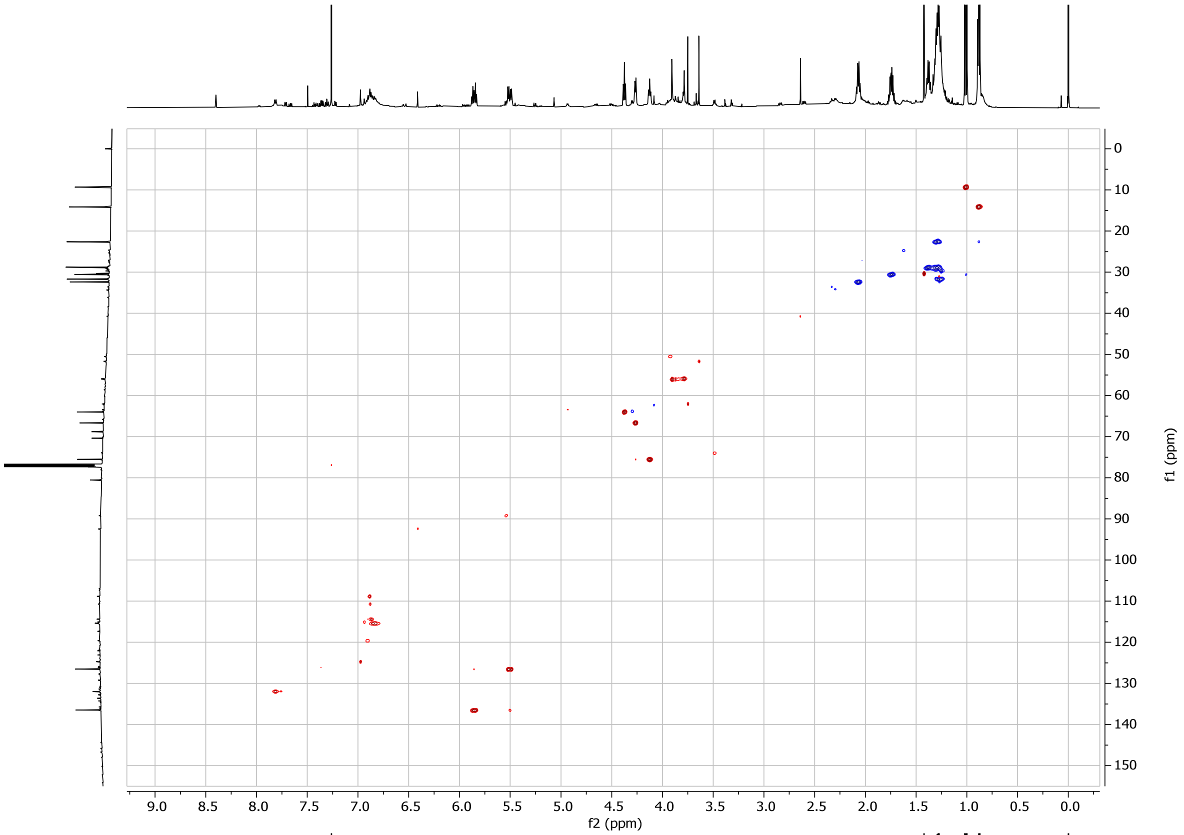

f2 (ppm)
f1 (ppm)

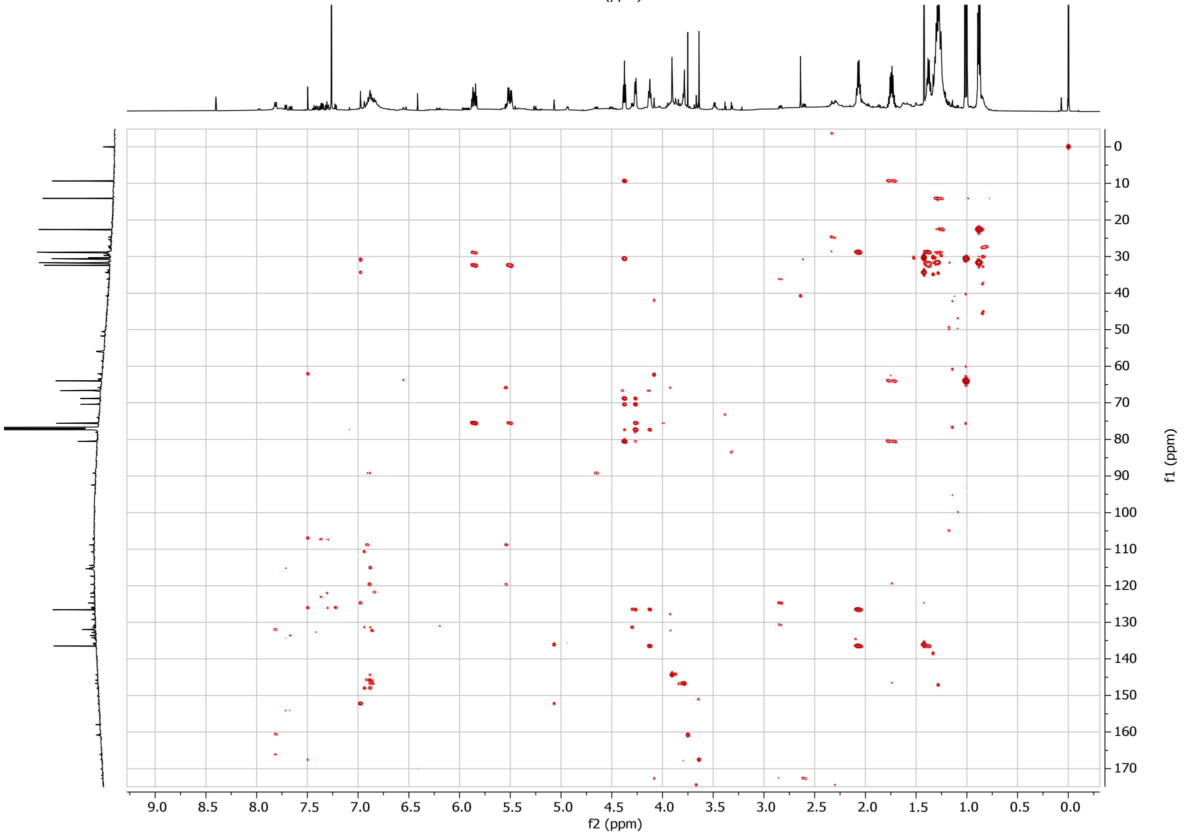

f2 (ppm)
f1 (ppm)

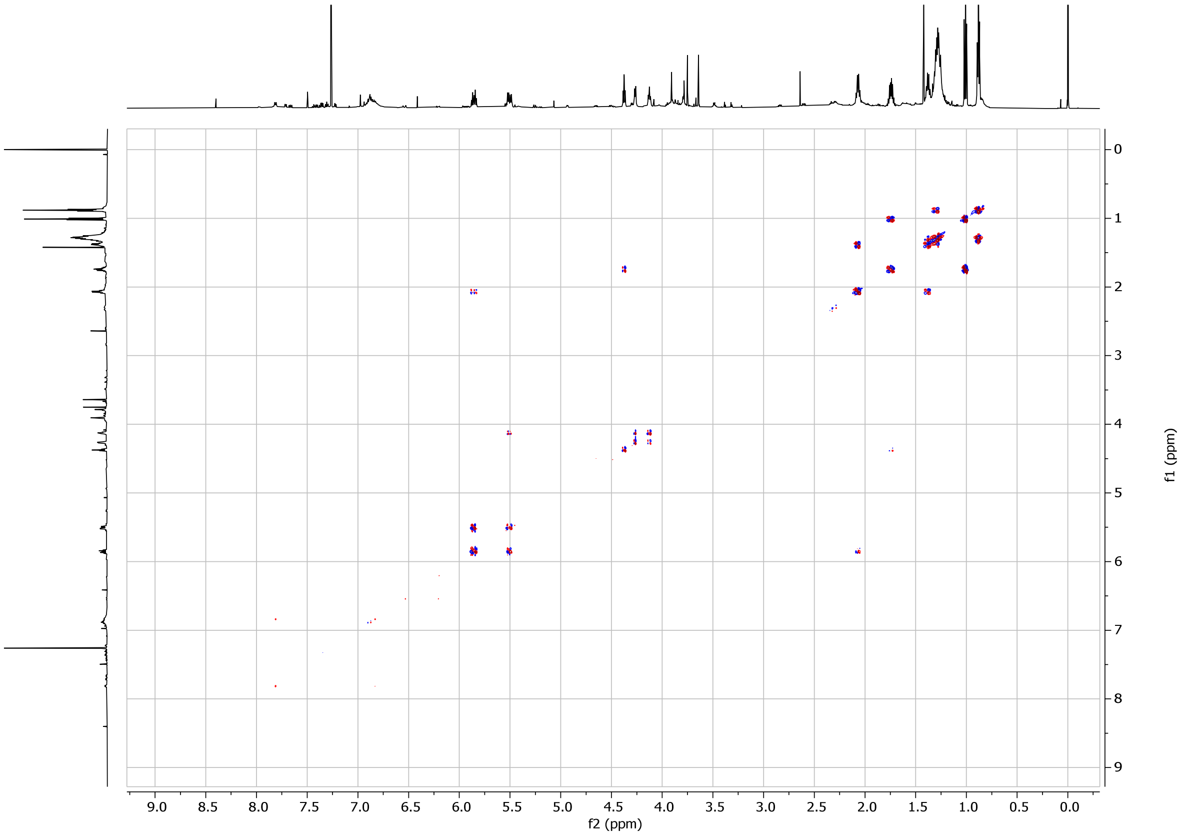
f2 (ppm)
f1 (ppm)

10 *Spectra to Natural Abundance of Isofalcarintriol*

10.1 Synthesis of Isofalcarintriol-*d*5

Synthesis of S9 (^{1}H, ^{2}H, ^{13}C, spectra)

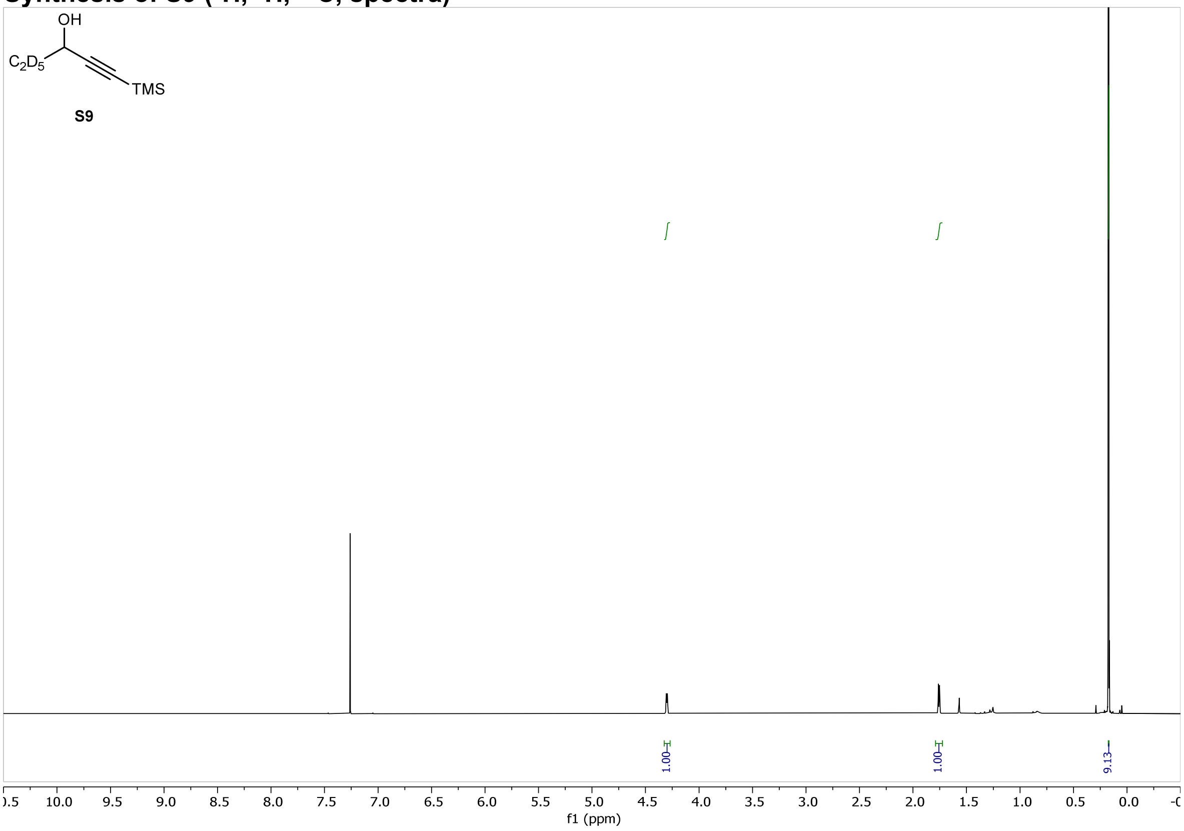

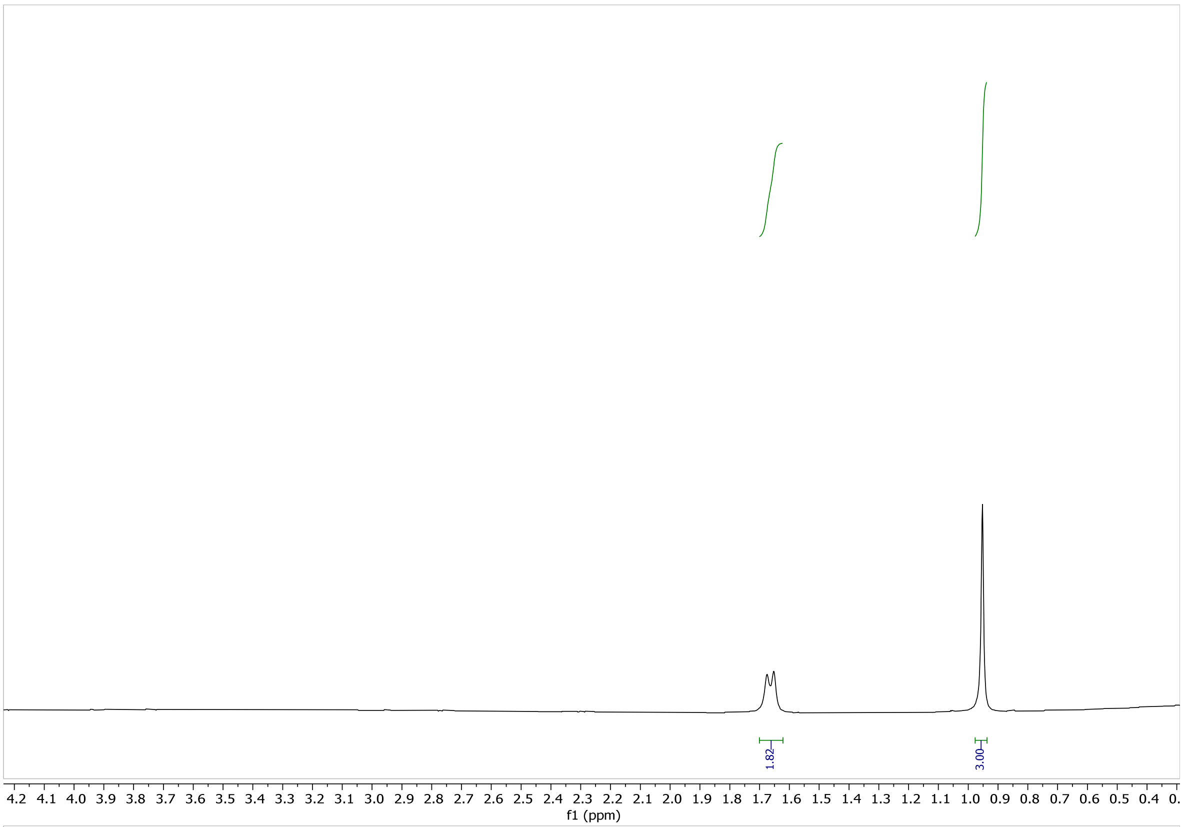
1.82
3.00
4.2 4.1 4.0 3.9 3.8 3.7 3.6 3.5 3.4 3.3 3.2 3.1 3.0 2.9 2.8 2.7 2.6 2.5 2.4 2.3 2.2 2.1 2.0 1.9 1.8 1.7 1.6 1.5 1.4 1.3 1.2 1.1 1.0 0.9 0.8 0.7 0.6 0.5 0.4
f1 (ppm)

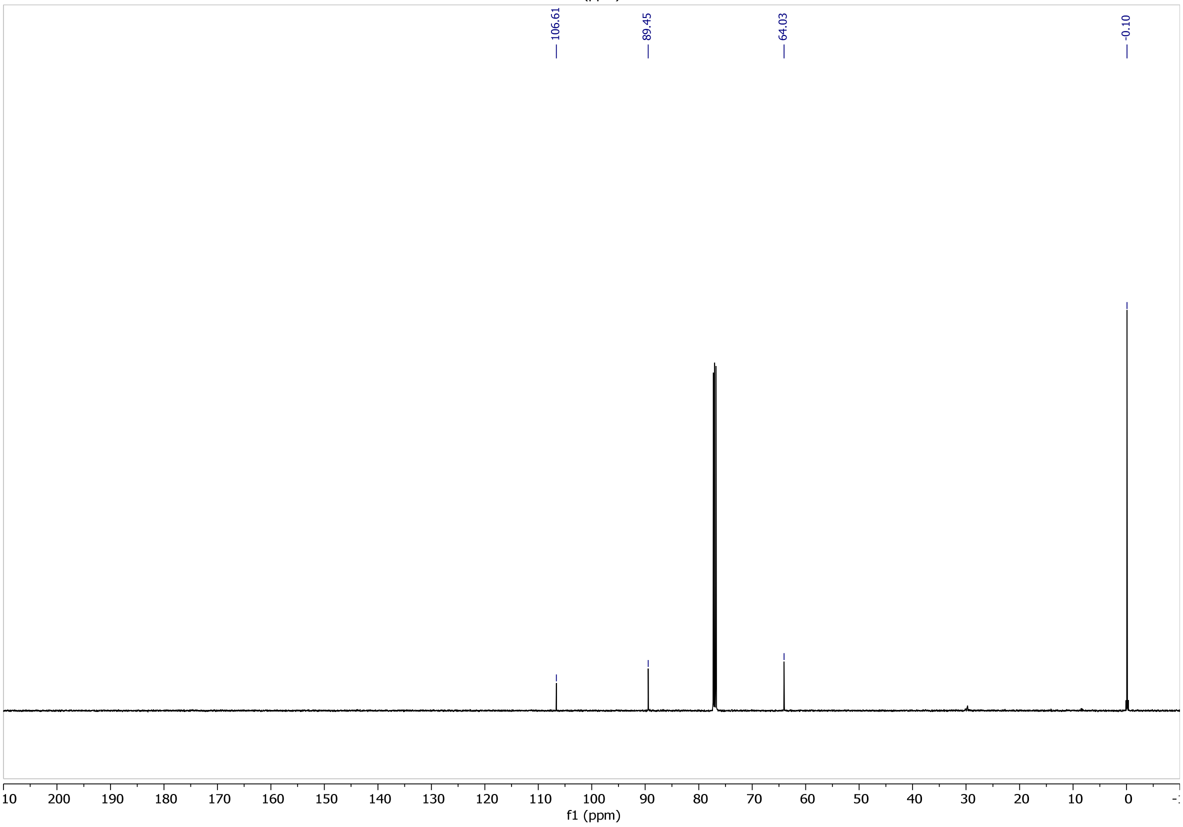
106.61
89.45
64.03
-0.10
200 190 180 170 160 150 140 130 120 110 100 90 80 70 60 50 40 30 20 10 0
f1 (ppm)

Synthesis of (*S*)-S9 and (*R*)-S10
(*S*)-S9 (^{1}H, ^{2}H, ^{13}C, HMBC spectra)

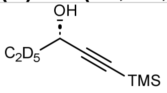

(*S*)-**S9**

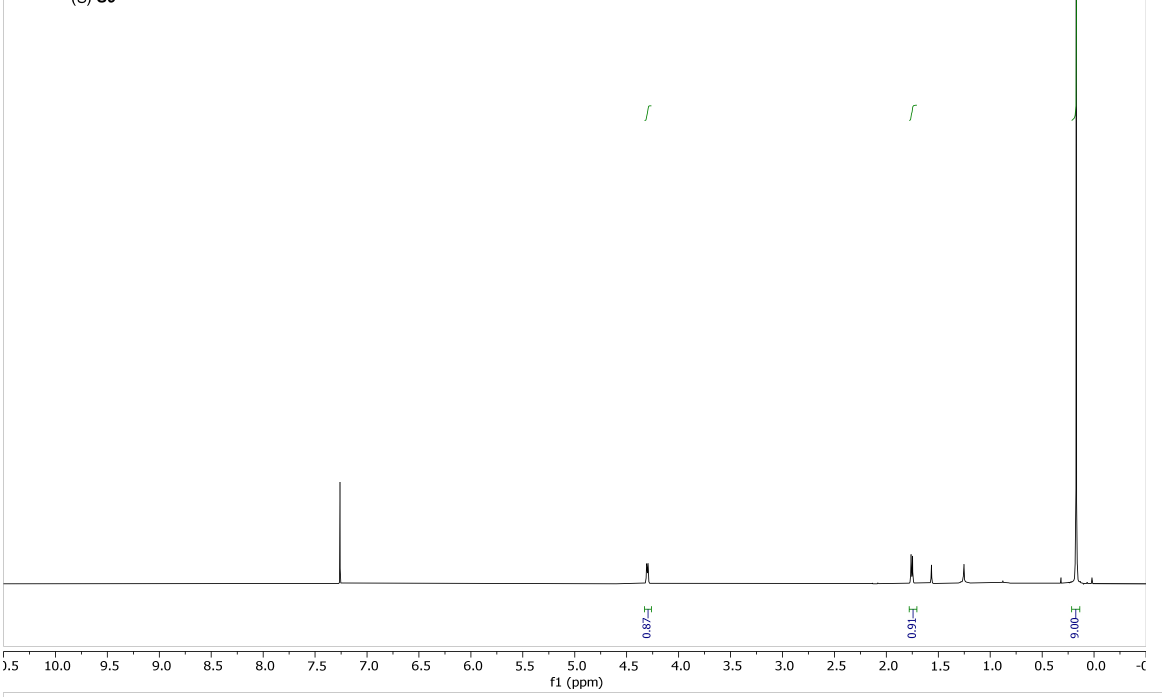

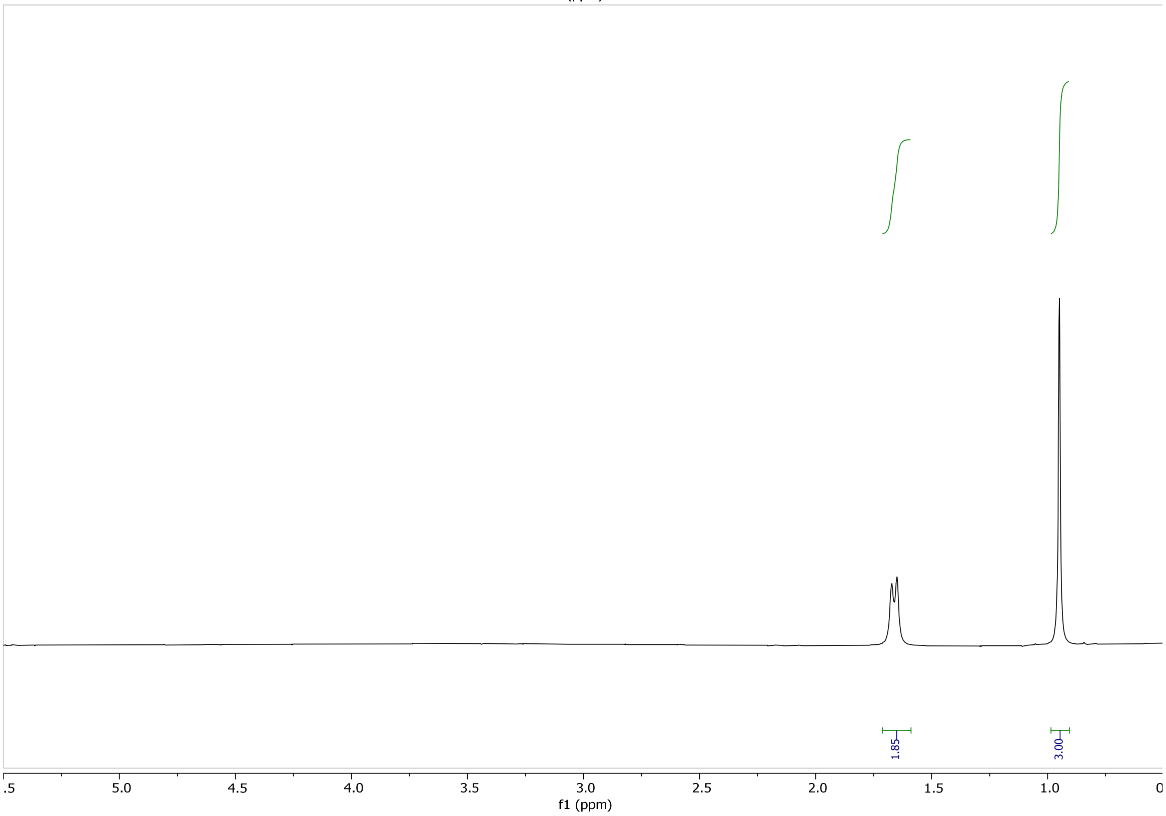

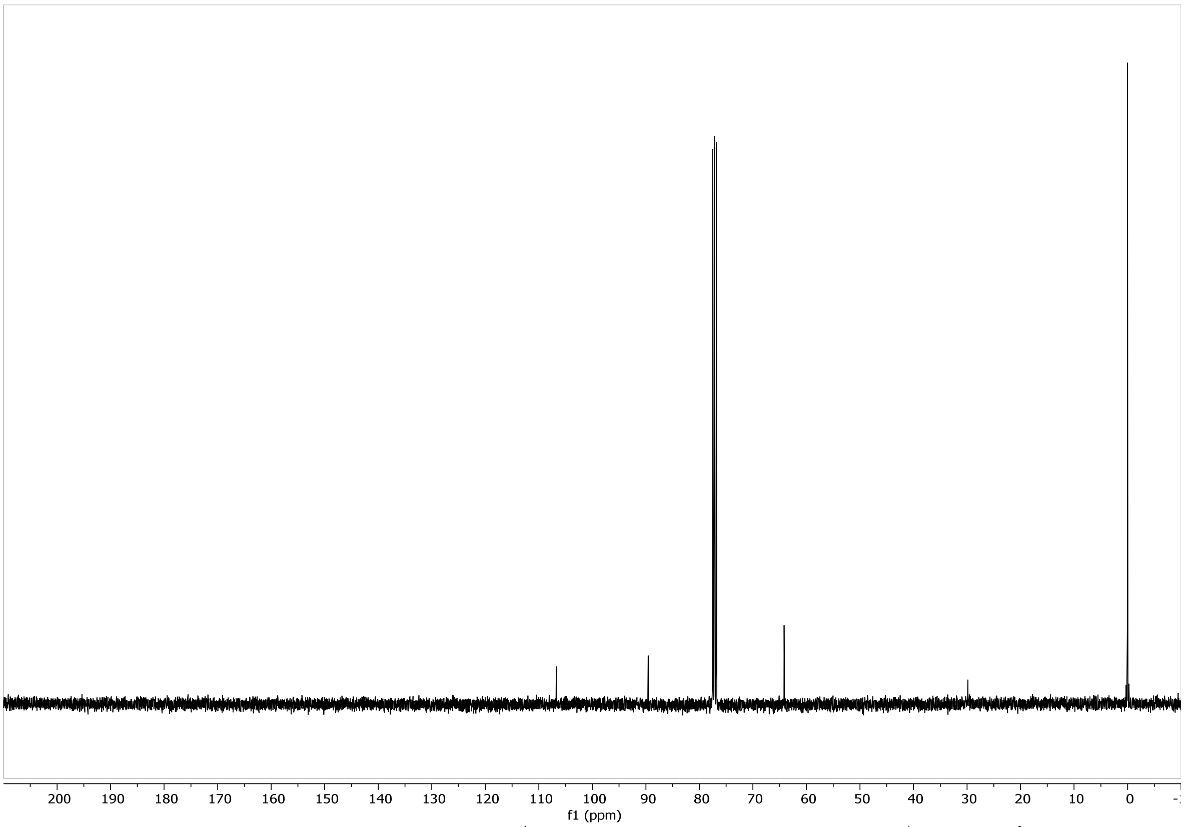
200
190
180
170
160
150
140
130
120
110
100
90
80
70
60
50
40
30
20
10
0
f1 (ppm)

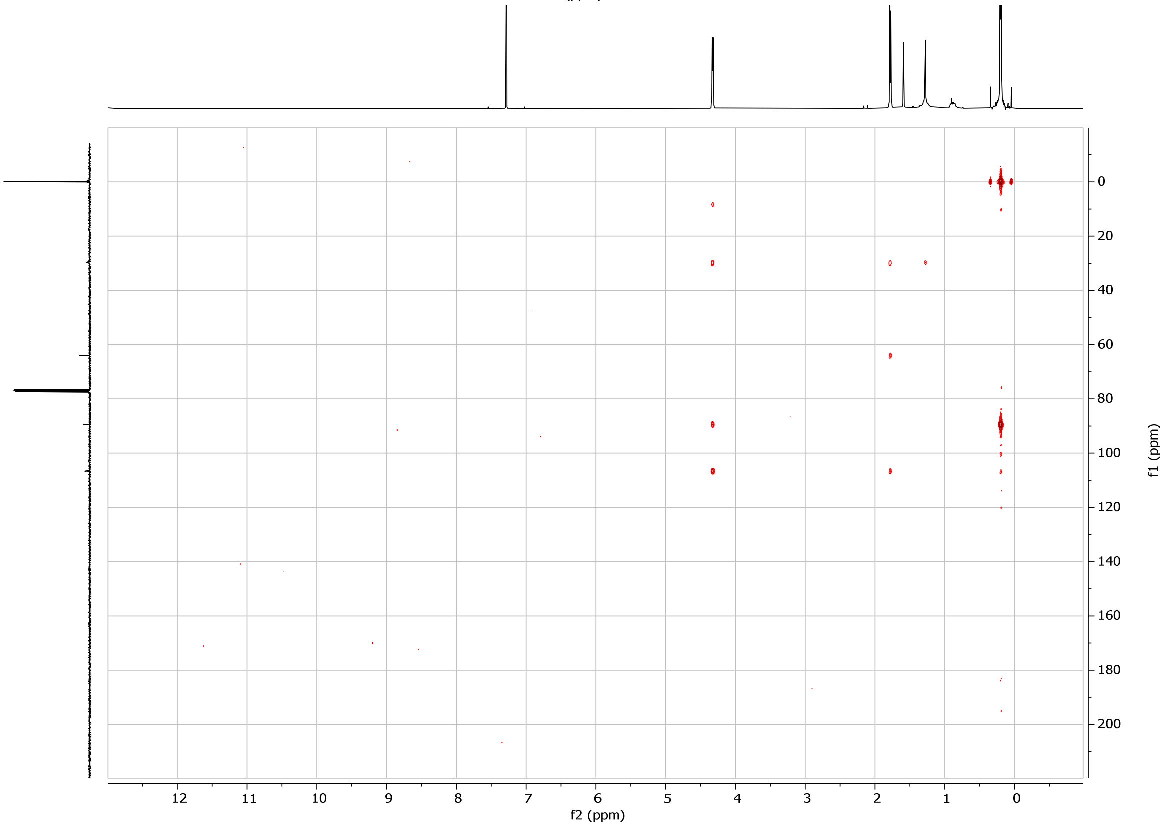
12
11
10
9
8
7
6
5
4
3
2
1
0
f2 (ppm)
0
20
40
60
80
100
120
140
160
180
200
f1 (ppm)

(*R*)-S10 (^{1}H, ^{2}H, ^{13}C, HMBC spectra)

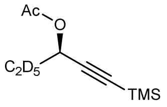

(*R*)-**S10**

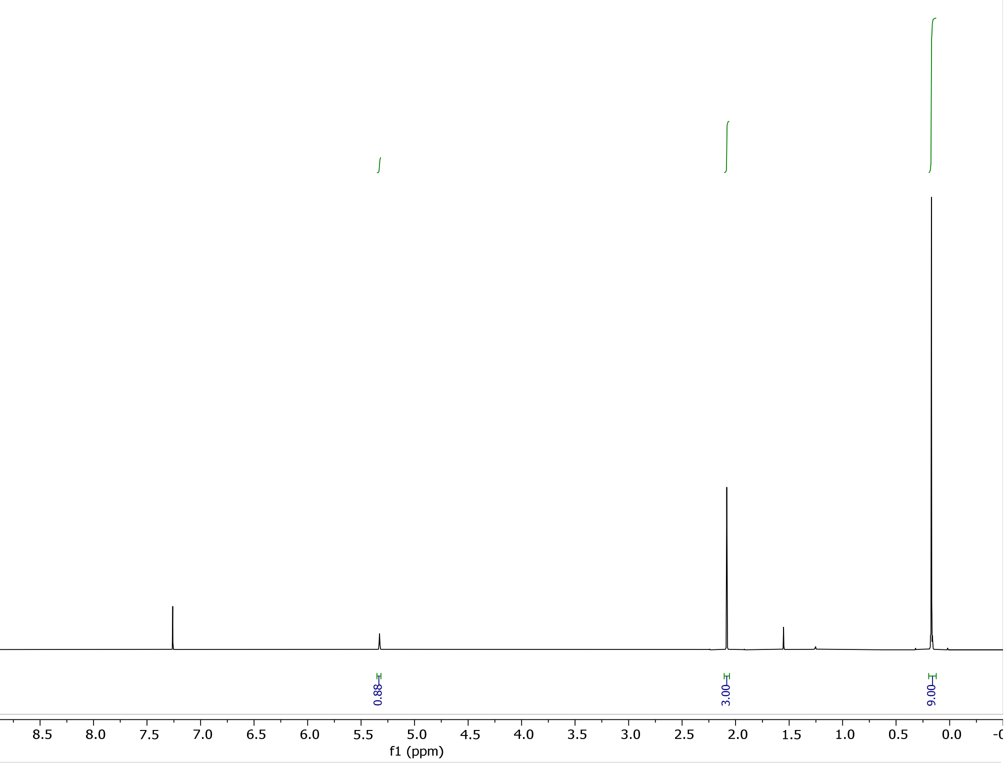

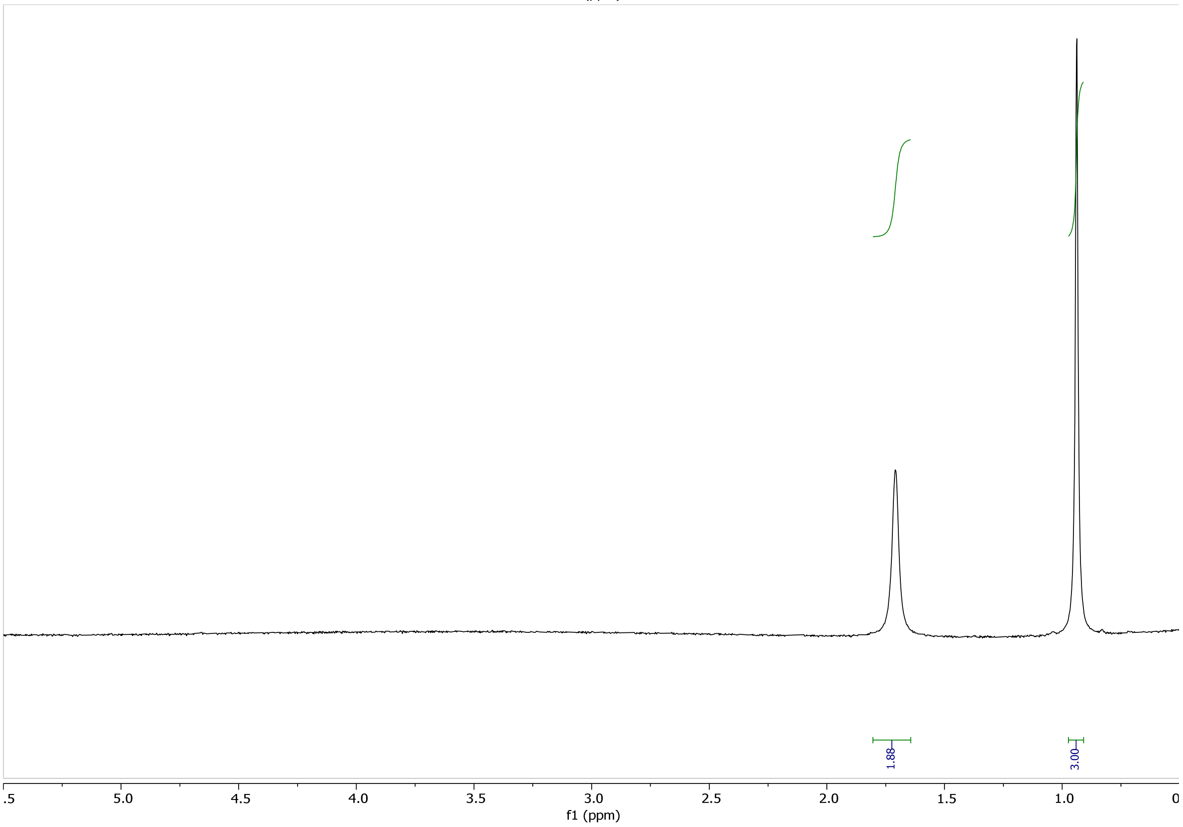

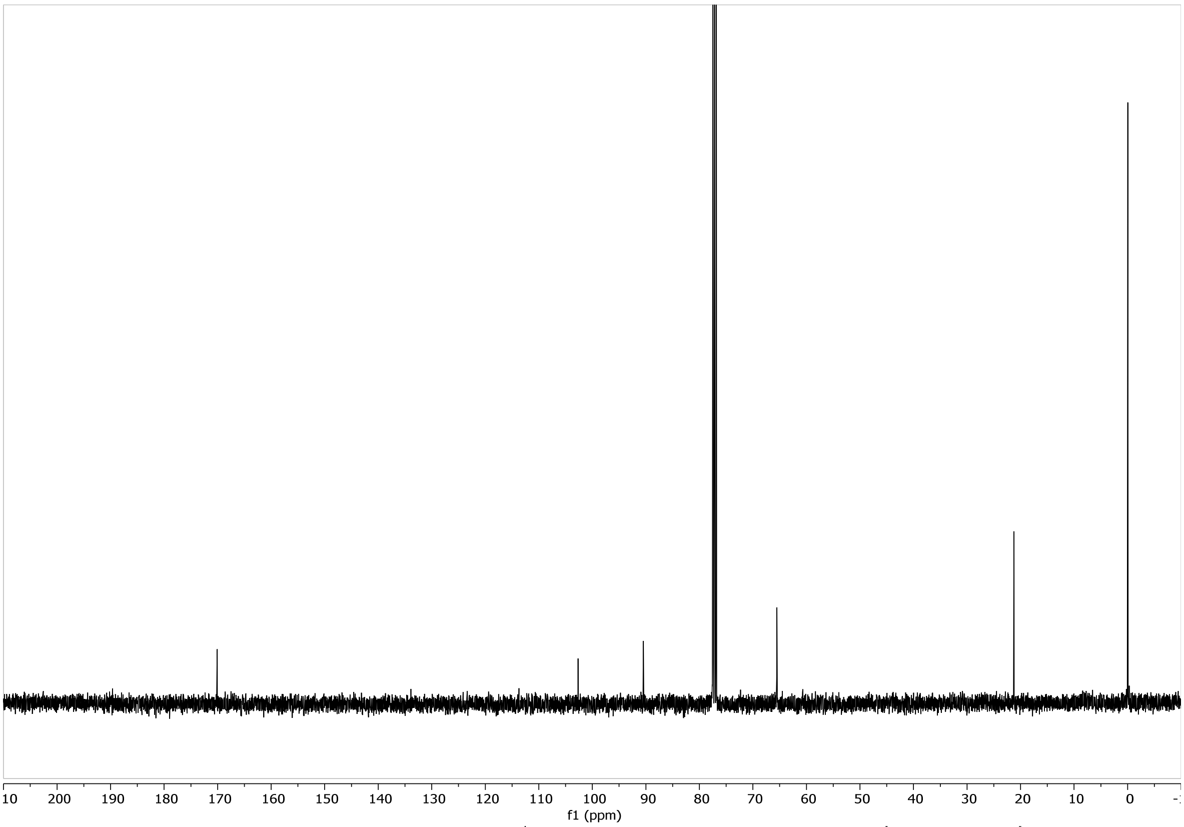

f1 (ppm)

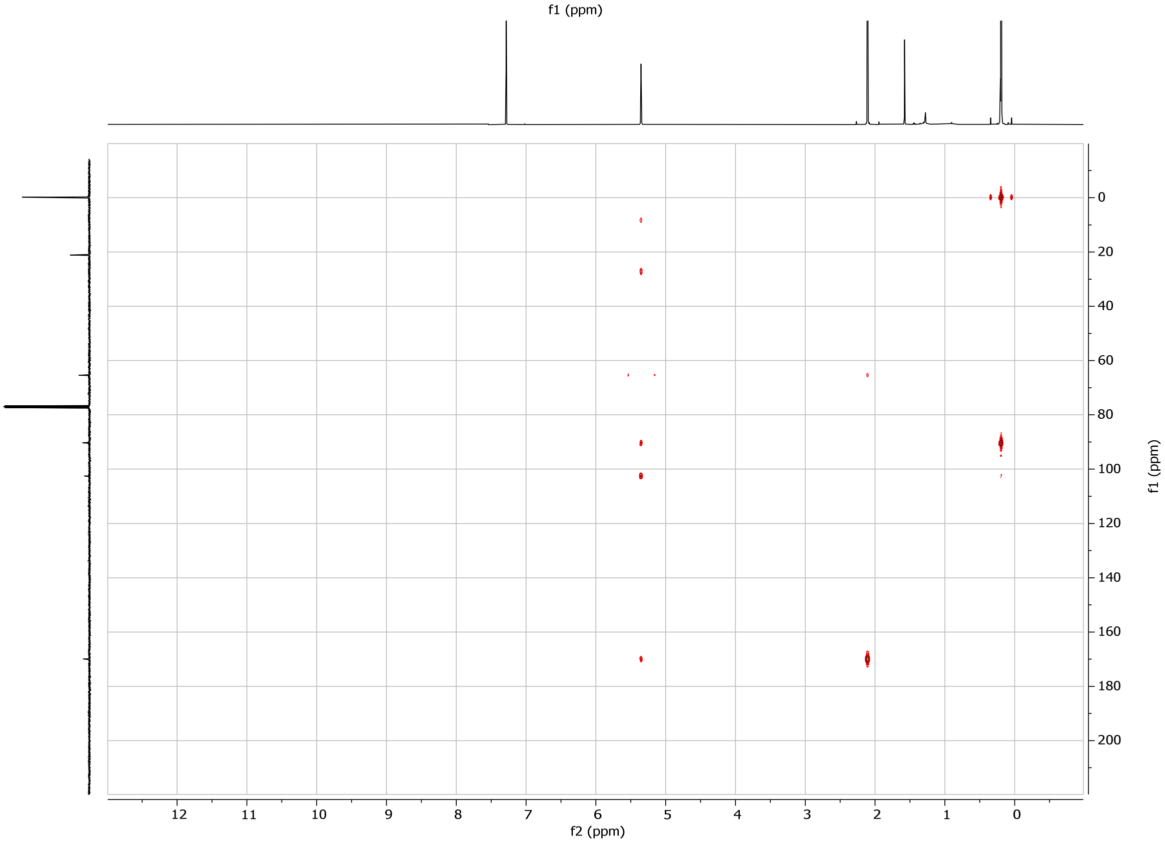

f2 (ppm)
f1 (ppm)

Synthesis of 13 (^{1}H, ^{13}C, HMBC spectra)

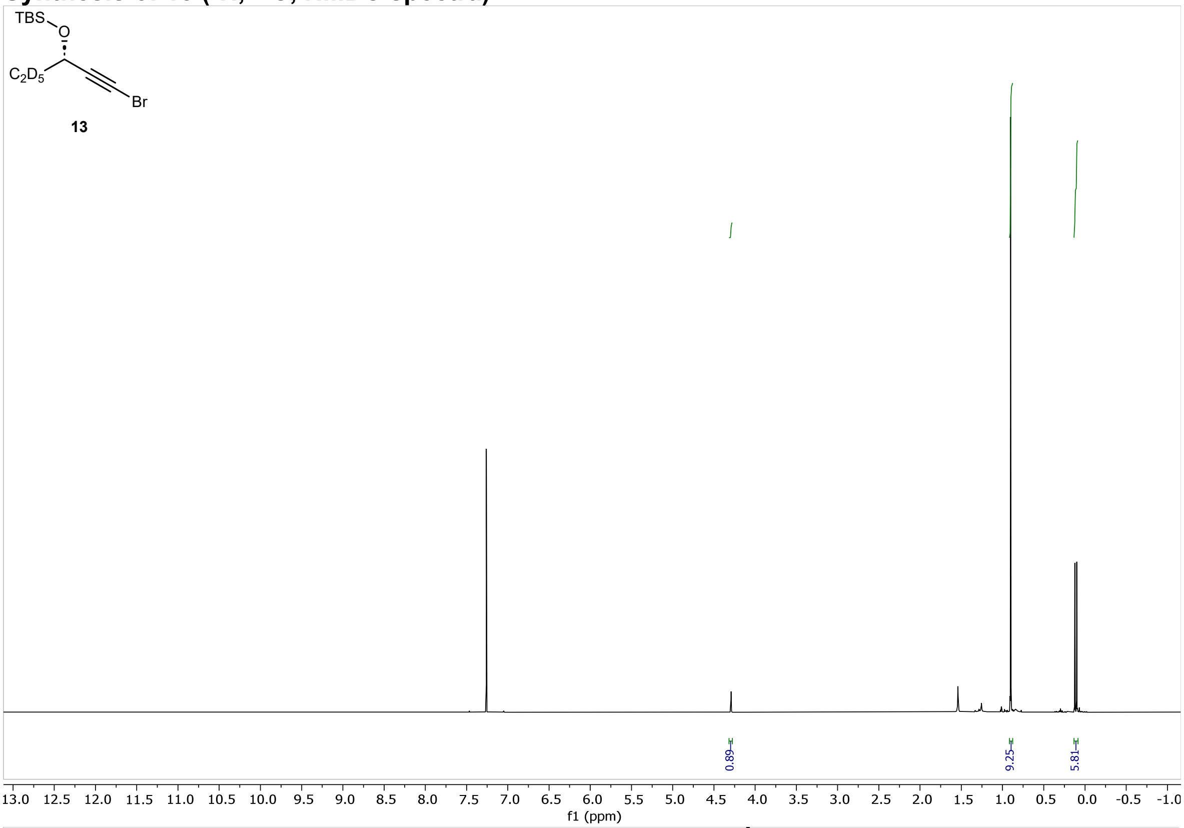

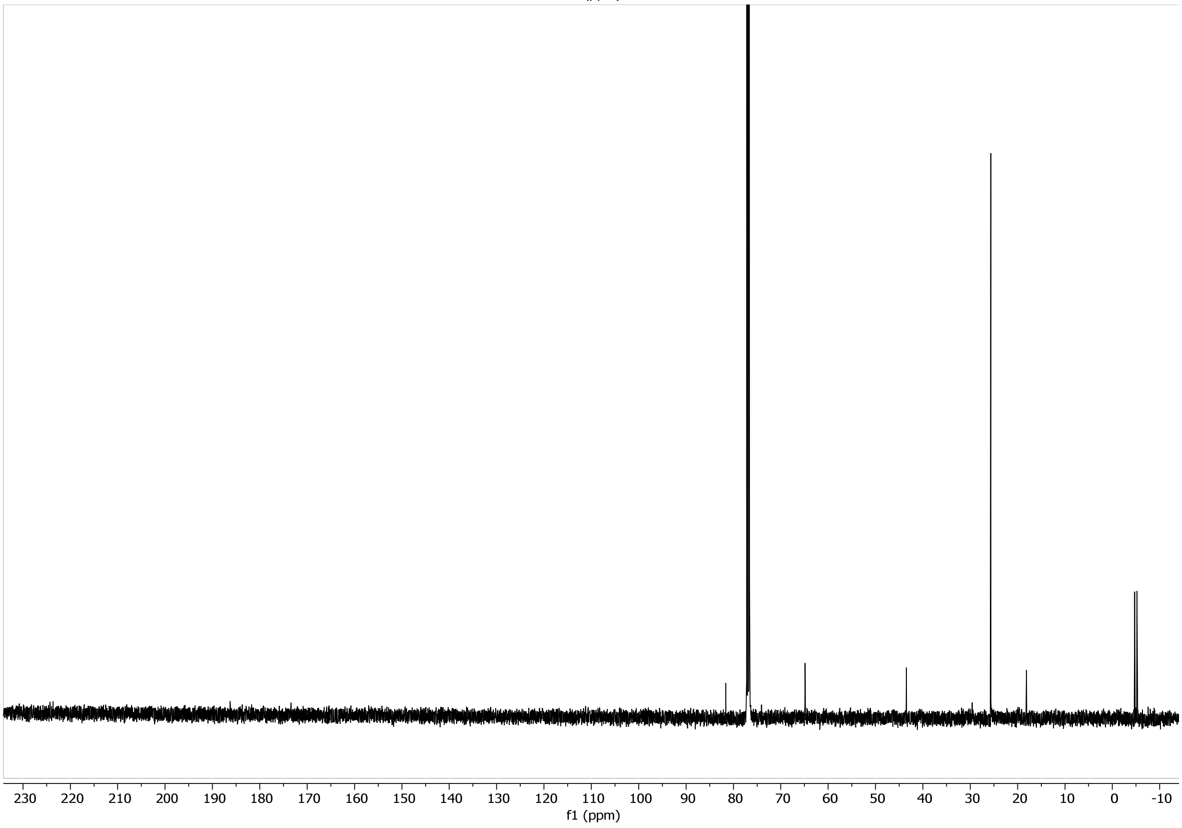

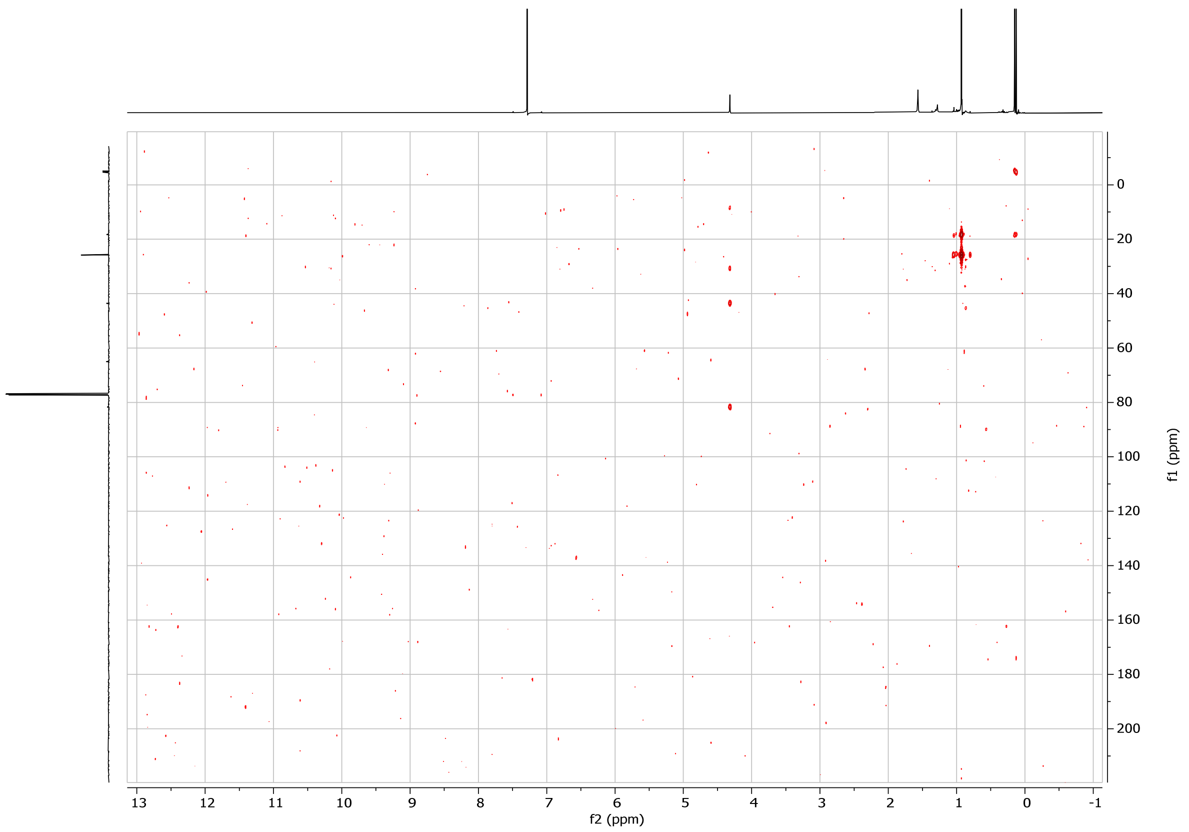

f2 (ppm)
f1 (ppm)

Synthesis of S11 (^{1}H, ^{13}C, HSQC, HMBC, COSY spectra)

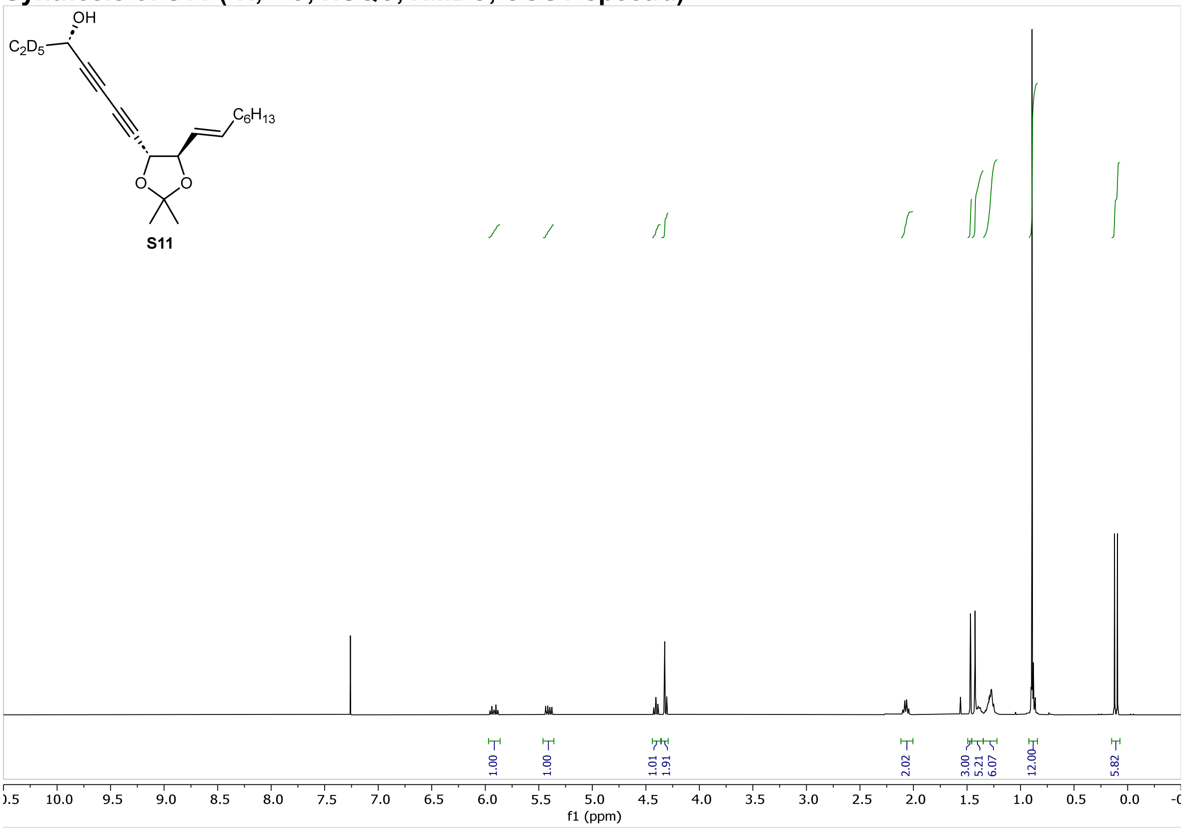

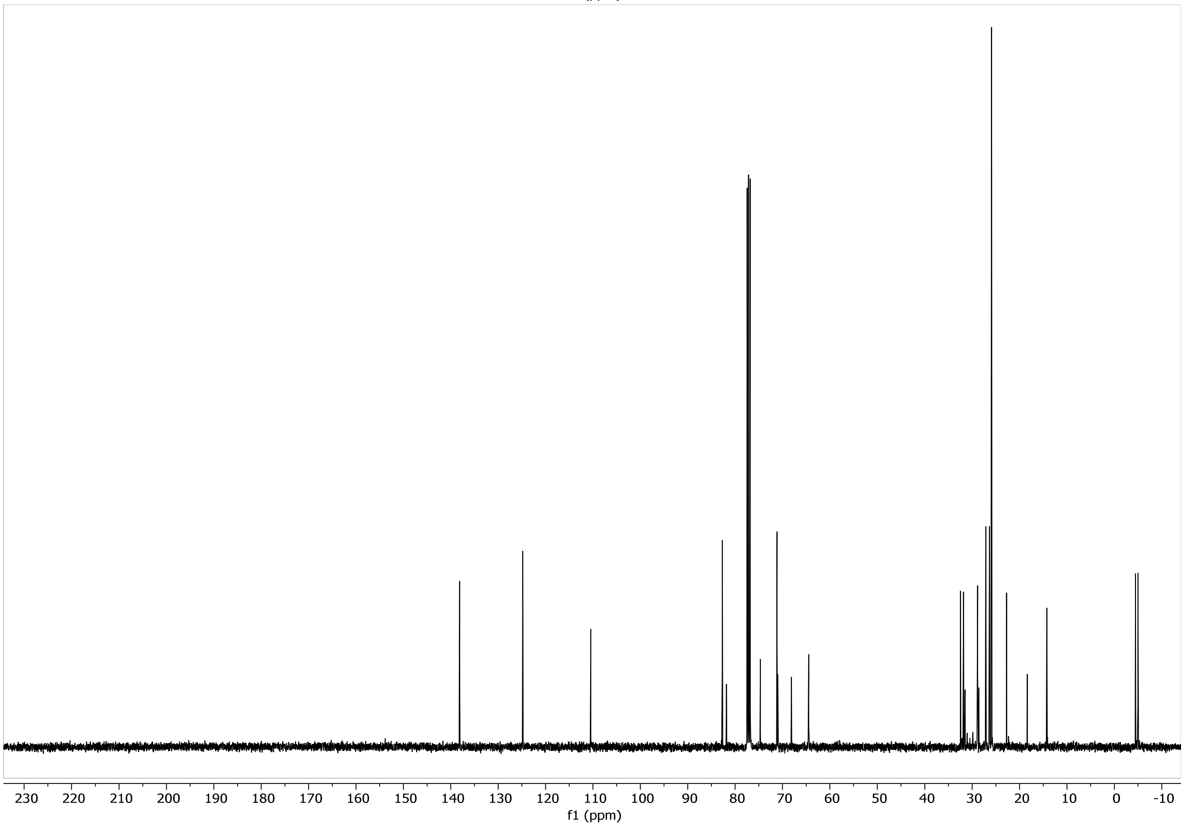

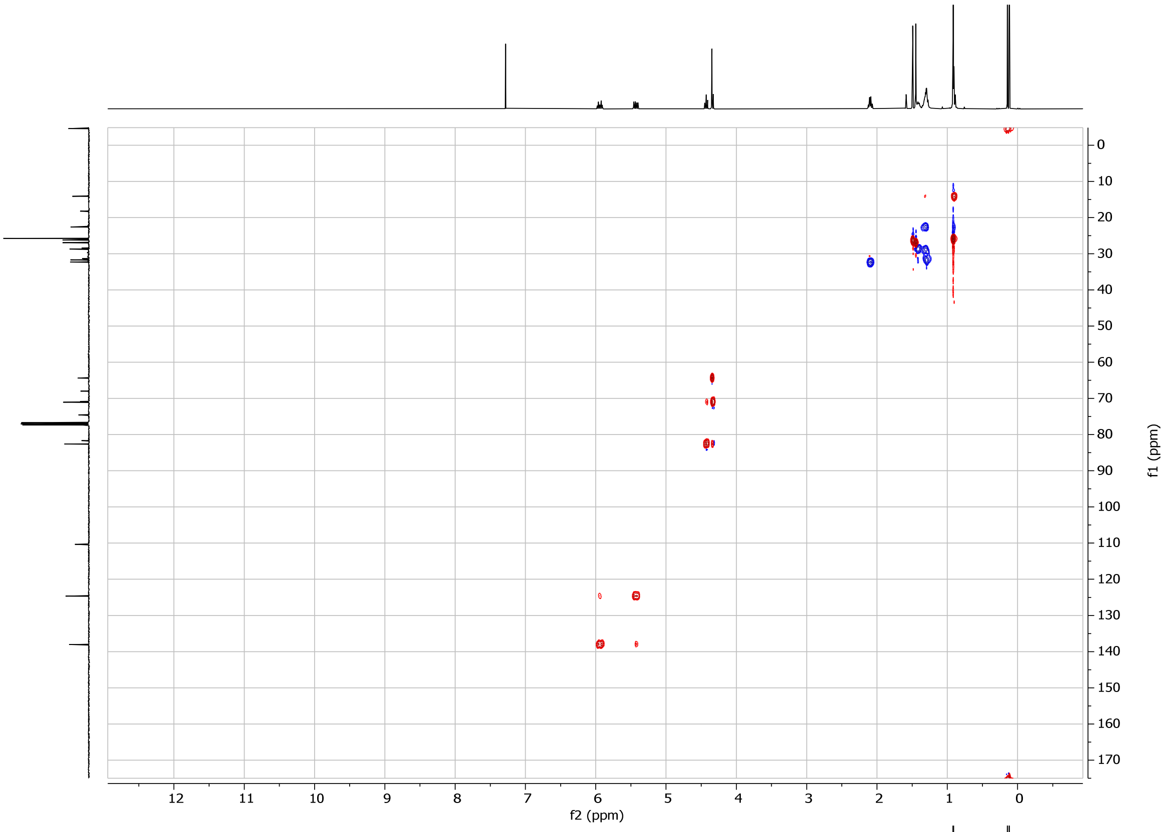
12 11 10 9 8 7 6 5 4 3 2 1 0
f2 (ppm)
0 10 20 30 40 50 60 70 80 90 100 110 120 130 140 150 160 170
f1 (ppm)

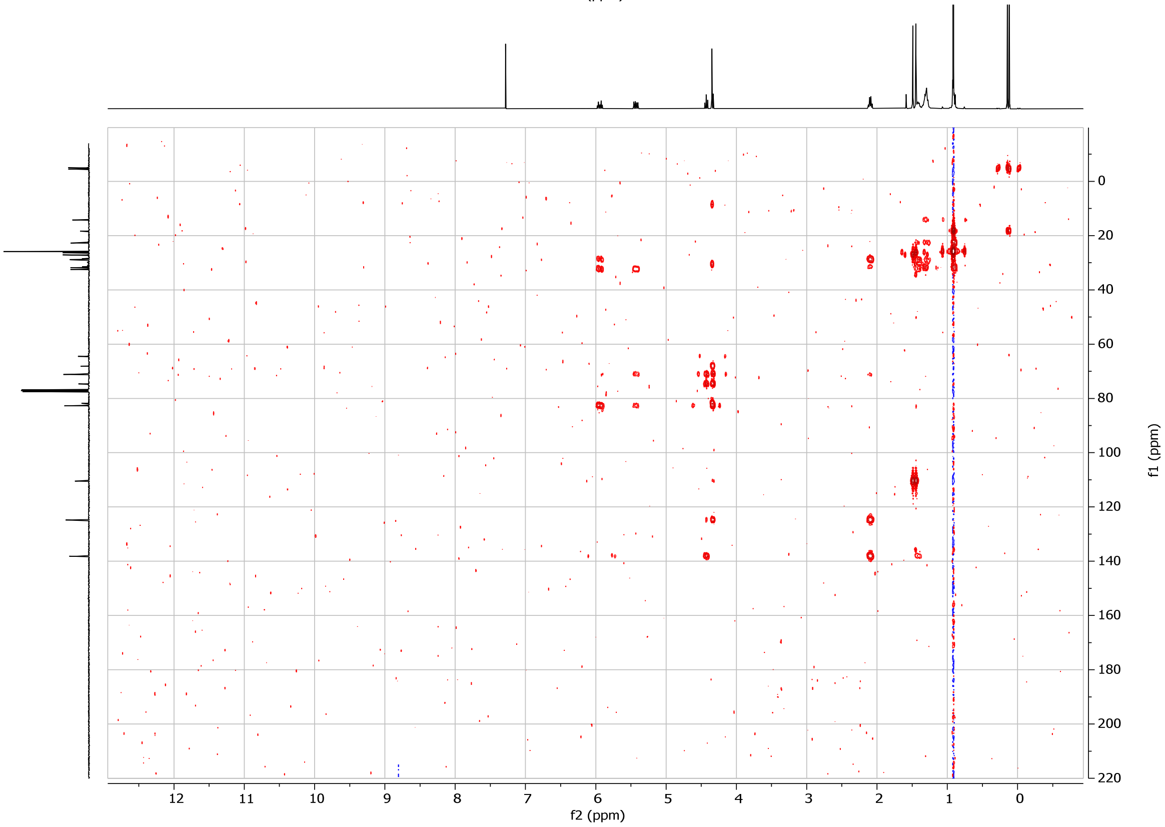
12 11 10 9 8 7 6 5 4 3 2 1 0
f2 (ppm)
0 20 40 60 80 100 120 140 160 180 200 220
f1 (ppm)

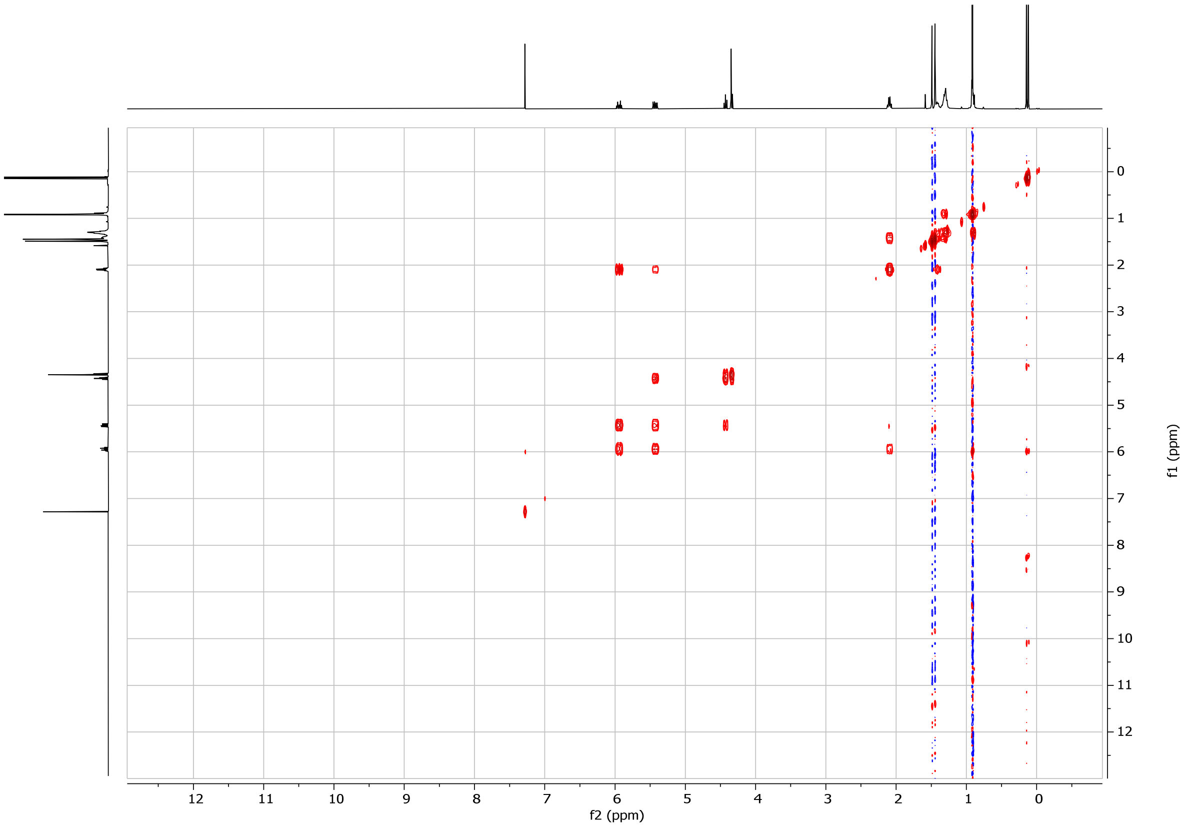
12
11
10
9
8
7
6
5
4
3
2
1
0
f2 (ppm)
f1 (ppm)

Synthesis of 12 (^{1}H, ^{2}H, ^{13}C, HSQC, HMBC, COSY spectra)

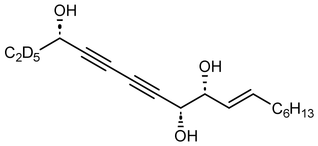

12

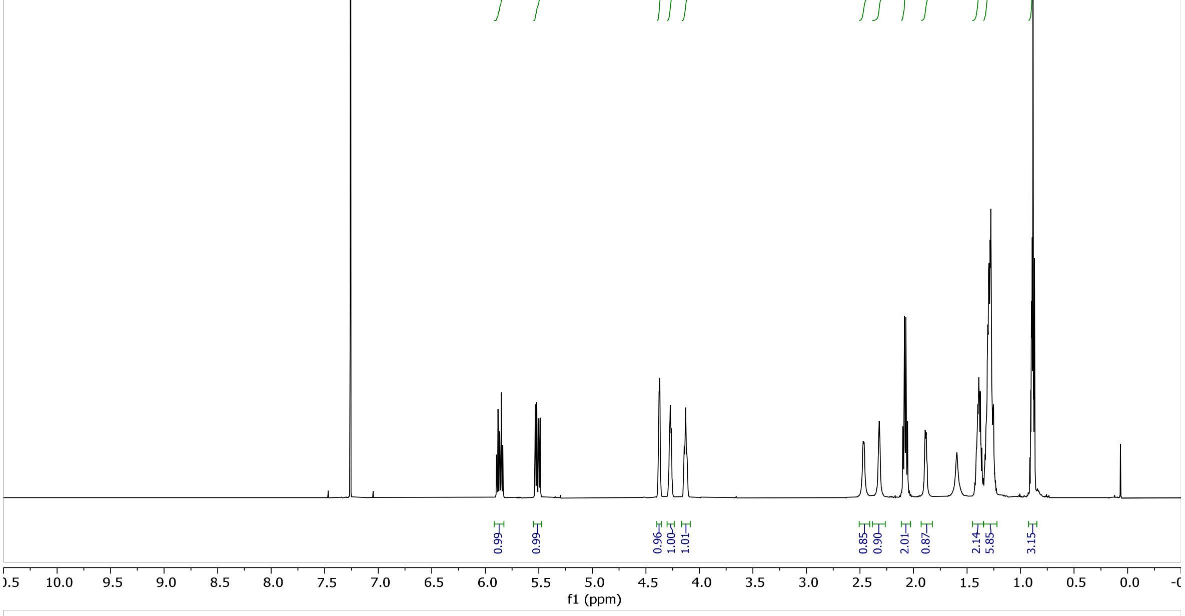

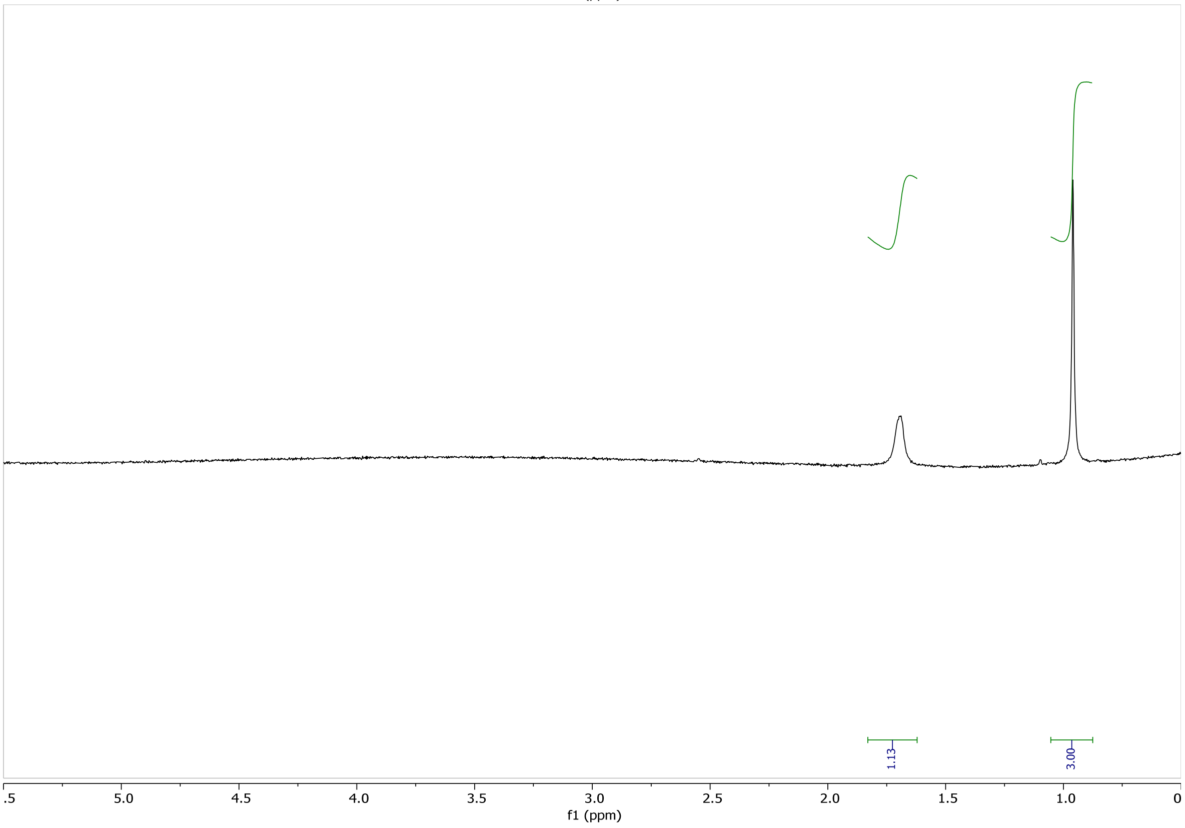

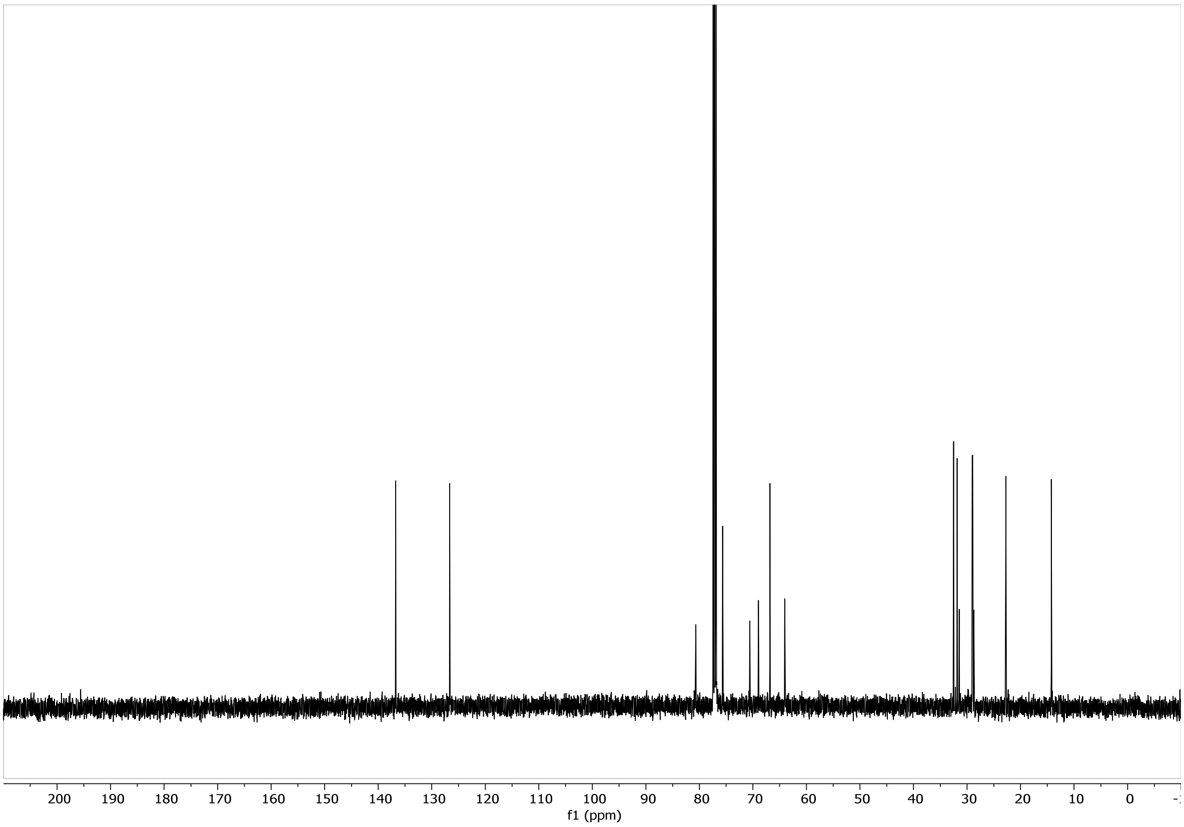
200 190 180 170 160 150 140 130 120 110 100 90 80 70 60 50 40 30 20 10 0
f1 (ppm)

400 MHz

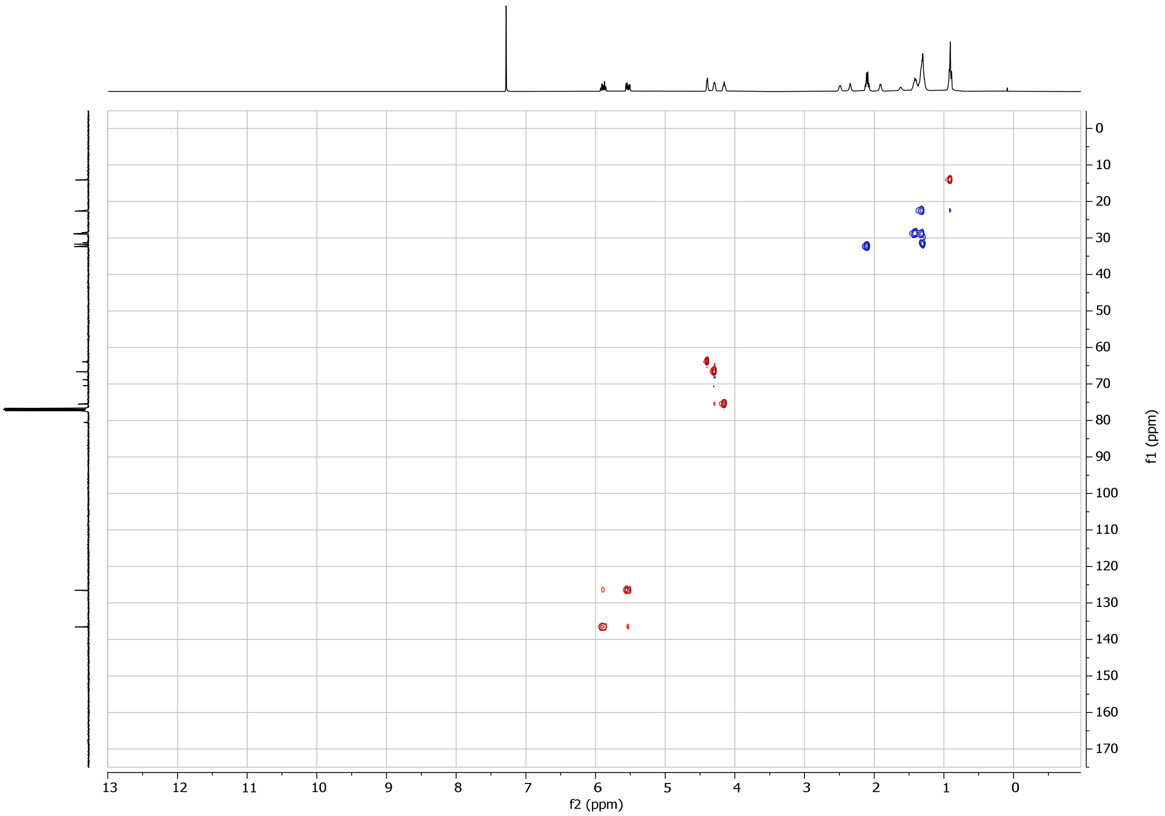
f2 (ppm)
f1 (ppm)

500MHz

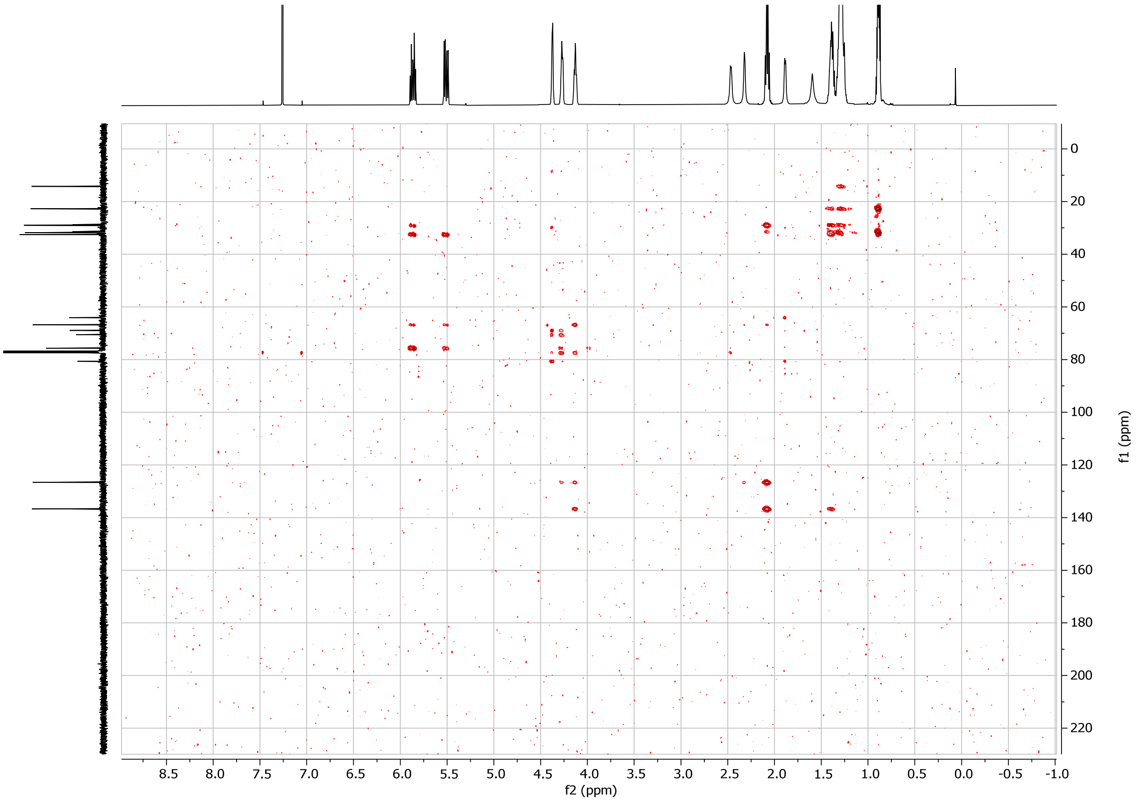

400MHz

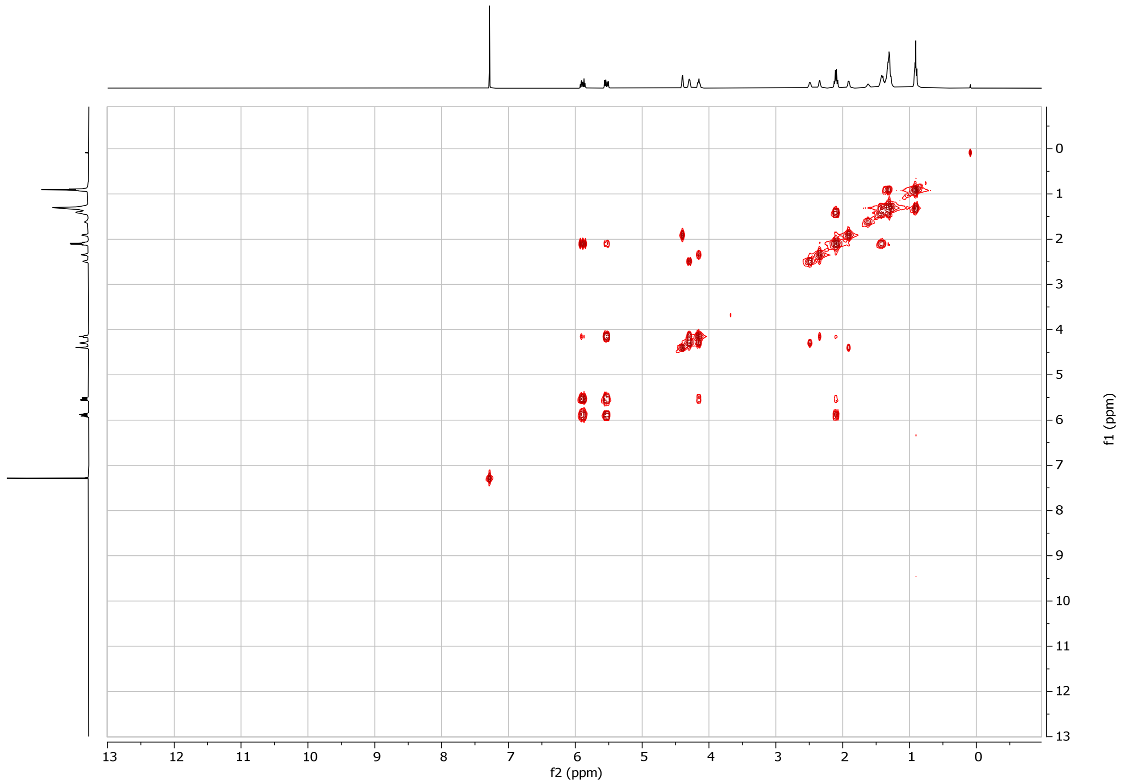

11 Spectra to Structure-Activity Relationship of Isofalcarintriol

Synthesis of S20 (^{1}H, ^{13}C, HMBC spectra)

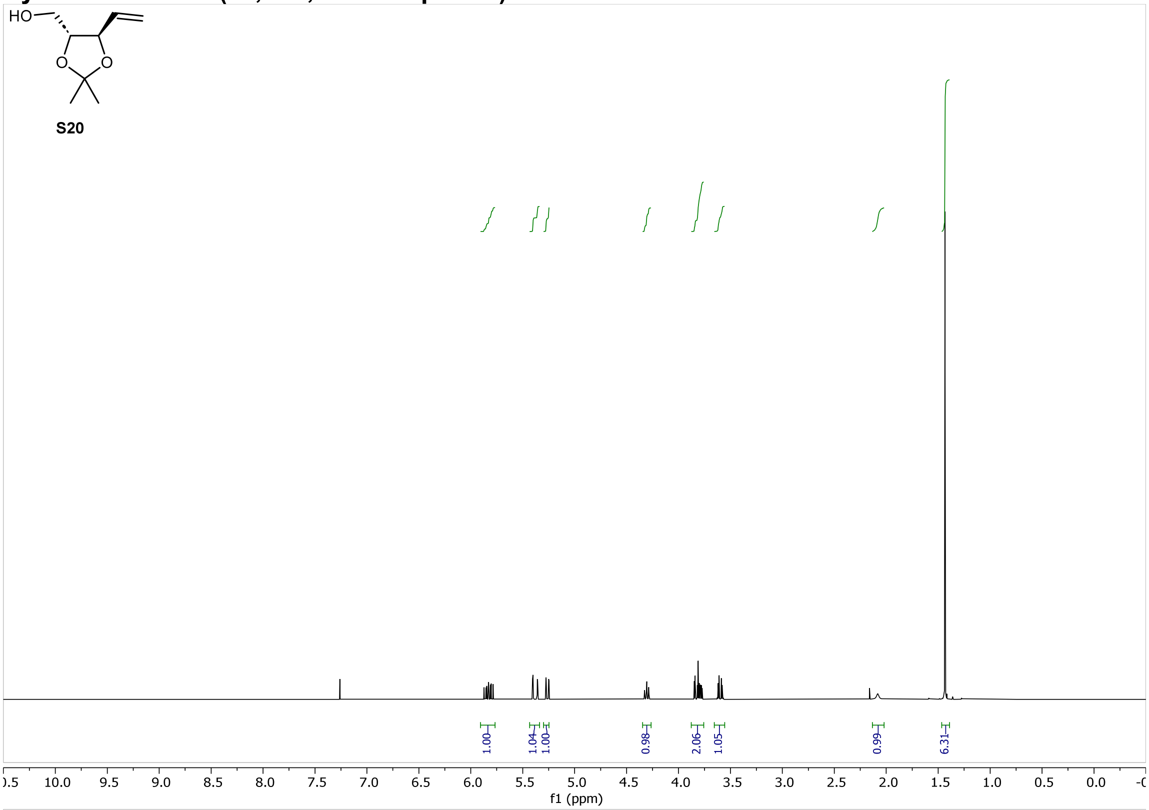

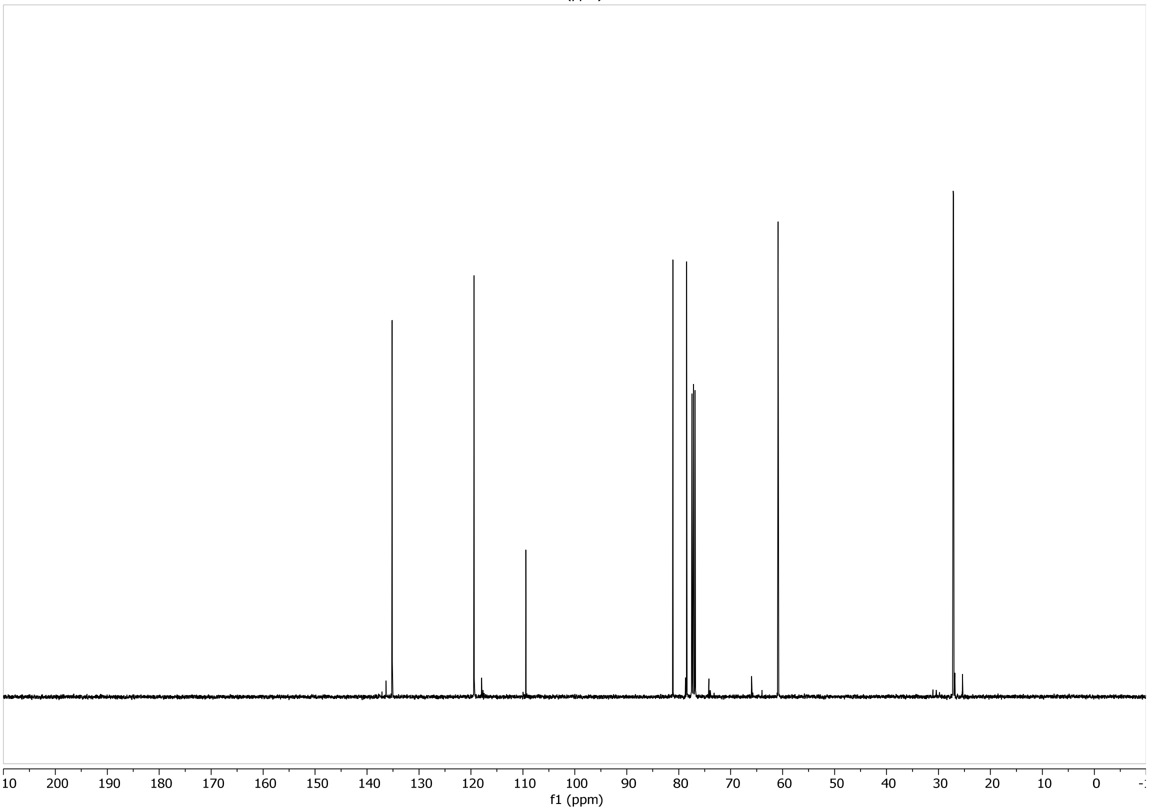

Synthesis of S13 (^{1}H, ^{13}C spectra)

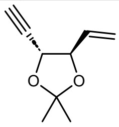

S13

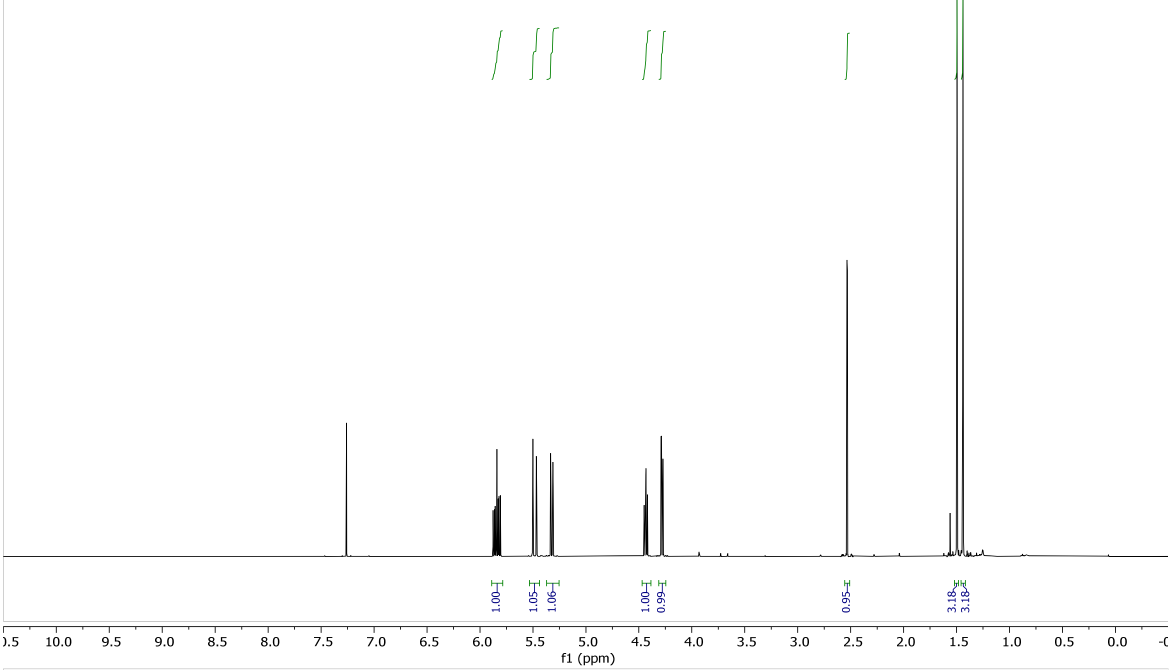

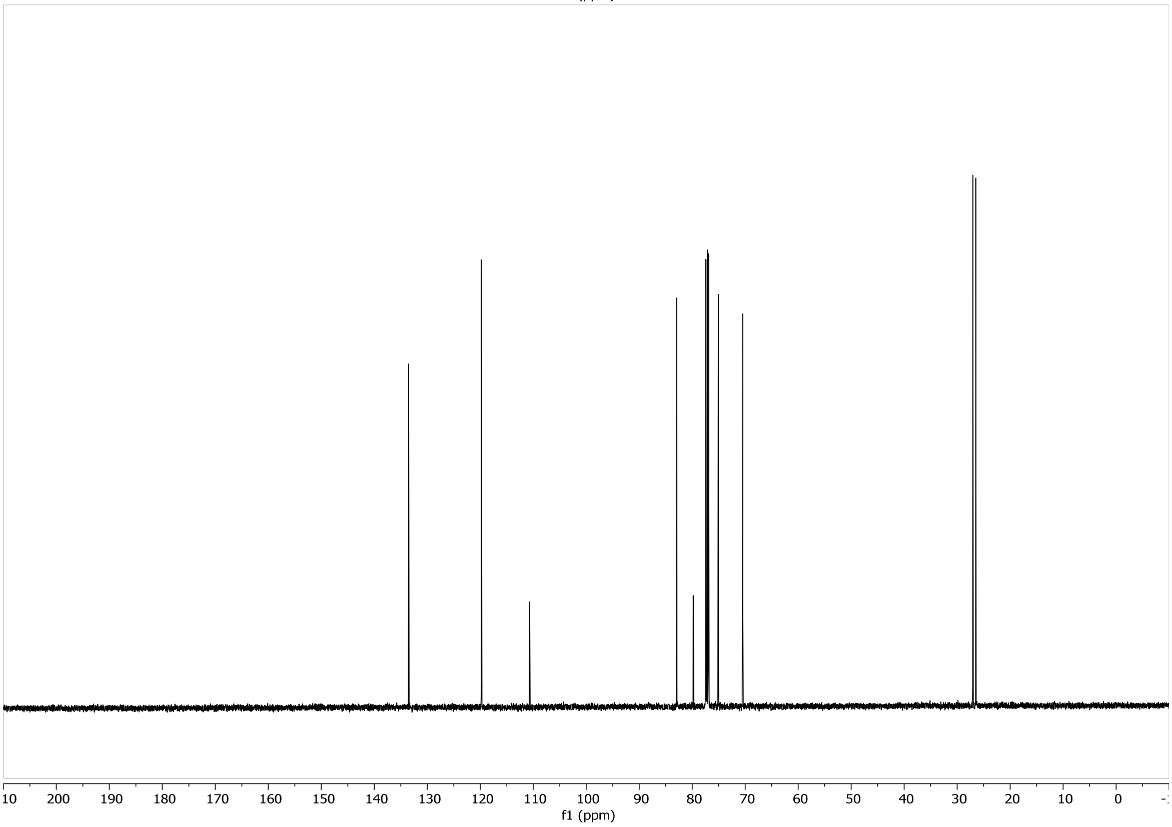

Synthesis of S14 (^{1}H, ^{13}C, HSQC, HMBC, COSY spectra)

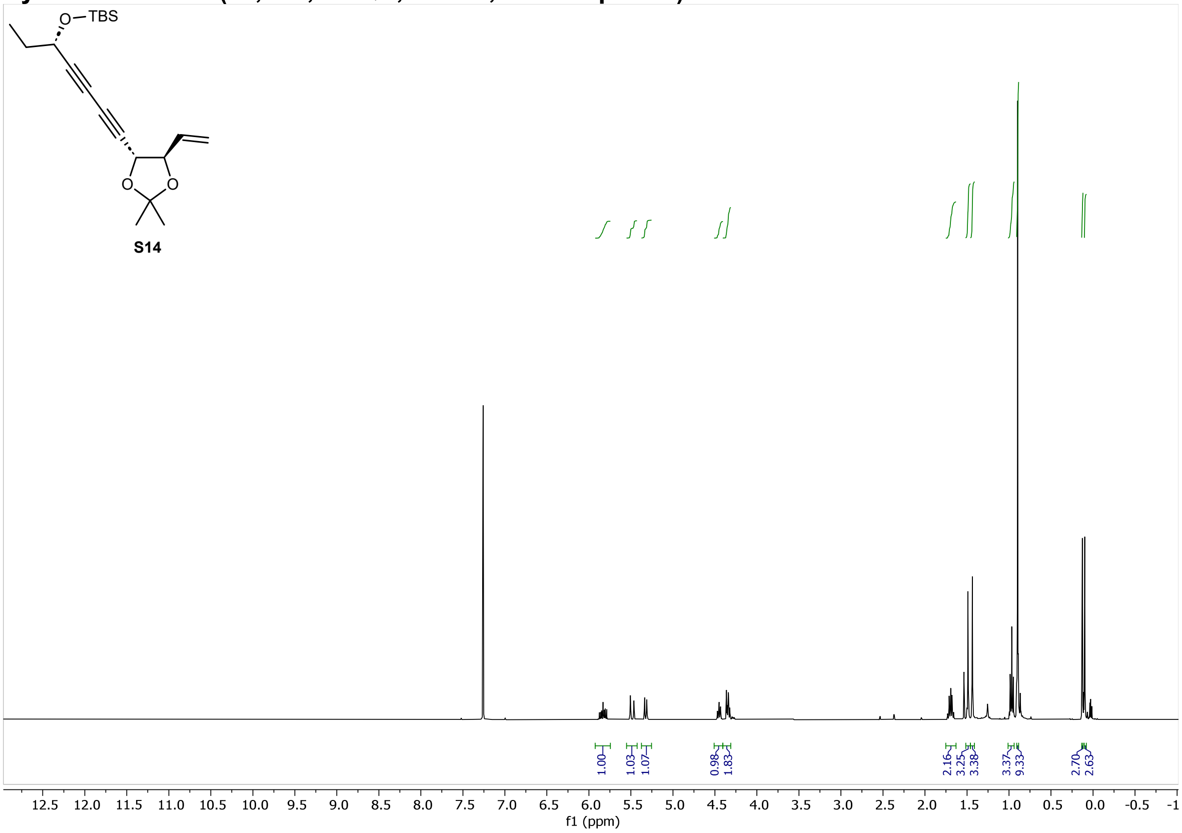

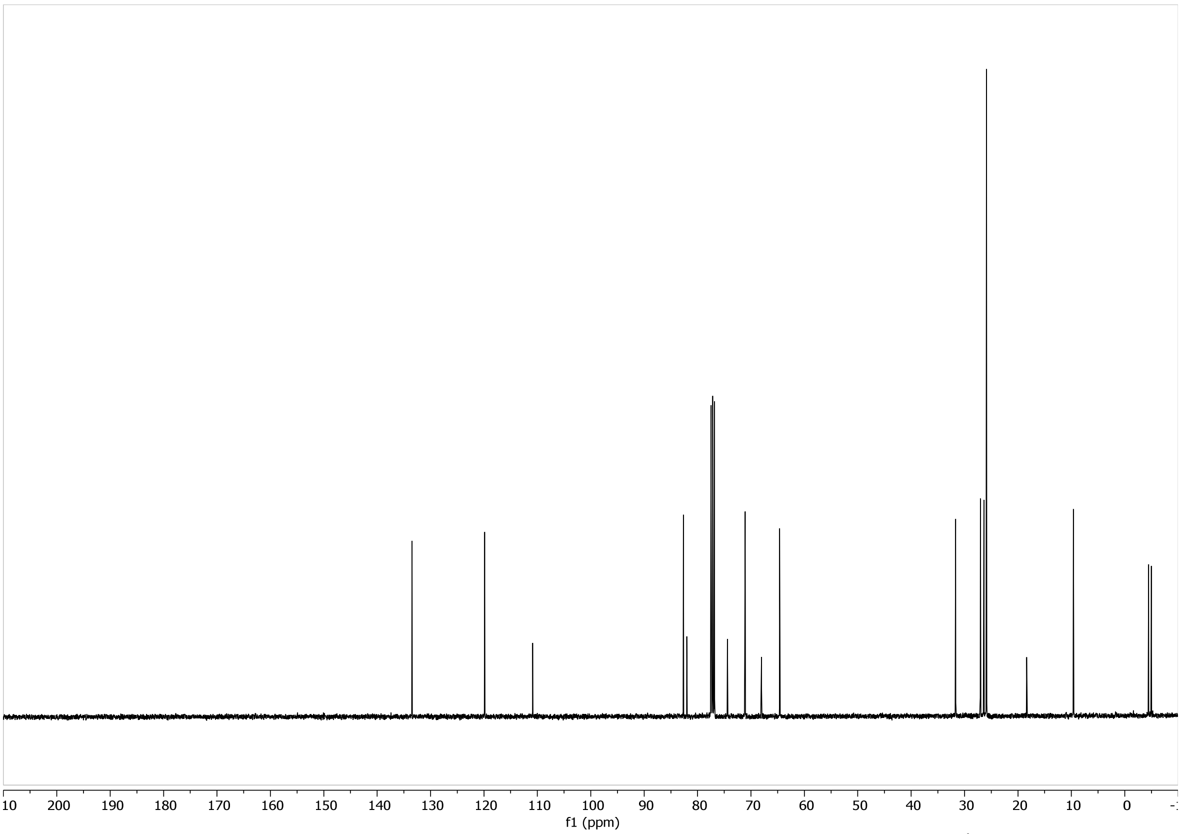
f1 (ppm)

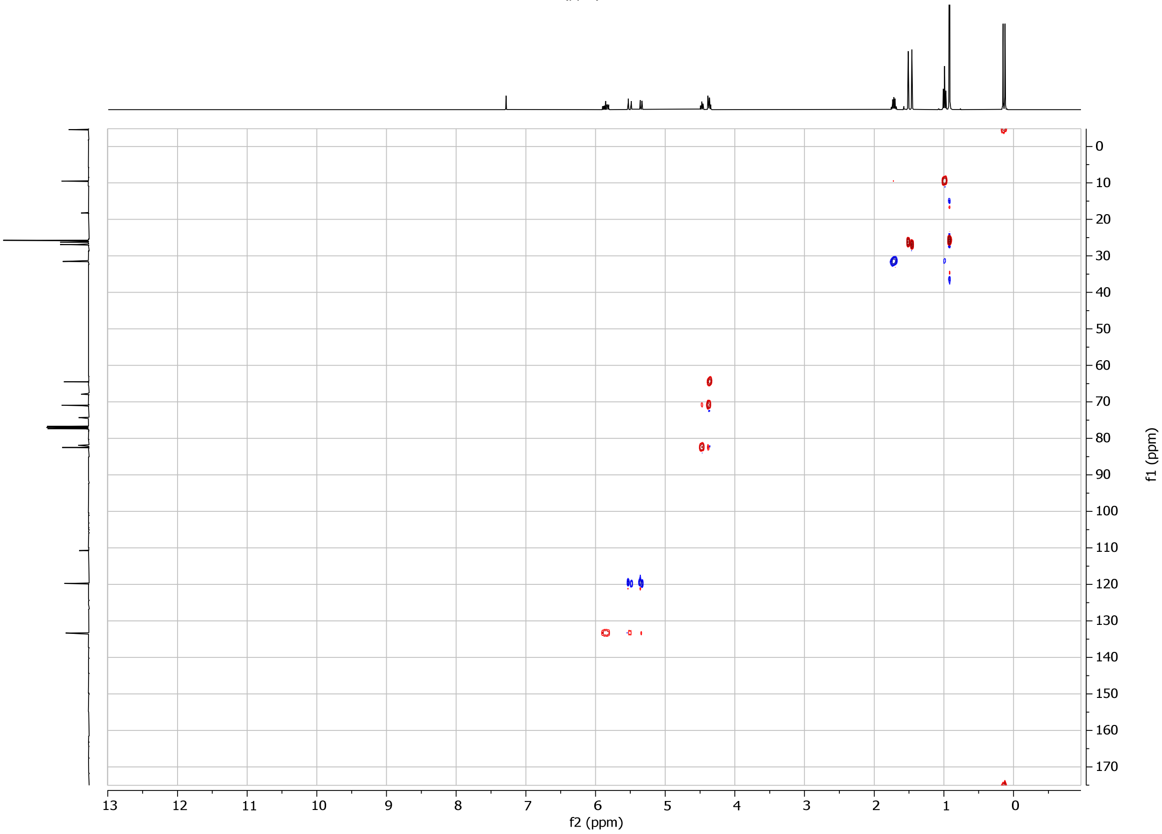
f2 (ppm)
f1 (ppm)

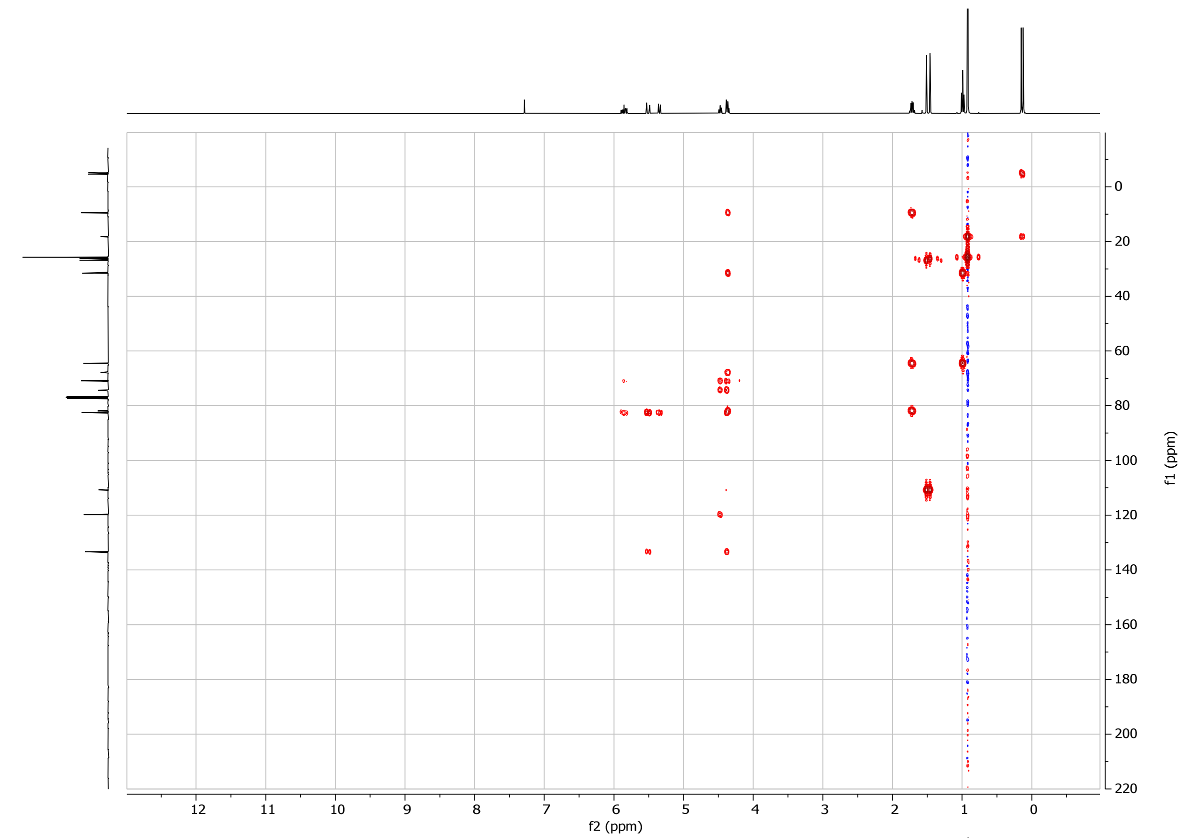
f2 (ppm)
f1 (ppm)

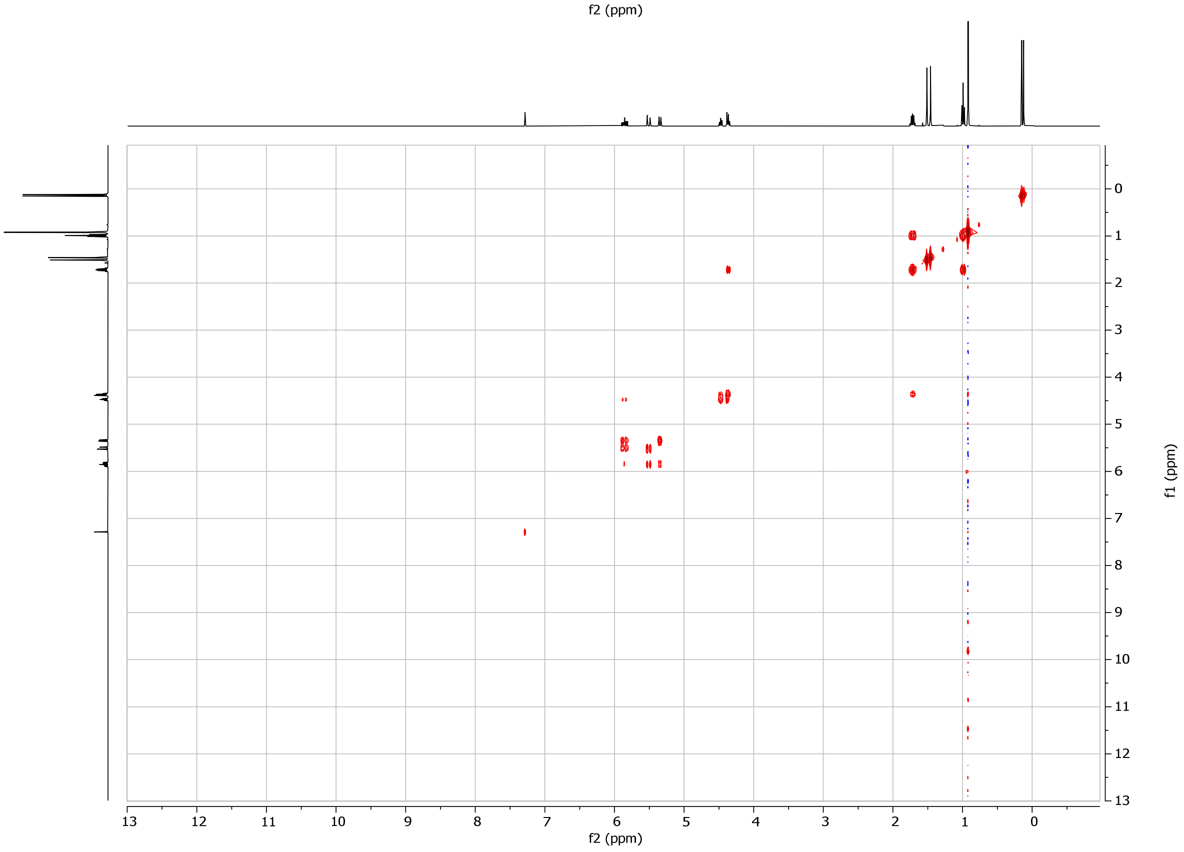
f2 (ppm)
f1 (ppm)

Synthesis of 17 (^{1}H, ^{13}C, HSQC, HMBC, COSY spectra)

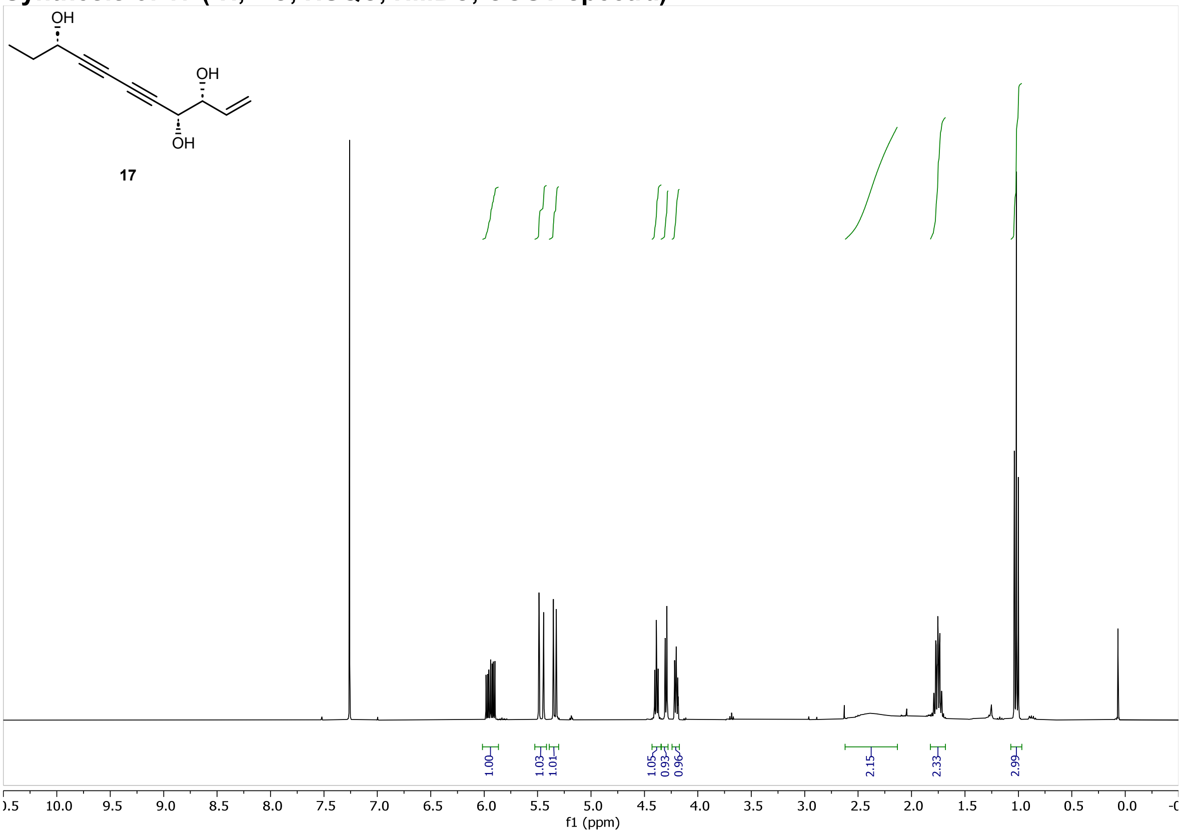

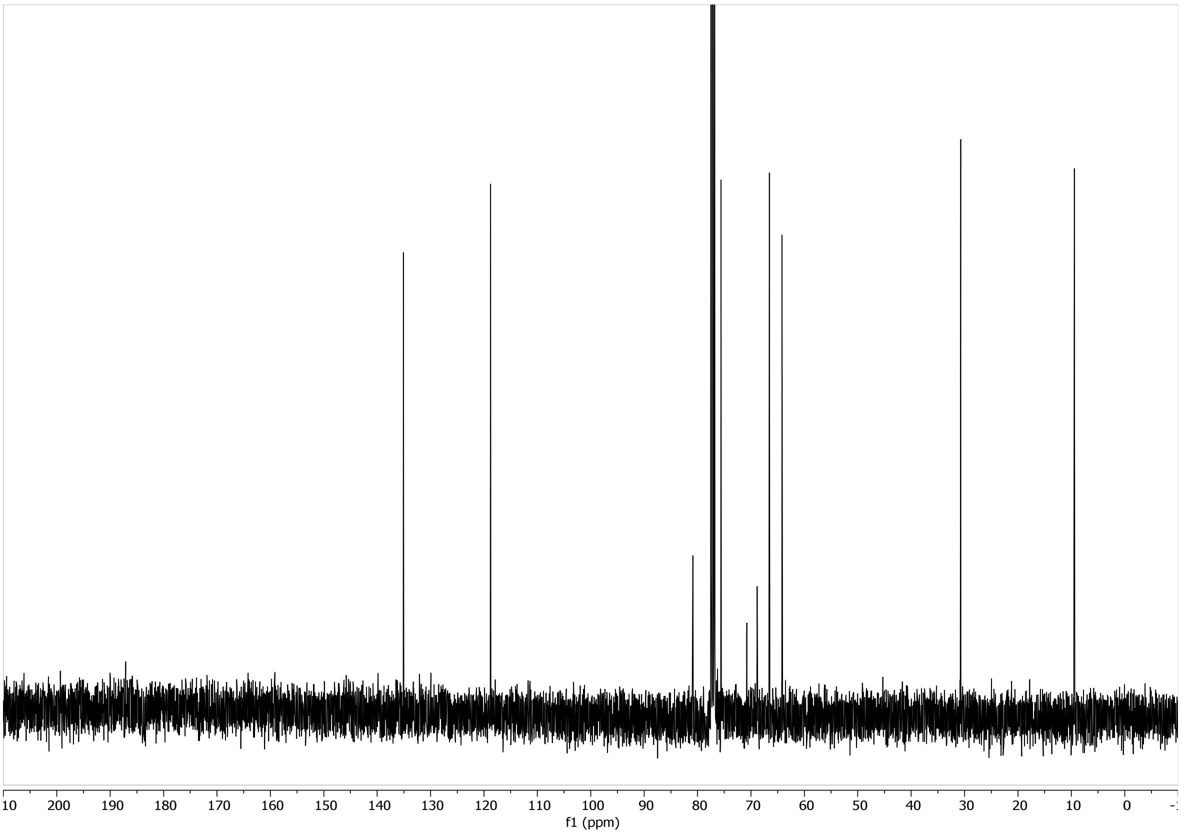
10 200 190 180 170 160 150 140 130 120 110 100 90 80 70 60 50 40 30 20 10 0
f1 (ppm)

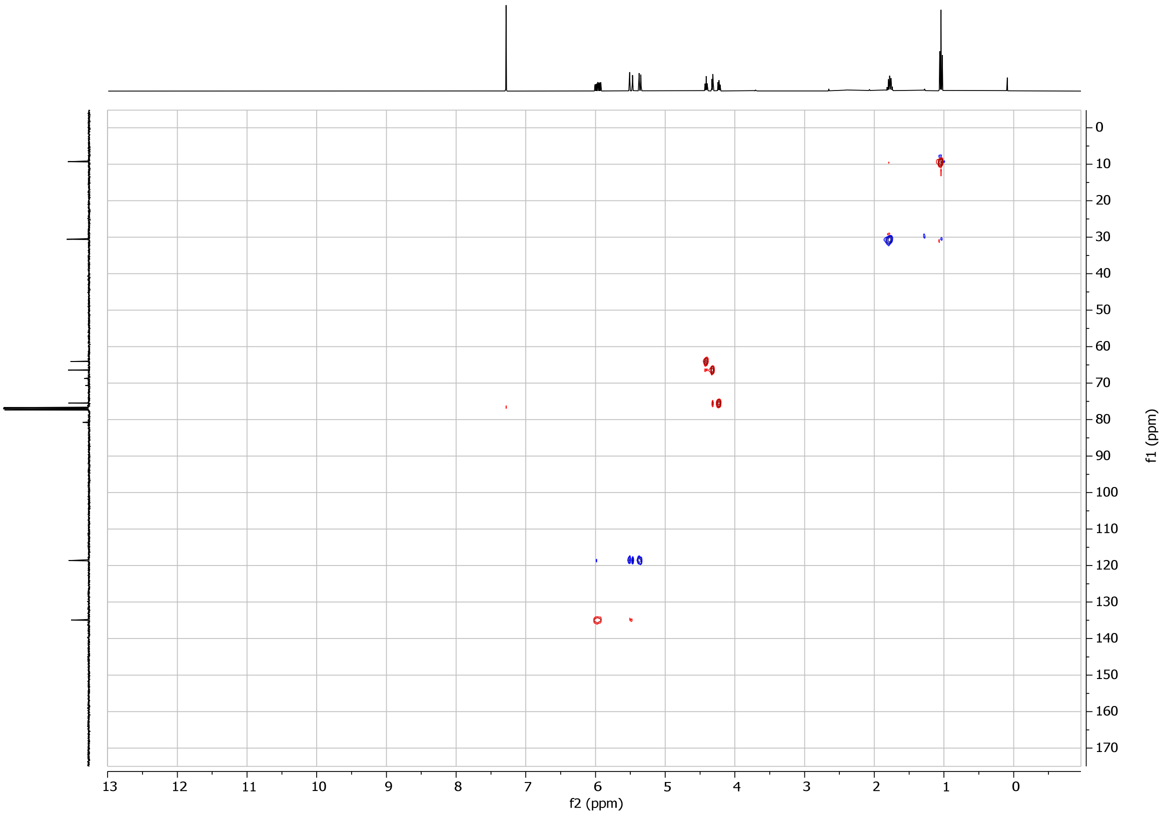
0 10 20 30 40 50 60 70 80 90 100 110 120 130 140 150 160 170
f1 (ppm)
13 12 11 10 9 8 7 6 5 4 3 2 1 0
f2 (ppm)

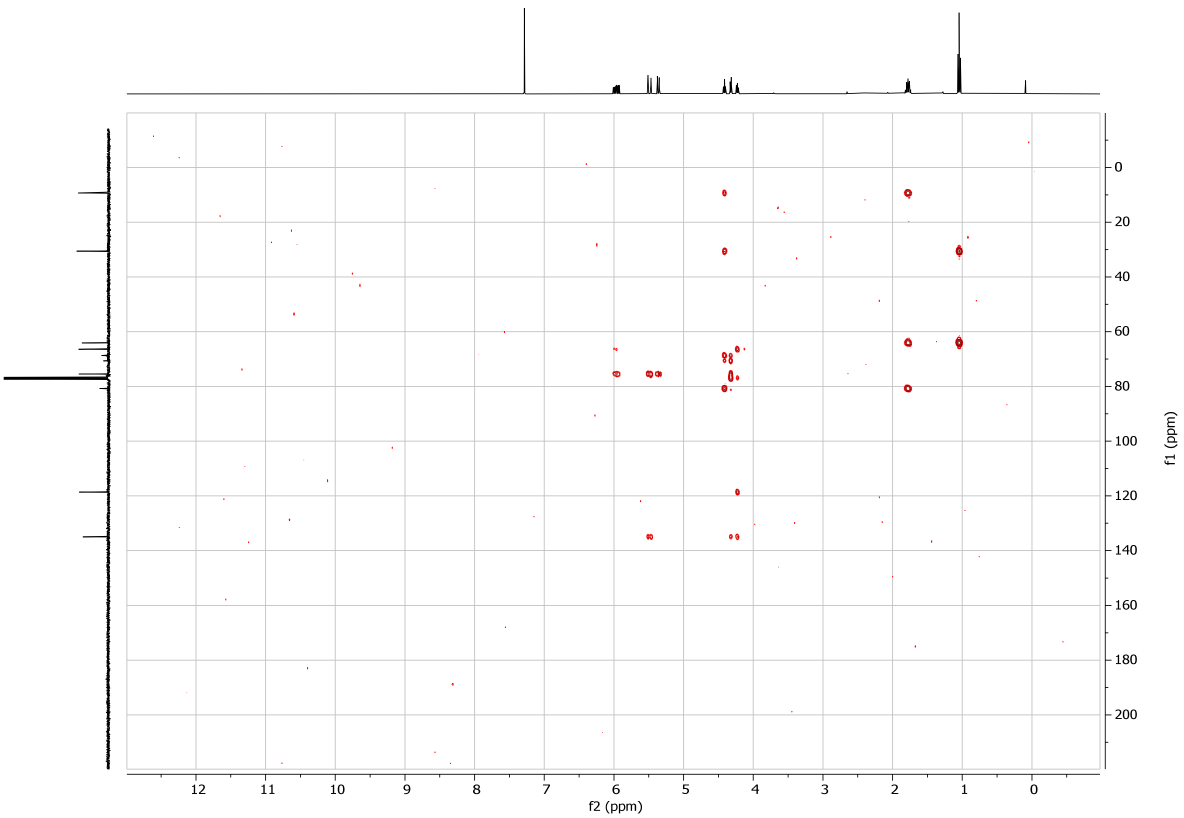

f2 (ppm)
f1 (ppm)

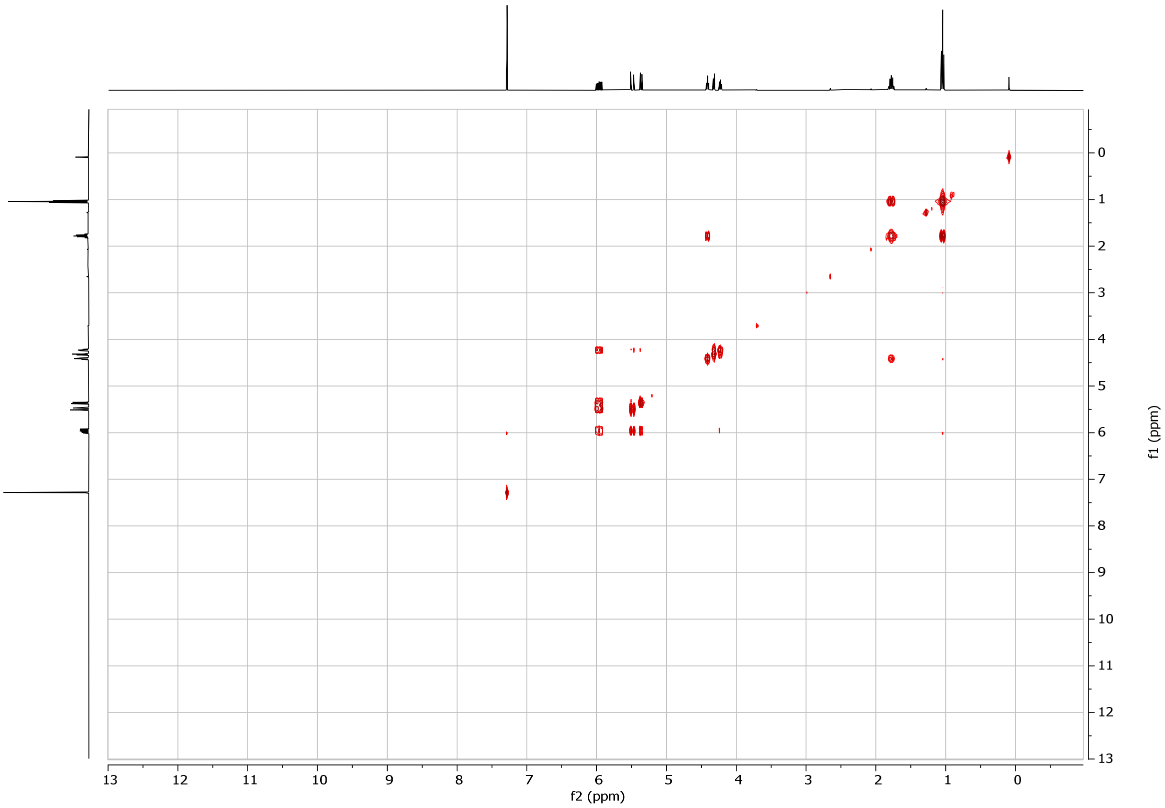

f2 (ppm)
f1 (ppm)

Synthesis of 19 (^{1}H, ^{13}C, HSQC, HMBC, COSY spectra)

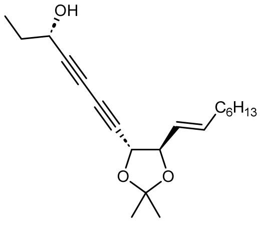

19

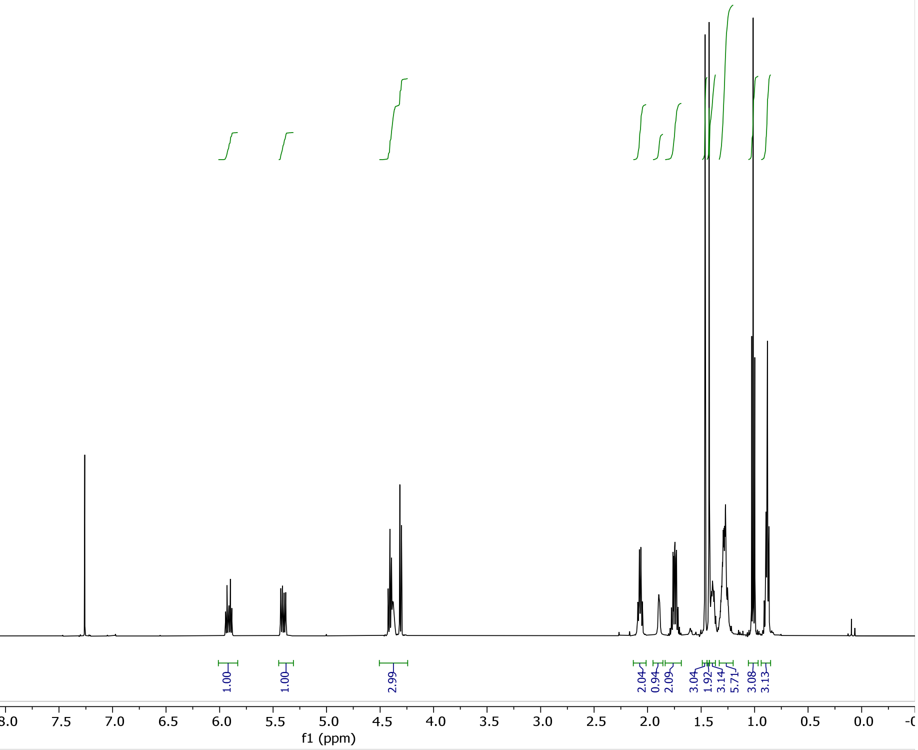

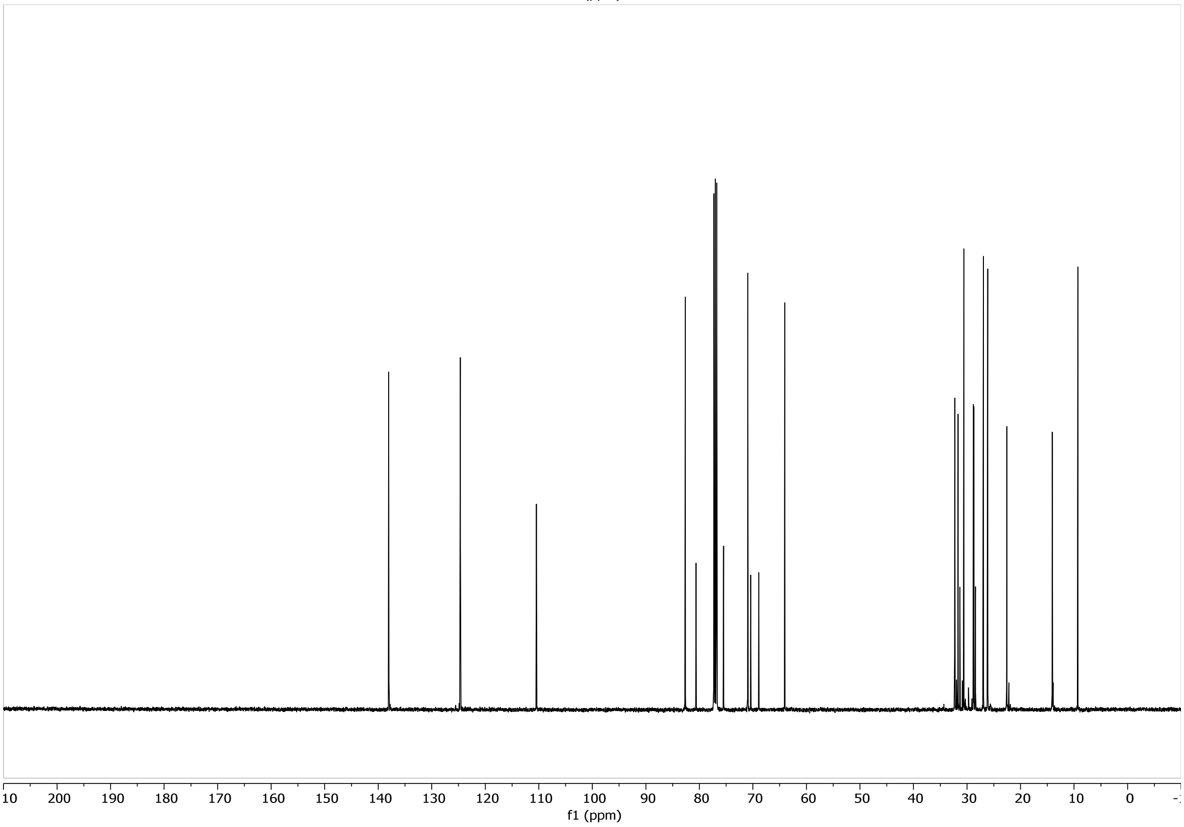

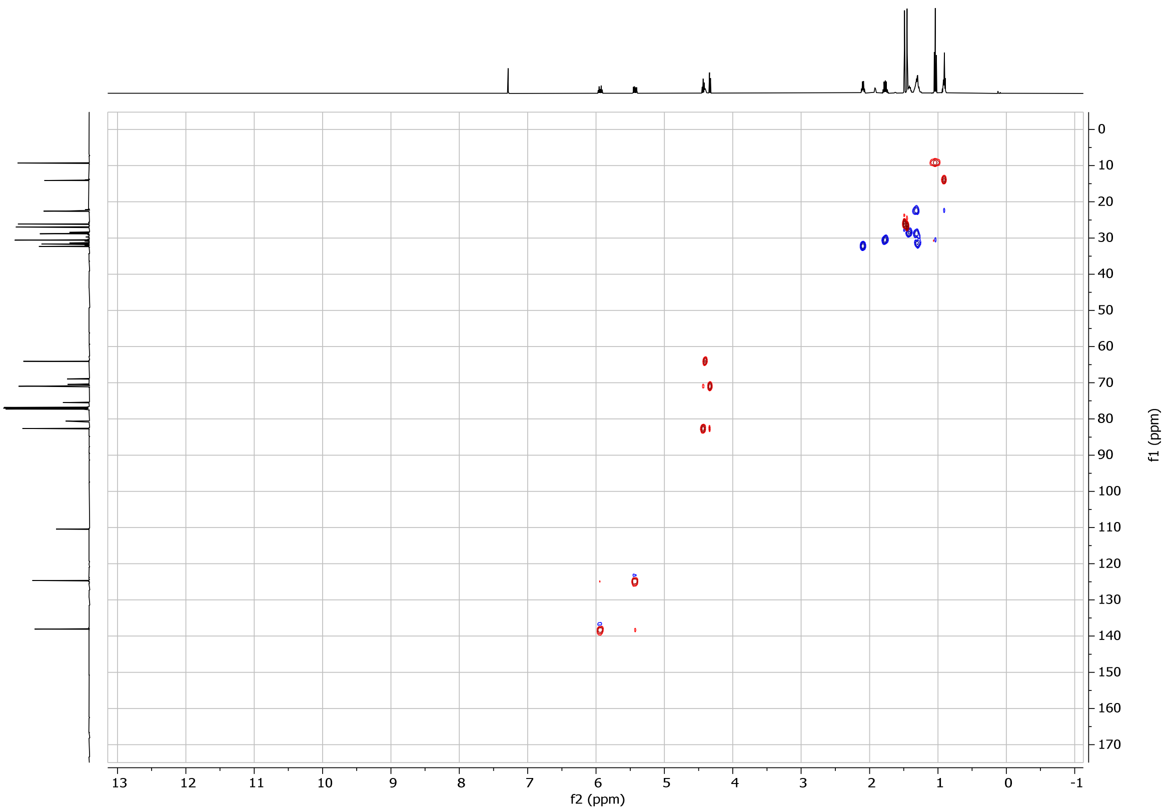

f1 (ppm)
f2 (ppm)

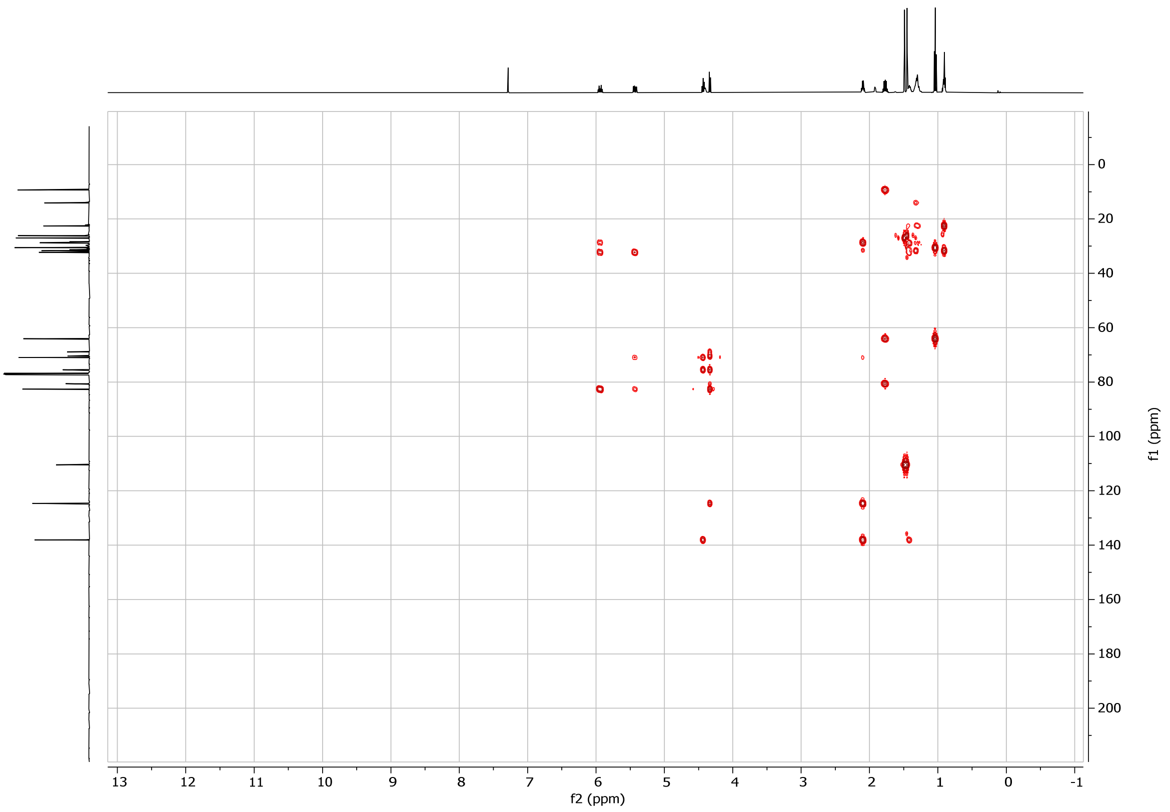

f1 (ppm)
f2 (ppm)

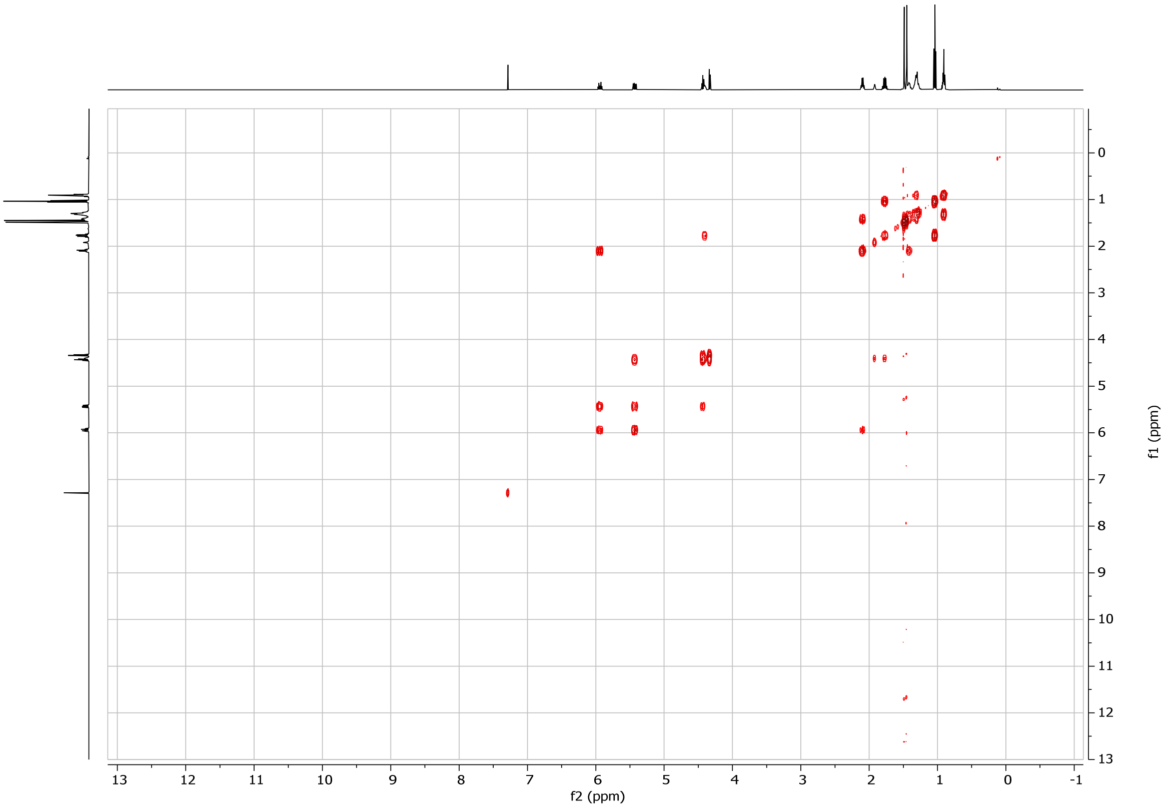

f2 (ppm)
f1 (ppm)

Synthesis of S16 (^{1}H, ^{13}C, HSQC, HMBC, COSY spectra)

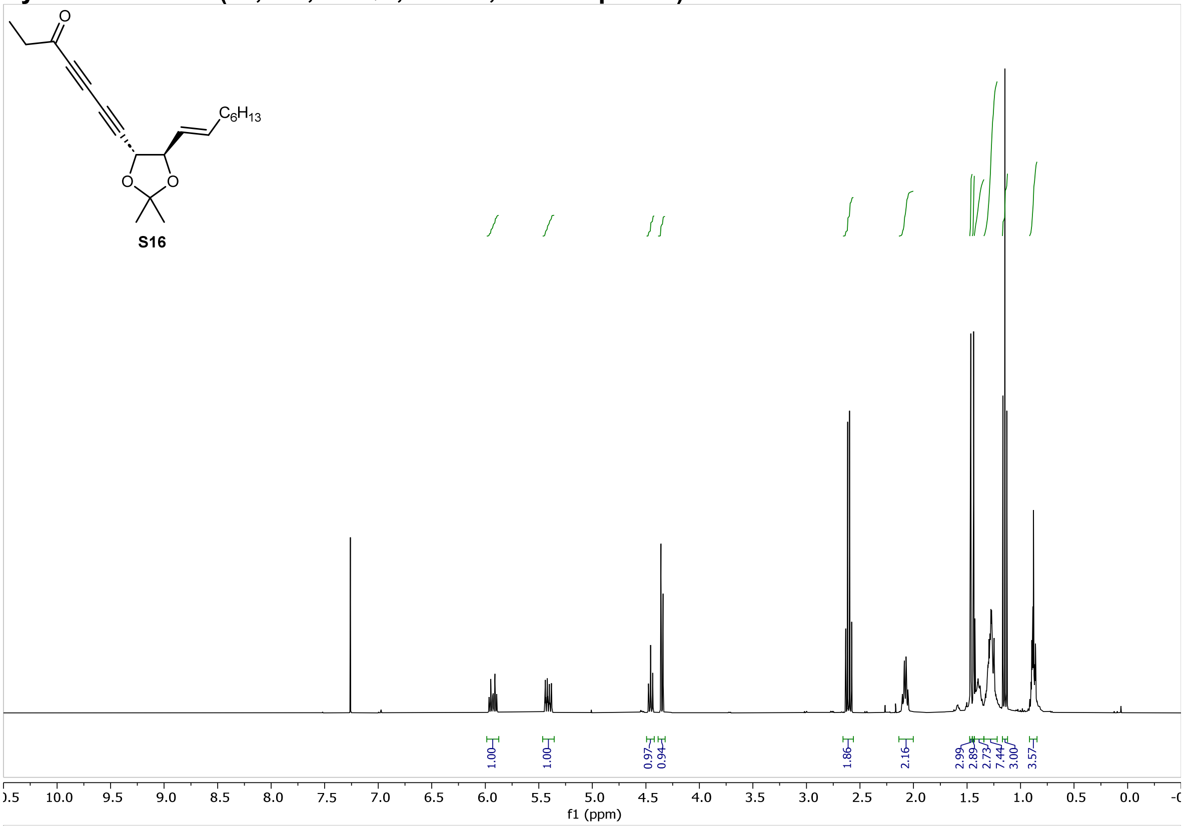

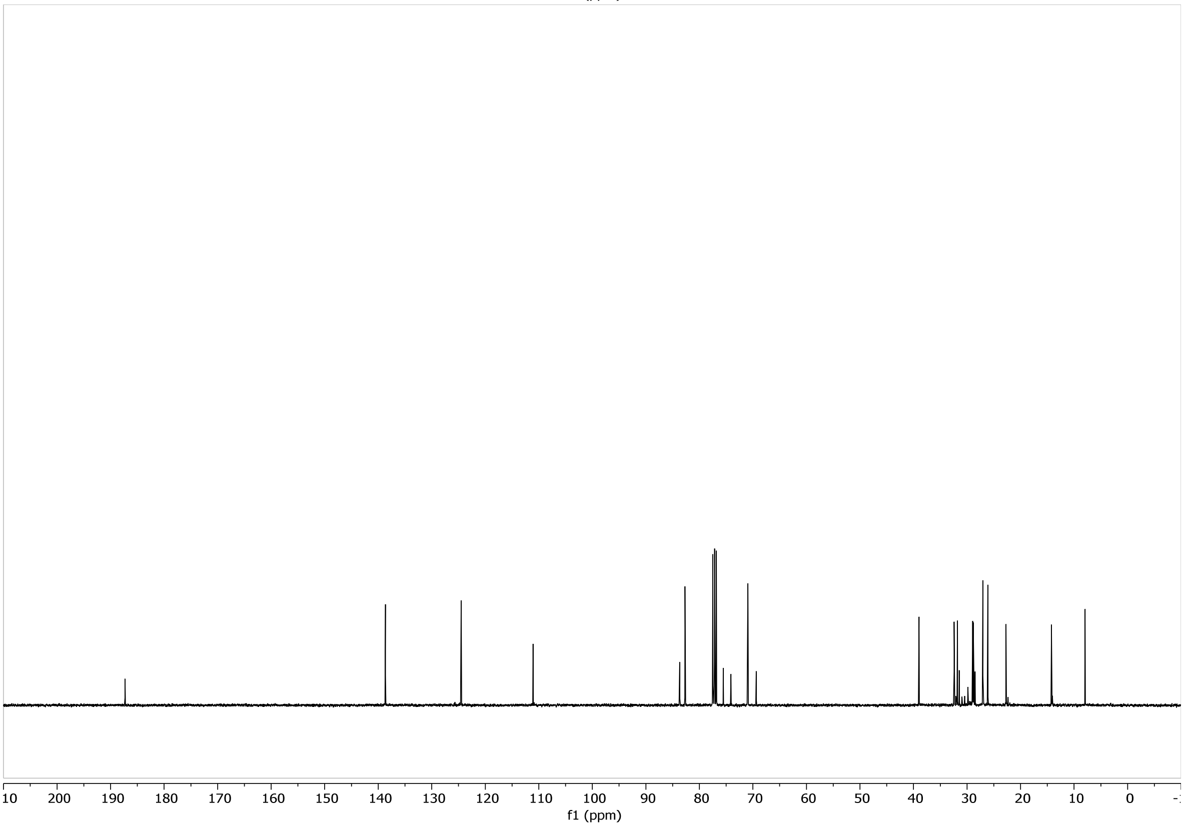

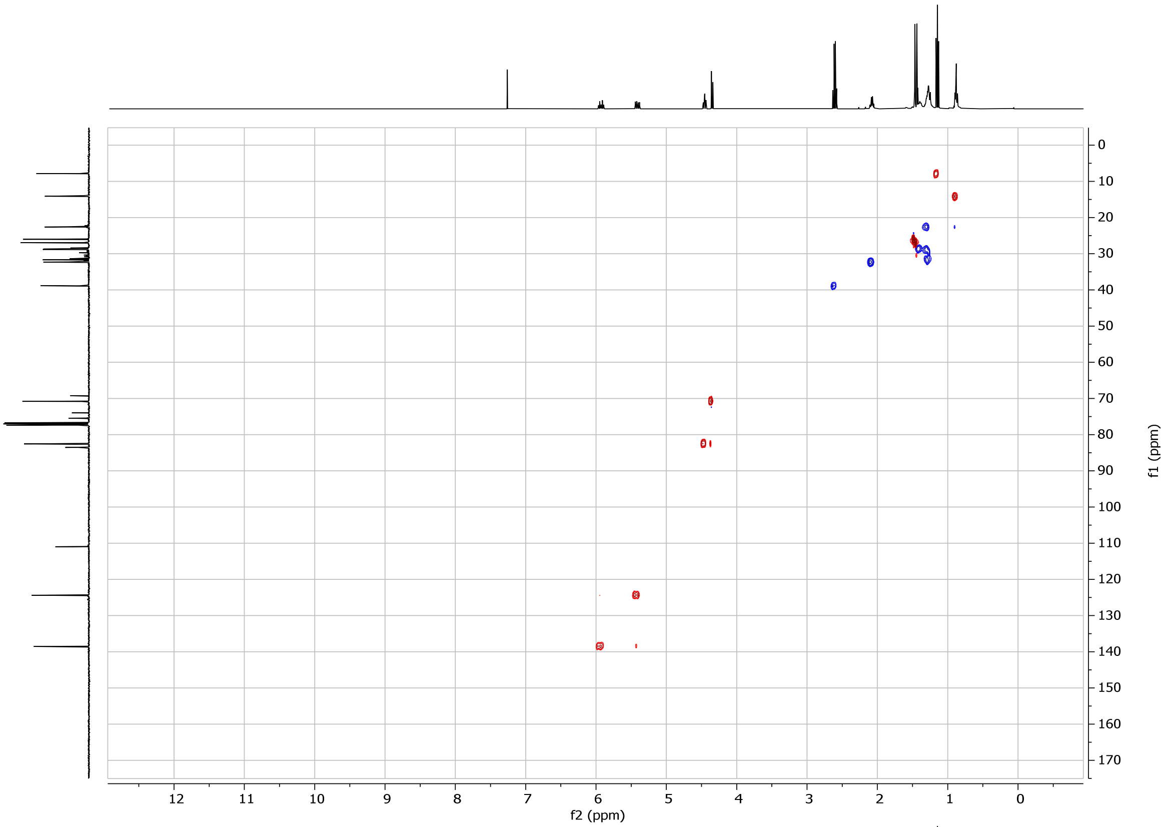

f2 (ppm)
f1 (ppm)

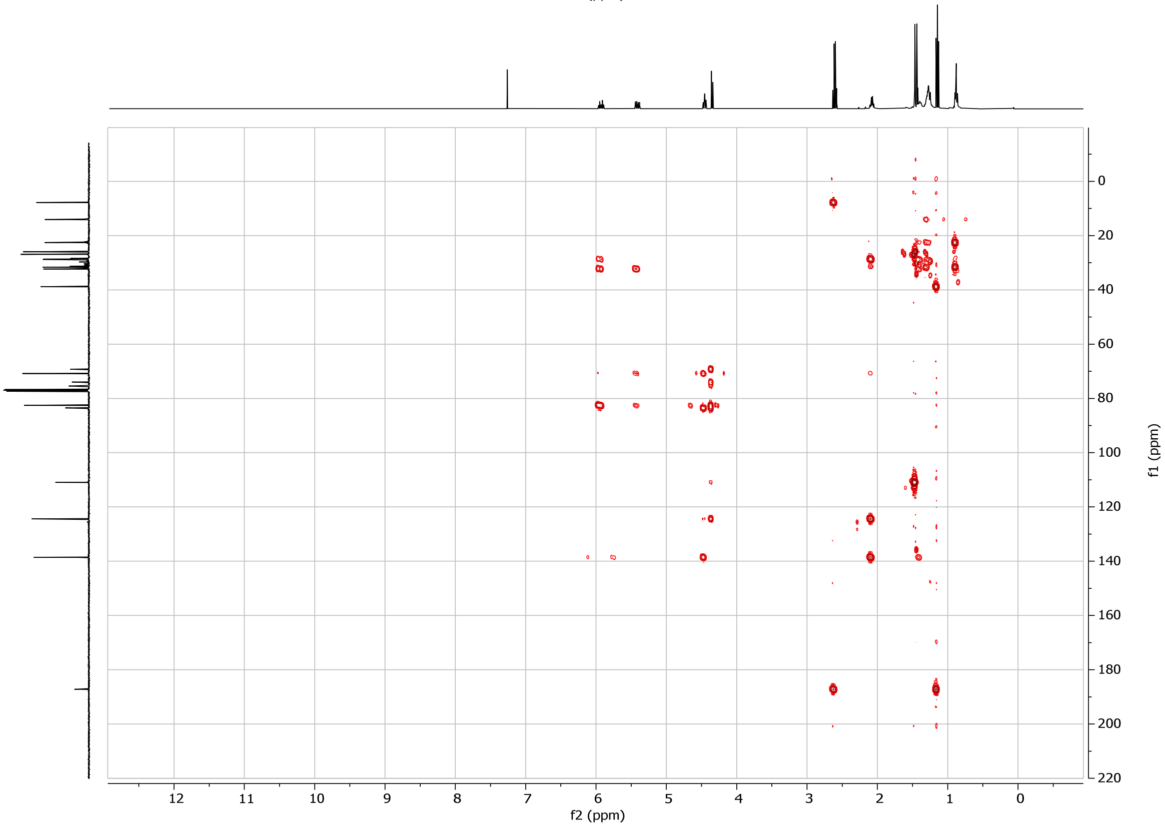

f2 (ppm)
f1 (ppm)

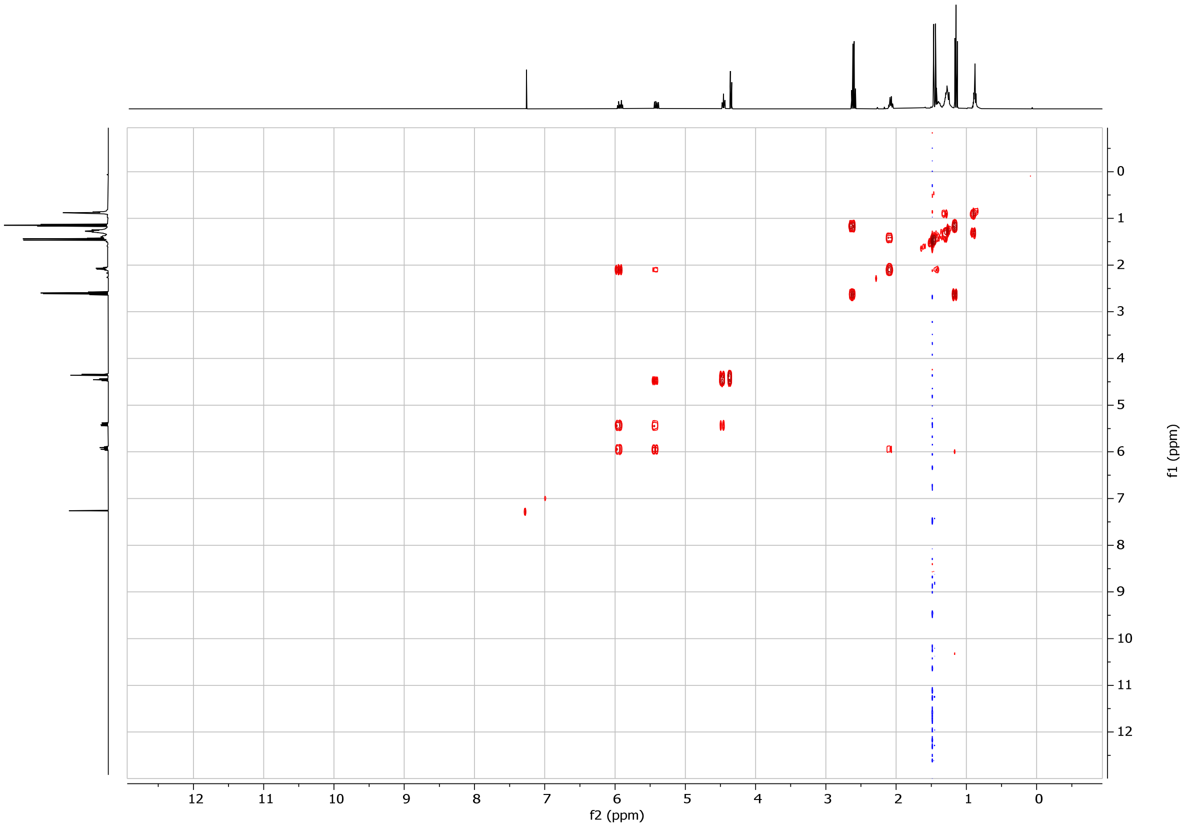

12
11
10
9
8
7
6
5
4
3
2
1
0
f2 (ppm)
f1 (ppm)

Synthesis of 18 (^{1}H, ^{13}C, HSQC, HMBC, COSY spectra)

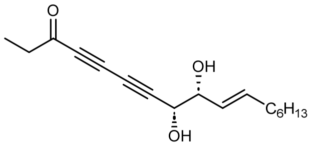

18

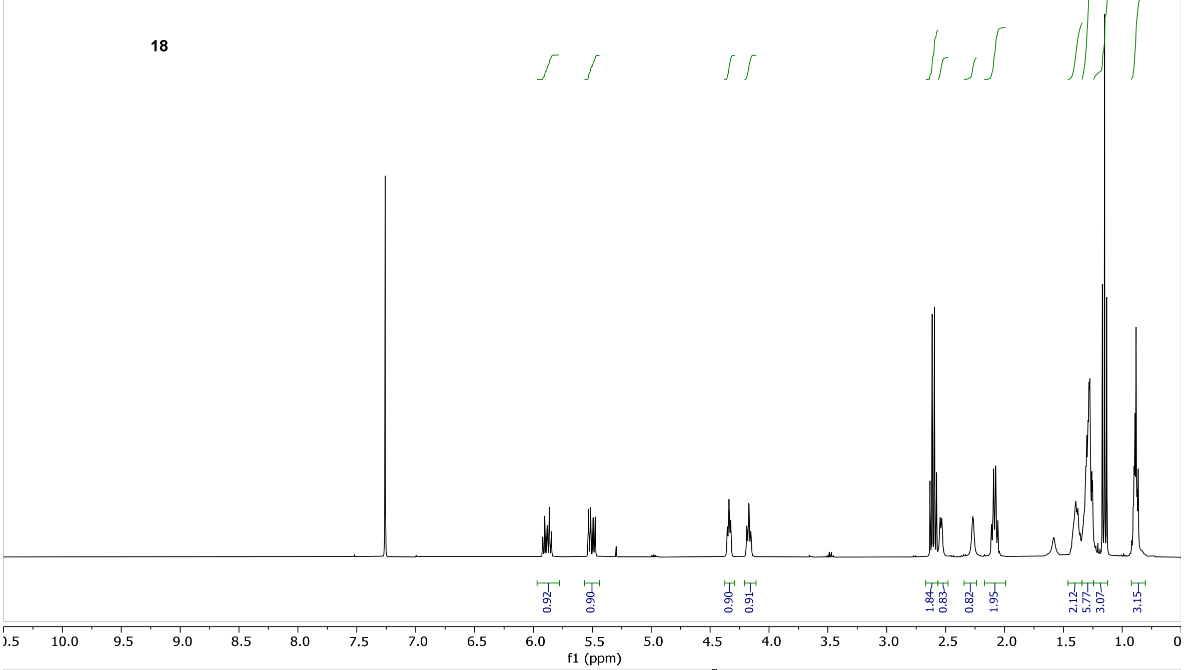

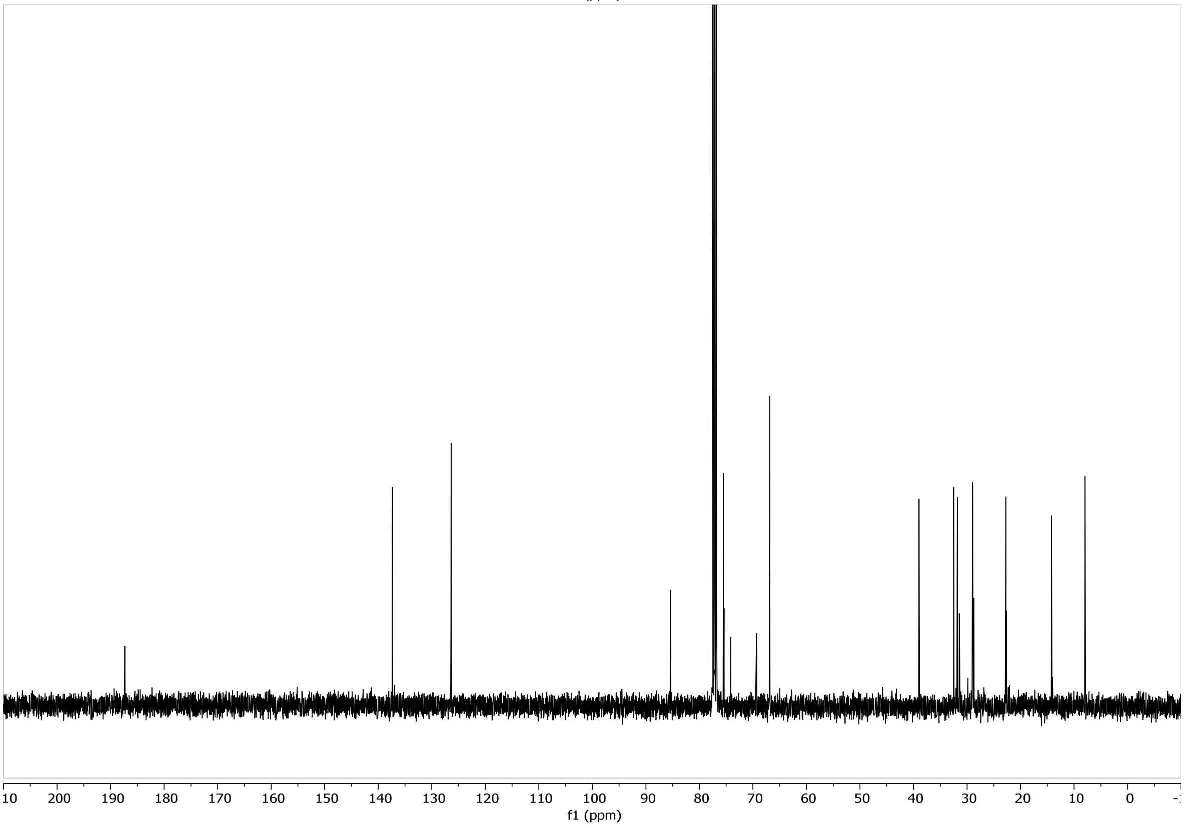

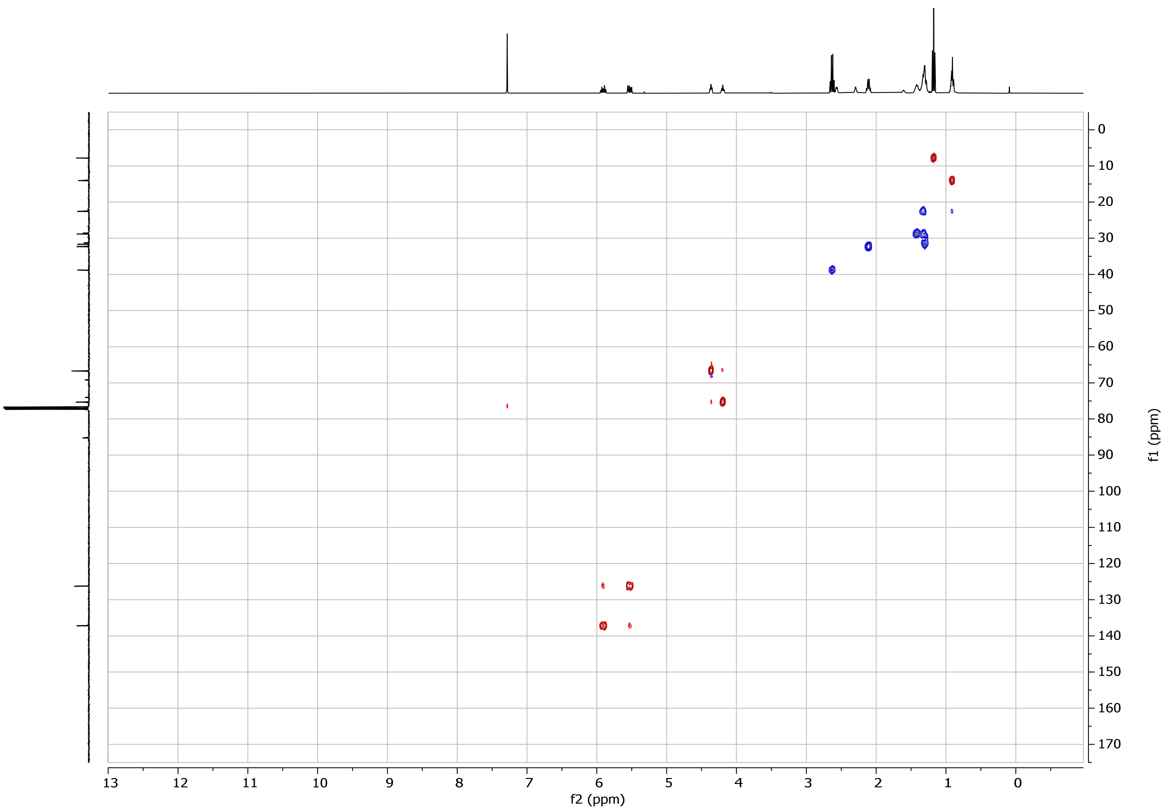

f2 (ppm)
f1 (ppm)

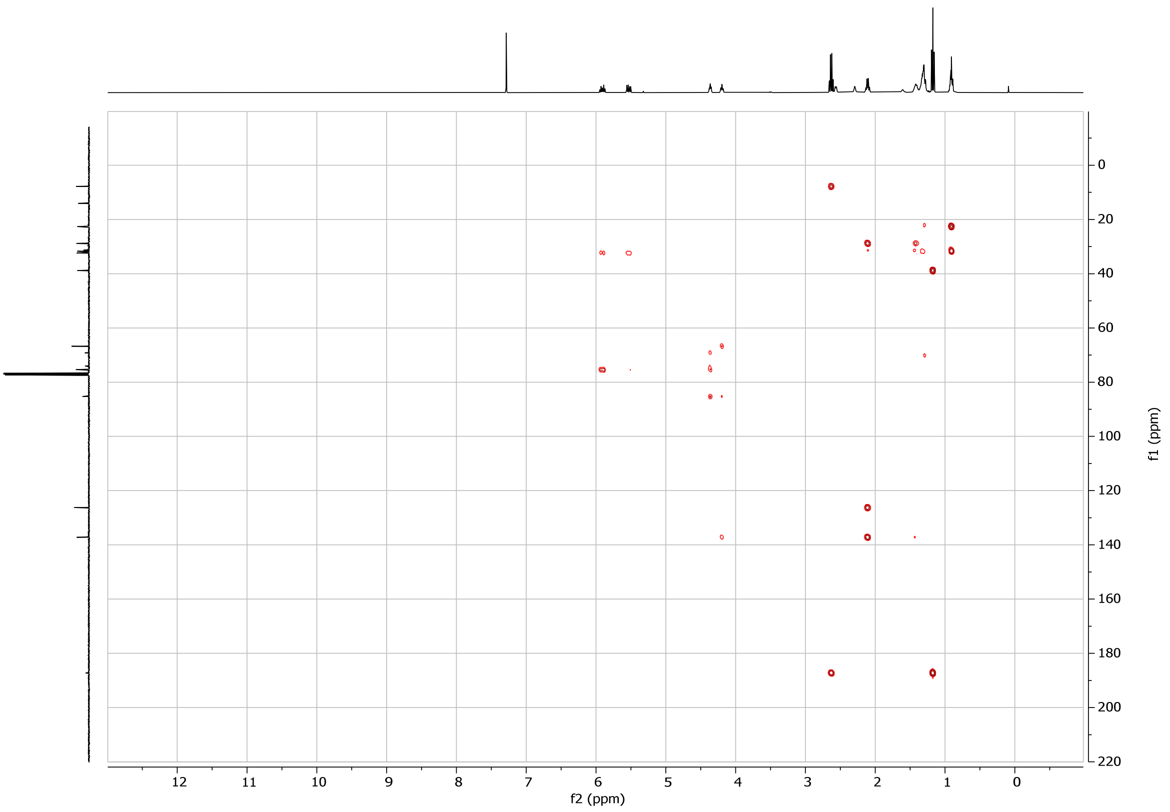

f2 (ppm)
f1 (ppm)

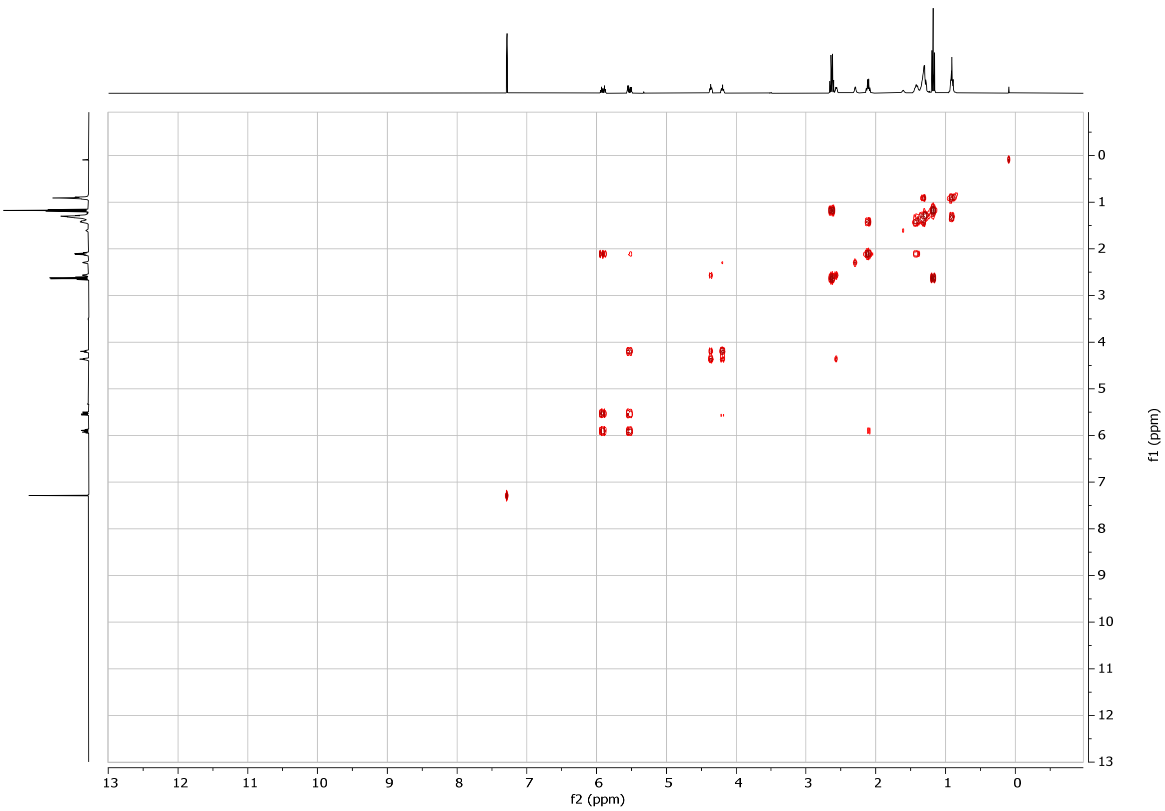
f2 (ppm)
f1 (ppm)

Synthesis of S17 (^{1}H, ^{13}C spectra)

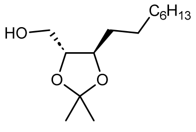

S17

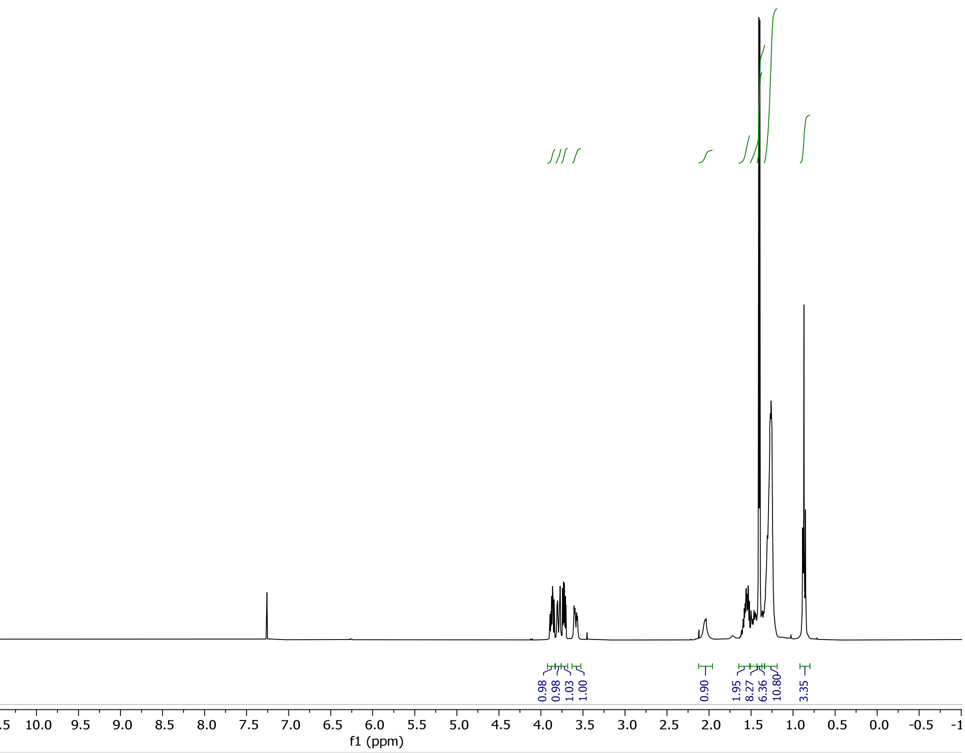

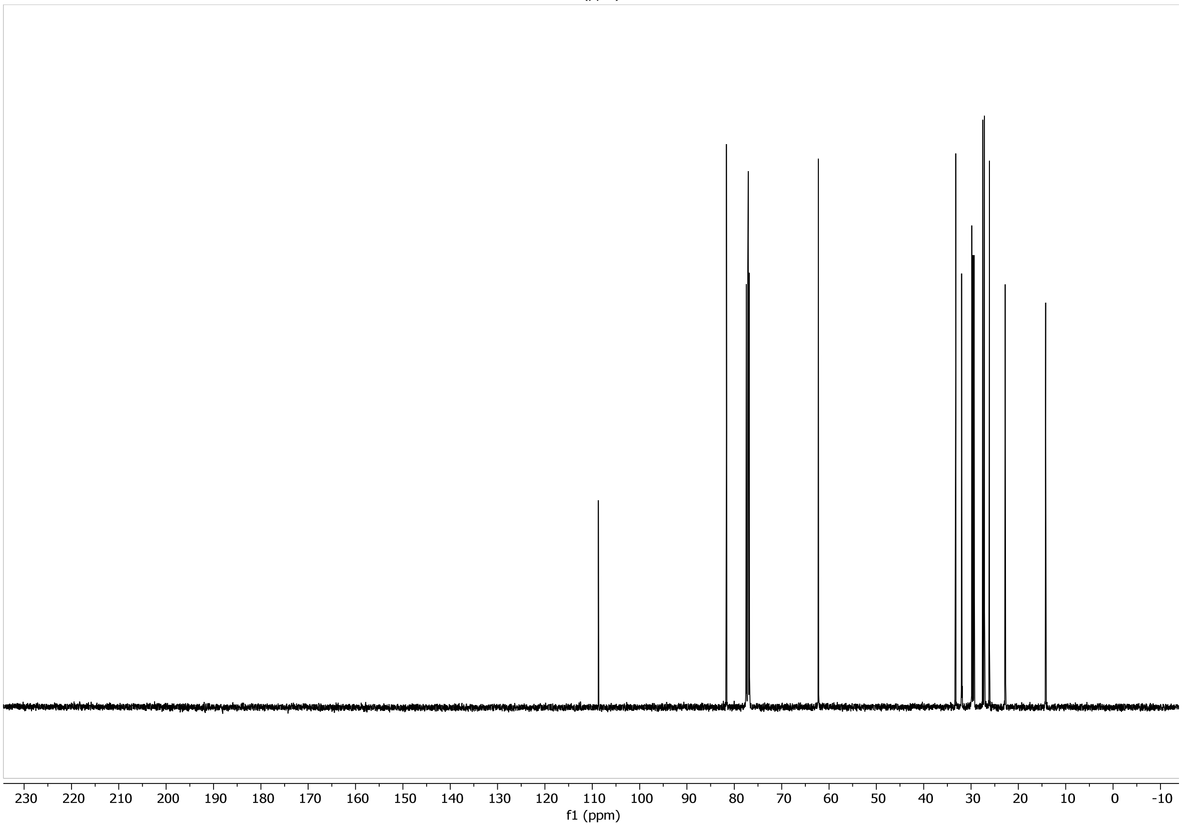

Synthesis of S18 (^1H, ^{13}C spectra)

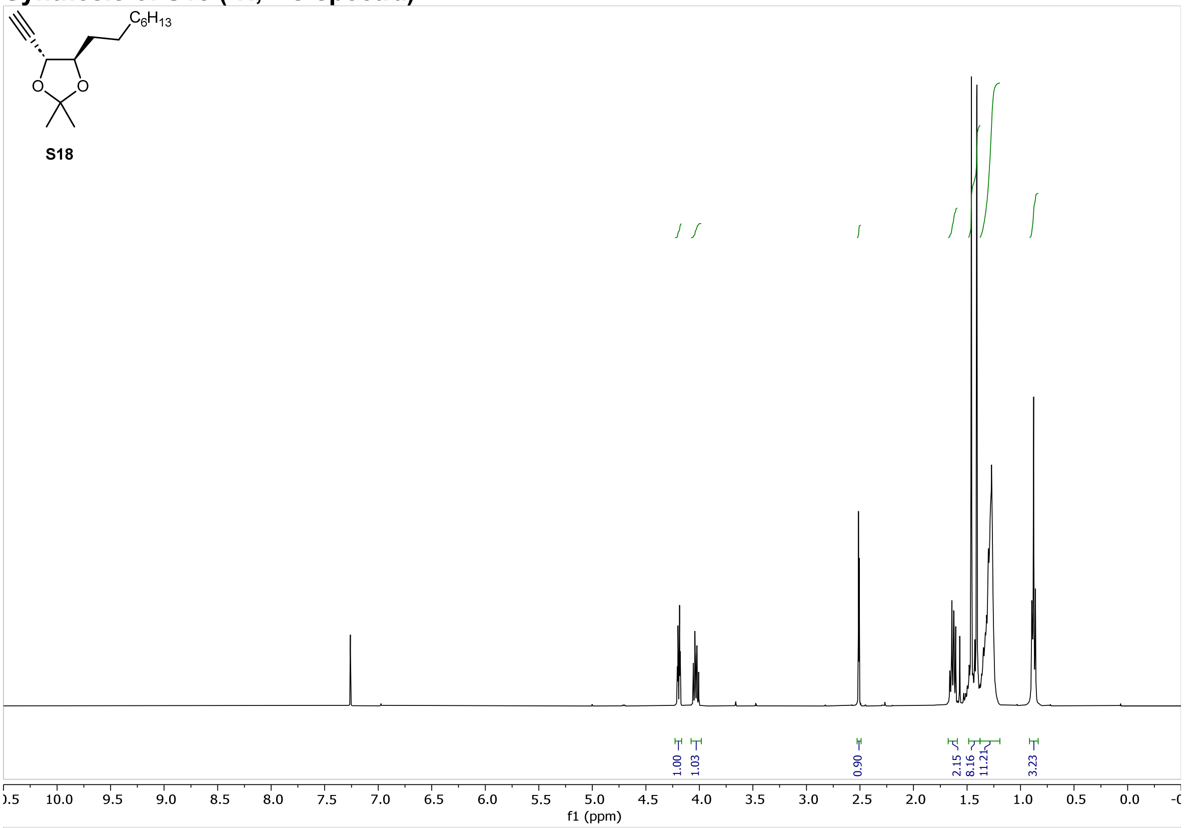

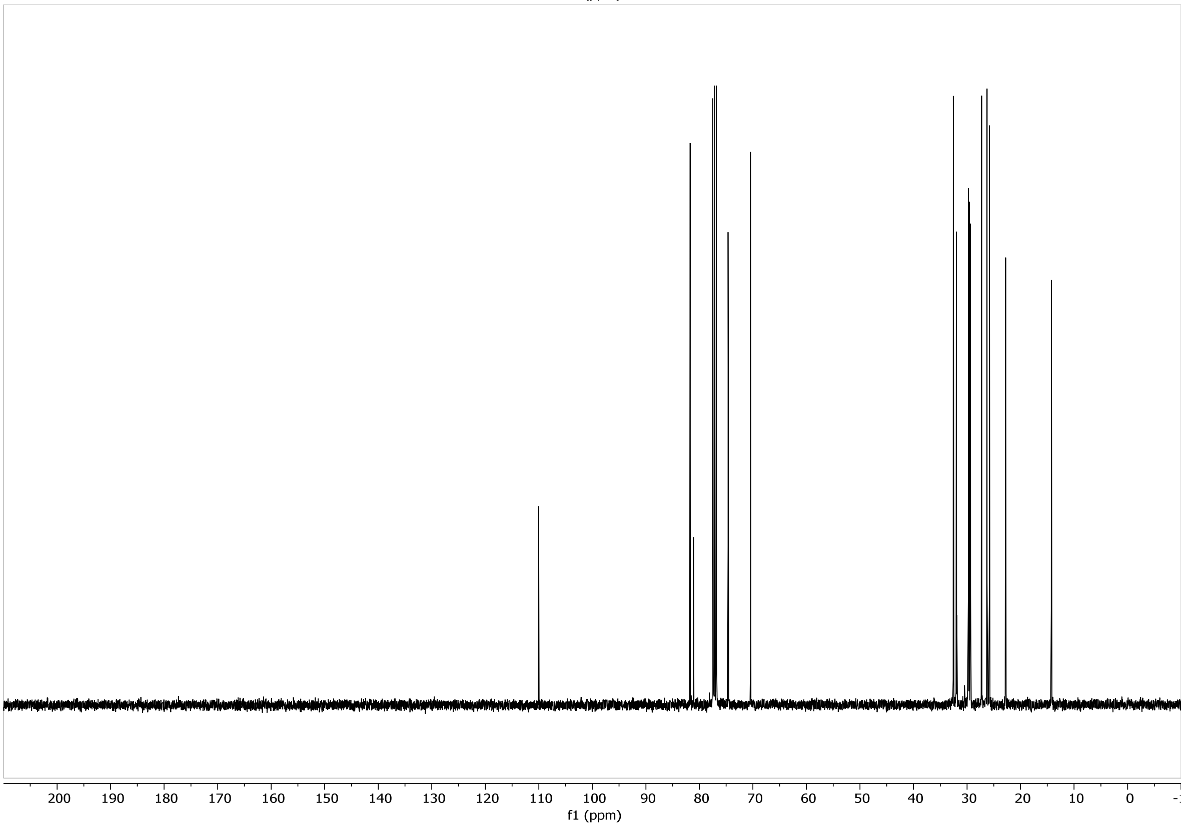

Synthesis of S19 (^{1}H, ^{13}C, HSQC, HMBC, COSY spectra)

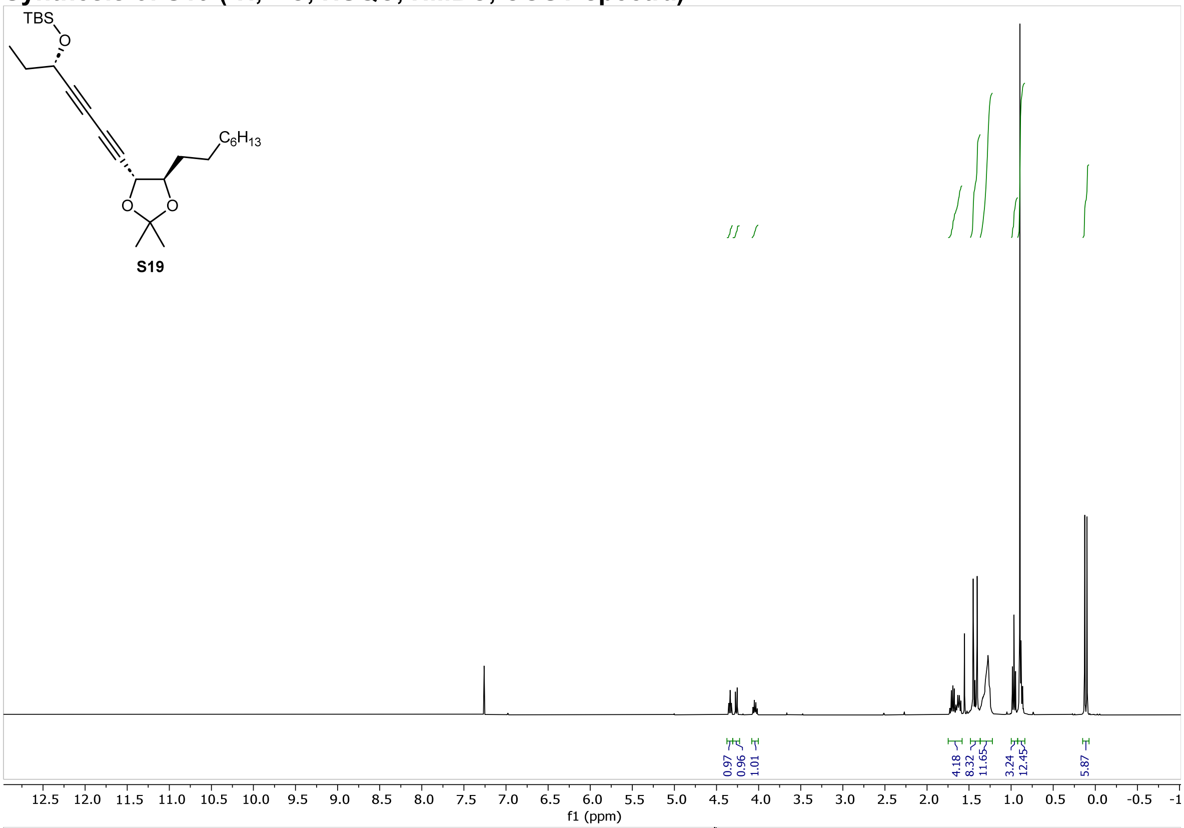

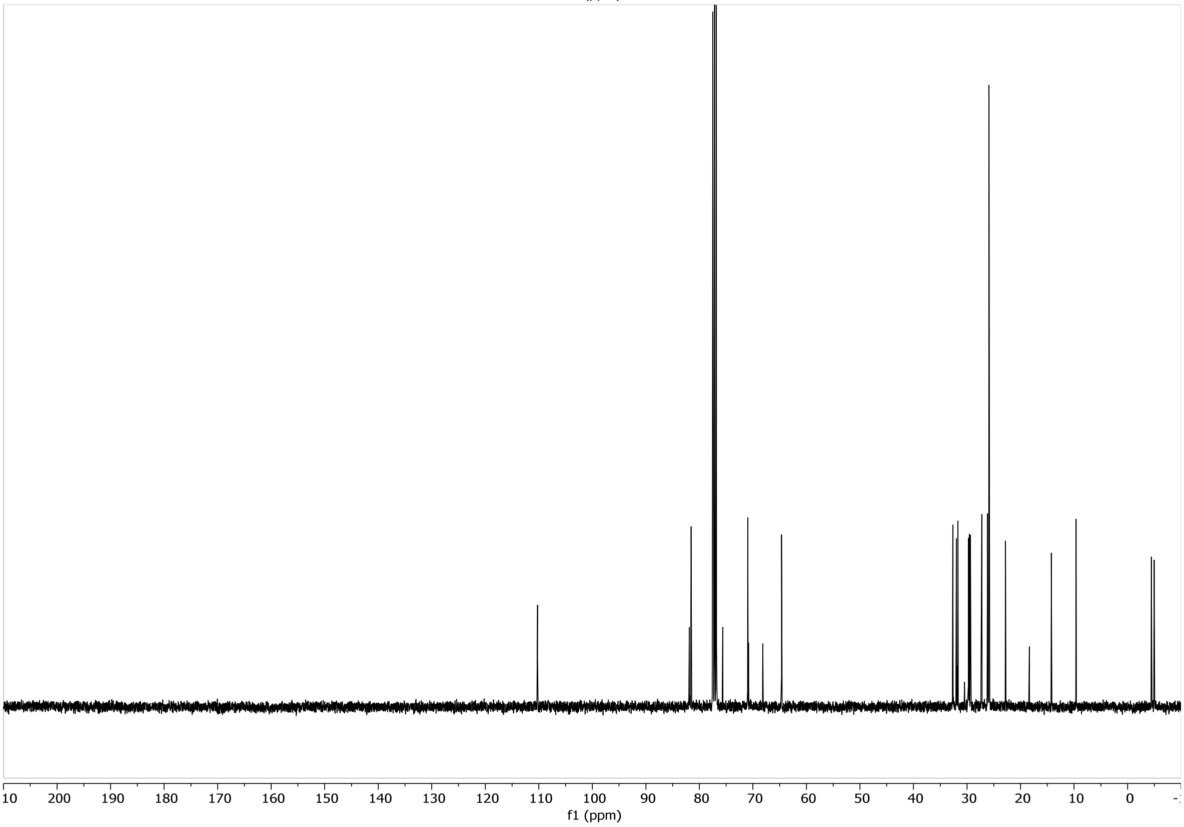

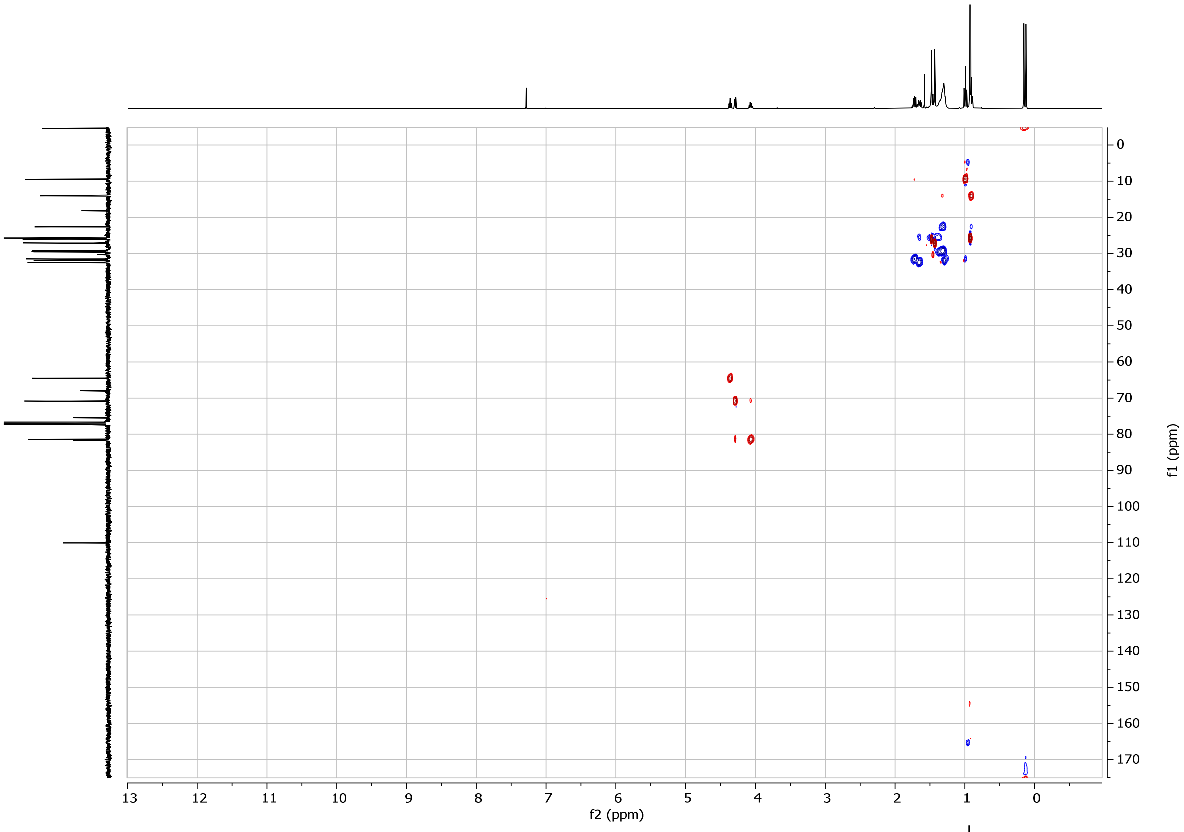

f2 (ppm)
f1 (ppm)

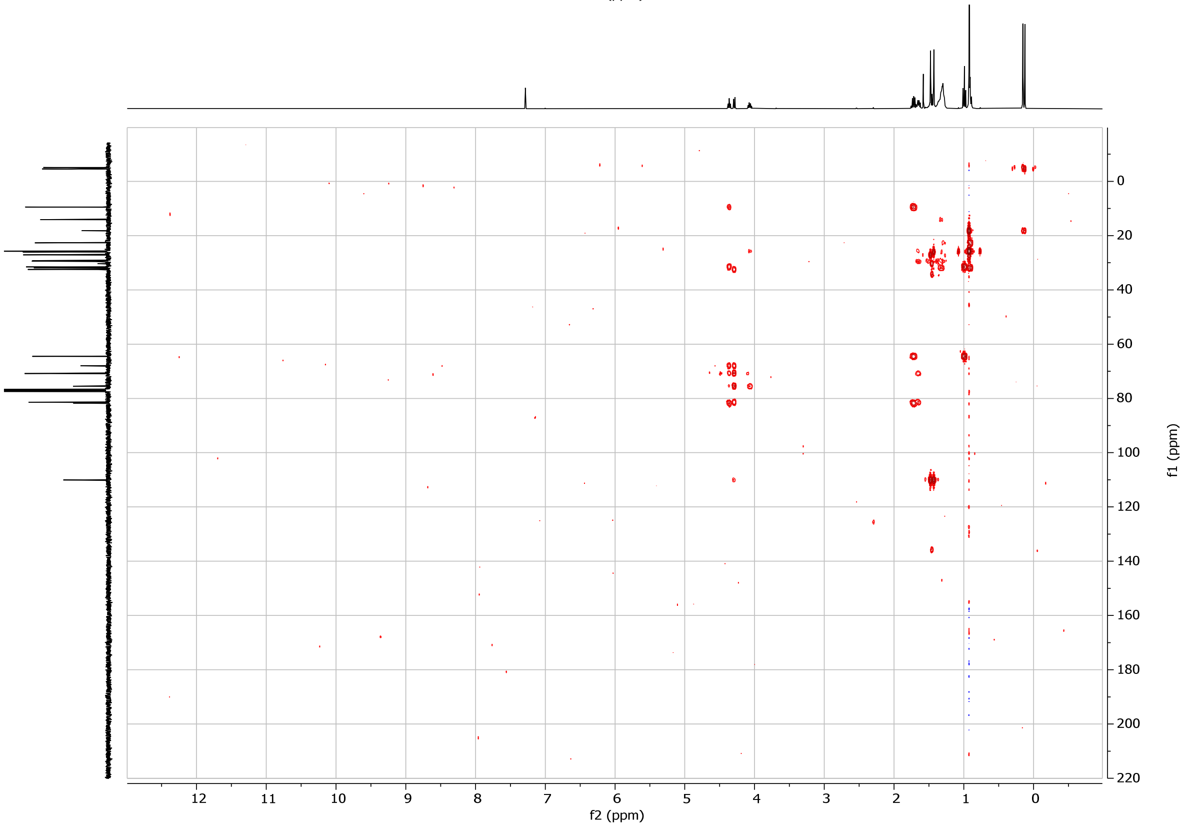

f2 (ppm)
f1 (ppm)

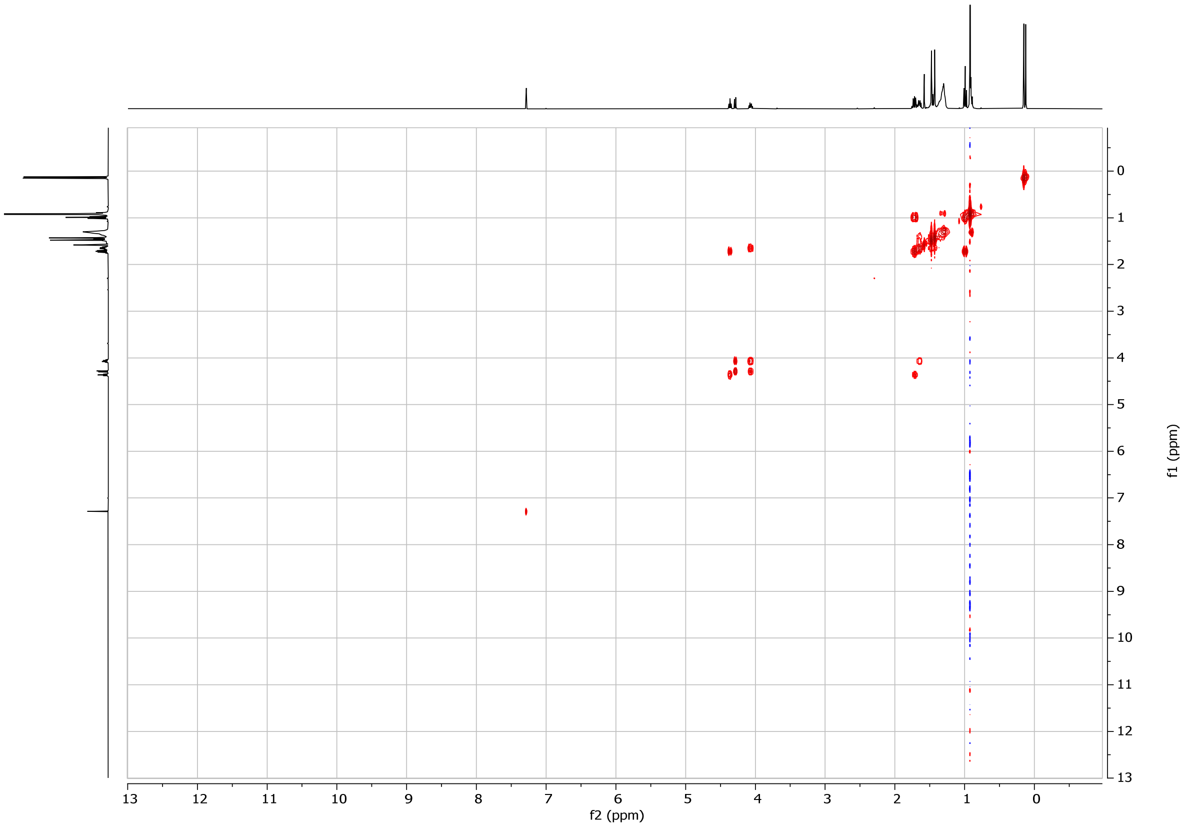

f2 (ppm)
f1 (ppm)

Synthesis of 20 (^{1}H, ^{13}C, HSQC, HMBC, COSY spectra)

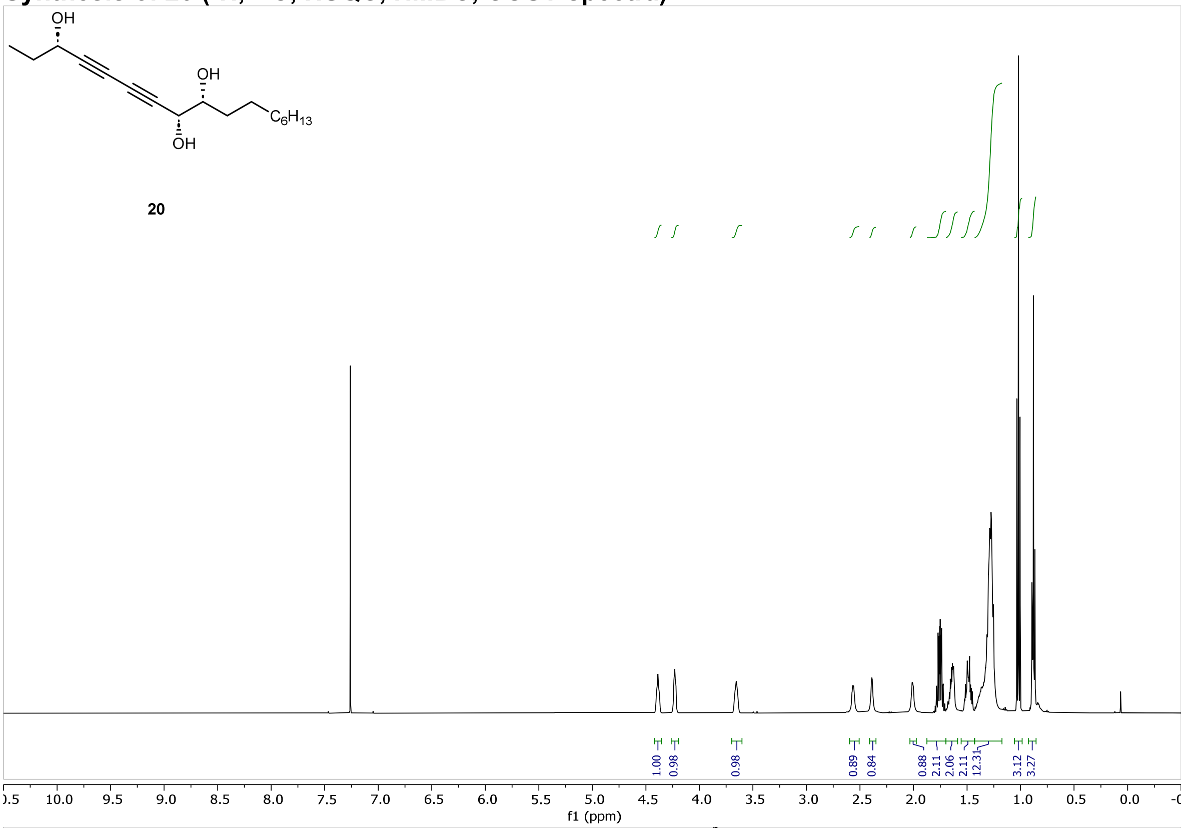

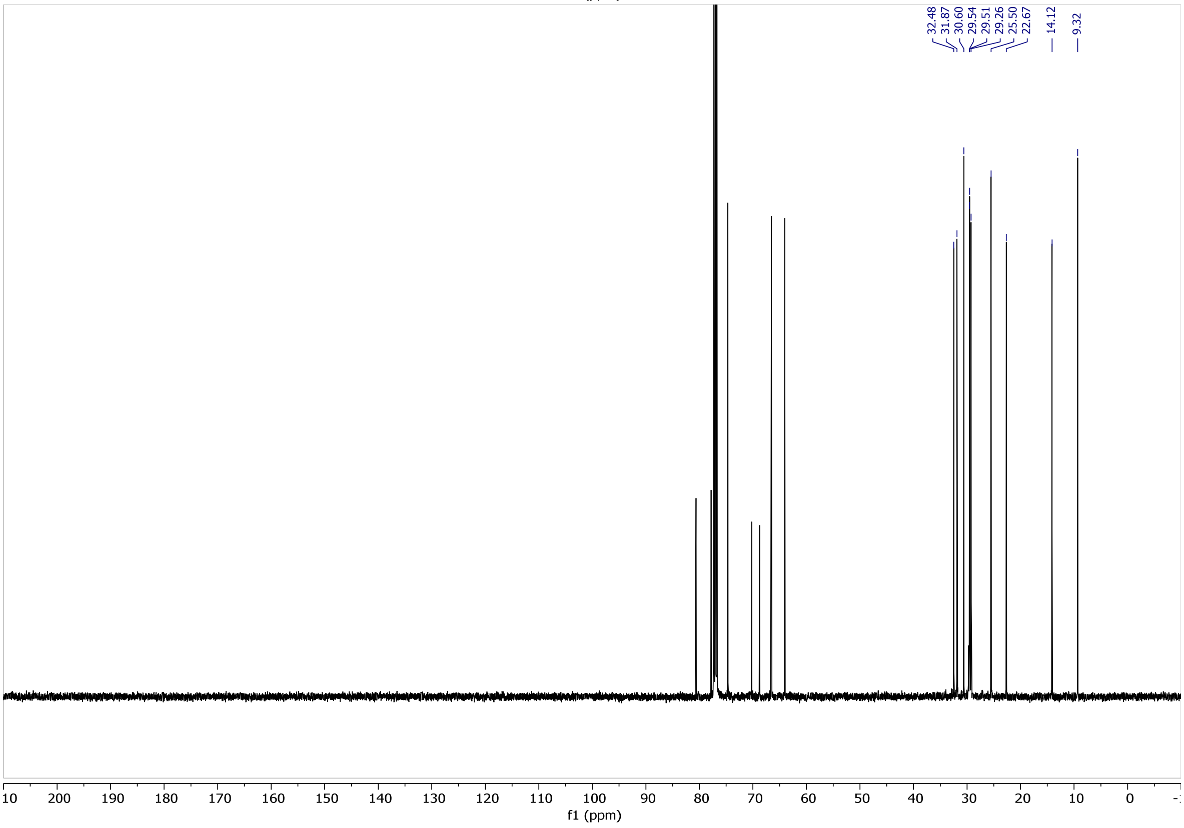

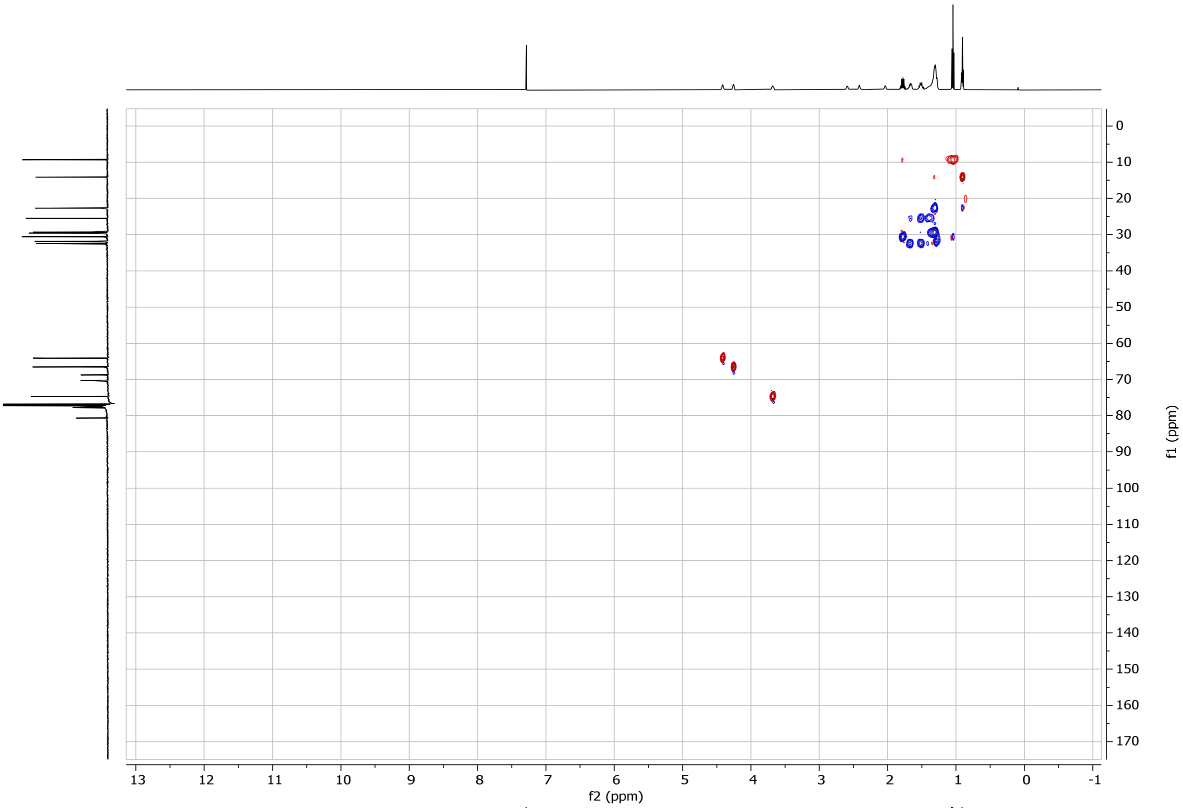
f2 (ppm)
f1 (ppm)

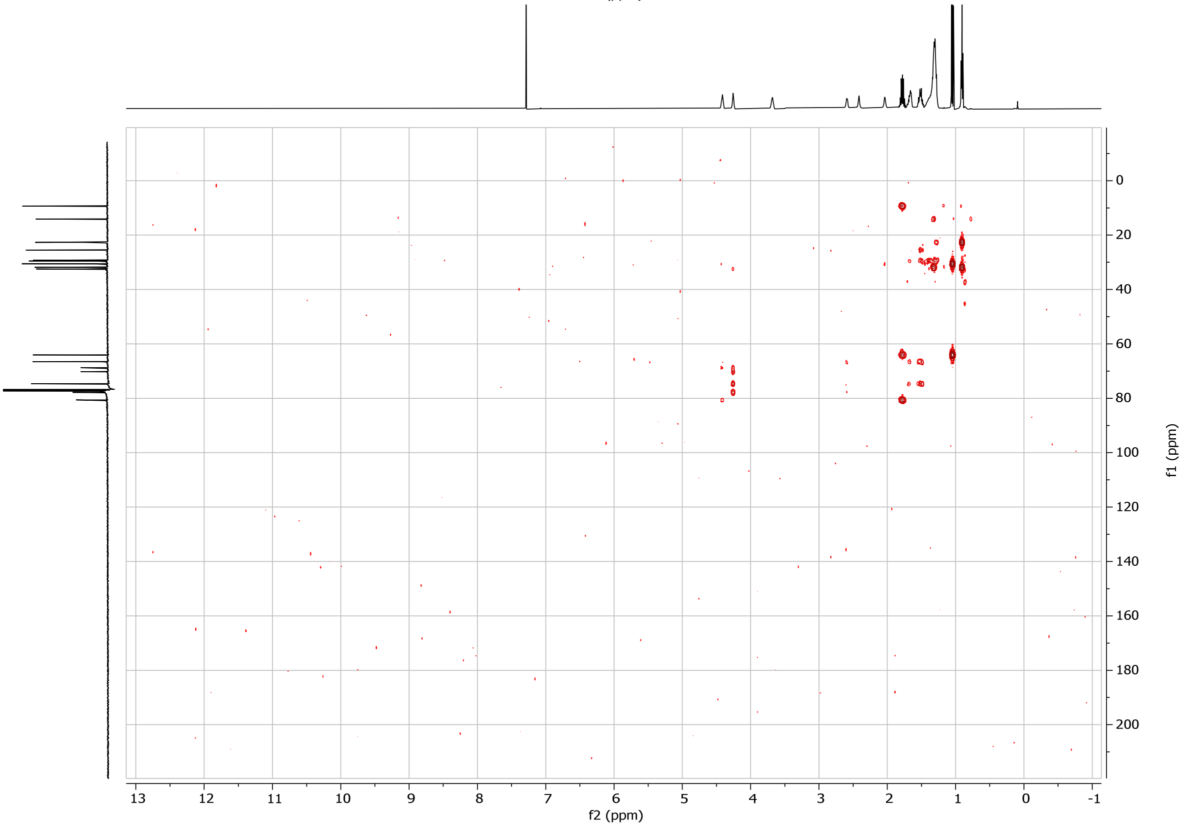
f2 (ppm)
f1 (ppm)

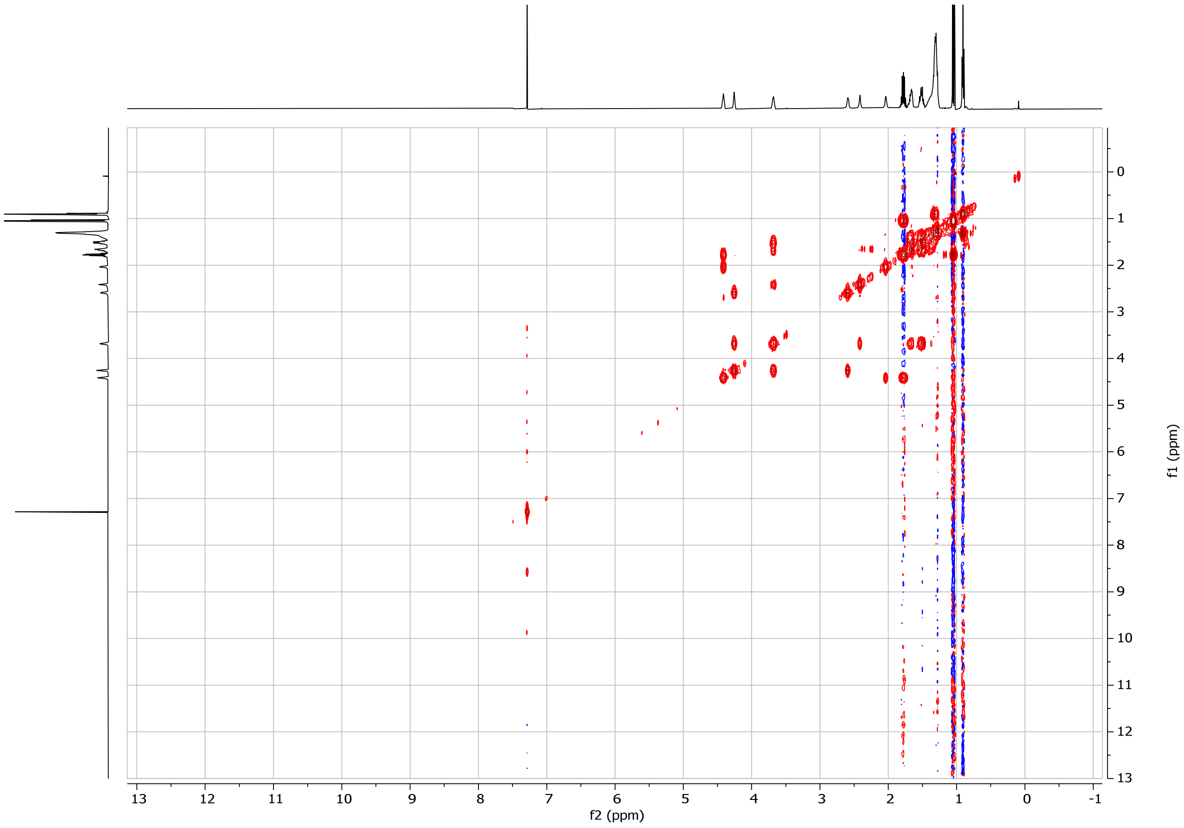

f2 (ppm)
f1 (ppm)

12 Spectra to Isofalcarintriol Derived Functional Probes

13 Synthesis of Biotin Labeled Isofalcarintriol

Synthesis of S12 (^{1}H, ^{13}C, HSQC, HMBC, COSY spectra)

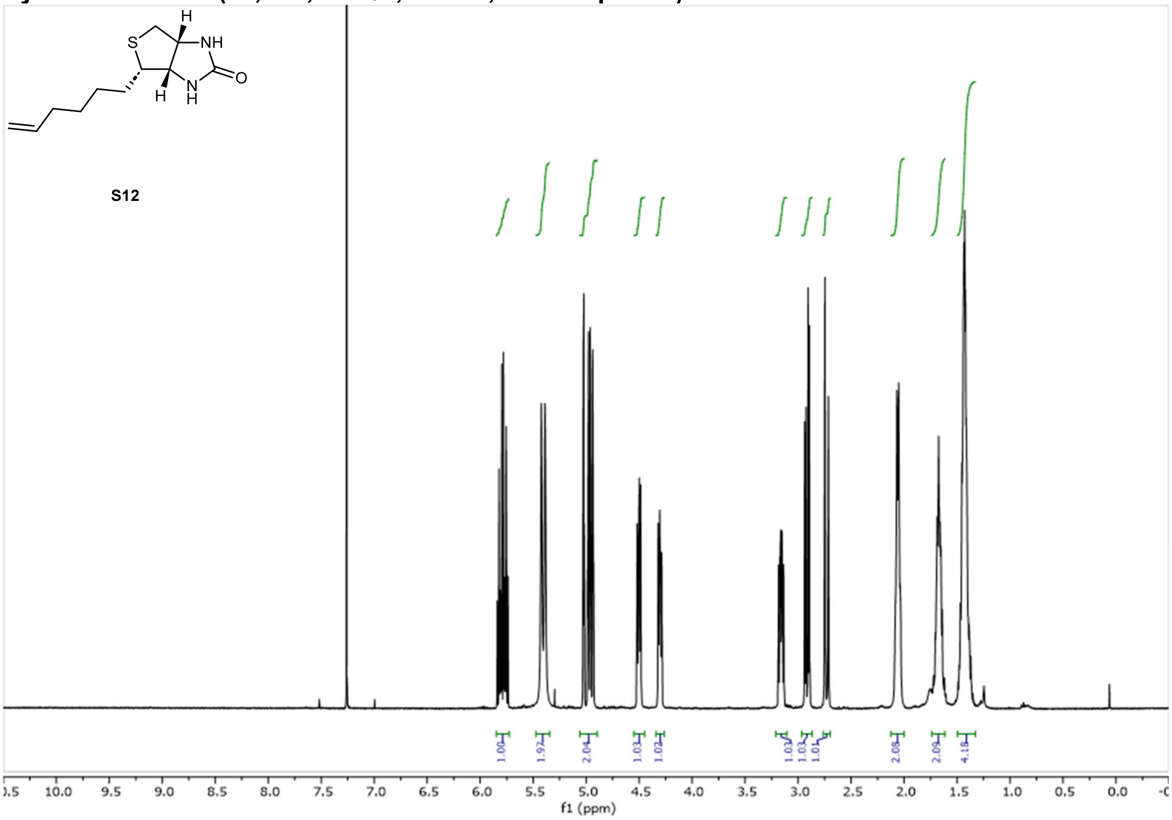

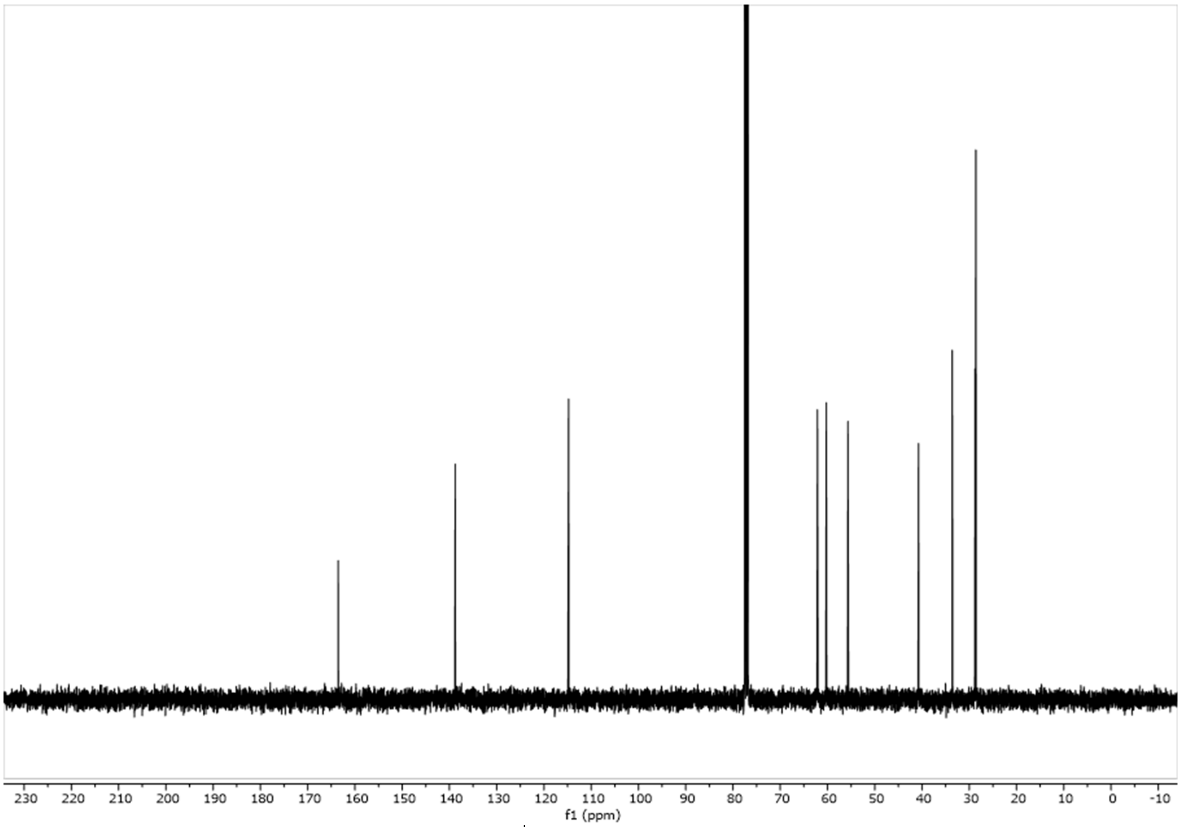
230 220 210 200 190 180 170 160 150 140 130 120 110 100 90 80 70 60 50 40 30 20 10 0 -10
f1 (ppm)

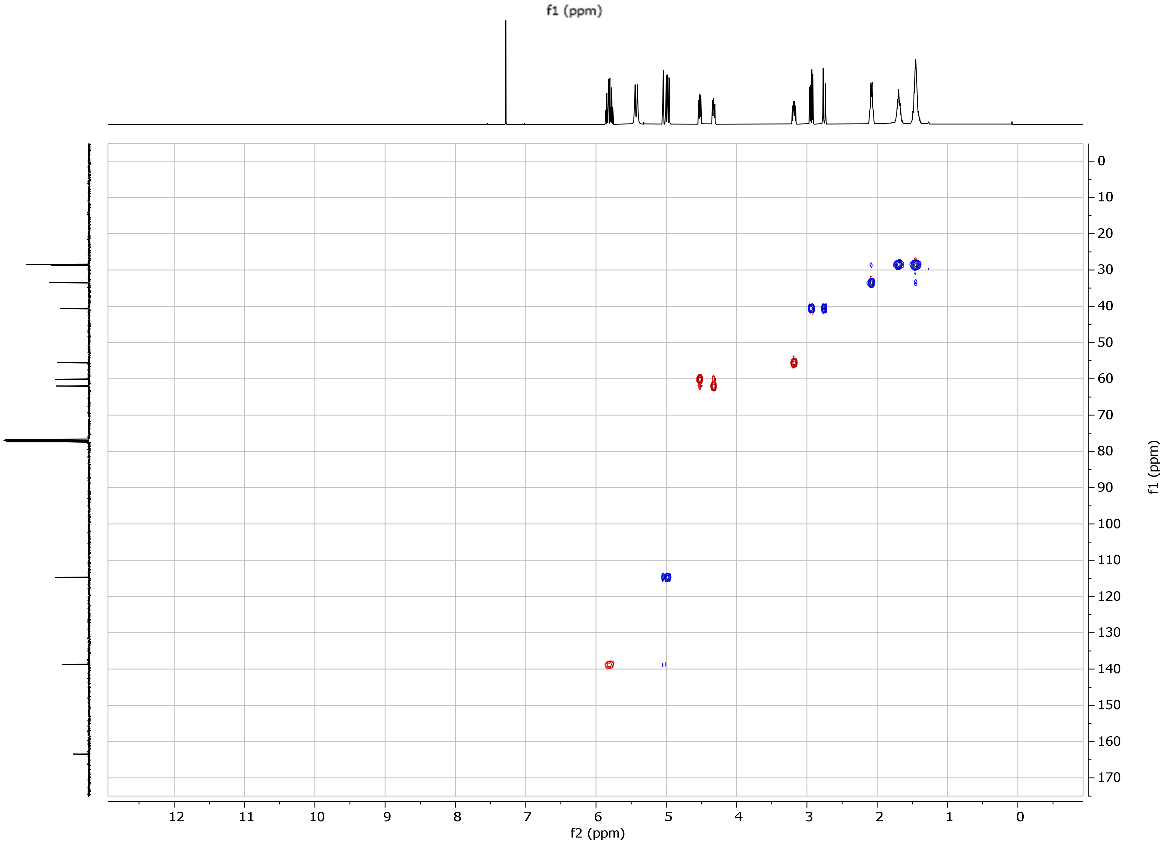
12 11 10 9 8 7 6 5 4 3 2 1 0
f2 (ppm)
0 10 20 30 40 50 60 70 80 90 100 110 120 130 140 150 160 170
f1 (ppm)

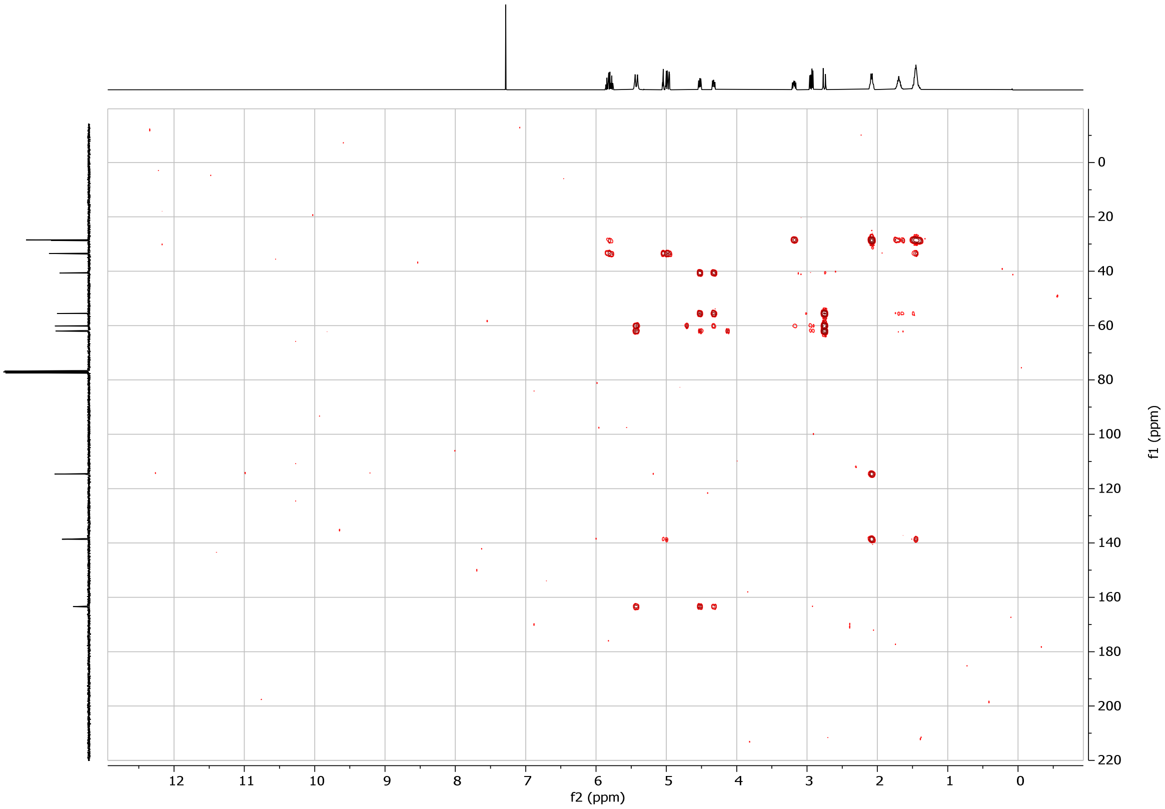
f2 (ppm)
f1 (ppm)

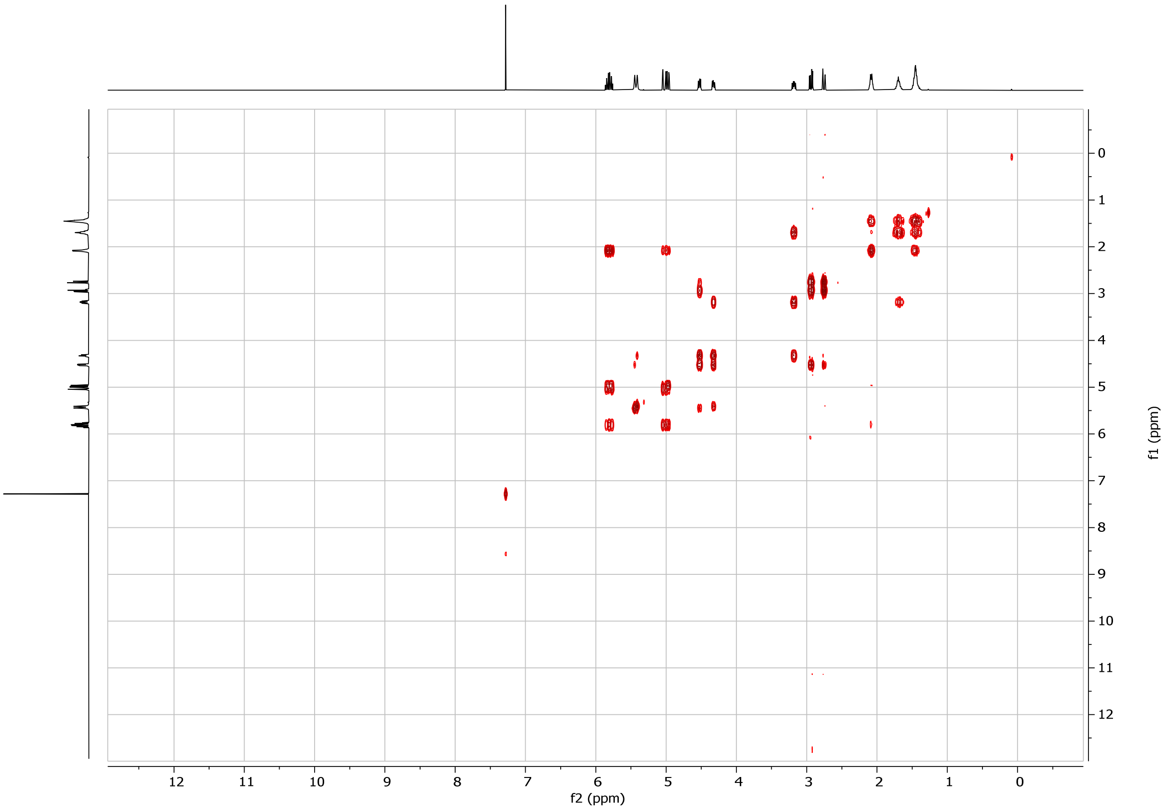
f2 (ppm)
f1 (ppm)

Synthesis of 22 (^{1}H, ^{13}C, HSQC, HMBC, COSY spectra)

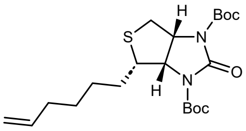

22

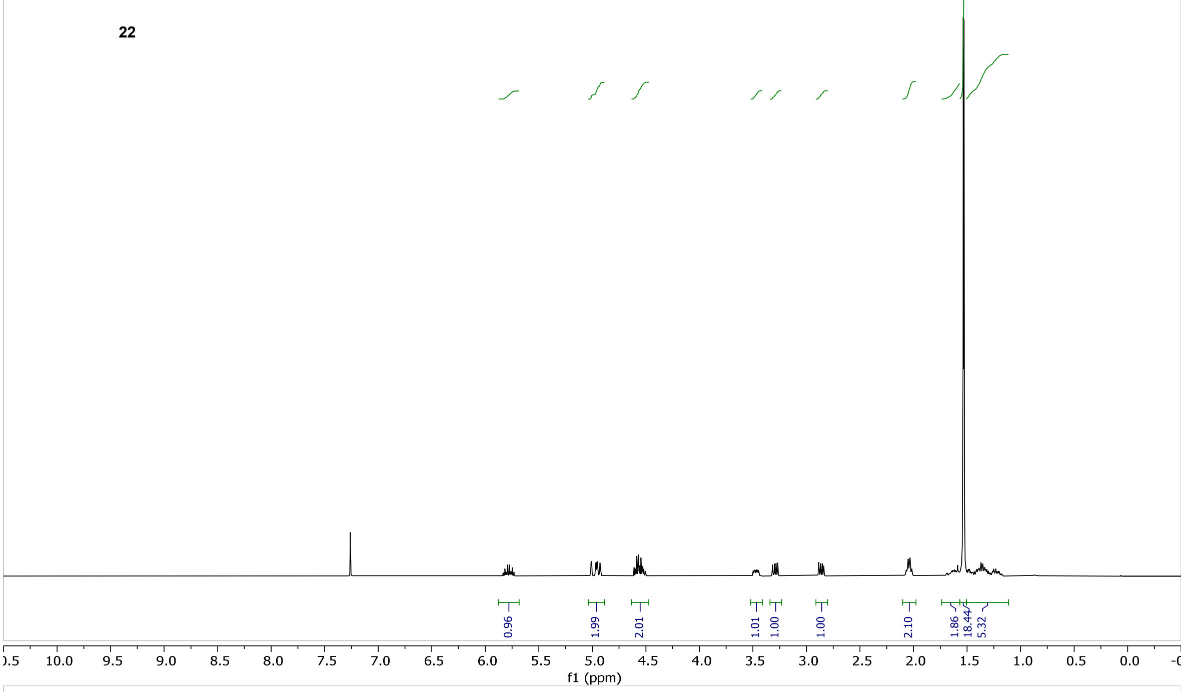

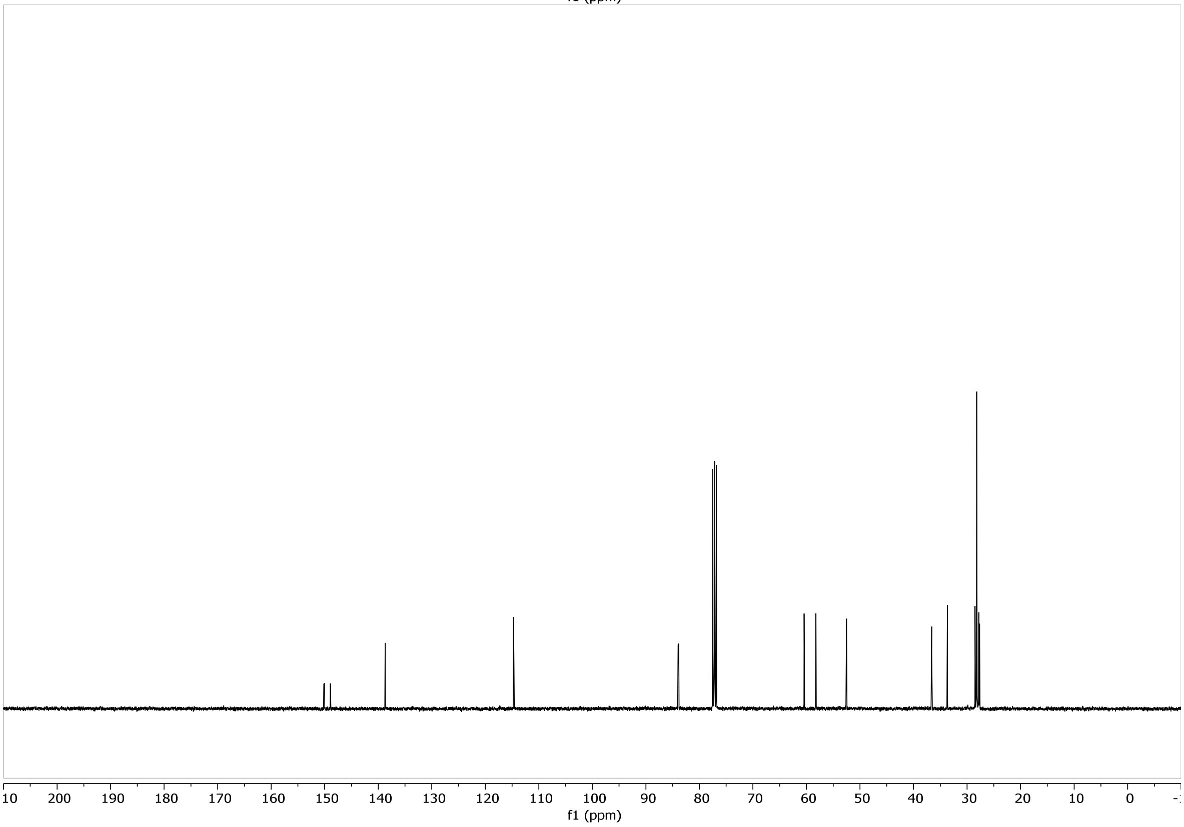

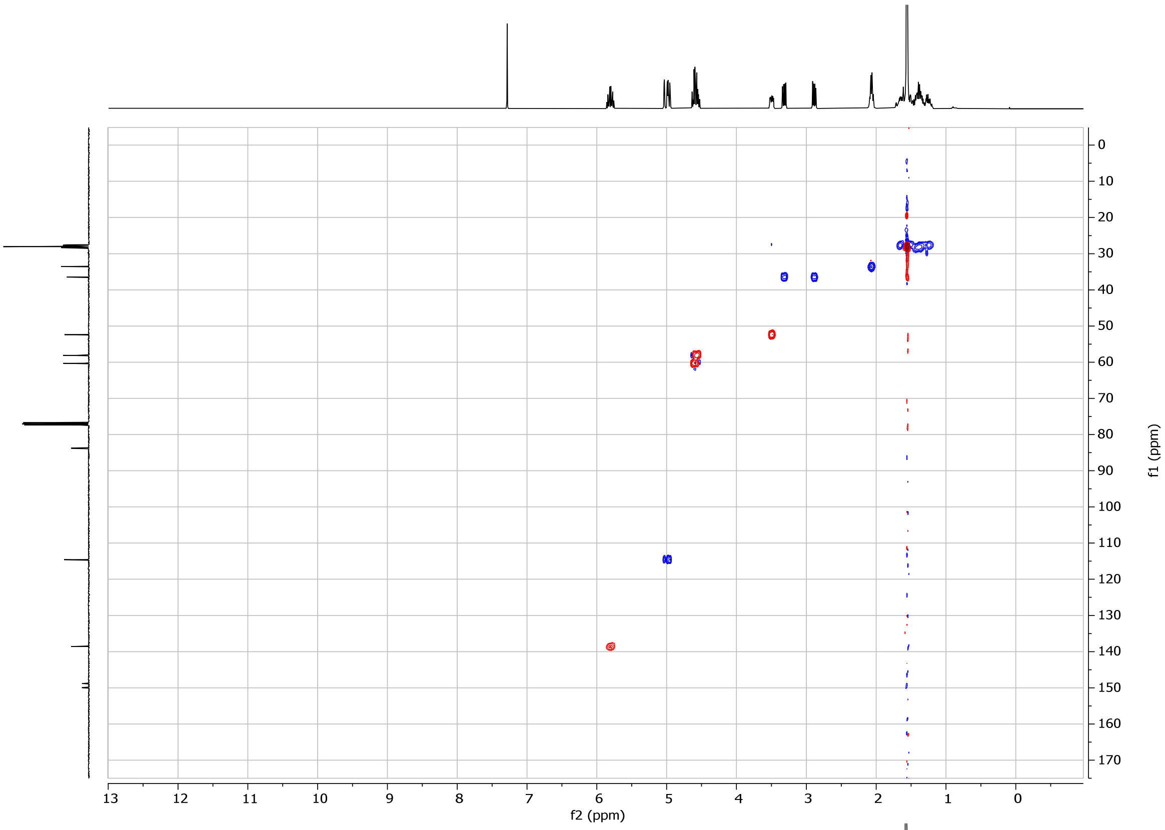

f2 (ppm)
f1 (ppm)

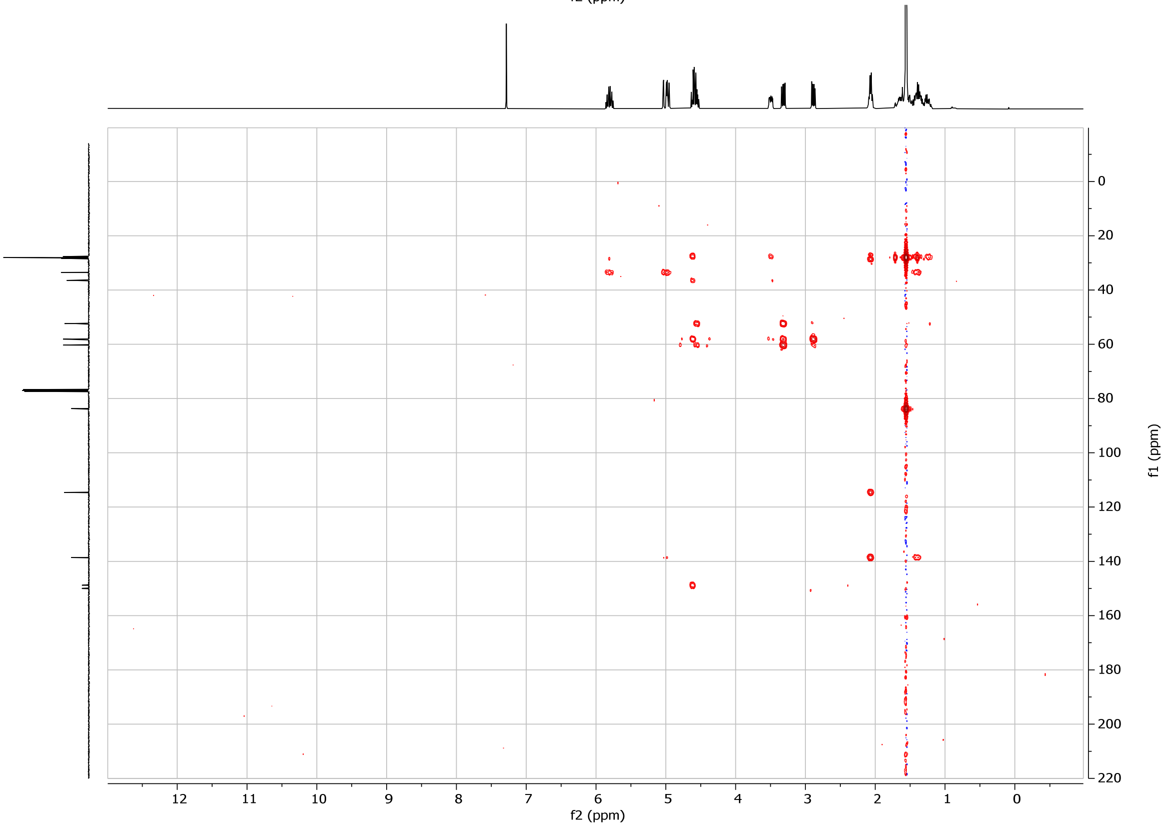

f2 (ppm)
f1 (ppm)

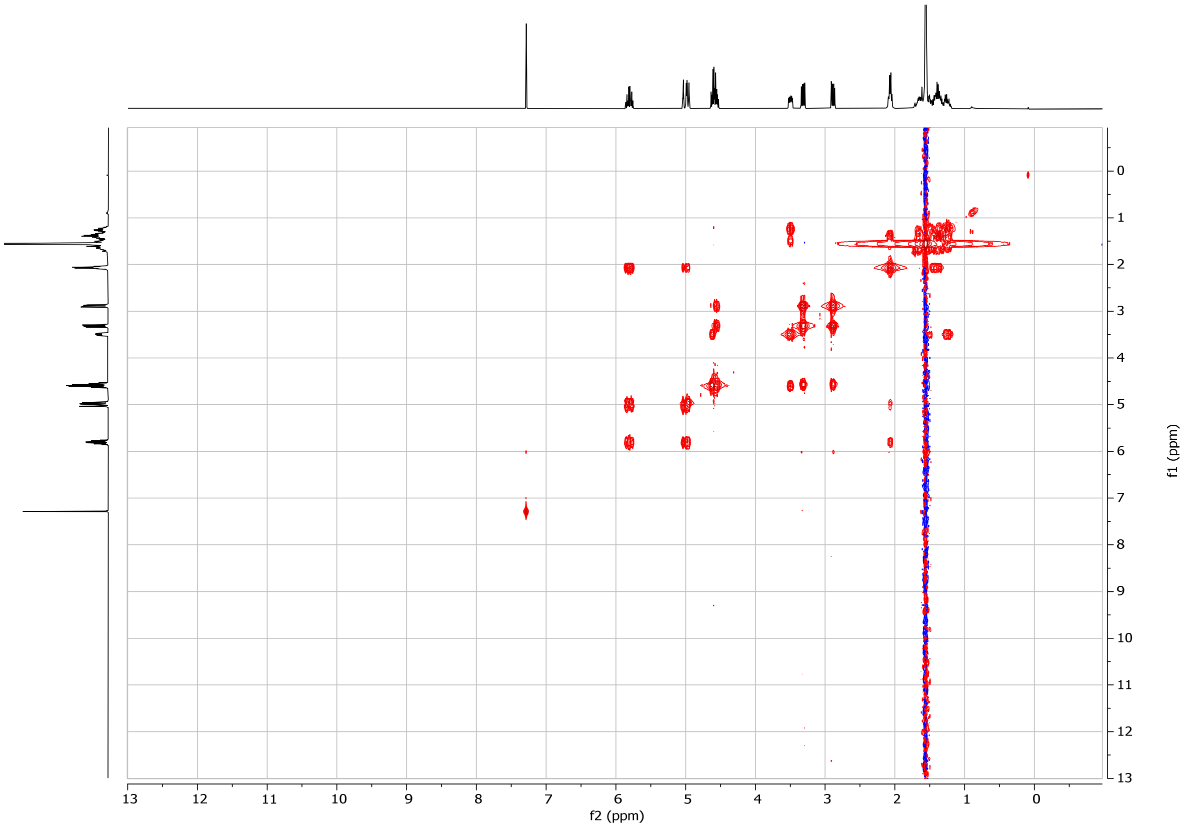
f2 (ppm)
f1 (ppm)

Synthesis of 23 (^1H, ^{13}C, HSQC, HMBC, COSY spectra)

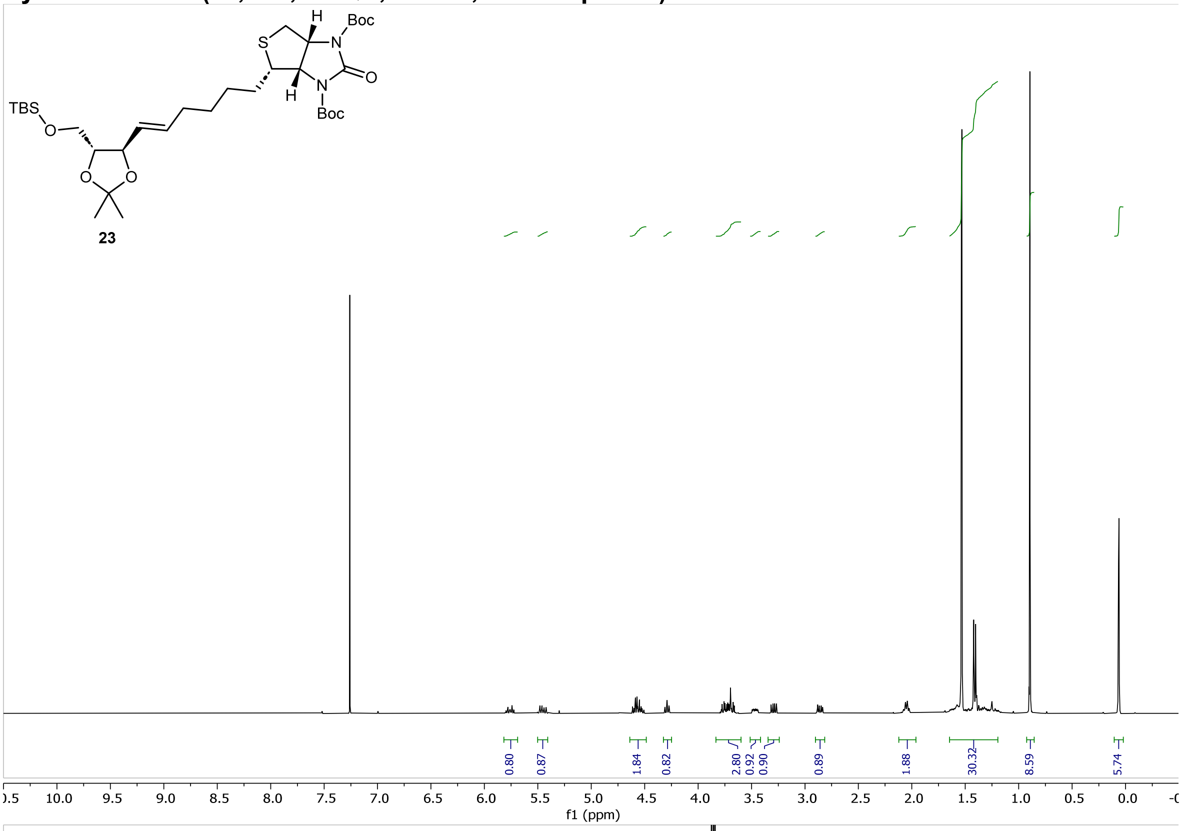

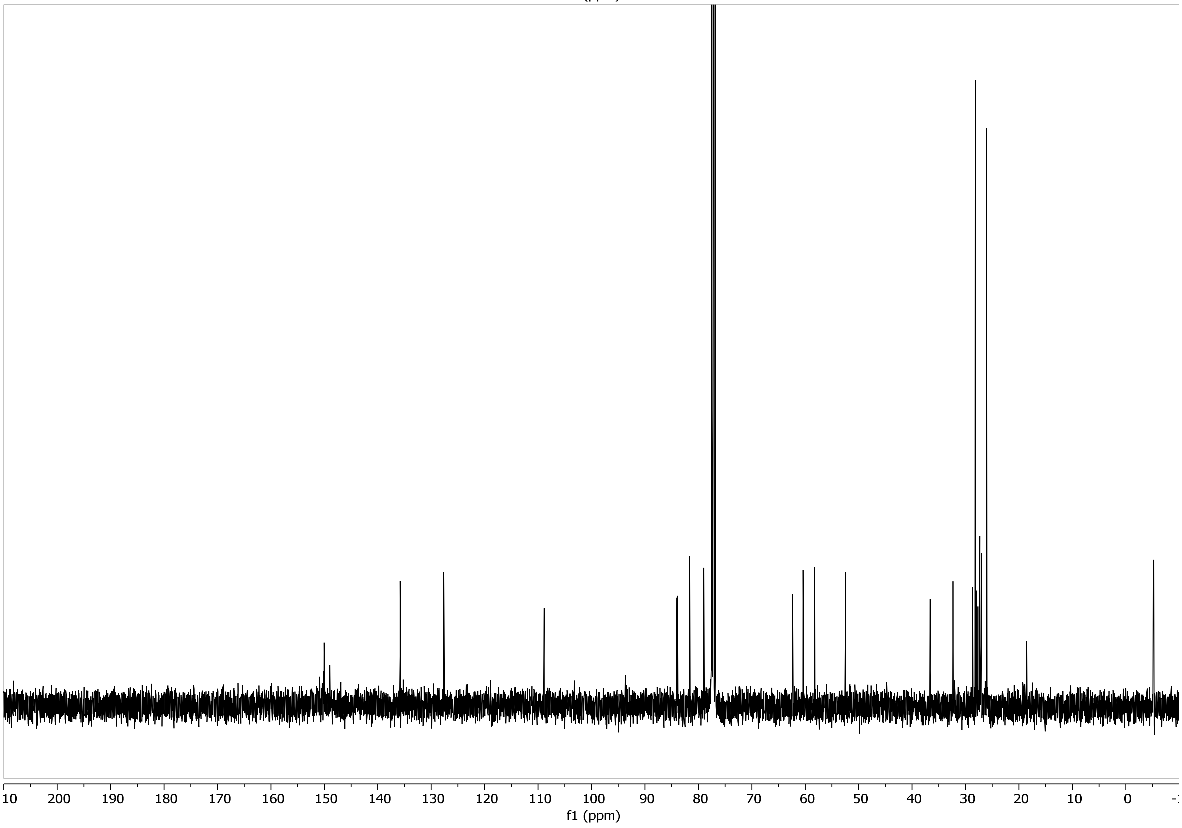

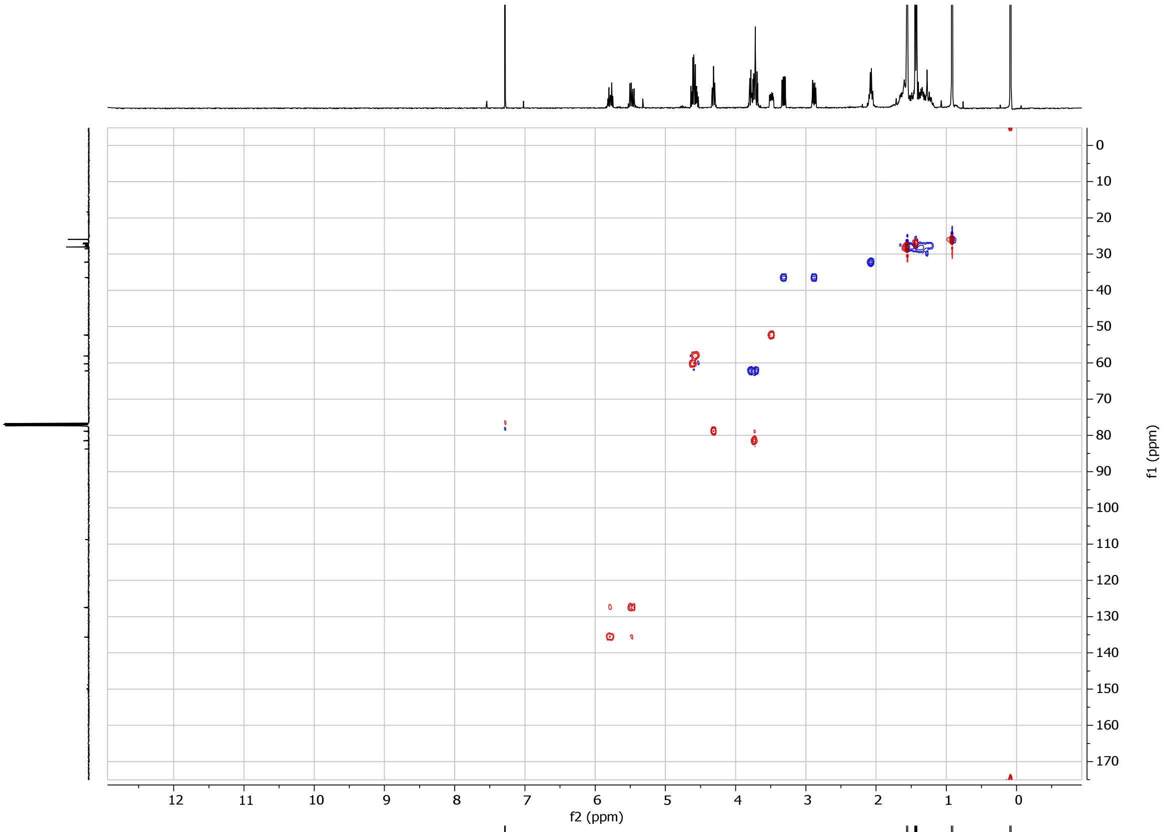

f2 (ppm)
f1 (ppm)

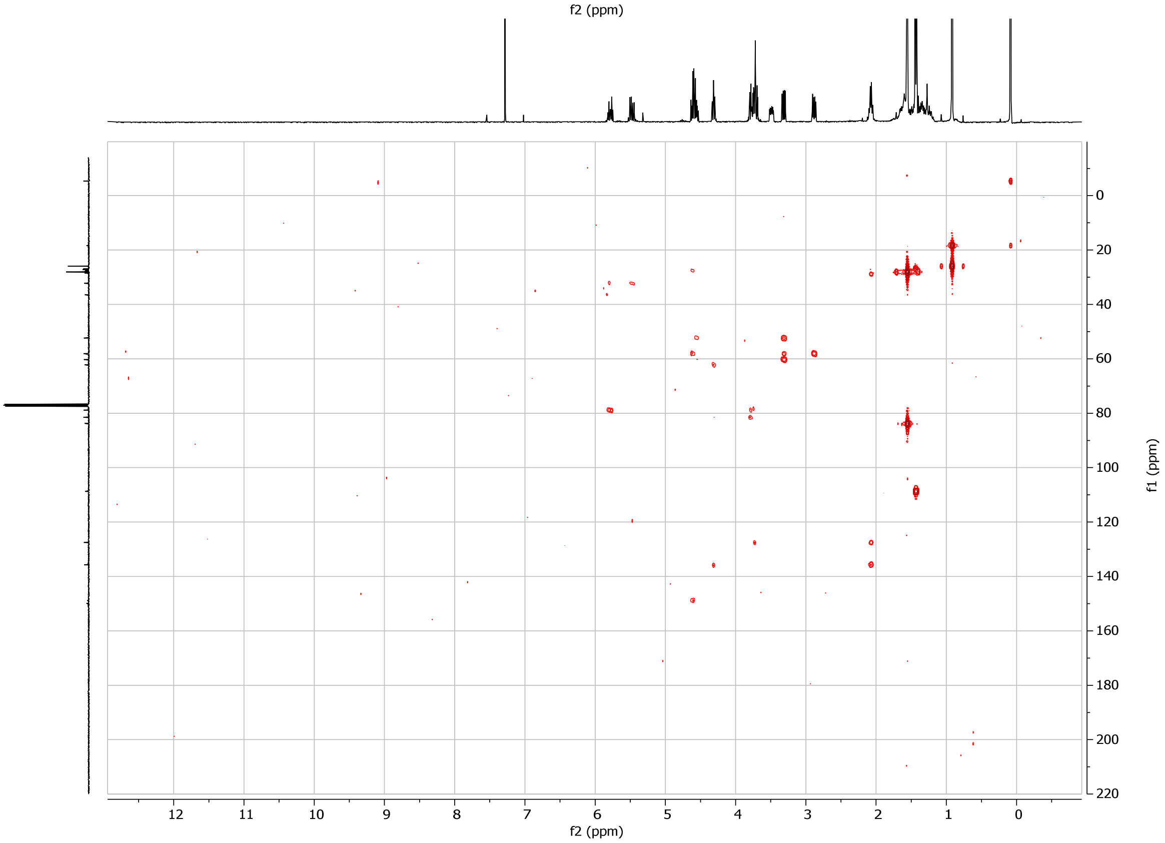

f2 (ppm)
f1 (ppm)

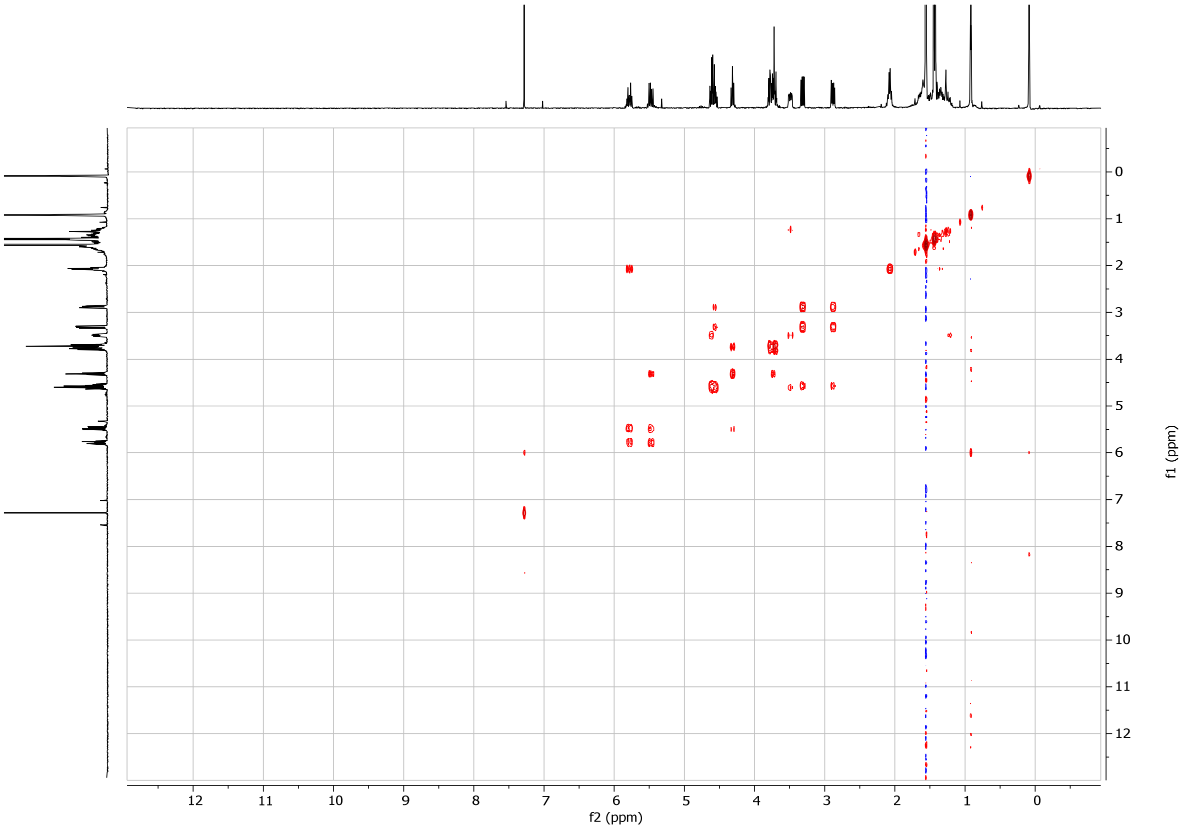
f2 (ppm)
f1 (ppm)

Synthesis of 24 (^{1}H, ^{13}C, HSQC, HMBC, COSY spectra)

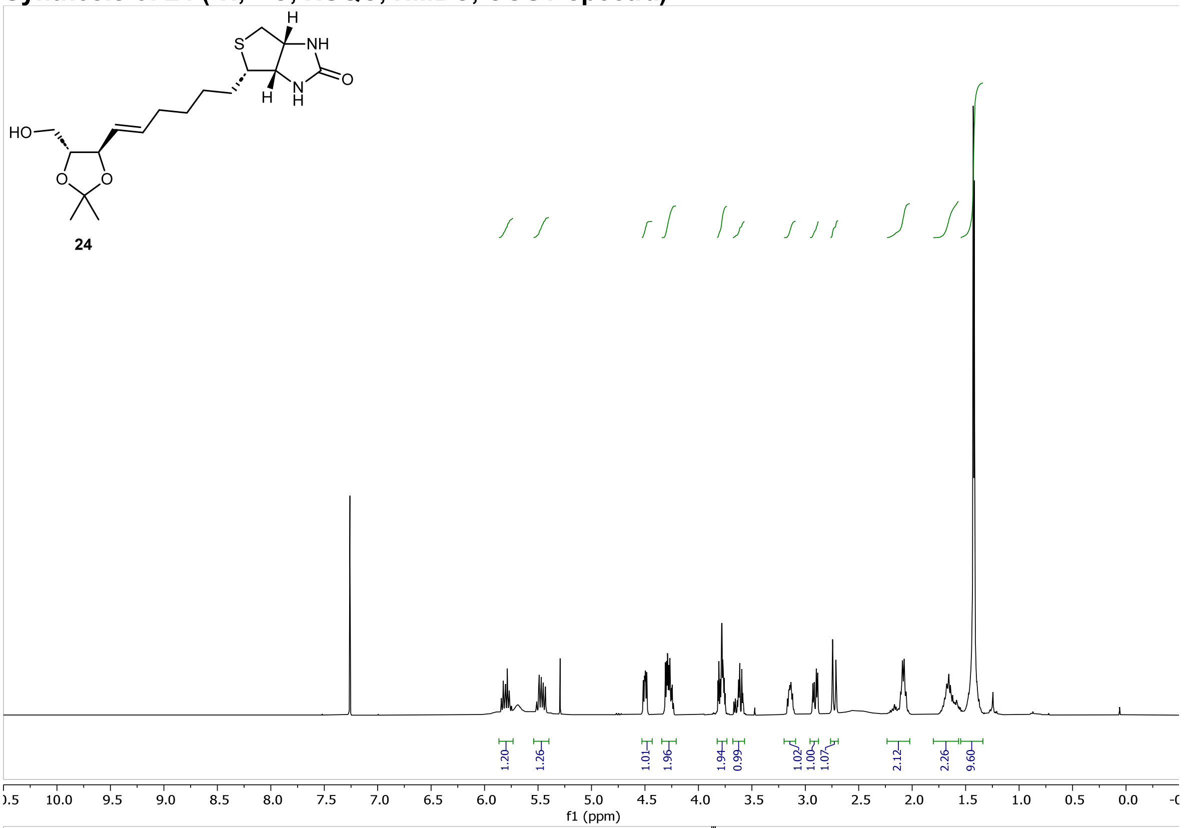

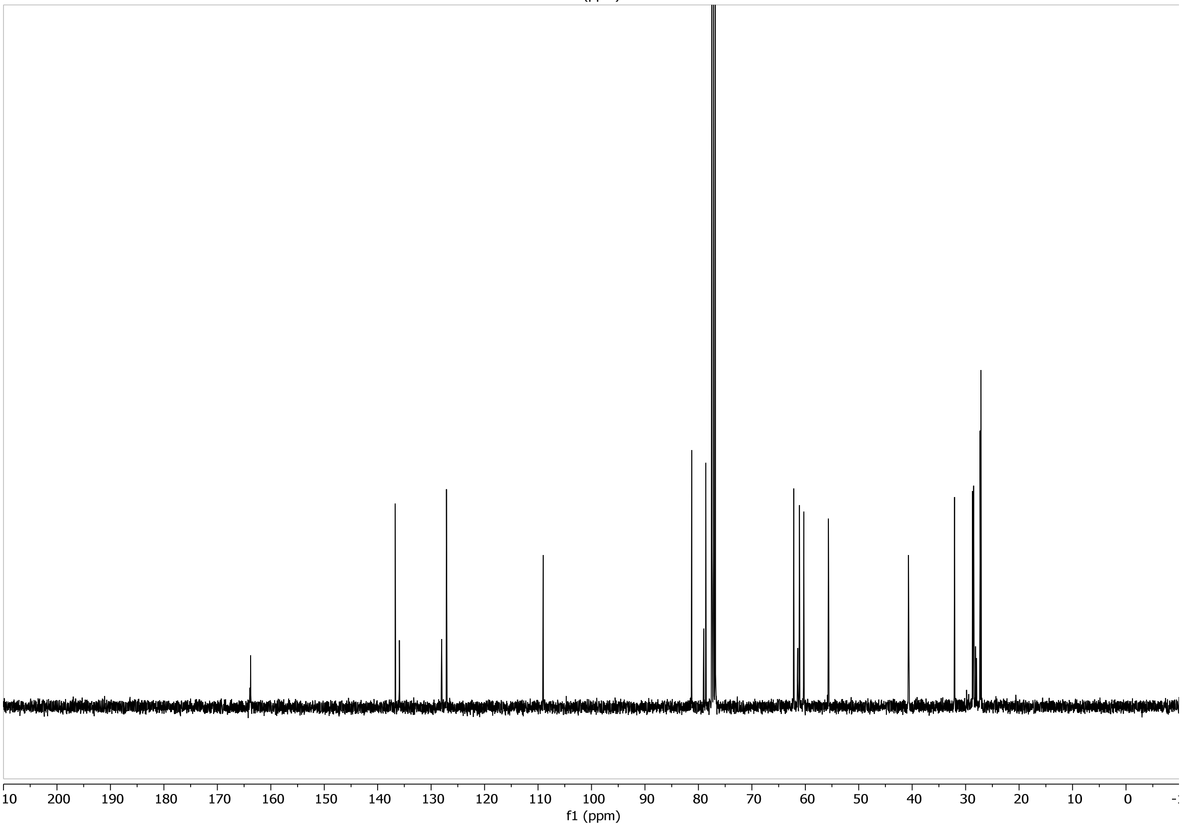

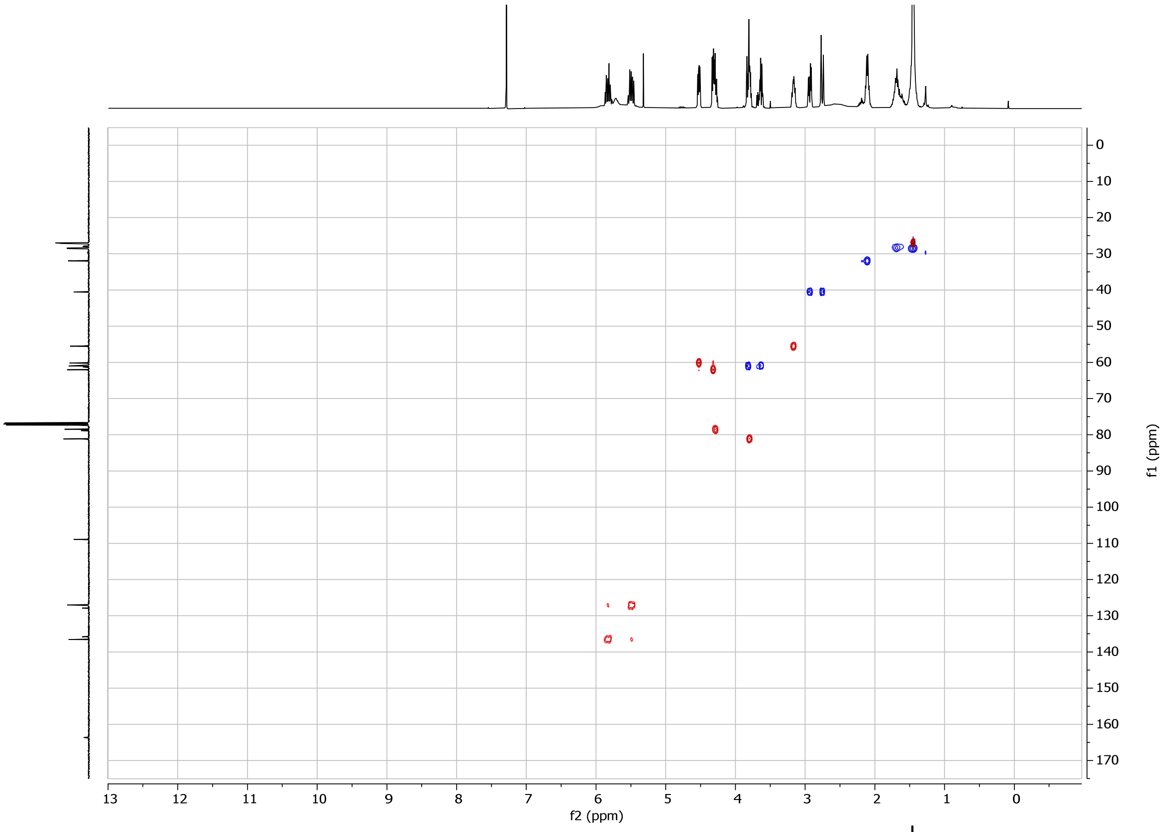
f2 (ppm)
f1 (ppm)

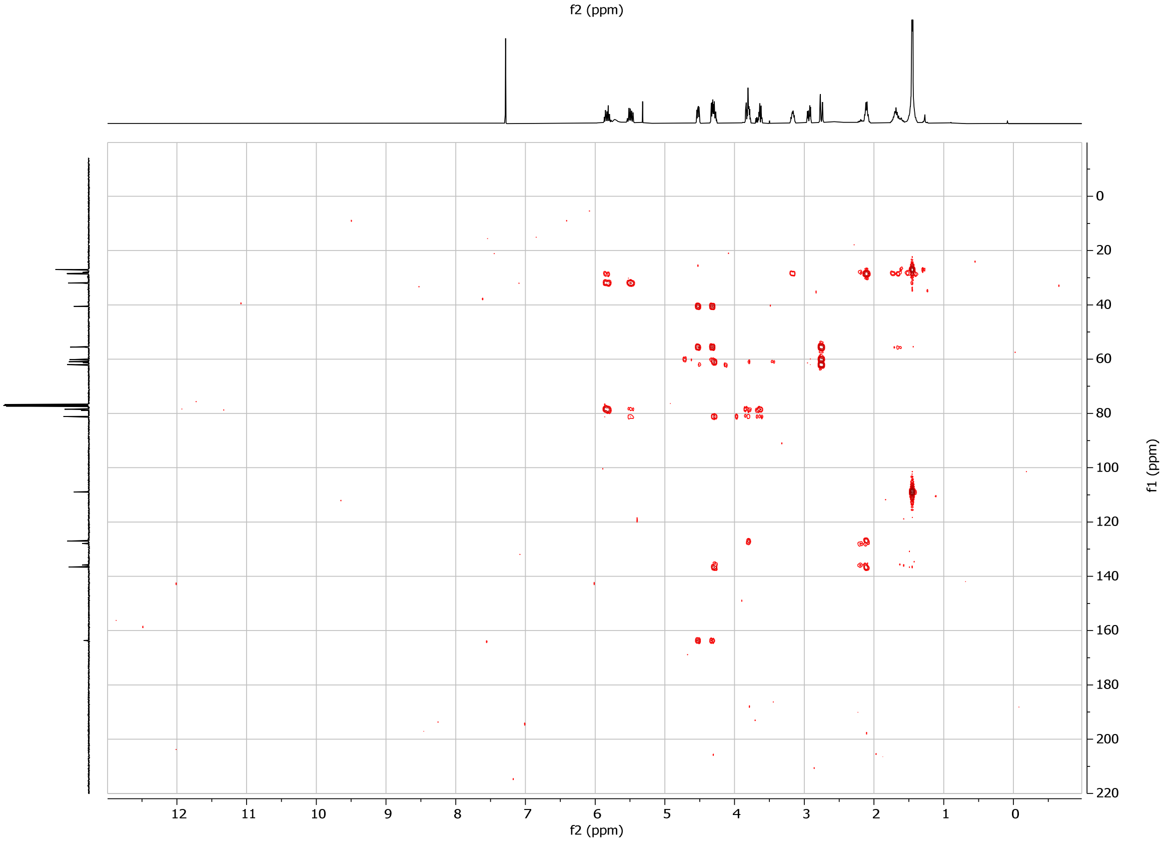
f2 (ppm)
f1 (ppm)

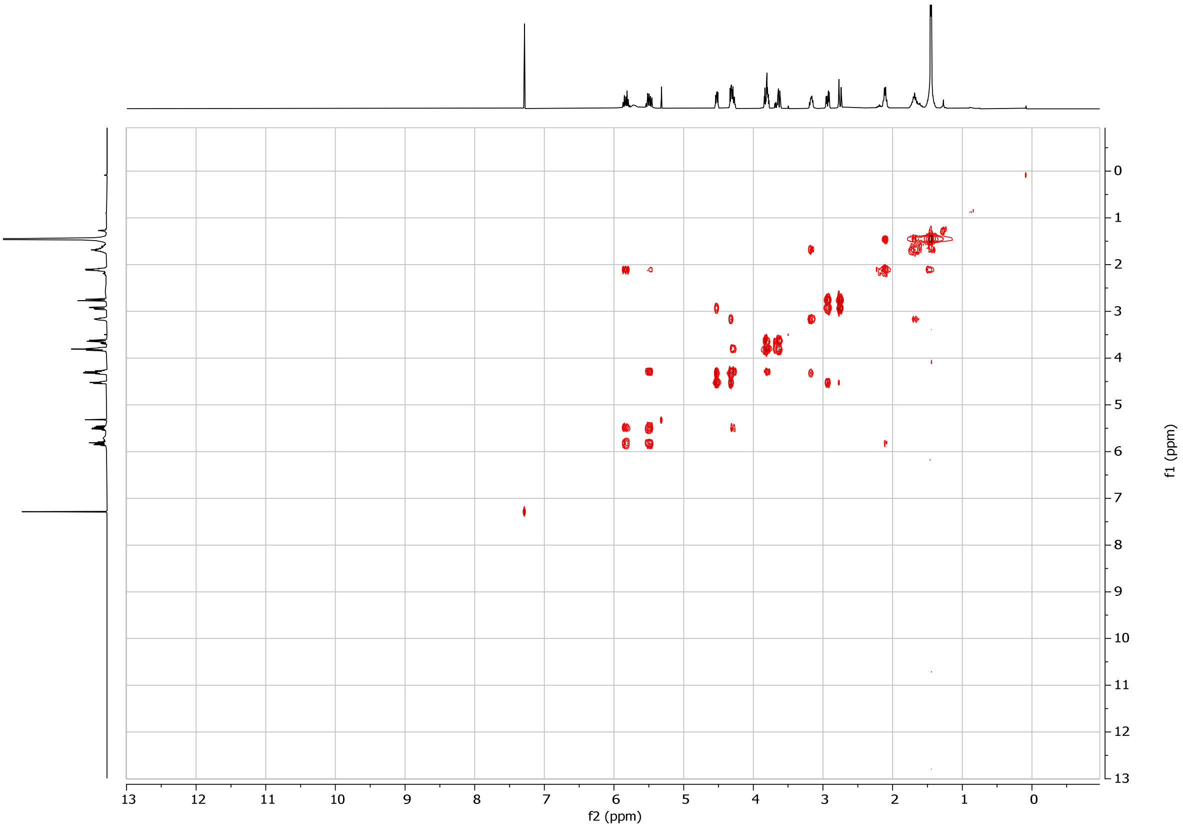
f2 (ppm)
f1 (ppm)

Synthesis of 25 (^{1}H, ^{13}C, HSQC, HMBC, COSY spectra)

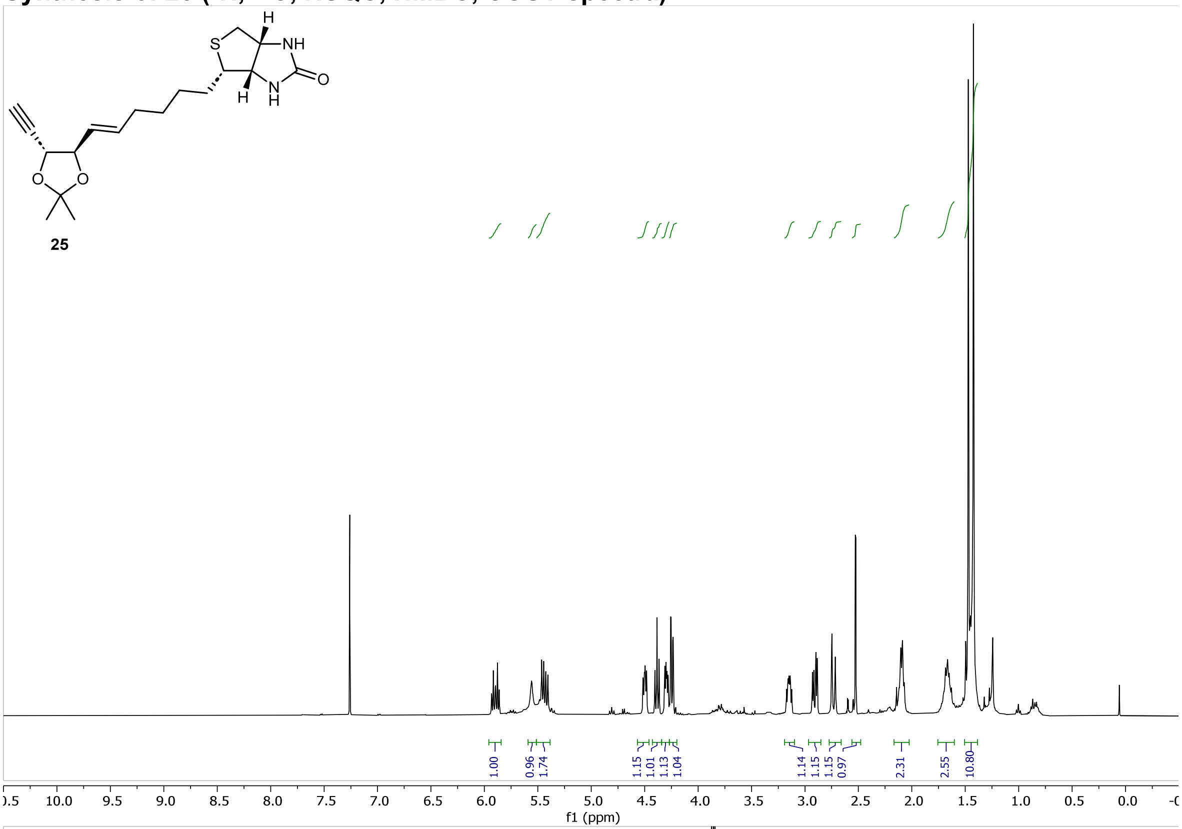

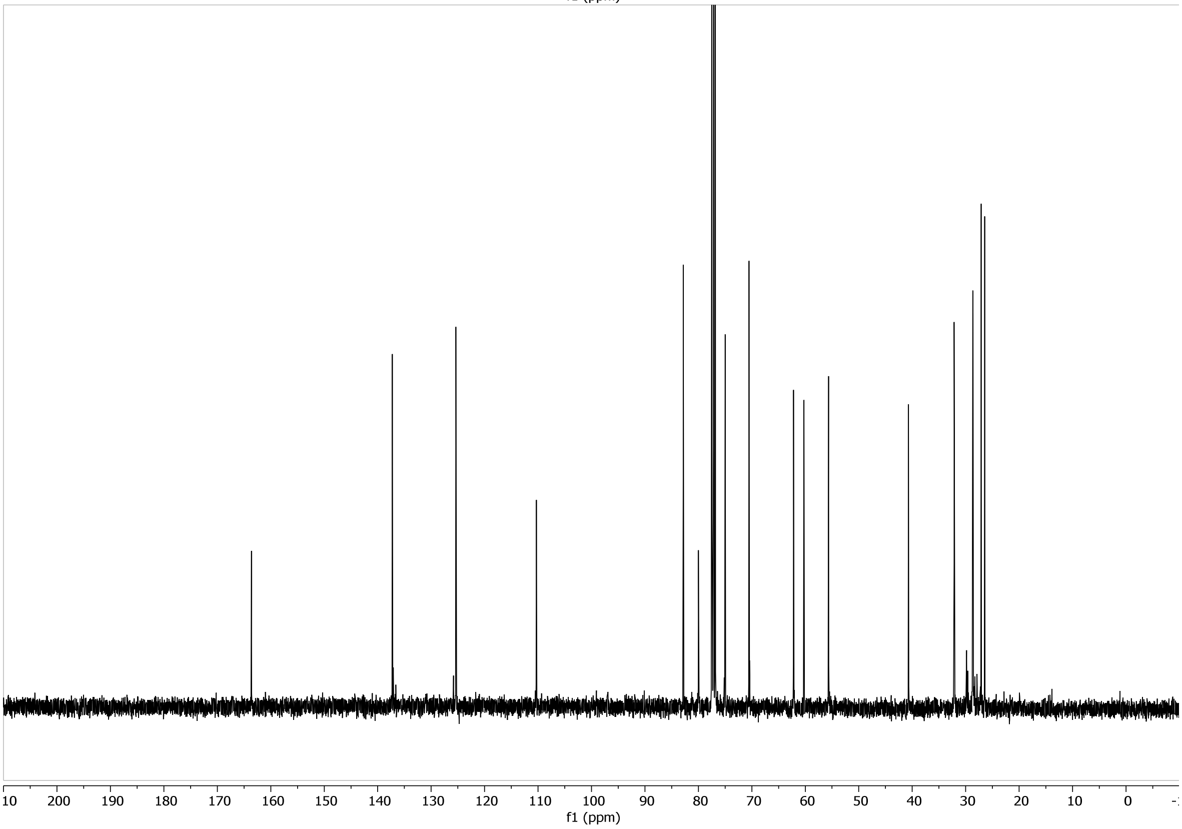

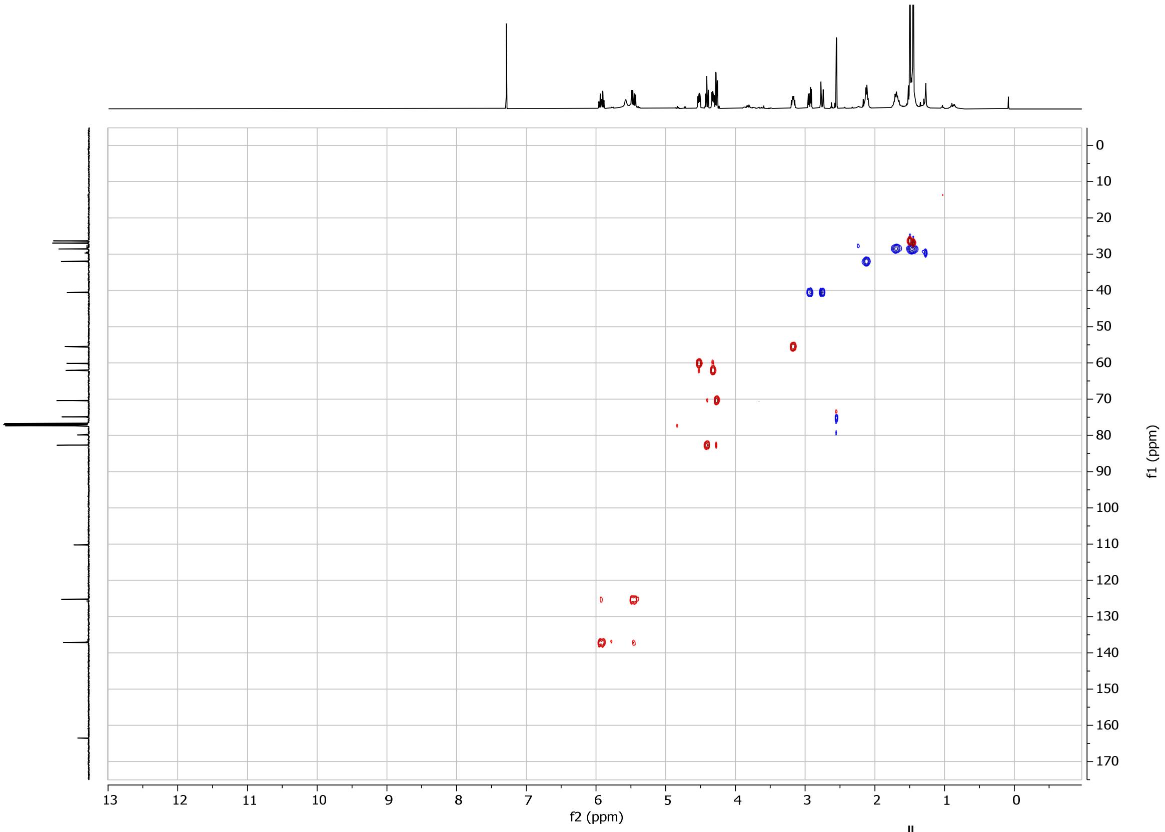
f2 (ppm)
f1 (ppm)

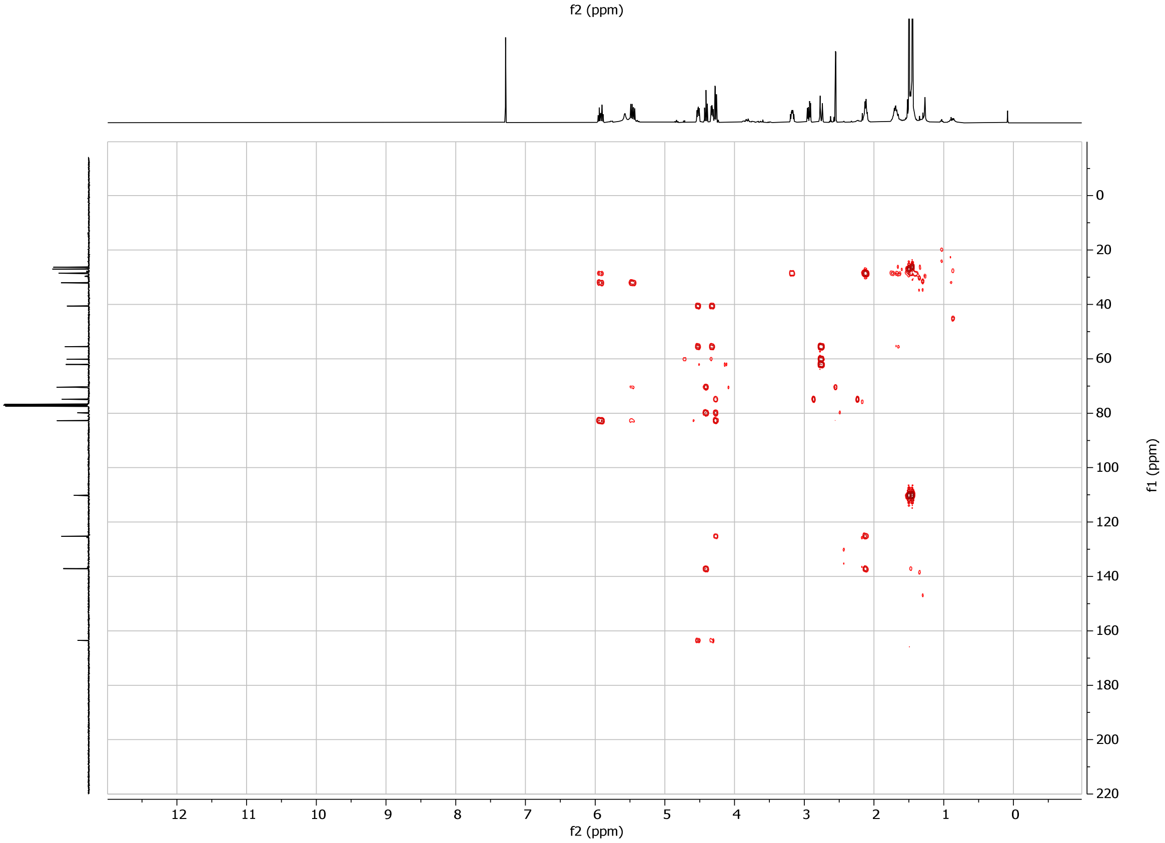
f2 (ppm)
f1 (ppm)

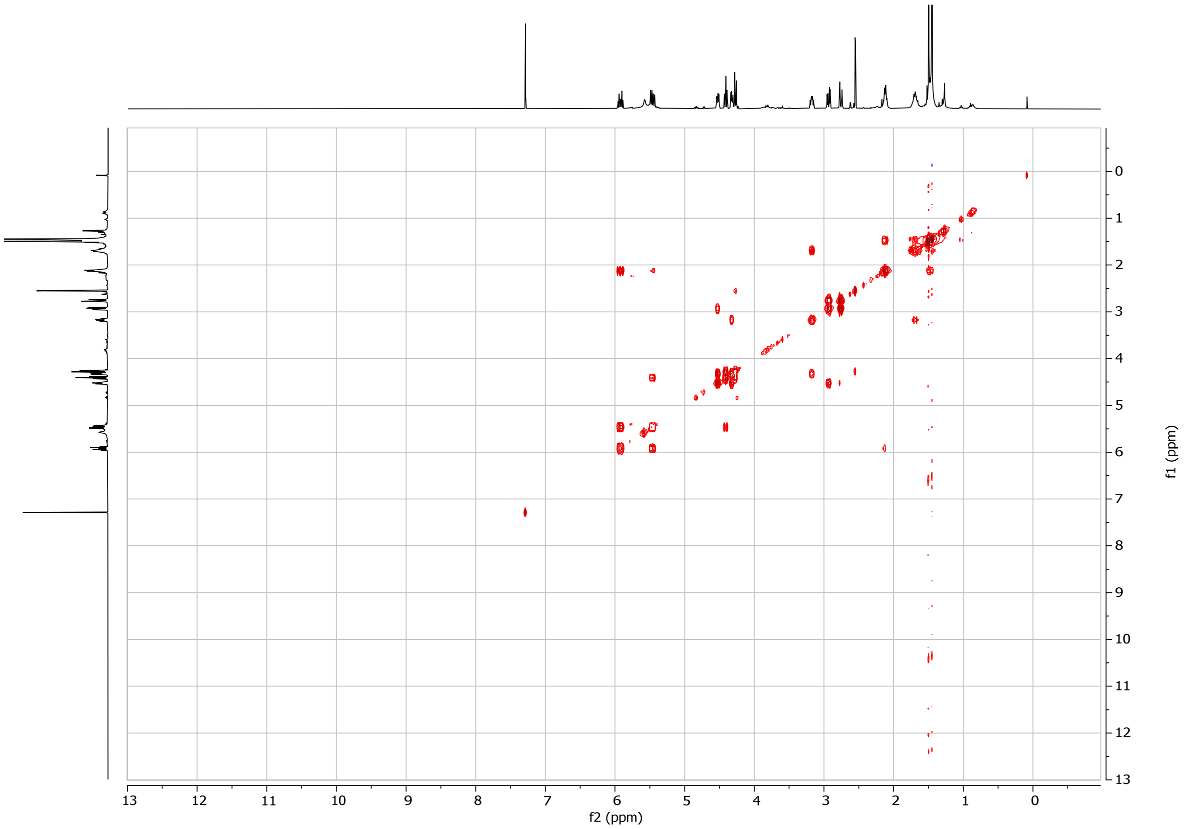

f2 (ppm)
f1 (ppm)

Synthesis of 26 (^{1}H, ^{13}C, HSQC, HMBC, COSY spectra)

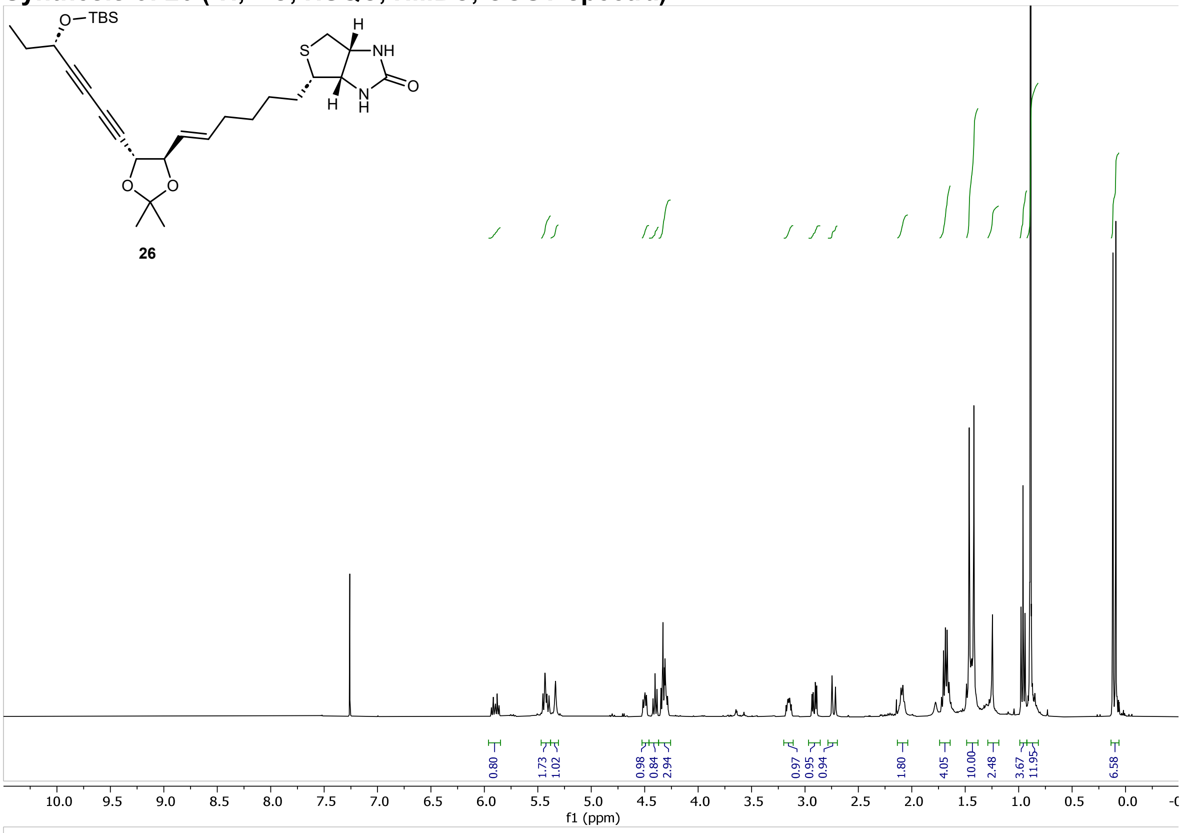

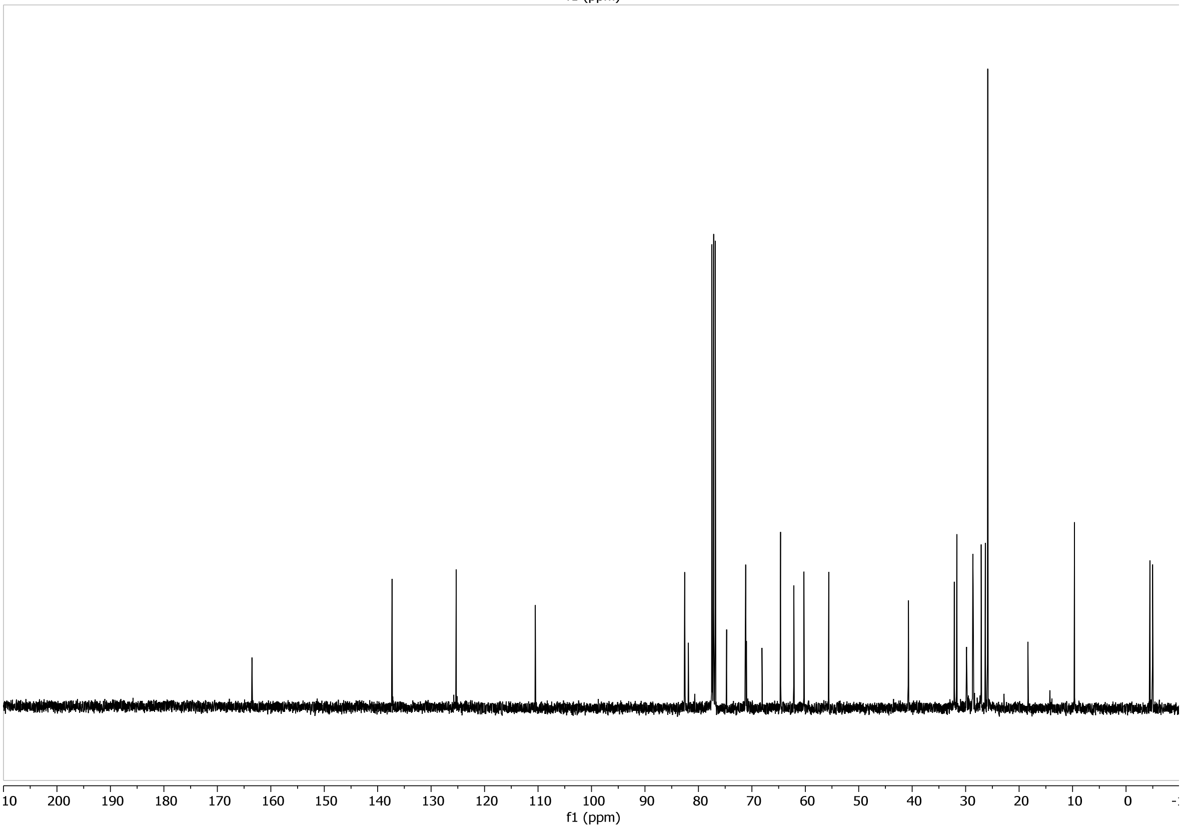

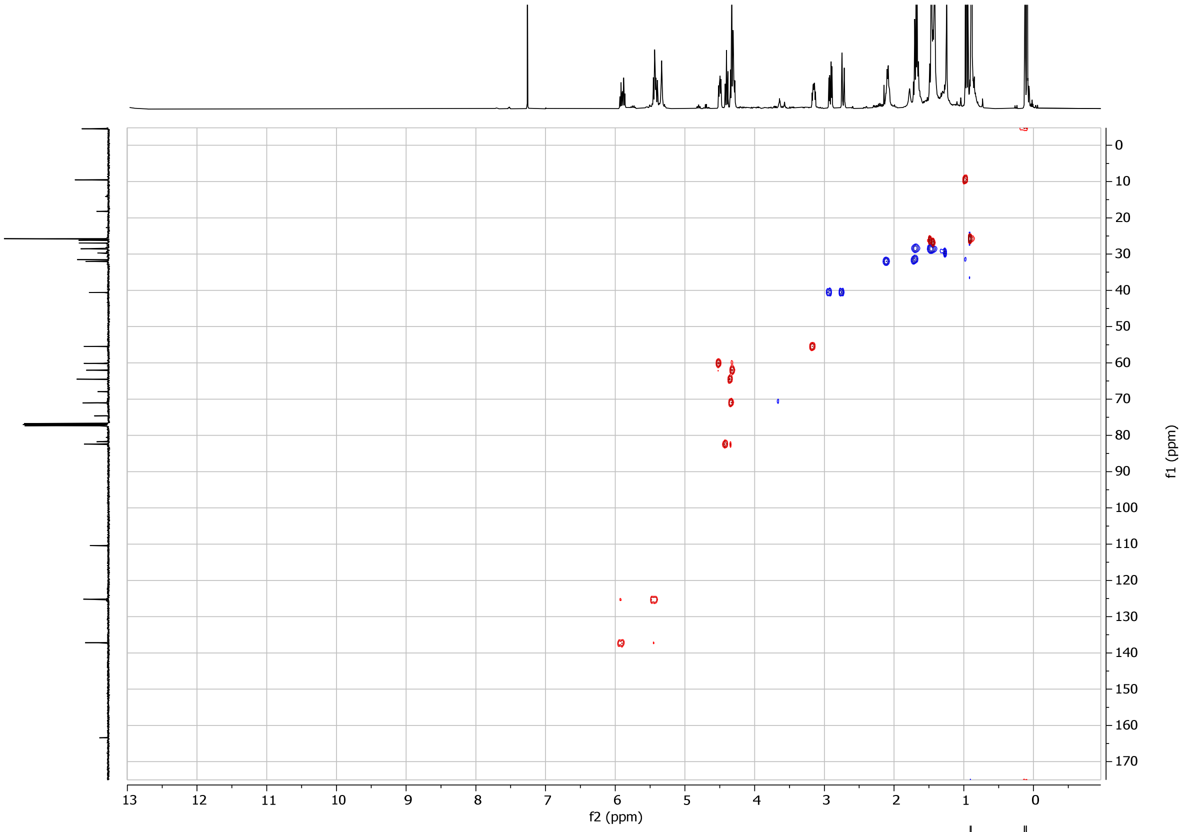

13
12
11
10
9
8
7
6
5
4
3
2
1
0
f2 (ppm)
f1 (ppm)
0
10
20
30
40
50
60
70
80
90
100
110
120
130
140
150
160
170

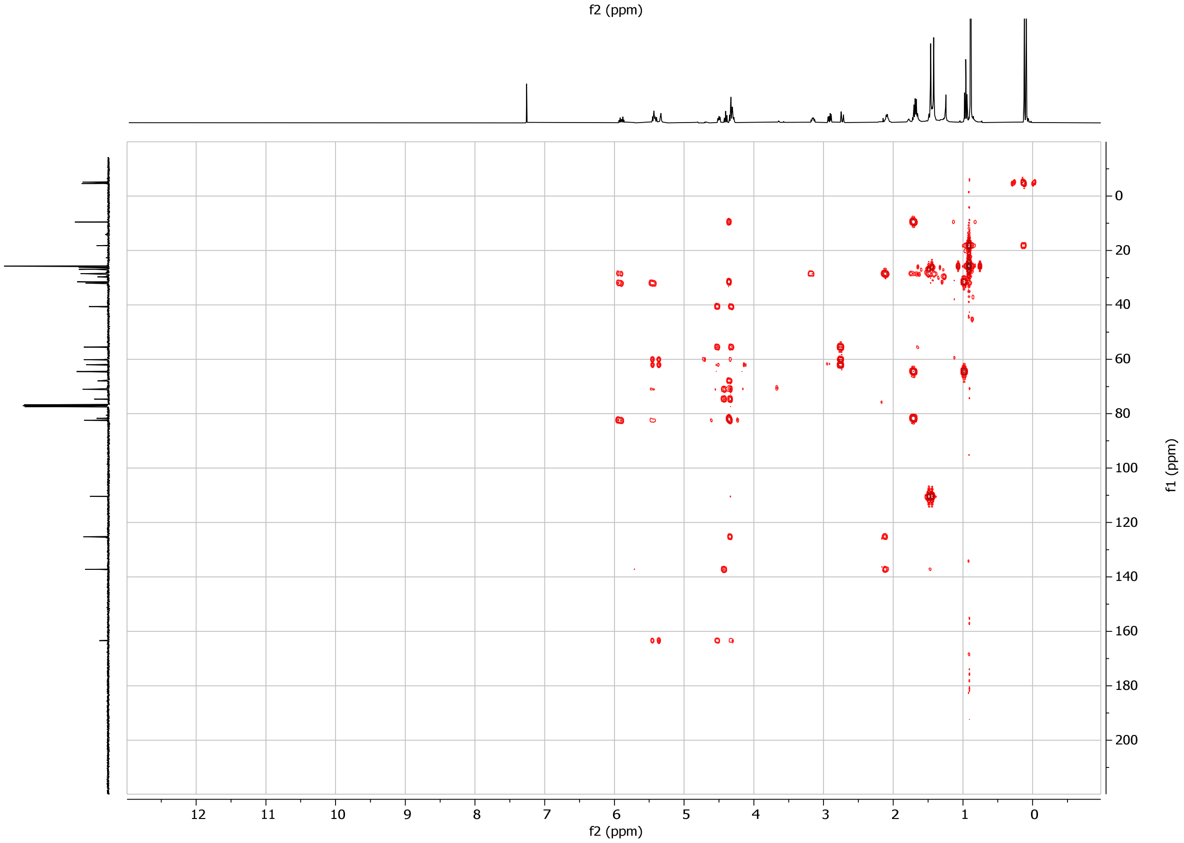

12
11
10
9
8
7
6
5
4
3
2
1
0
f2 (ppm)
f1 (ppm)
0
20
40
60
80
100
120
140
160
180
200

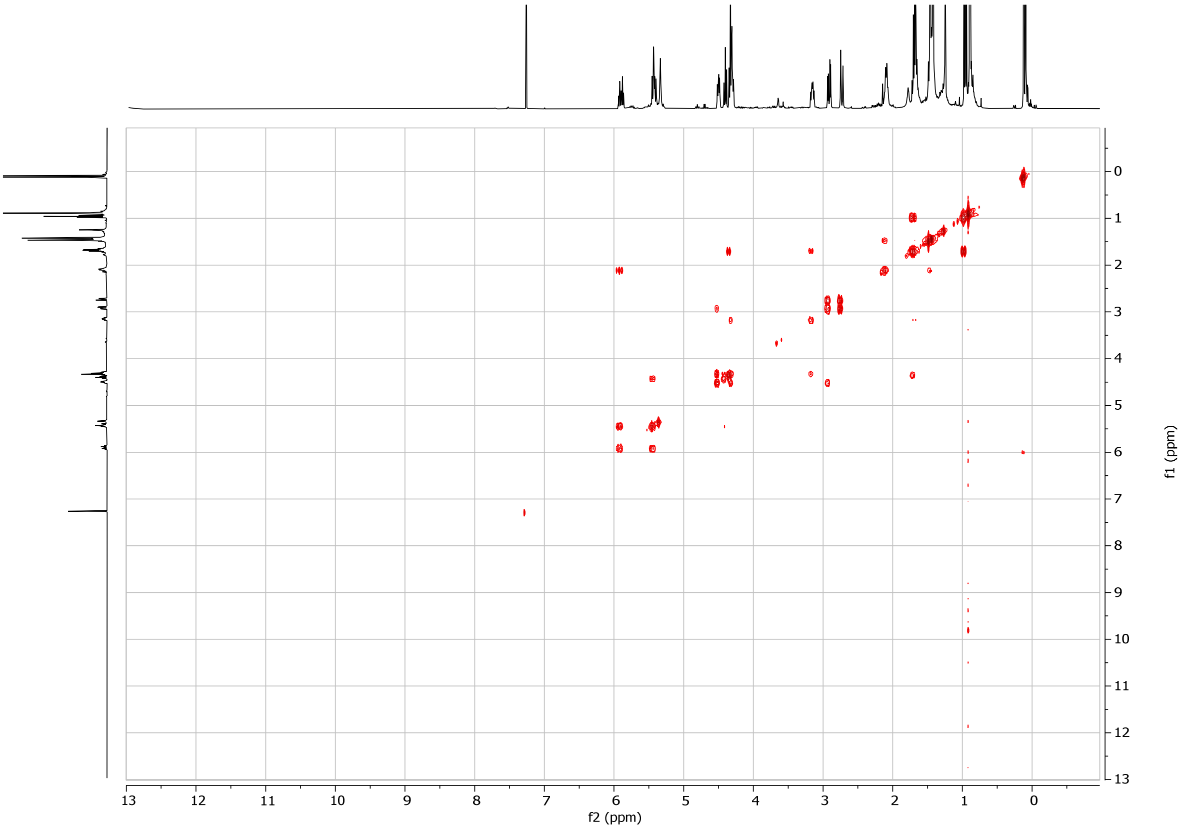

f2 (ppm)
f1 (ppm)

Synthesis of 15 (^{1}H, ^{13}C, HSQC, HMBC, COSY spectra)

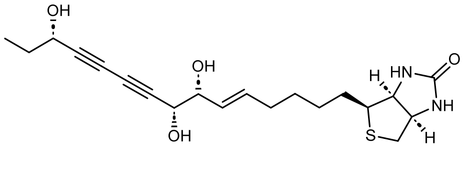

15

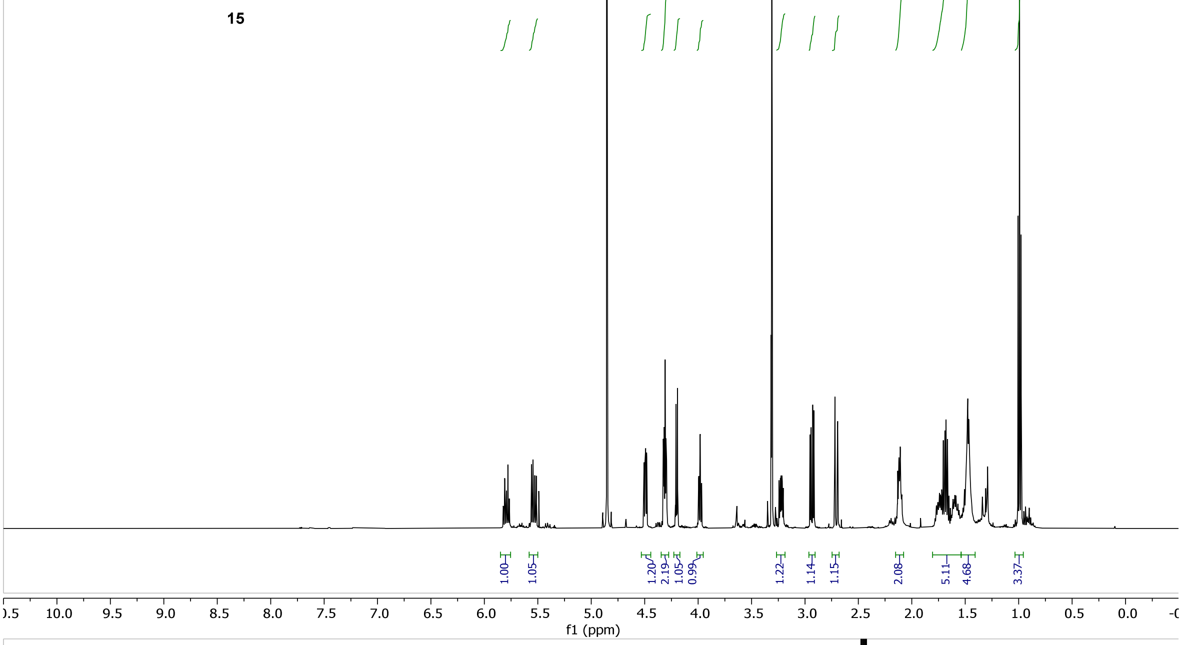

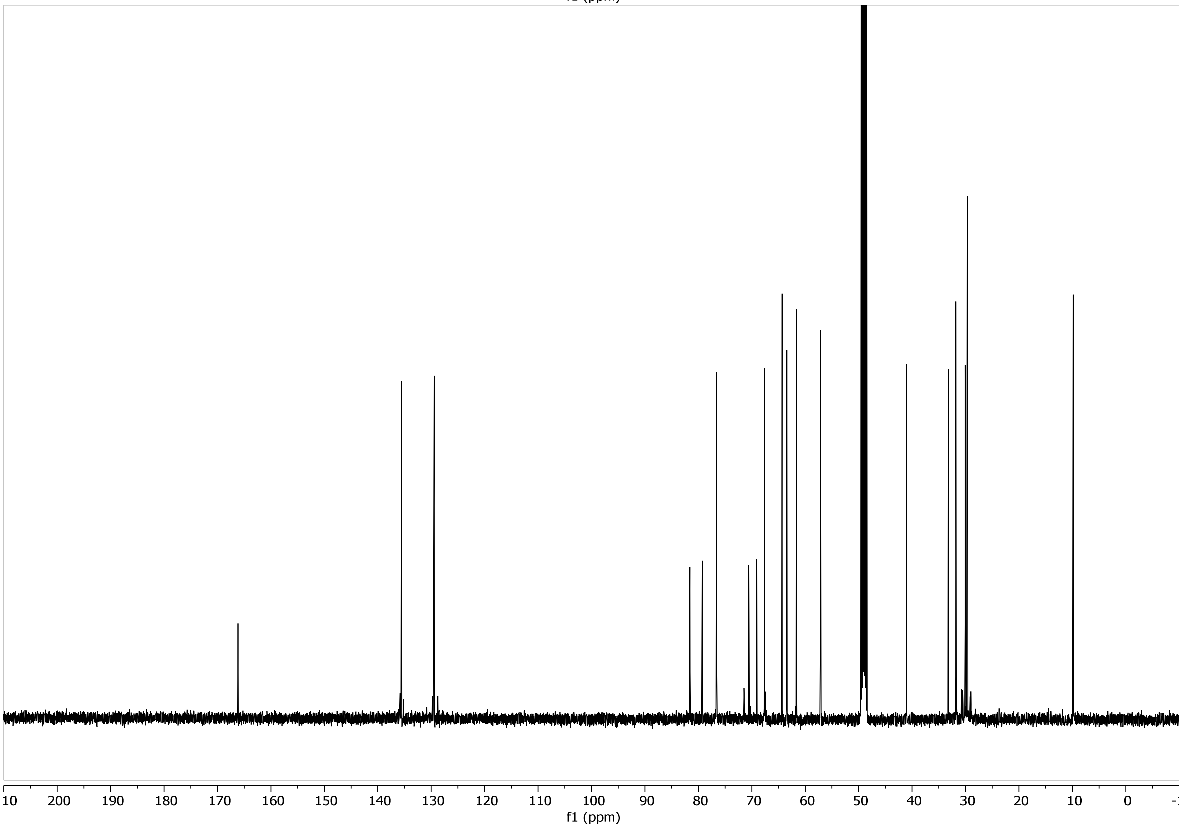

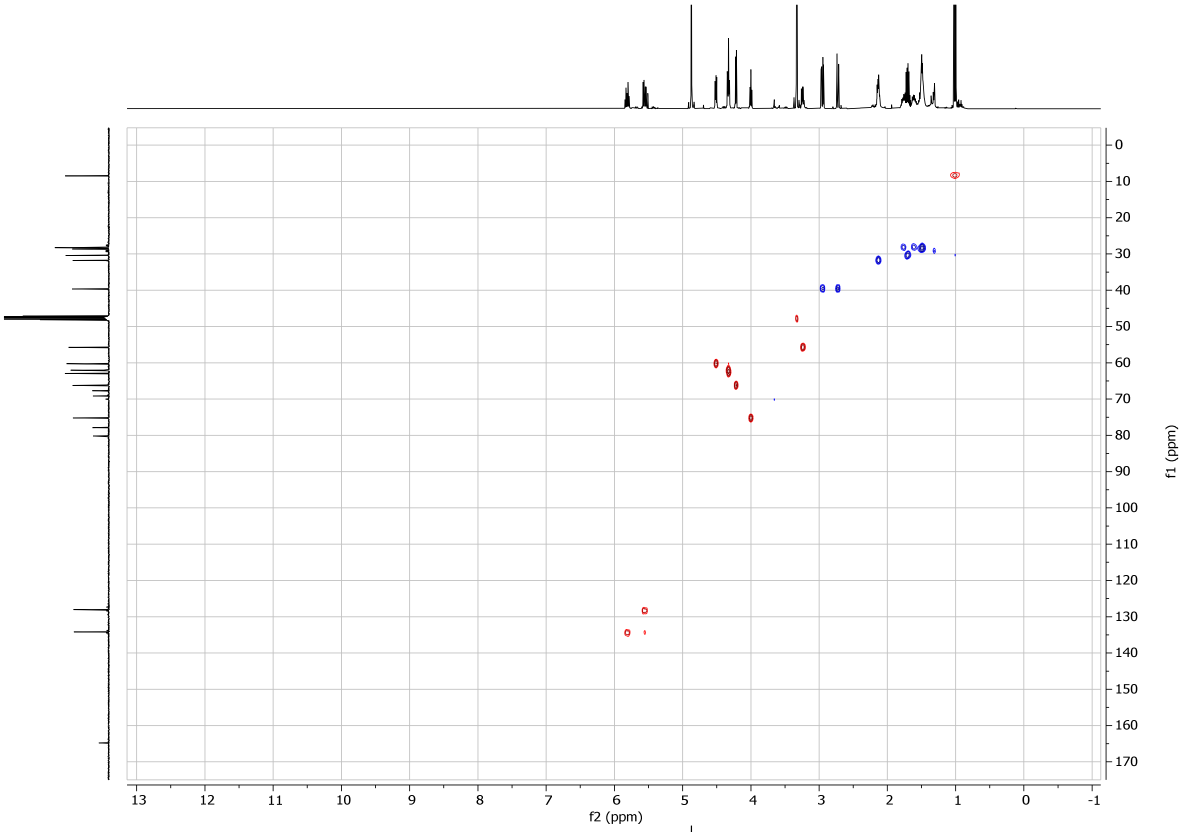

f1 (ppm)
f2 (ppm)

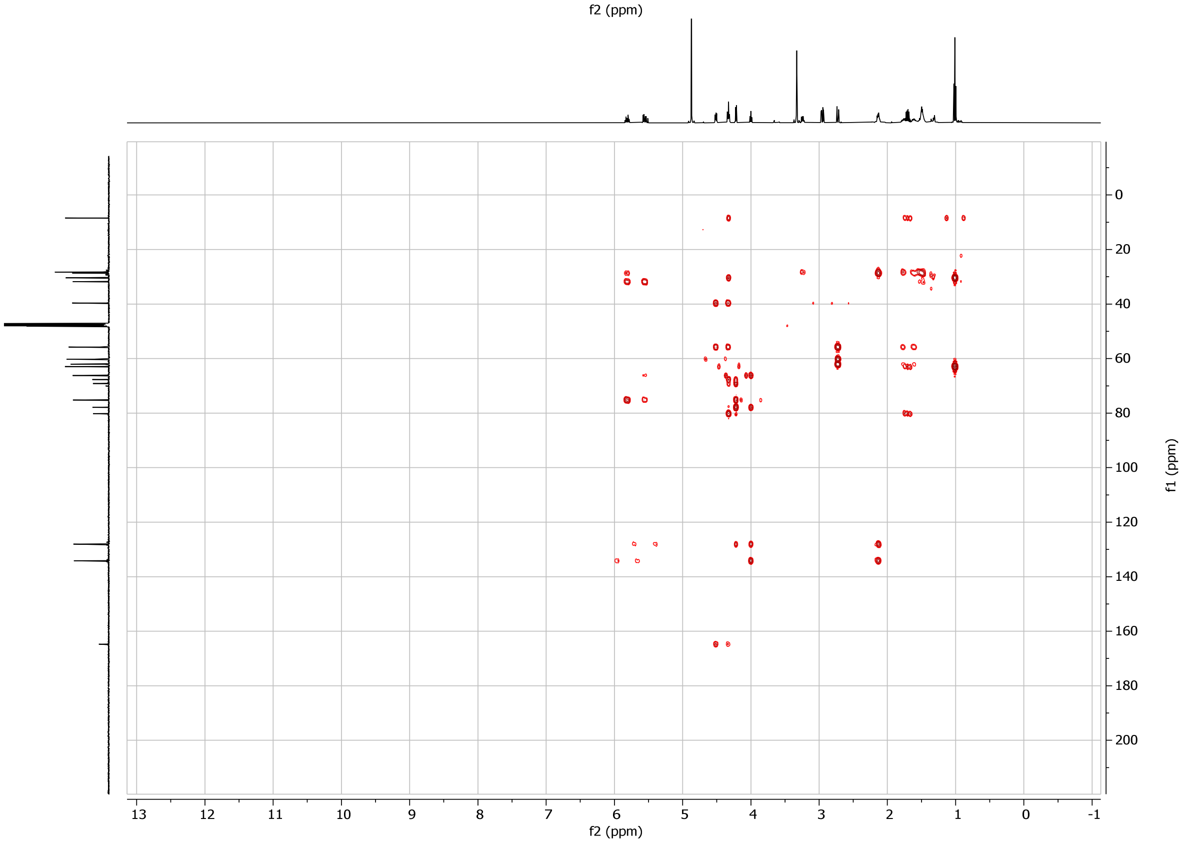

f1 (ppm)
f2 (ppm)

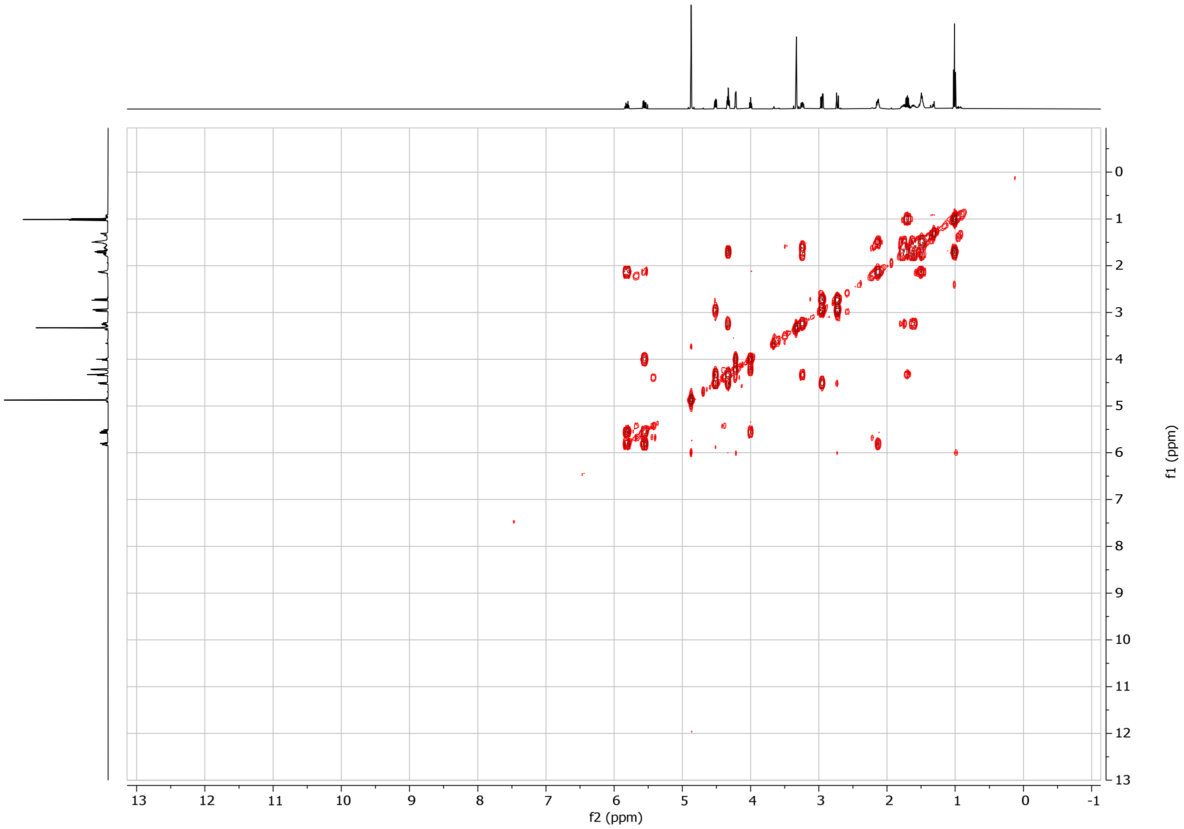
f2 (ppm)
f1 (ppm)

14 Synthesis of Clickable Isofalcarintriol Derivate

Synthesis of S22 (^{1}H, ^{13}C, HSQC, HMBC, COSY spectra)

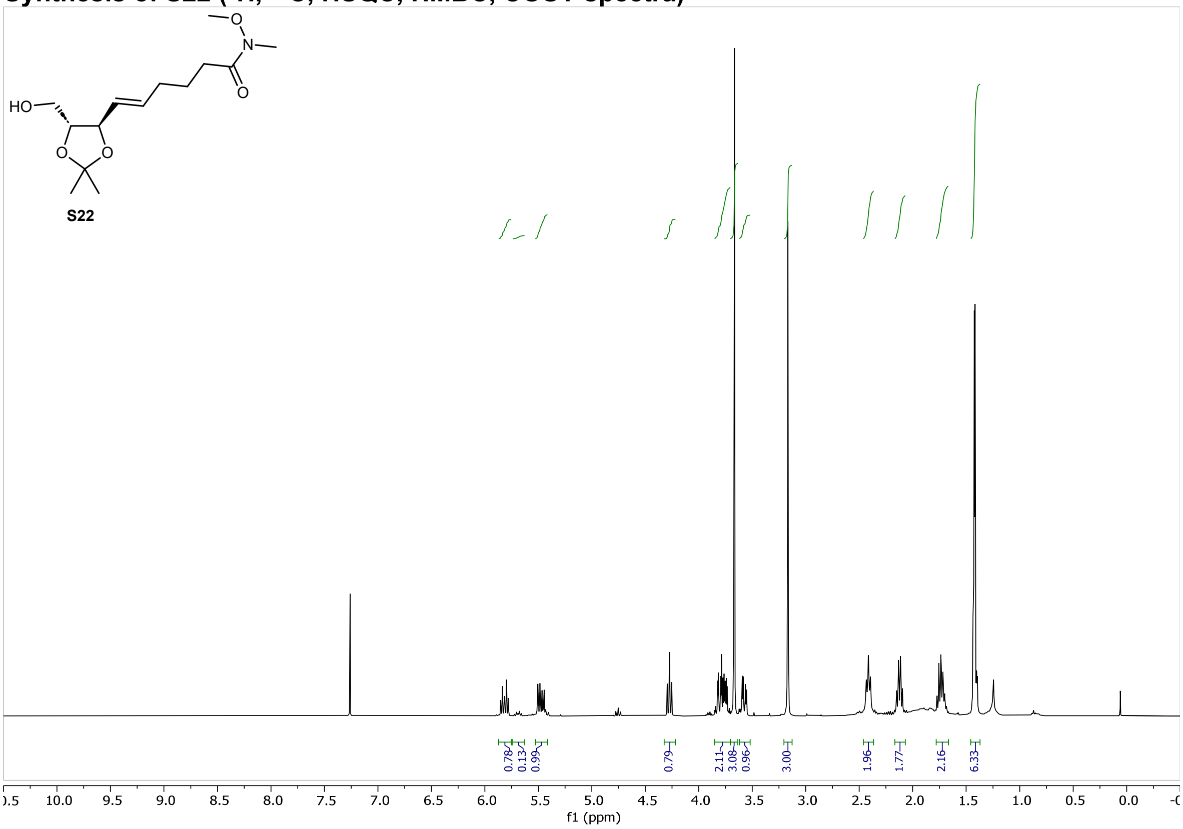

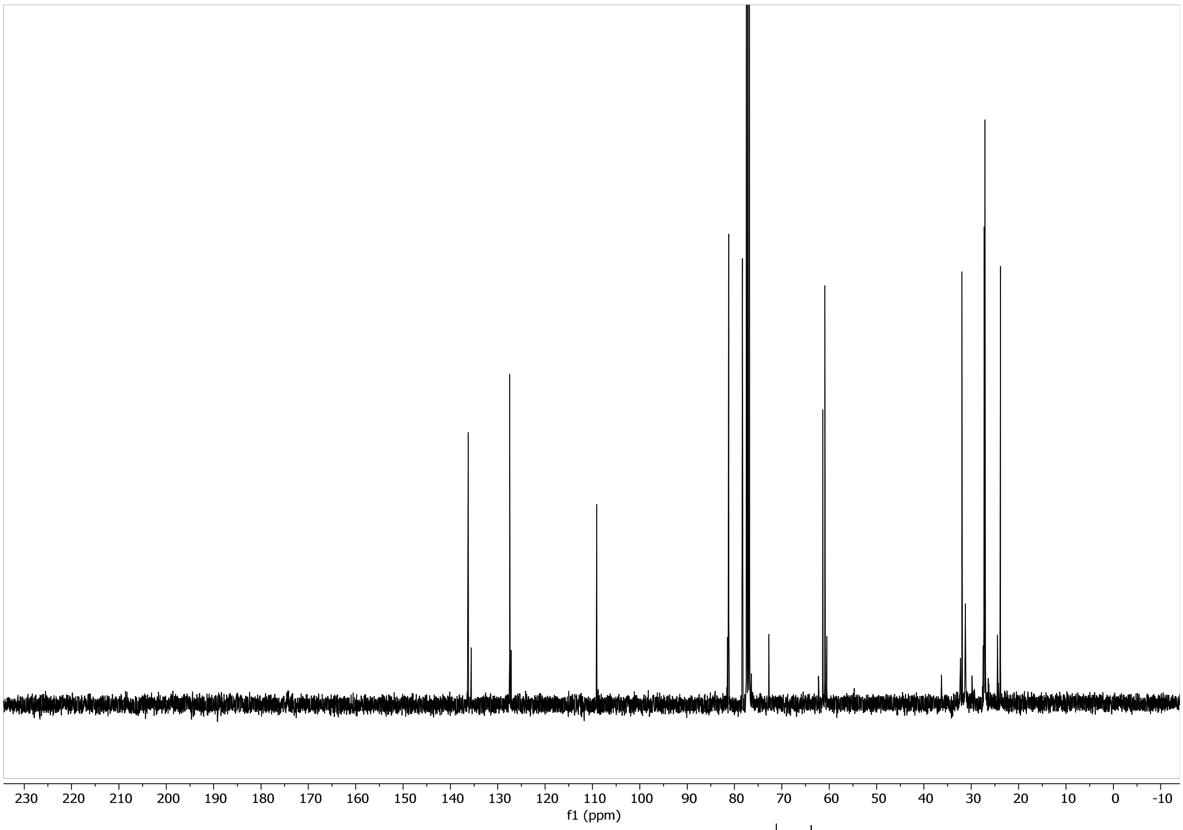

230
220
210
200
190
180
170
160
150
140
130
120
110
100
90
80
70
60
50
40
30
20
10
0
-10
f1 (ppm)

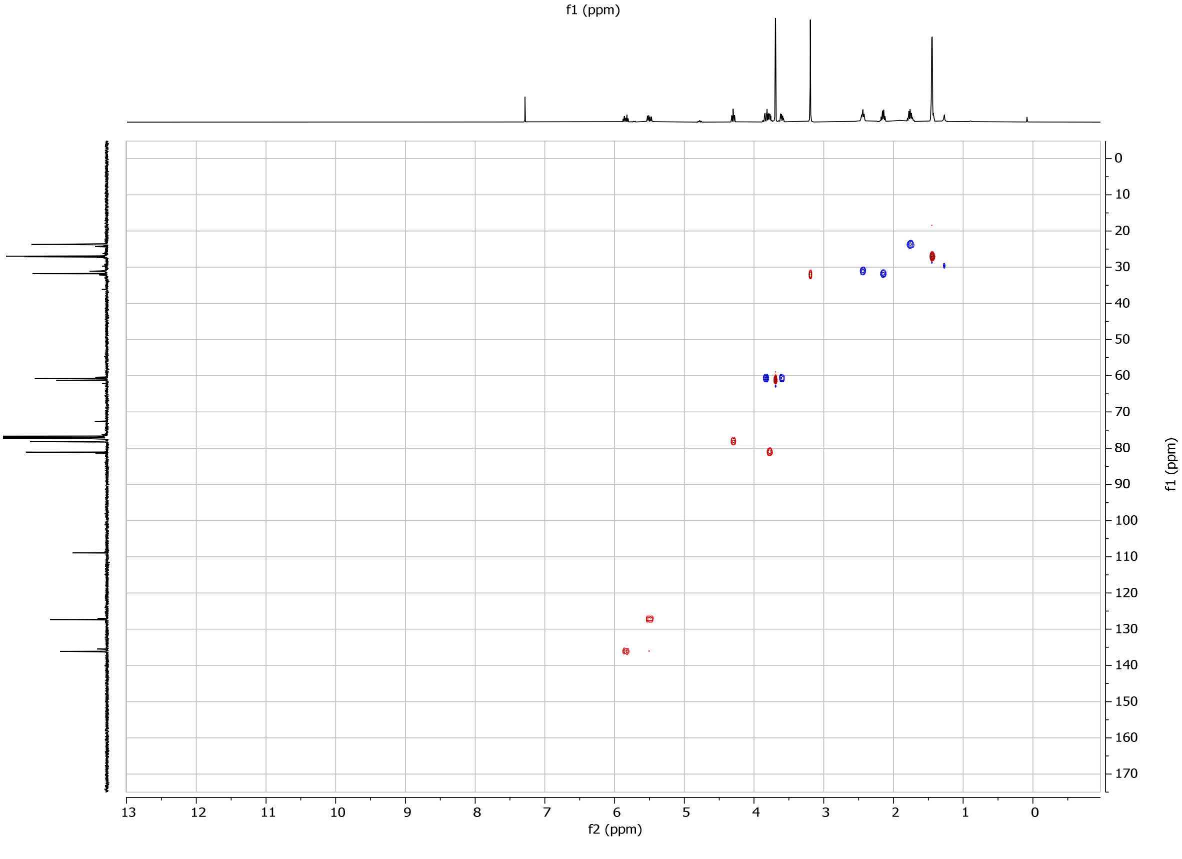

13
12
11
10
9
8
7
6
5
4
3
2
1
0
f2 (ppm)
0
10
20
30
40
50
60
70
80
90
100
110
120
130
140
150
160
170
f1 (ppm)

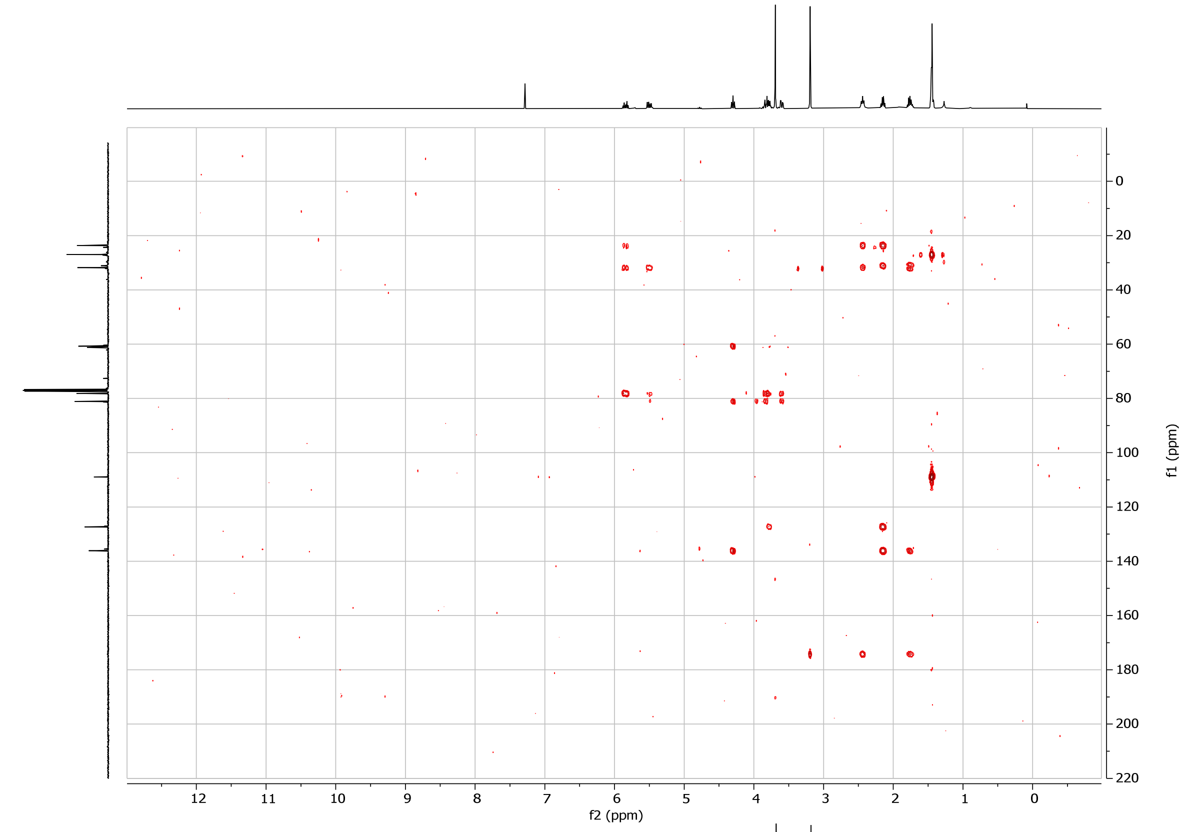
f2 (ppm)
f1 (ppm)

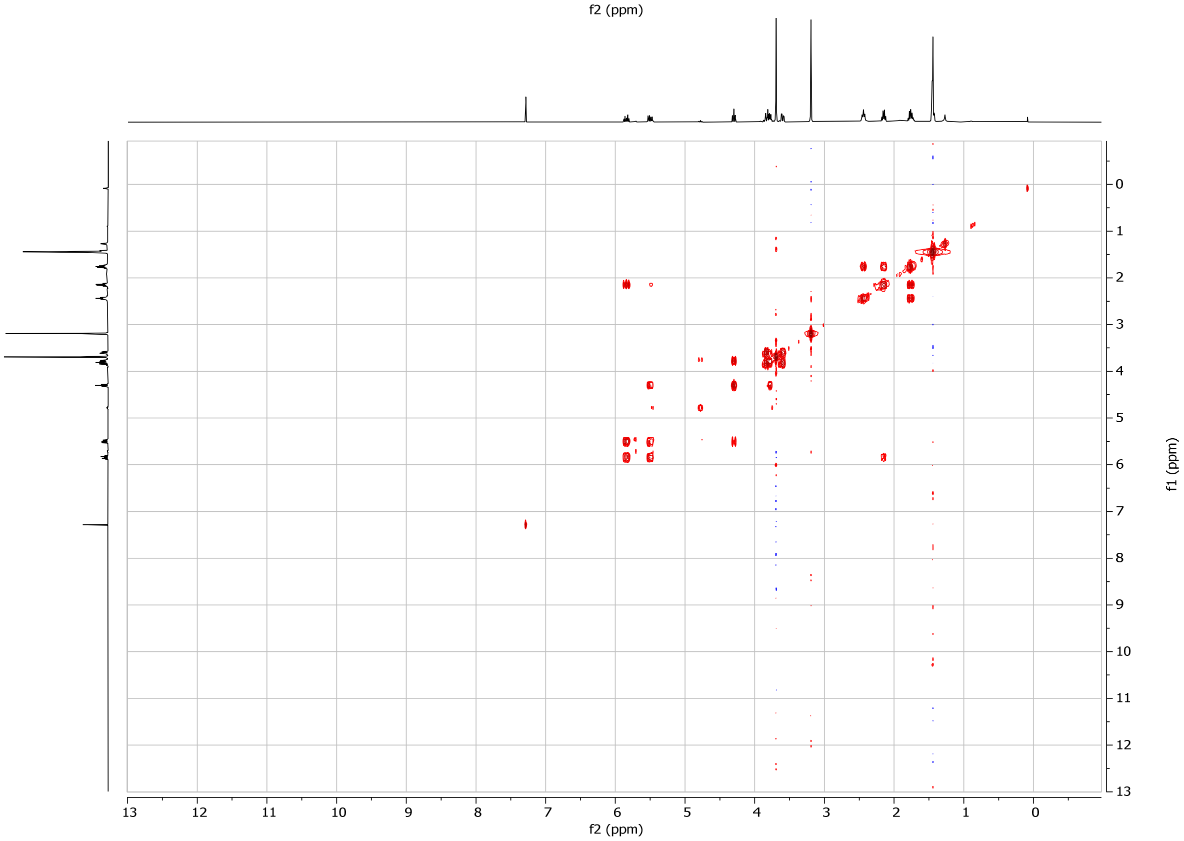
f2 (ppm)
f1 (ppm)

Synthesis of S23 (^{1}H, ^{13}C, HSQC, HMBC, COSY spectra)

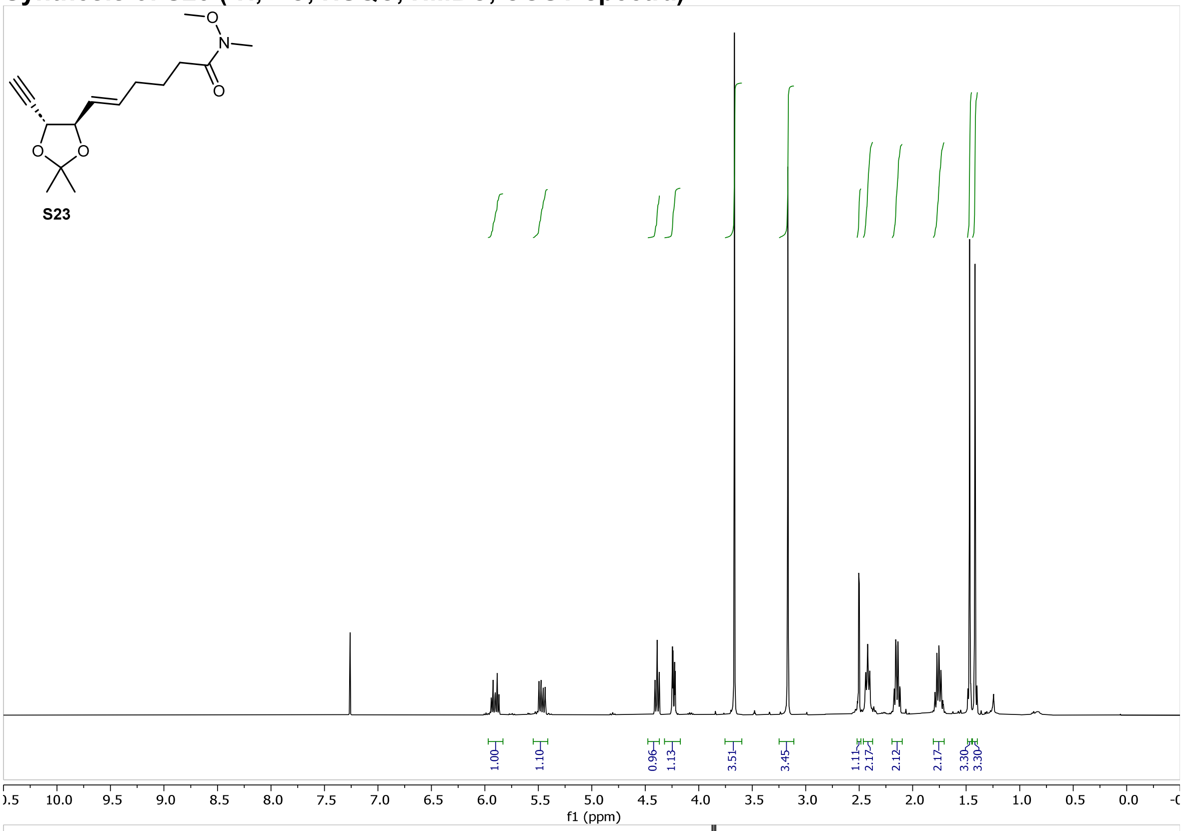

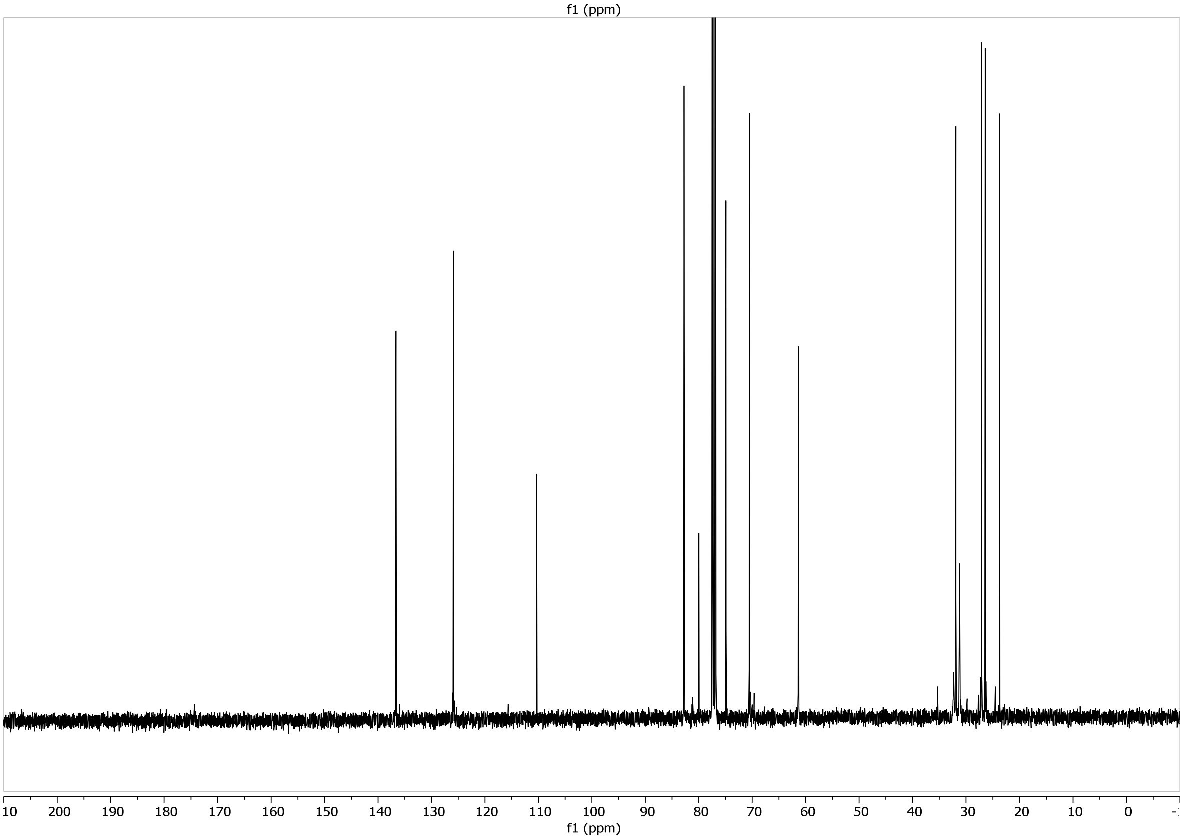

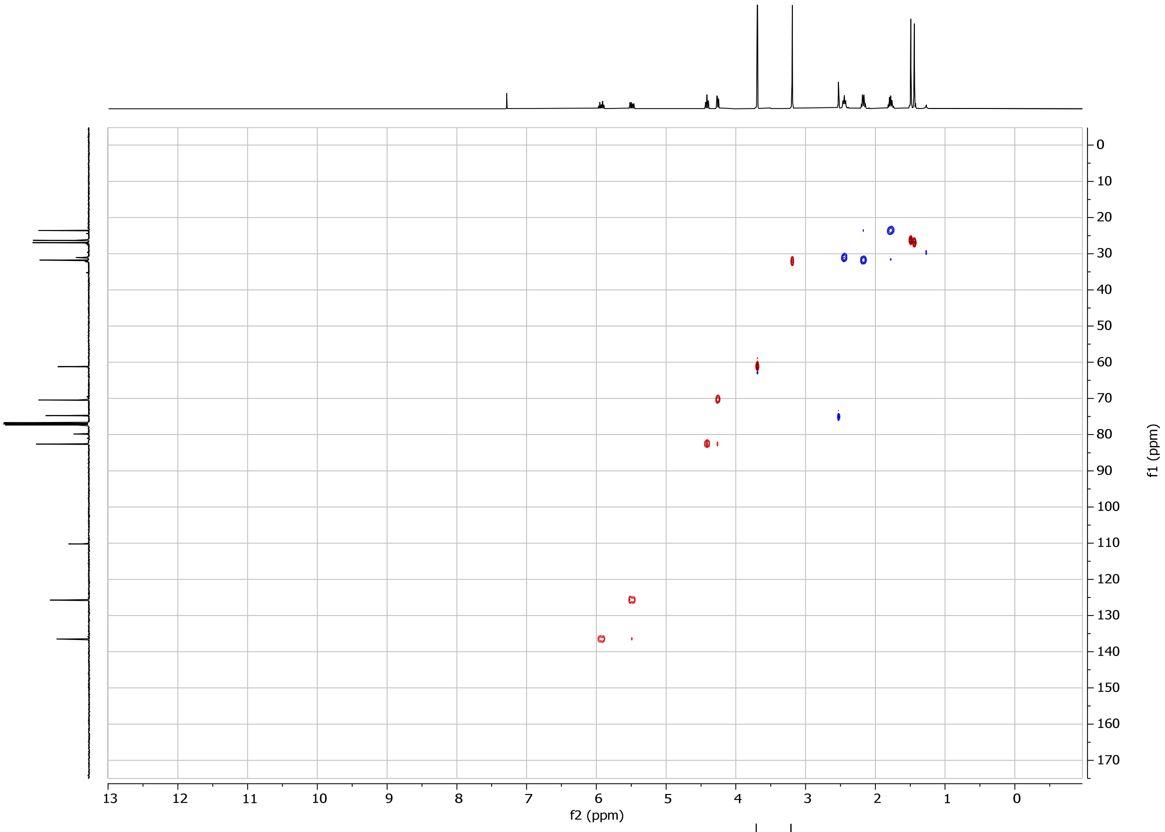
f2 (ppm)
f1 (ppm)

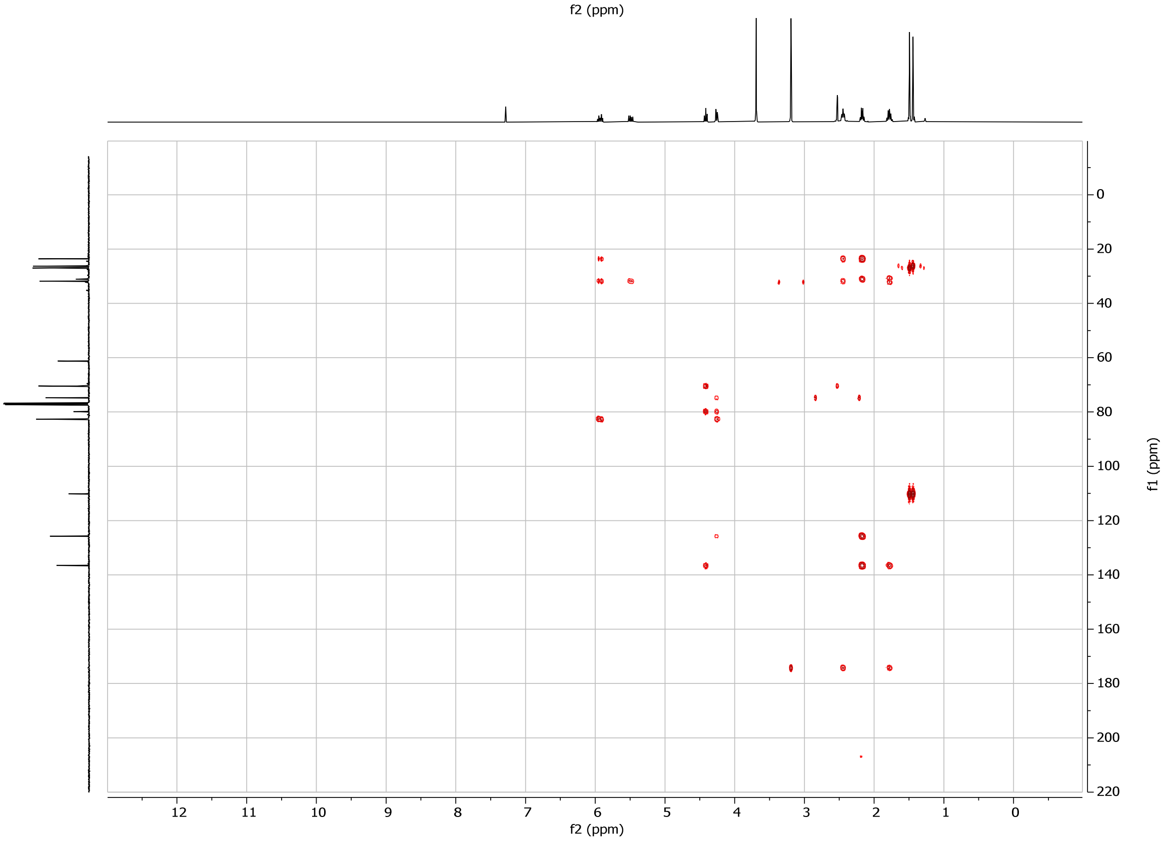
f2 (ppm)
f1 (ppm)

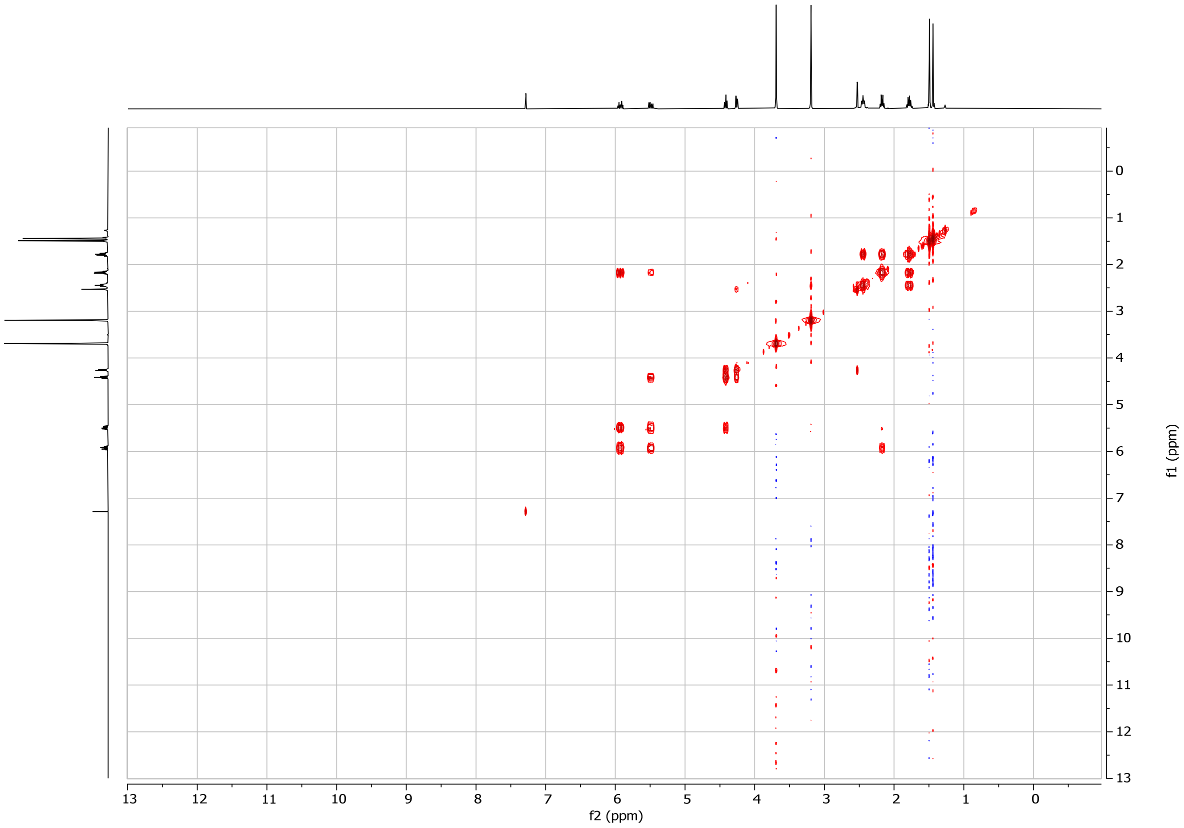

f2 (ppm)
f1 (ppm)

Synthesis of S24 (^{1}H, ^{13}C, HSQC, HMBC, COSY spectra)

500 MHz

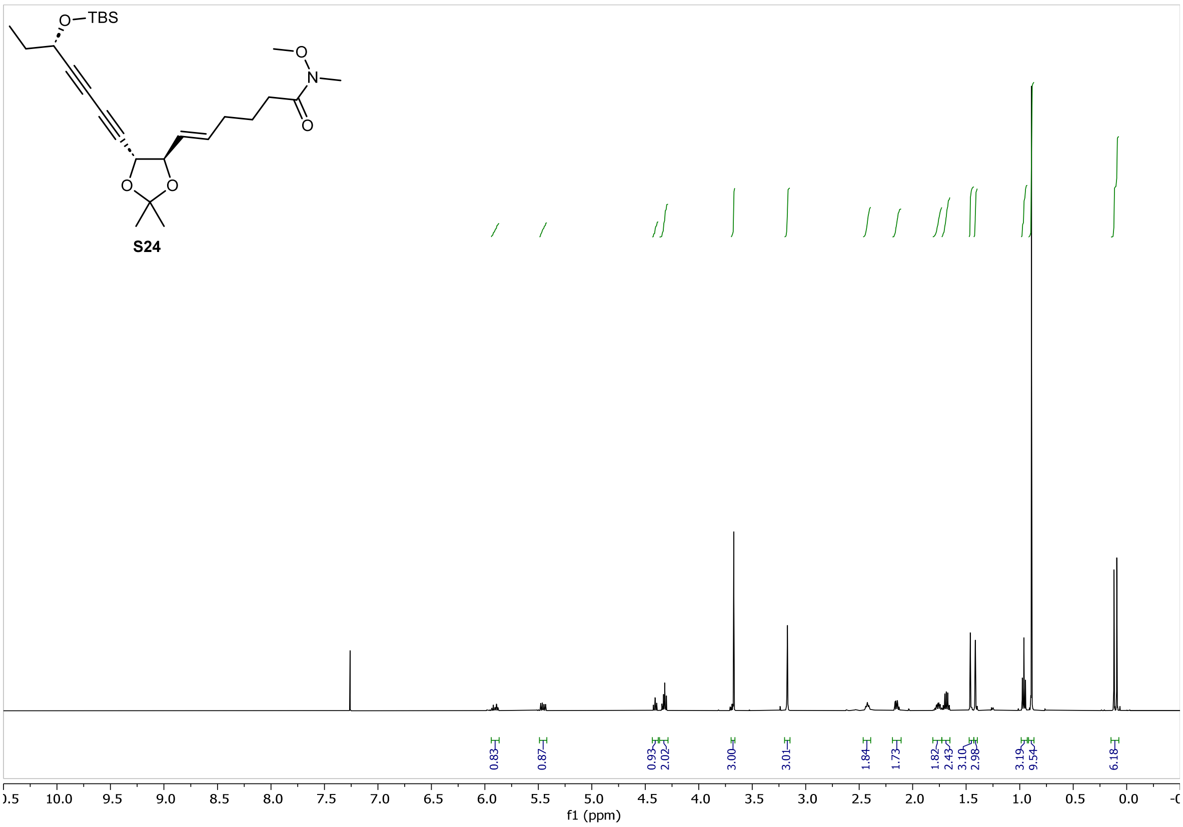

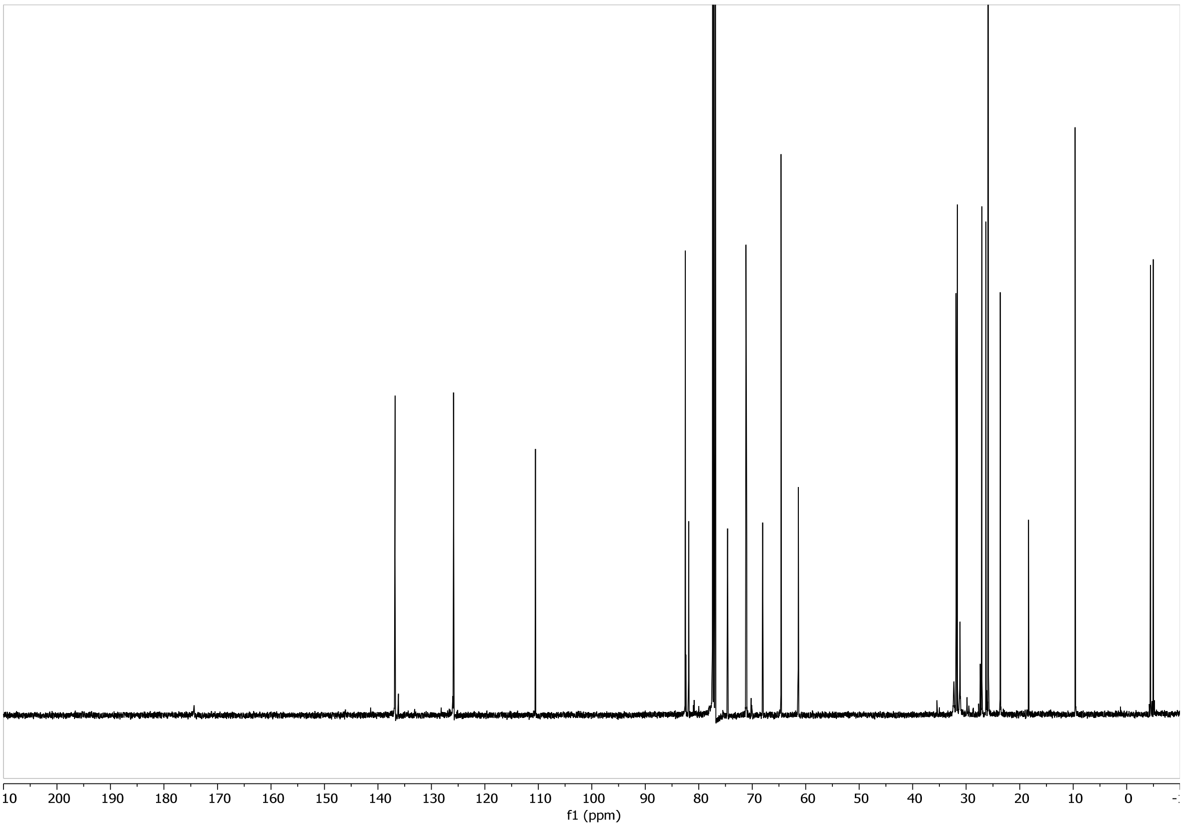

400 MHz

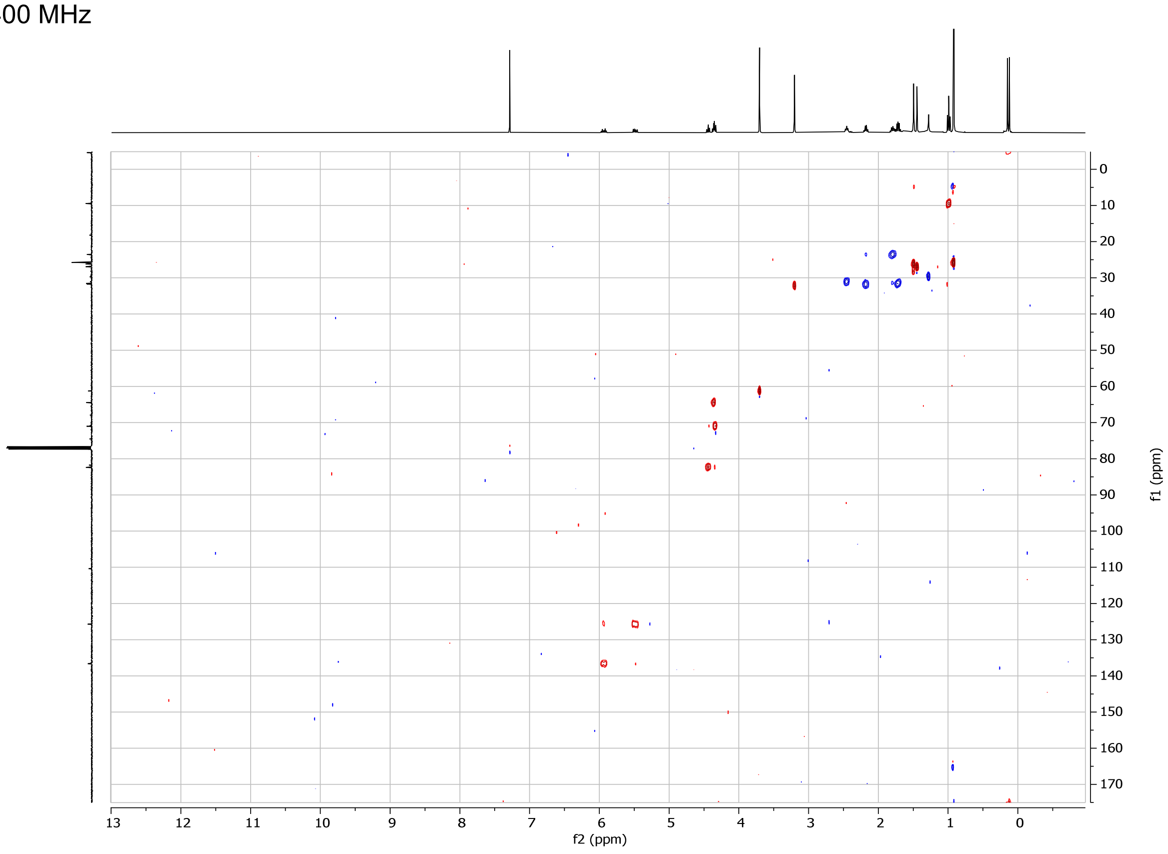

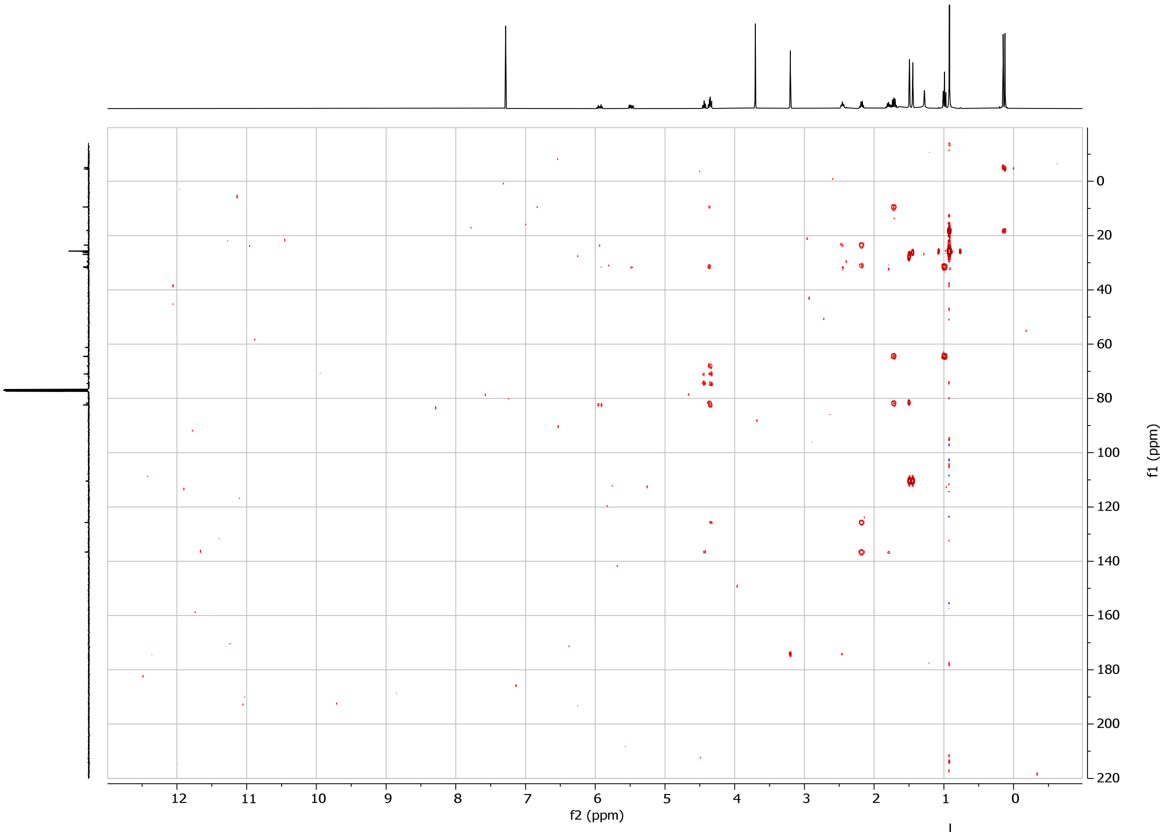
f2 (ppm)
f1 (ppm)

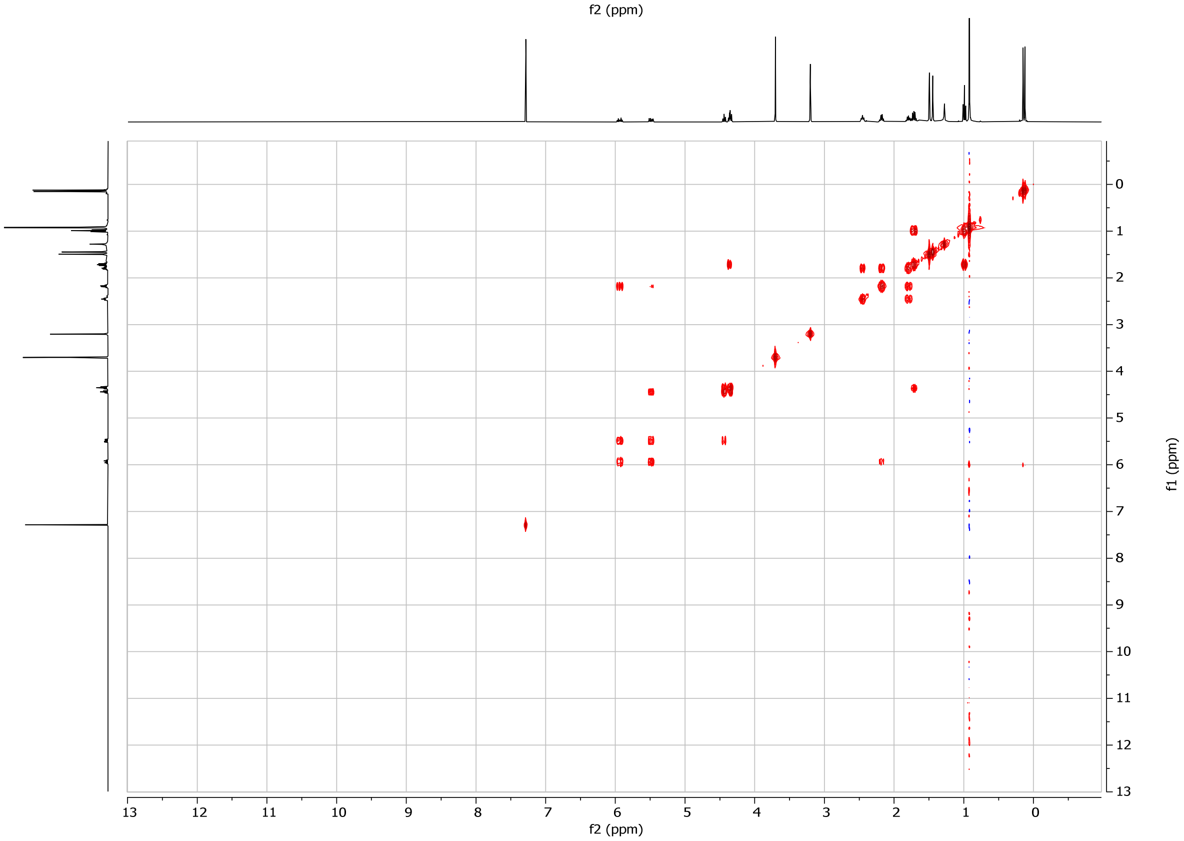
f2 (ppm)
f1 (ppm)

Synthesis of S28 (^{1}H, ^{13}C, HSQC, HMBC, COSY spectra)

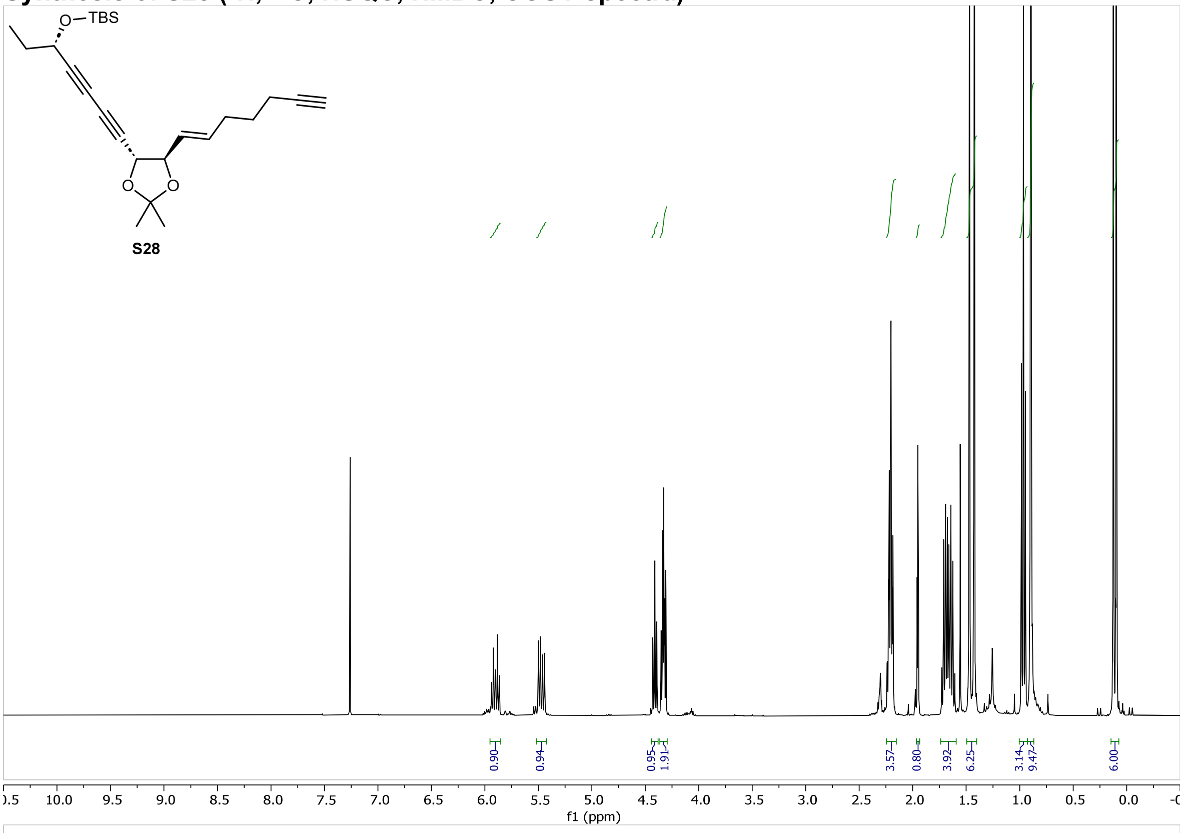

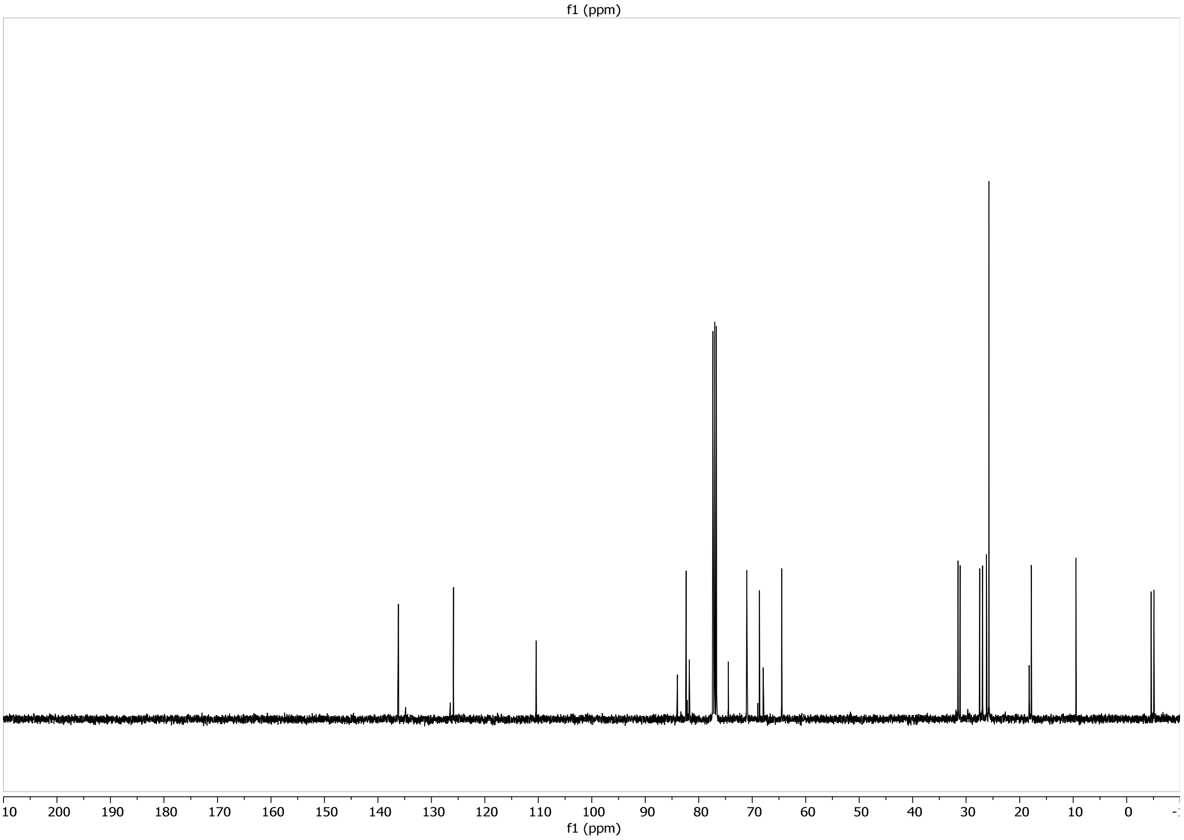

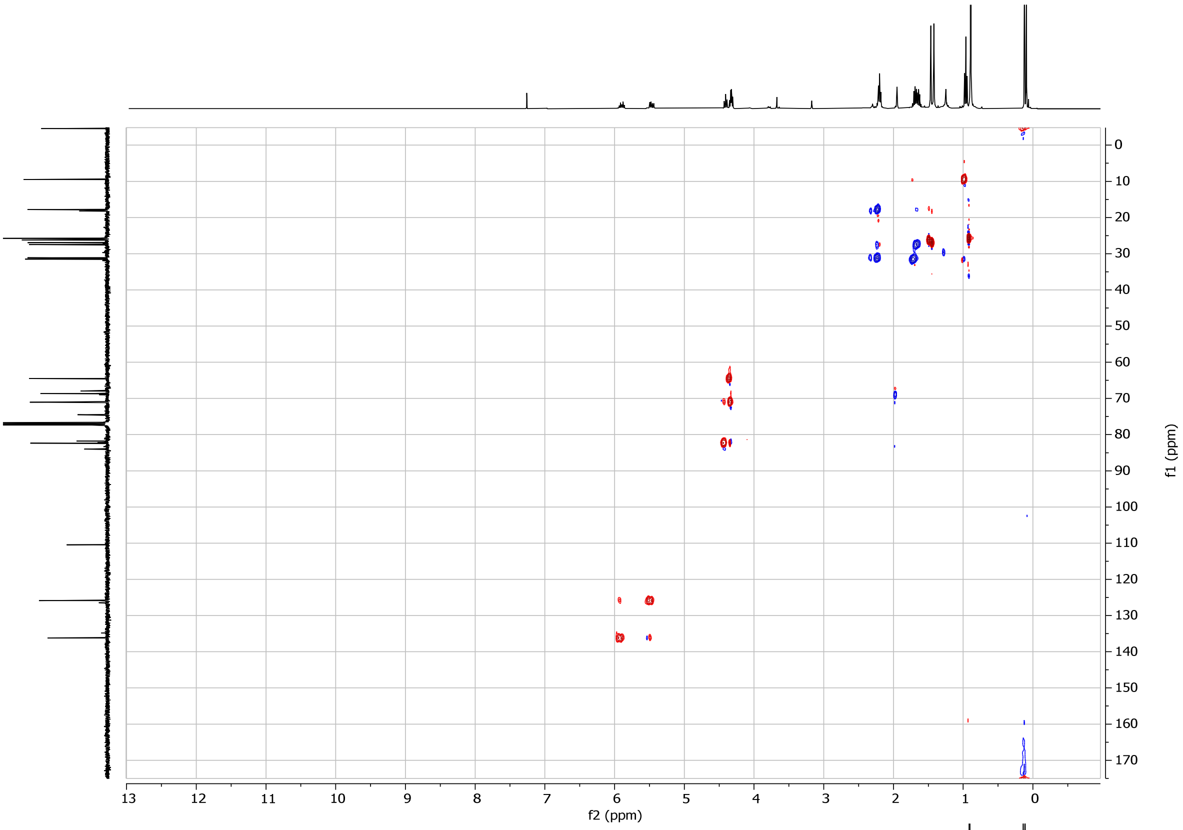
f2 (ppm)
f1 (ppm)

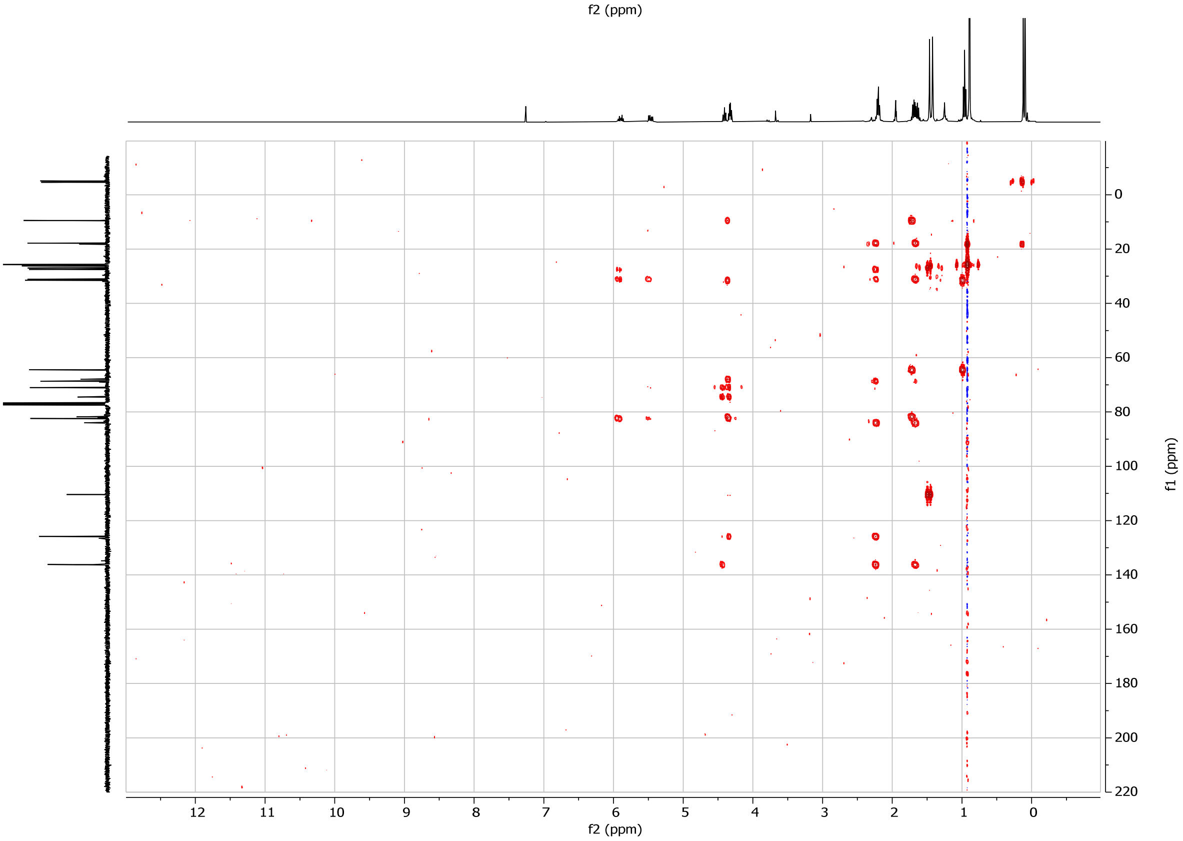
f2 (ppm)
f1 (ppm)

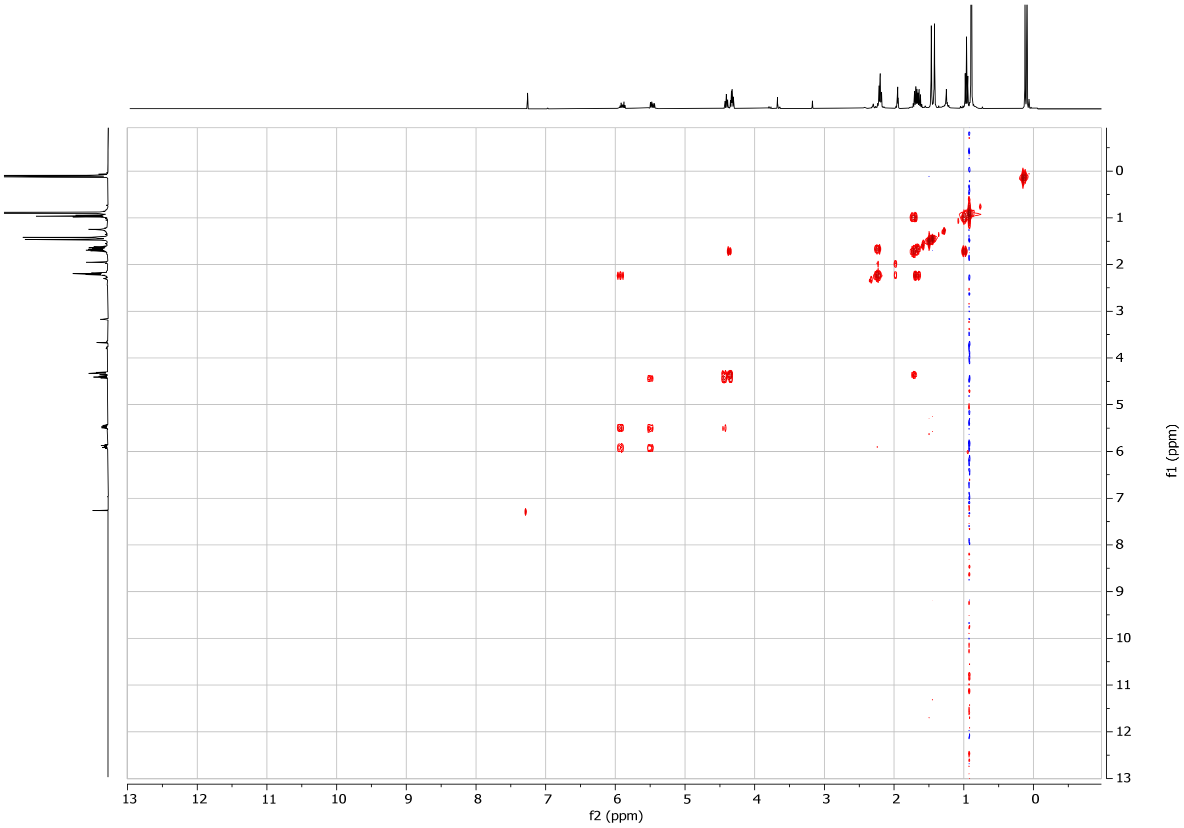

f2 (ppm)
f1 (ppm)

Synthesis of 27 (^{1}H, ^{13}C, HSQC, HMBC, COSY spectra)

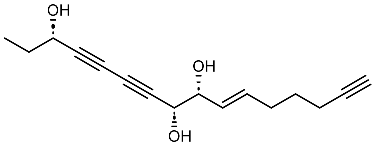

27

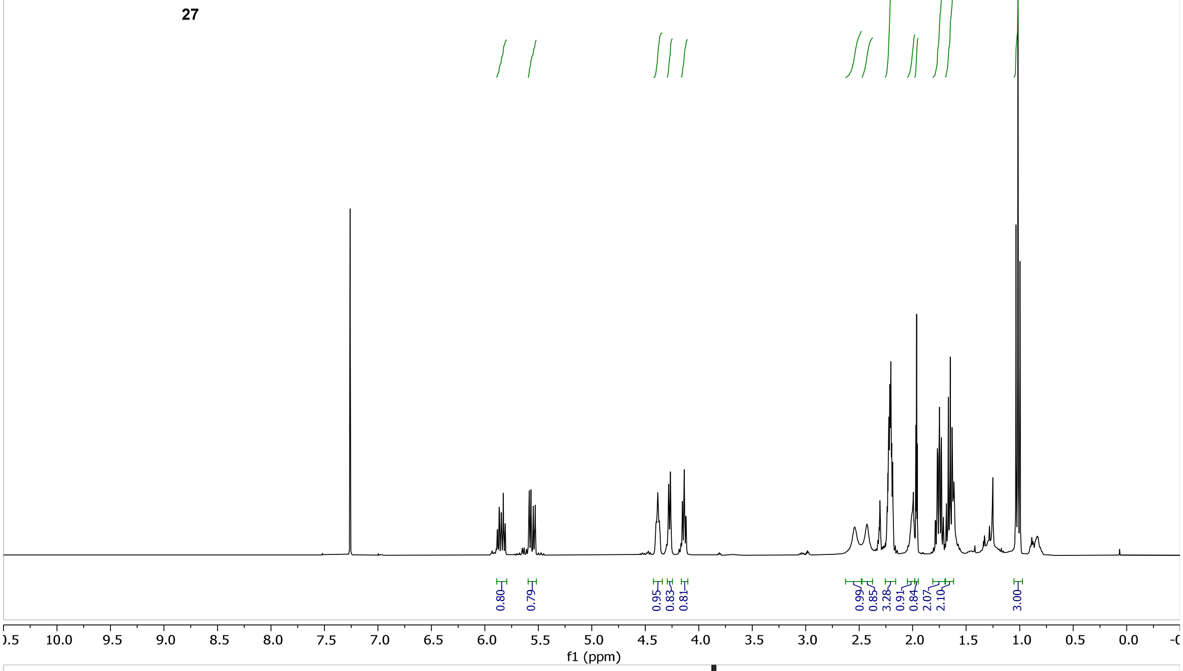

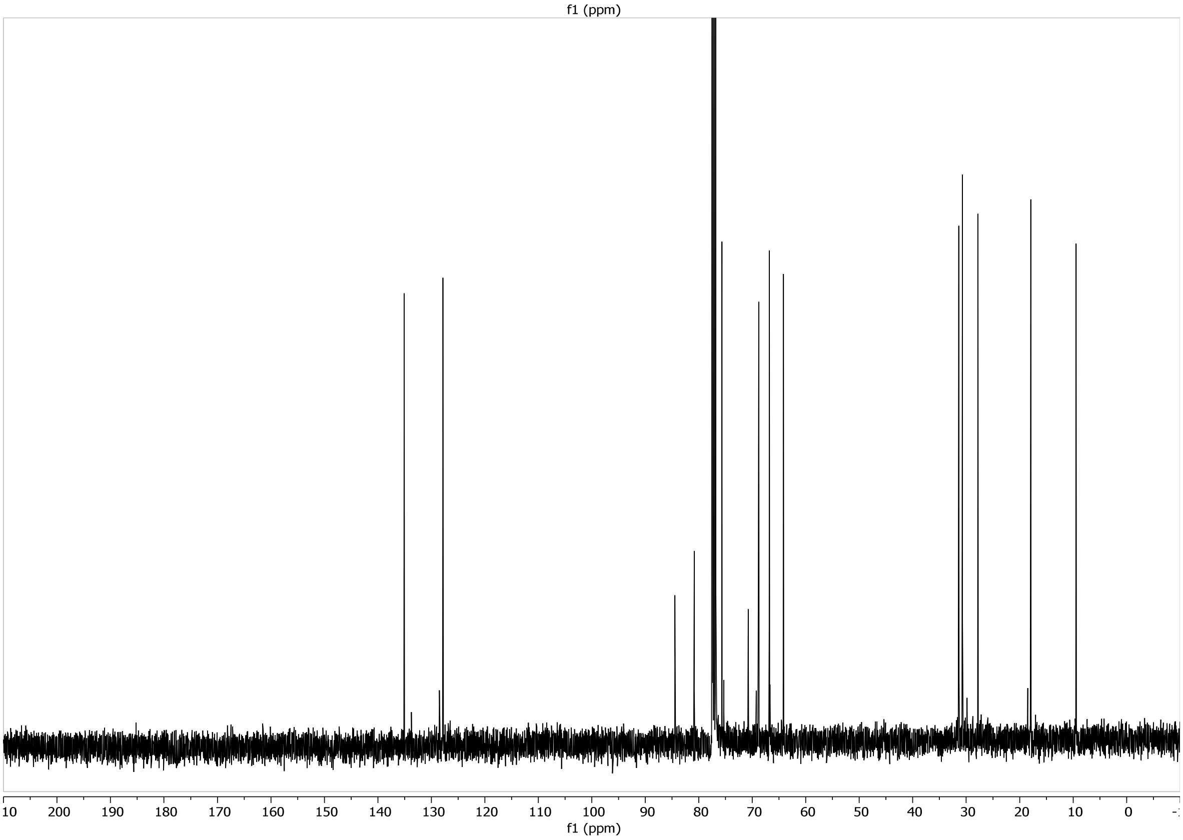

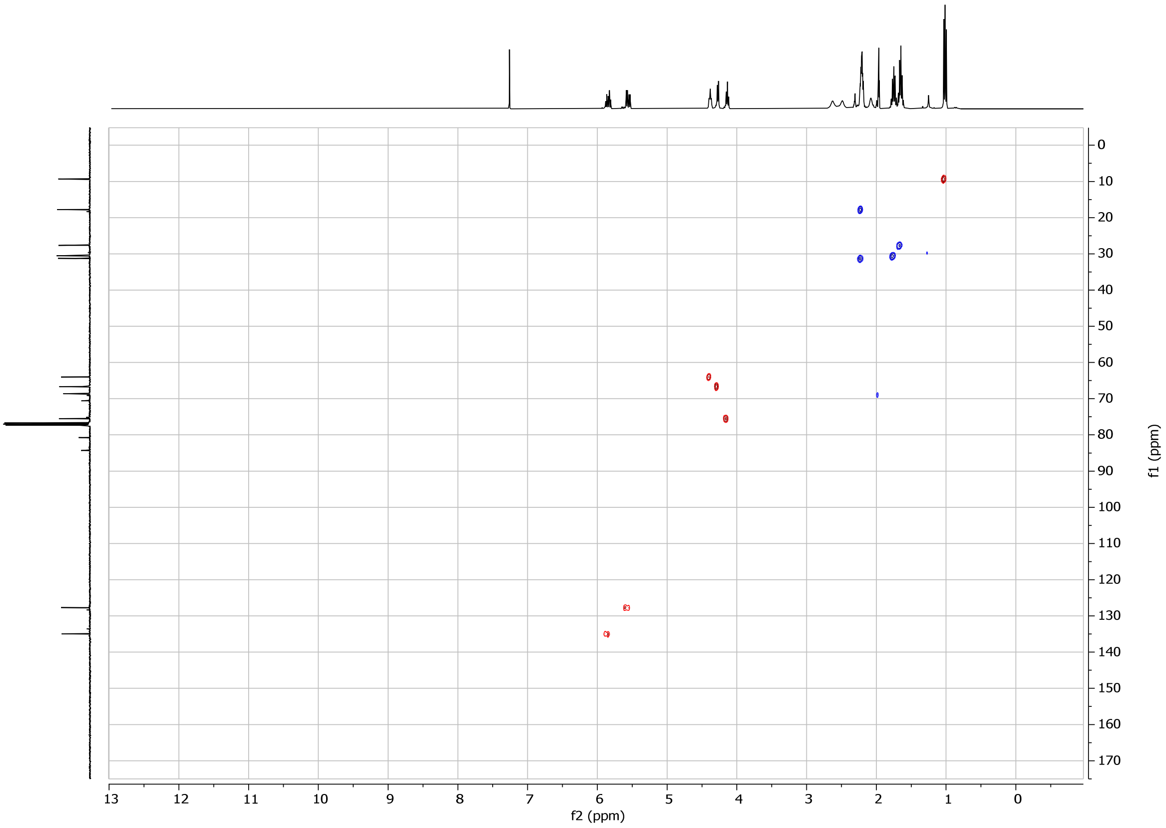
f2 (ppm)
f1 (ppm)

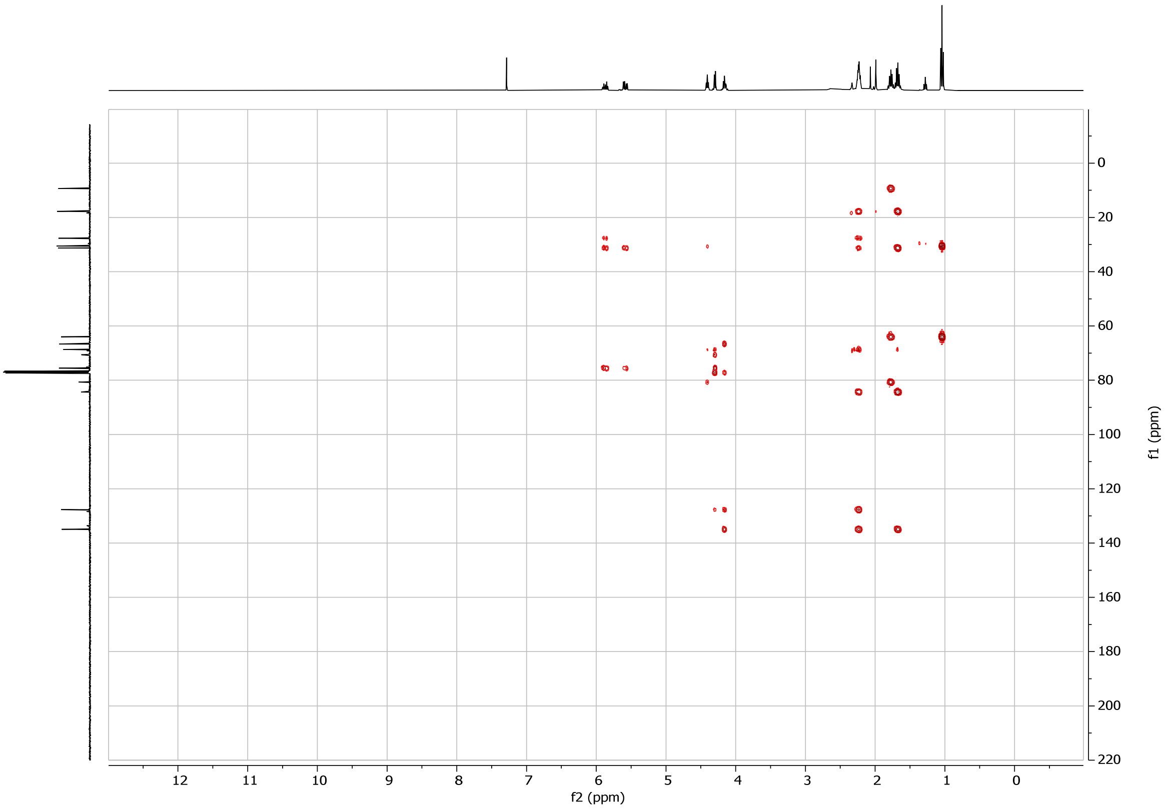
f2 (ppm)
f1 (ppm)

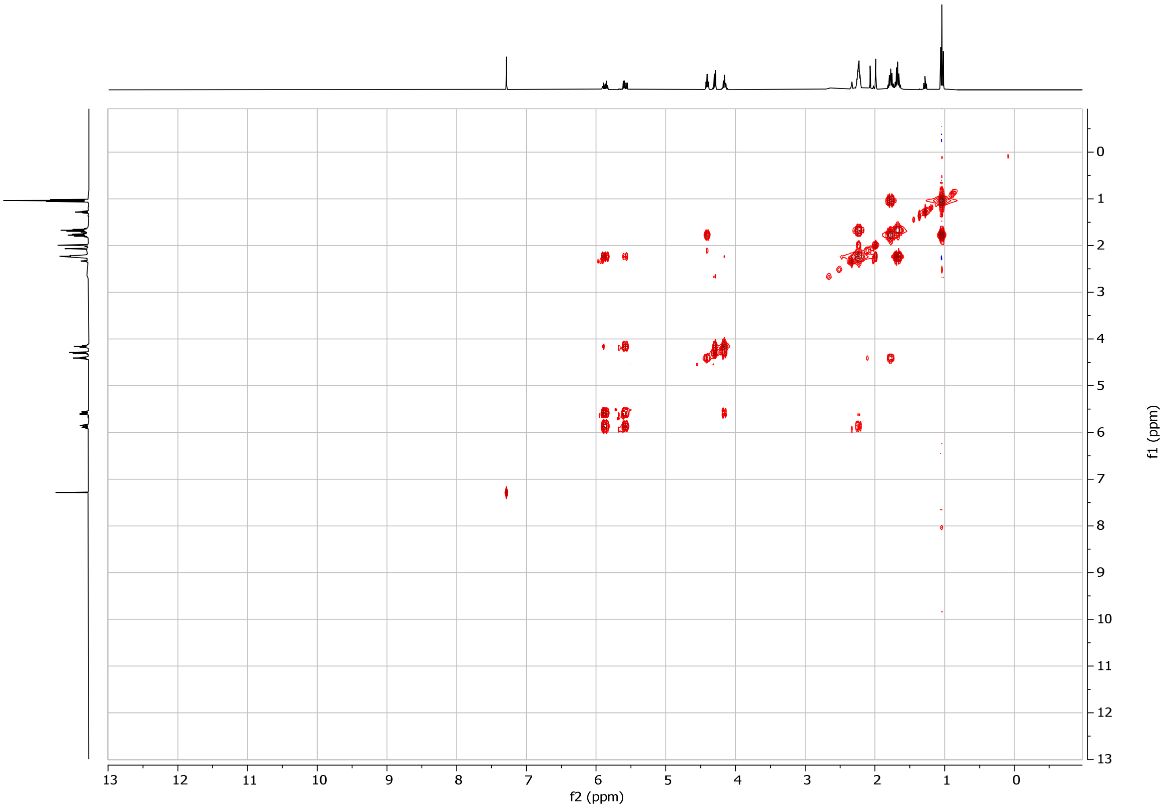

f2 (ppm)
f1 (ppm)

Supplementary References

1 Sabitha, G., Bhaskar, V., Reddy, C. S. & Yadav, J. S. Stereoselective approaches for the total synthesis of polyacetylenic (3R,8S)-falcarindiol. *Synthesis-Stuttgart*, 115-121, (2008).

2 Mao, J. Y. *et al.* Synthesis of panaxytriol and its stereoisomers as potential antitumor drugs. *Tetrahedron-Asymmetr* **27**, 330-337, (2016).

3 Yadav, J. S. *et al.* Stereoselective total synthesis of (+)-oploxyne A, (-)-oploxyne B, and their C-10 epimers and structure revision of natural oploxyne B. *J Org Chem* **76**, 2568-2576, (2011).

4 Schmiech, L., Alayrac, C., Witulski, B. & Hofmann, T. Structure determination of bisacetylenic oxylipins in carrots (Daucus carota L.) and enantioselective synthesis of falcarindiol. *J Agric Food Chem* **57**, 11030-11040, (2009).

5 Brock, E. A., Davies, S. G., Lee, J. A., Roberts, P. M. & Thomson, J. E. Polyhydroxylated pyrrolizidine alkaloids from transannular iodoaminations: application to the asymmetric syntheses of (-)-hyacinthacine A1, (-)-7a-epi-hyacinthacine A1, (-)-hyacinthacine A2, and (-)-1-epi-alexine. *Org Biomol Chem* **11**, 3187-3202, (2013).

6 Moon, H. R., Choi, W. J., Kim, H. O. & Jeong, L. S. Improved and alternative synthesis of D- and L-cyclopentenone derivatives, the versatile intermediates for the synthesis of carbocyclic nucleosides. *Tetrahedron-Asymmetr* **13**, 1189-1193, (2002).

7 Lian, X. & Ma, S. An efficient approach to substituted 1,5,7,8,9-pentahydrocyclopenta[h]-2-benzopyran-3-one derivatives by a palladium-catalyzed tandem reaction of 2,7-alkadiynylic carbonates with 2,3-allenoic acids. *Angew Chem Int Ed Engl* **47**, 8255-8258, (2008).

8 Allevi, P., Ciuffreda, P. & Anastasia, M. Lipase catalysed resolution of (R)- and (S)-1-trimethylsilyl-1-alkyn-3-ols: useful intermediates for the synthesis of optically active γ-lactones. *Tetrahedron: Asymmetry* **8**, 93-99, (1997).

9 Metzger, B. T. & Barnes, D. M. Polyacetylene diversity and bioactivity in orange market and locally grown colored carrots (Daucus carota L.). *J Agric Food Chem* **57**, 11134-11139, (2009).

10 Christensen, L. P. & Kreutzmann, S. Determination of polyacetylenes in carrot roots (Daucus carota L.) by high-performance liquid chromatography coupled with diode array detection. *J Sep Sci* **30**, 483-490, (2007).

11 Busta, L. *et al.* Identification of Genes Encoding Enzymes Catalyzing the Early Steps of Carrot Polyacetylene Biosynthesis. *Plant Physiol* **178**, 1507-1521, (2018).

12 Yang, Q., Xiao, W. J. & Yu, Z. Lewis acid assisted ring-closing metathesis of chiral diallylamines: an efficient approach to enantiopure pyrrolidine derivatives. *Org Lett* **7**, 871-874, (2005).

13 Ran, F. A. *et al.* Genome engineering using the CRISPR-Cas9 system. *Nat Protoc* **8**, 2281-2308, (2013).

14 Rozanov, L. *et al.* Redox-mediated regulation of aging and healthspan by an evolutionarily conserved transcription factor HLH-2/Tcf3/E2A. *Redox biology* **32**, 101448, (2020).

15 Grigolon, G. *et al.* Grainyhead 1 acts as a drug-inducible conserved transcriptional regulator linked to insulin signaling and lifespan. *Nat Commun* **13**, 107, (2022).

16 Rappsilber, J., Mann, M. & Ishihama, Y. Protocol for micro-purification, enrichment, pre-fractionation and storage of peptides for proteomics using StageTips. *Nat Protoc* **2**, 1896-1906, (2007).

17 Cox, J. *et al.* Andromeda: a peptide search engine integrated into the MaxQuant environment. *J Proteome Res* **10**, 1794-1805, (2011).

18 Perez-Riverol, Y. *et al.* The PRIDE database resources in 2022: a hub for mass spectrometry-based proteomics evidences. *Nucleic Acids Res* **50**, D543-D552, (2022).

19 Johansson, L. H. & Borg, L. A. A spectrophotometric method for determination of catalase activity in small tissue samples. *Anal Biochem* **174**, 331-336 (1988).

20 Peskin, A. V. & Winterbourn, C. C. A microtiter plate assay for superoxide dismutase using a water-soluble tetrazolium salt (WST-1). *Clin Chim Acta* **293**, 157-166, (2000).

21 Zarse, K. *et al.* Impaired insulin/IGF1 signaling extends life span by promoting mitochondrial L-proline catabolism to induce a transient ROS signal. *Cell Metab* **15**, 451-465, (2012).
22 Huang, W., Sherman, B. T. & Lempicki, R. A. Systematic and integrative analysis of large gene lists using DAVID bioinformatics resources. *Nat Protoc* **4**, 44-57, (2009).
23 Huang da, W., Sherman, B. T. & Lempicki, R. A. Bioinformatics enrichment tools: paths toward the comprehensive functional analysis of large gene lists. *Nucleic Acids Res* **37**, 1-13, (2009).
24 Venegas, V. & Halberg, M. C. Measurement of mitochondrial DNA copy number. *Methods Mol Biol* **837**, 327-335, (2012).
25 Gonzalez-Hunt, C. P. *et al.* PCR-Based Analysis of Mitochondrial DNA Copy Number, Mitochondrial DNA Damage, and Nuclear DNA Damage. *Curr Protoc Toxicol* **67**, 20 11 21-20 11 25, (2016).
26 Li, T. S., Li, J. T. & Li, H. Z. Modified and convenient preparation of silica impregnated with silver nitrate and its application to the separation of steroids and triterpenes. *Journal of Chromatography A* **715**, 372-375, (1995).
27 Kumaraswamy, G. & Sadaiah, K. A flexible organocatalytic enantioselective synthesis of heptadeca-1-ene-4,6-diyne-3S,8R,9S,10S-tetrol and its congeners. *Tetrahedron* **68**, 262-271, (2012).
28 Denmark, S. E. & Yang, S. M. Total synthesis of (+)-brasilenyne. Application of an intramolecular silicon-assisted cross-coupling reaction. *J Am Chem Soc* **126**, 12432-12440, (2004).
29 Ghosh, S. *et al.* The thiazolidinedione pioglitazone alters mitochondrial function in human neuron-like cells. *Mol Pharmacol* **71**, 1695-1702, (2007).
30 Dahlhoff, W. V. Amphiphilic Carbohydrate-Based Mesogens, VIII. A Facile Synthetic Route to Mesogenic L-ribo-1,2,3,4-alkanetetrols. *Liebigs Annalen der Chemie* **1992**, 109-113, (1992).
31 Yadav, J. S., Shankar, K. S., Reddy, A. S. & Reddy, B. V. S. Stereoselective Total Synthesis of Oxylipins: (6S,7E,9R,10S)-6,9,10-Trihydroxyoctadec-7-enoic Acid and (6Z,8R,9R,10S)-8,9,10-Trihydroxyoctadec-6-enoic Acid. *Helvetica Chimica Acta* **97**, 546-555, (2014).
32 Kang, S. K., Kim, Y. S., Lim, J. S., Kim, L. S. & Kim, S. G. Synthesis of chiral epoxy alcohols: synthesis of (+)-disparlure. *Tetrahedron Letters* **32**, 363-366, (1991).
33 Goswami, S. & Dey, S. Directed molecular recognition: design and synthesis of neutral receptors for biotin to bind both its functional groups. *J Org Chem* **71**, 7280-7287, (2006).
34 Satcharoen, V., McLean, N. J., Kemp, S. C., Camp, N. P. & Brown, R. C. Stereocontrolled synthesis of (-)-galanthamine. *Org Lett* **9**, 1867-1869, (2007).
