# Supplementary material for: A naturally occurring polyacetylene isolated from carrots promotes health and delays signatures of aging
Source: Nat Commun. 2023 Dec 8;14:8142. doi: 10.1038/s41467-023-43672-7 (PMC10709416; doi:10.1038/s41467-023-43672-7)
Supplement: Supplementary file 3 — Reporting Summary [file 41467_2023_43672_MOESM3_ESM.pdf]

nature portfolio

Corresponding author(s): Michael Ristow, Erick M. Carreira

Last updated by author(s): Nov 8, 2023

Reporting Summary

Nature Portfolio wishes to improve the reproducibility of the work that we publish. This form provides structure for consistency and transparency in reporting. For further information on Nature Portfolio policies, see our Editorial Policies and the Editorial Policy Checklist.

Statistics

For all statistical analyses, confirm that the following items are present in the figure legend, table legend, main text, or Methods section.

n/a	Confirmed	
☐	☒	The exact sample size (*n*) for each experimental group/condition, given as a discrete number and unit of measurement
☐	☒	A statement on whether measurements were taken from distinct samples or whether the same sample was measured repeatedly
☐	☒	The statistical test(s) used AND whether they are one- or two-sided<br>*Only common tests should be described solely by name; describe more complex techniques in the Methods section.*
☐	☒	A description of all covariates tested
☐	☒	A description of any assumptions or corrections, such as tests of normality and adjustment for multiple comparisons
☐	☒	A full description of the statistical parameters including central tendency (e.g. means) or other basic estimates (e.g. regression coefficient) AND variation (e.g. standard deviation) or associated estimates of uncertainty (e.g. confidence intervals)
☐	☒	For null hypothesis testing, the test statistic (e.g. *F*, *t*, *r*) with confidence intervals, effect sizes, degrees of freedom and *P* value noted<br>*Give P values as exact values whenever suitable.*
☒	☐	For Bayesian analysis, information on the choice of priors and Markov chain Monte Carlo settings
☒	☐	For hierarchical and complex designs, identification of the appropriate level for tests and full reporting of outcomes
☒	☐	Estimates of effect sizes (e.g. Cohen's *d*, Pearson's *r*), indicating how they were calculated

Our web collection on statistics for biologists contains articles on many of the points above.

Software and code

Policy information about availability of computer code

Data collection	Data were collected in Microsoft Office (Version 2310), CorelDraw X7, ExpeData 1.9.27
Data analysis	Data analysis was done with Microsoft Excel (Version 2310), GraphPad Prism 9.2, JMP 15, Fiji 1.51n, Wave Desktop 2.6.<br><br>For analysis of metabolic cage data, the web-based tool CalR (Version 2.1) was used (Mina, A. I. et al. CalR: A Web-Based Analysis Tool for Indirect Calorimetry Experiments. Cell Metab 28, 656-666 e651, doi:10.1016/j.cmet.2018.06.019 (2018).<br><br>To calculate murine age and life expectancy clocks, an algorithm from http://frailtyclocks.sinclairlab.org/ was used.

For manuscripts utilizing custom algorithms or software that are central to the research but not yet described in published literature, software must be made available to editors and reviewers. We strongly encourage code deposition in a community repository (e.g. GitHub). See the Nature Portfolio guidelines for submitting code & software for further information.

Data

Policy information about availability of data

All manuscripts must include a data availability statement. This statement should provide the following information, where applicable:

- Accession codes, unique identifiers, or web links for publicly available datasets
- A description of any restrictions on data availability
- For clinical datasets or third party data, please ensure that the statement adheres to our policy

The mass spectrometry proteomics data in this study have been deposited to the ProteomeXchange Consortium via the PRIDE48 partner repository with the dataset identifier PXD037671 .All data generated in this study are provided in the Supplementary Information and the Source Data file.

Research involving human participants, their data, or biological material

Policy information about studies with human participants or human data. See also policy information about sex, gender (identity/presentation), and sexual orientation and race, ethnicity and racism.

Reporting on sex and gender	N/A
Reporting on race, ethnicity, or other socially relevant groupings	N/A
Population characteristics	N/A
Recruitment	N/A
Ethics oversight	N/A

Note that full information on the approval of the study protocol must also be provided in the manuscript.

Field-specific reporting

Please select the one below that is the best fit for your research. If you are not sure, read the appropriate sections before making your selection.

[x] Life sciences [ ] Behavioural & social sciences [ ] Ecological, evolutionary & environmental sciences

For a reference copy of the document with all sections, see nature.com/documents/nr-reporting-summary-flat.pdf

Life sciences study design

All studies must disclose on these points even when the disclosure is negative.

Sample size	The sample size for mouse cohorts was based on previous experience and published data and was validated via sample size calculation by G*Power (https://www.psychologie.hhu.de/arbeitsgruppen/allgemeine-psychologie-und-arbeitspsychologie/gpower.html)<br><br>The sample size used for all other experiments were chosen based on previous experience (20 + years experience of corresponding author)
Data exclusions	Outliers were excluded based on the ESD (extreme studentized deviate) method with an alpha = 0.05 threshold
Replication	All experiments were conducted in at least 3 technical replicates. Most experiments were further assessed in several independent replicates/ biological replicates. All replication were successful in validating initial results.
Randomization	Mouse studies: Randomization was done by stratified random allocation to treatment groups based on sex, body weight, and body composition.<br><br>Randomization for all other experiments was omitted as each C. elegans plate and cell culture vessel contains individual randomized cells/ organisms.
Blinding	Mouse studies: The investigators were blinded during the performance and analysis of mouse experiments<br><br>Blinding was not relevant for all other experiments as the analysis was conducted in an unbiased way by the analysis software.

Reporting for specific materials, systems and methods

We require information from authors about some types of materials, experimental systems and methods used in many studies. Here, indicate whether each material, system or method listed is relevant to your study. If you are not sure if a list item applies to your research, read the appropriate section before selecting a response.

Materials & experimental systems

n/a	Involved in the study
[ ]	[x] Antibodies
[ ]	[x] Eukaryotic cell lines
[x]	[ ] Palaeontology and archaeology
[ ]	[x] Animals and other organisms
[x]	[ ] Clinical data
[x]	[ ] Dual use research of concern
[ ]	[x] Plants

Methods

n/a	Involved in the study
[x]	[ ] ChIP-seq
[x]	[ ] Flow cytometry
[x]	[ ] MRI-based neuroimaging

Antibodies

Antibodies used	phospho-AMPKα (Thr172) (40H9) (Cell signaling; #2535, lot21); AMPKα (Cell signaling; #2532, lot19); actin (Sigma Aldrich; #A5060); HRP linked secondary antibody against rabbit (Cell Signaling, #7074S, lot30)
Validation	phospho-AMPKα (Thr172) (40H9) was validated for applications such as WB, IP, IHC in following species: H, M, R, Hm, Mk, Dm, Sc, Ce; AMPKα (Cell signaling; #2532) was validated for applications such as WB, IP in following species: H, M, R, Hm, Mk; actin (Sigma Aldrich; #A5060) was validated for applications such as WB, IF, IHC, ARR in a wide range of animals incl invertebrates

Eukaryotic cell lines

Policy information about cell lines and Sex and Gender in Research

Cell line source(s)	HepG2 (#300198), HEK293 (#300192), and MCF-7 (#300273) were derived from CLS Cell Lines Service GmbH, HT-29 from Leibniz institute DSMZ (#ACC 299), HepG2 ARE reporter cells from BPS Bioscience (#60513), and HMEpC from Sigma Aldrich (#830-05A). The HEK293 ARE/Nrf2 reporter cell line, as well as Nrf2 -/- knock-out HEK293 ARE/Nrf2 luciferase reporter cells and Catalase overexpressing HEK293 ARE/Nrf2 luciferase reporter cells were generated by us. We did not establish cell lines from primary cells.
Authentication	Cell lines derived from CLS Cell lines Service GmbH: STR profiling<br>HT-29 from Leibniz institute DSMZ: STR profiling<br>HepG2 ARE reporter cells from BPS Bioscience: by cellular functionality assay<br>HMEpC from Sigma Aldrich: STR profiling<br>Catalase overexpressing HEK293 ARE/Nrf2 luciferase reporter cells: PCR genotyping with species-specific primers
Mycoplasma contamination	All cell lines were tested negative for mycoplasma contamination by the supplier
Commonly misidentified lines (See ICLAC register)	No commonly misidentified cell lines were used in this study

Animals and other research organisms

Policy information about studies involving animals; ARRIVE guidelines recommended for reporting animal research, and Sex and Gender in Research

Laboratory animals	Mouse experiments were performed according to Art.18 Tierschutzgesetz (TSchG), Art. 141 Tierschutzverordnung (TschV), Art. 30 Tierversuchsverordnung (TVV) (all Switzerland) and were approved by the cantonal veterinary office Zürich, Switzerland. The studies were carried out on male and female C57BL/6NRj mice (Janvier Labs). The animals were housed in a specific pathogen-free environment at 22 °C with a reverse 12h/12h light/dark cycle with ad libitum excess to water and food.<br><br>Unless otherwise stated, C. elegans was cultured at 20°C on nematode growth media (NGM) seeded with E. coli OP50. Strains that were obtained from Caenorhabditis Genetics Center (University of Minnesota, USA) included Bristol N2 (wild type), EU31 skn-1 (zu135), RB754 aak-2 (ok524), GMC101 (dvls100). AM23 (rmIs298[pF25B3.3::Q19::CFP]) and AM716 (rmIs284[pF25B3.3::Q67::YFP]) strains were kindly provided by R. I. Morimoto. MIR257 risIs28[hsp-16.2p::CTL1::GFP + unc119(+)] was previously generated by us. Experiments were done with L4 stage nematodes until their death.
Wild animals	The study did not involve wild animals
Reporting on sex	Mouse studies were performed in both sexes. Sex-dependent parameter are shown in independent graphs (male, female). Sex-independent parameters were identified by two-way ANOVA analysis and combined in a single graph.
Field-collected samples	The study did not contain field-collected samples
Ethics oversight	Experiments were performed according to Art.18 Tierschutzgesetz(TSchG), Art.141 Tierschutzverordnung(TSchV),Art.30 Tierversuchsverordnung(TVV) and as approved by the cantonal veterinary office Zürich, Switzerland

Note that full information on the approval of the study protocol must also be provided in the manuscript.

Dual use research of concern

Policy information about dual use research of concern

Hazards

Could the accidental, deliberate or reckless misuse of agents or technologies generated in the work, or the application of information presented in the manuscript, pose a threat to:

No	Yes	
☒	☐	Public health
☒	☐	National security
☒	☐	Crops and/or livestock
☒	☐	Ecosystems
☒	☐	Any other significant area

Experiments of concern

Does the work involve any of these experiments of concern:

No	Yes	
☒	☐	Demonstrate how to render a vaccine ineffective
☒	☐	Confer resistance to therapeutically useful antibiotics or antiviral agents
☒	☐	Enhance the virulence of a pathogen or render a nonpathogen virulent
☒	☐	Increase transmissibility of a pathogen
☒	☐	Alter the host range of a pathogen
☒	☐	Enable evasion of diagnostic/detection modalities
☒	☐	Enable the weaponization of a biological agent or toxin
☒	☐	Any other potentially harmful combination of experiments and agents
